# Formononetin and Rhein from Bitong Mixture Alleviate Rheumatoid Arthritis-Related Inflammation: An Integrated WGCNA, Machine Learning and In Vitro Study

**DOI:** 10.3390/ph19050735

**Published:** 2026-05-07

**Authors:** Futing Tan, Jiangtao Wang, Keqing Fan, Runzi Gao, Zhibin Yang

**Affiliations:** 1 Yunnan Provincial Key Laboratory of Entomological Biopharmaceutical R&D, College of Pharmacy, Dali University, Dali 671003, China; 17869025994@163.com (F.T.); andrevvw@163.com (J.W.); fan18869805251@163.com (K.F.); 2 Nanjing Hospital of Chinese Medicine Affiliated to Nanjing University of Chinese Medicine, Nanjing 210001, China; 3 National-Local Joint Engineering Research Center of Entomoceutics, Dali 671003, China

**Keywords:** rheumatoid arthritis, formononetin, rhein, WGCNA, network pharmacology, machine learning, JUN, PPARG

## Abstract

**Background**: Bitong Mixture (BTM) is used clinically for rheumatoid arthritis (RA), but its active components and mechanisms remain unclear. **Methods**: WGCNA, network pharmacology, machine learning, and ROC analysis were integrated to identify candidate targets. We used molecular docking and molecular dynamics simulations for prioritization. In vitro validation was performed in TNF-α-stimulated MH7A cells. **Results**: Multiple analytical approaches consistently identified *JUN* and *PPARG*. Formononetin and Rhein showed favorable binding and stable interactions. Both compounds significantly reduced *IL6* and *MMP9* expression and suppressed fibroblast activation. **Conclusions**: BTM may exert anti-inflammatory effects through a JUN- and PPARG-related regulatory framework, supported by integrated computational and experimental evidence, providing a foundation for further in vivo investigation.

## 1. Introduction

Rheumatoid arthritis (RA) is a debilitating chronic autoimmune pathology characterized by synovial inflammation, articular destruction, and functional impairment [1,2]. The etiology of RA is multifactorial, involving a complex interplay of genetic predisposition, environmental triggers, and aberrant signal transduction pathways [3,4,5,6]. Pro-inflammatory cytokines, particularly tumor necrosis factor-alpha (TNF-α) and interleukin-6 (IL-6), are pivotal drivers of RA pathogenesis, orchestrating inflammatory cell infiltration, synovial hyperplasia, and progressive joint degradation [7,8]. Li et al. found that activation of PPAR-γ attenuates the TNF-α-induced inflammatory response in fibroblast-like synoviocytes (FLSs) and suppresses pathological FLS proliferation in rheumatoid arthritis [9]. Hannemann et al. reported that the c-Jun component of the AP-1 transcription factor is activated in RA synovium and macrophages and promotes inflammatory gene expression, thereby driving arthritis progression and joint destruction [10].

The Bitong Mixture (BTM; Jiangsu Drug Preparation Approval No. Z04000864) was prepared and supplied by Nanjing Municipal Hospital of Traditional Chinese Medicine (Nanjing, Jiangsu, China). is a well-established Traditional Chinese Medicine (TCM) formulation. Previous studies indicate that BTM attenuates secondary joint inflammation and reduces the production of pro-inflammatory cytokines, including interleukin-1β (IL-1β), interleukin-6 (IL-6), and tumor necrosis factor-α (TNF-α), effects that may be associated with suppression of the RIP140/NF-κB signaling axis in synovial tissue [11,12]. BTM consists of nineteen medicinal herbs, as detailed in Supplementary Table S1. Many of the medicinal herbs exhibit broad anti-inflammatory and immunomodulatory activities. For example, resveratrol, a major bioactive constituent of *Reynoutria japonica*, has been shown to alleviate synovial inflammation and bone destruction by inhibiting key pro-inflammatory signaling pathways, including NF-κB and MAPK, and by downregulating inflammatory cytokines such as TNF-α, IL-1β, and IL-6 [13,14,15]. Meanwhile, glycyrrhizin, a representative active component of *Glycyrrhiza uralensis*, exerts anti-inflammatory and immunomodulatory effects primarily through inhibition of high-mobility group box 1 (HMGB1) and blockade of the HMGB1-mediated TLR4/NF-κB signaling axis [16,17,18]. These findings support its therapeutic potential, yet the specific active constituents and molecular mechanisms responsible for BTM’s effects remain to be clarified. BTM was selected for this study because it is a clinically used formulation with reported anti-inflammatory effects in RA-related conditions. Unlike conventional anti-rheumatic drugs such as methotrexate, leflunomide, or anti-TNF biologics, the present work does not aim to compare therapeutic efficacy. Instead, it addresses a different question: which bioactive components and molecular targets underlie the effects of BTM? This represents a key knowledge gap for this formulation and provides the rationale for the current mechanistic investigation.

Advances in bioinformatics and network pharmacology enable systematic investigation of multi-component mechanisms [19,20]. In RA research, high-throughput transcriptomic profiling and data-mining analyses facilitate the delineation of gene signatures associated with disease progression and therapeutic responsiveness, a direction strengthened by the growing availability of public datasets and increasingly standardized analytical workflows. By integrating these layers of evidence, researchers can construct a molecular framework for RA management that captures the multi-target features inherent to complex prescriptions.

In this study, we integrated network pharmacology, WGCNA, machine learning, molecular docking, and molecular dynamics simulations to identify candidate bioactive compounds and targets of BTM in RA. In addition, in vitro experiments using TNF-α-stimulated MH7A cells were conducted to evaluate anti-inflammatory effects. This approach provides a systematic view of BTM mechanisms.

**Figure 1:**
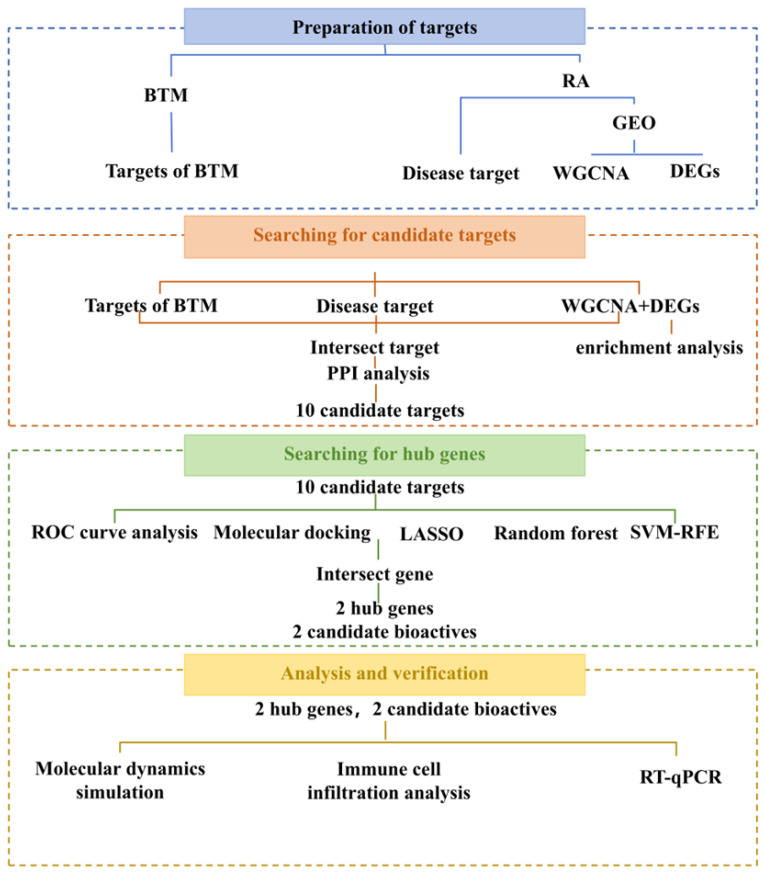
Schematic overview of the analytical and experimental workflow used in this study, including data processing, WGCNA, machine learning, network pharmacology, molecular docking, molecular dynamics simulation, and in vitro validation.

An overview of the analytical and experimental workflow is provided in Figure 1 to facilitate understanding of the study design.

## 2. Results

### 2.1. Identification of BTM Active Ingredients and RA Therapeutic Targets

To systematically characterize the pharmacological basis of BTM, we screened its constituents using strict ADME thresholds. A total of 242 active ingredients were obtained after screening with oral bioavailability (OB) ≥ 30% and drug-likeness (DL) ≥ 0.18, comprising 224 compounds from the TCMSP database and 18 additional entries curated from the HERB database (Supplementary Tables S2-1 and S2-2). Given that downstream network construction depends on reliable ingredient–target links, we applied target prediction to this ingredient set, yielding 1409 non-redundant putative targets (Supplementary Table S3).

To reduce the bias and incompleteness inherent to any single repository, we integrated RA-related therapeutic targets across four curated resources. GeneCards and DisGeNET entries were filtered at the median relevance/association score, yielding 1482 and 1567 targets, respectively, while TTD and DrugBank contributed 100 and 453 targets; all records were harmonized to UniProt-standardized gene symbols. Merging the four lists and removing duplicates produced a nonredundant set of 2289 RA-associated targets for downstream network analyses (Supplementary Table S4).

### 2.2. Data Harmonization and DEG Identification

**Figure 2:**
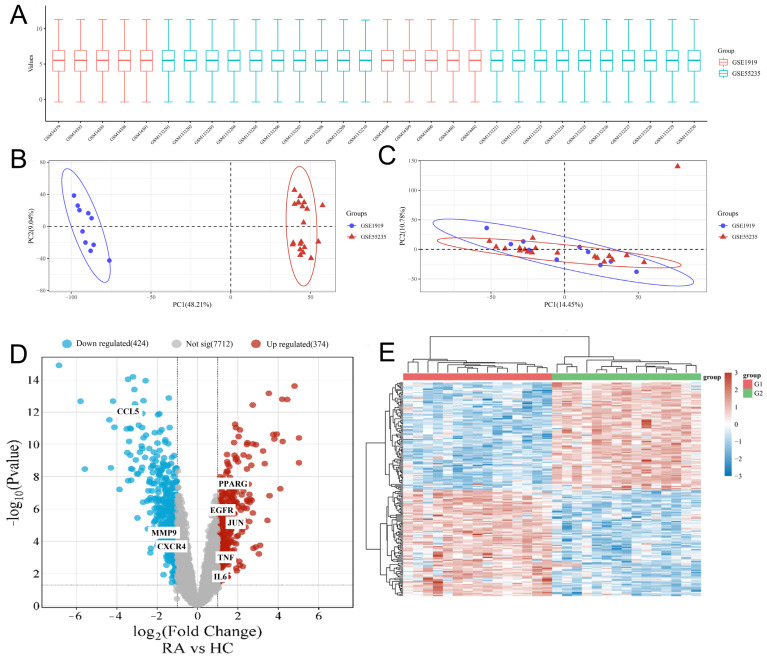
Data cleaning and preprocessing. (**A**) Boxplot showing the distribution of gene expression values after normalization. (**B**) Principal component analysis (PCA) of multiple datasets before batch-effect correction. (**C**) PCA plot following batch-effect removal, demonstrating improved dataset harmonization. (**D**) Volcano plot of differentially expressed genes. (**E**) Heatmap illustrating differential gene expression patterns across samples.

To support reliable cross-cohort inference, we normalized and batch-adjusted expression profiles from GSE1919 and GSE55235. PCA analysis of the corrected matrix showed that the dominant batch-driven structure was largely removed, with RA and control samples forming well-defined, diagnosis-consistent clusters and exhibiting clear group separation (Figure 2A–C). Using a |log_2_FC| > 1 and *p* < 0.05 threshold, differential expression analysis identified 798 DEGs, including 374 upregulated and 424 downregulated genes (Figure 2D and Supplementary Table S5). Consistent with these global shifts, the top 50 DEGs displayed sharply contrasting expression patterns between RA and control cohorts, underscoring pronounced between-group transcriptional heterogeneity (Figure 2E).

### 2.3. Functional Enrichment Analysis of RA-Related Signatures

**Figure 3:**
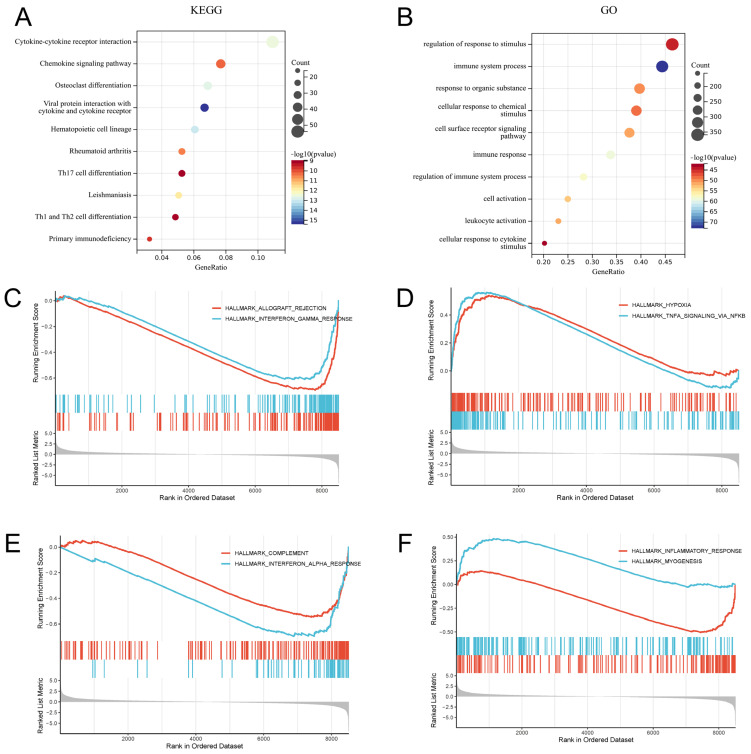
Enrichment analysis of differentially expressed genes. (**A**) KEGG pathway enrichment bubble plot. (**B**) GO functional enrichment bubble plot. (**C**–**F**) GSEA enrichment plots of the top eight pathways; vertical bars indicate the positions of genes in the ranked list, and the grey area at the bottom represents the ranked list metric (ranking score). (**C**) Allograft rejection and interferon-γ response pathways. (**D**) TNFα signaling via NF-κB and hypoxia pathways. (**E**) Complement and interferon-α response pathways. (**F**) Inflammatory response and myogenesis pathways.

GO enrichment profiling placed the DEGs primarily within immune-regulatory programs. Within the BP domain, the most significant terms centered on immune system processes, leukocyte activation, and regulation of immune responses, while strong signals were also observed for cellular responses to chemical and cytokine stimuli. This pattern aligns with the cytokine-rich inflammatory microenvironment that typifies RA and suggests that the transcriptional changes captured here largely reflect ongoing immune activation (Figure 3B and Supplementary Table S6).

KEGG enrichment added pathway-level resolution to the DEG signals and linked them to well-described RA pathogenic modules. The “Rheumatoid arthritis” pathway was among the most significantly enriched terms, supporting the disease relevance of the transcriptional signature captured in this analysis. DEGs were also concentrated in core immune-communication routes, including “Cytokine-cytokine receptor interaction” and the “Chemokine signaling pathway,” consistent with coordinated leukocyte trafficking into inflamed synovial tissue. Pathways governing immune lineage commitment—“Th1 and Th2 cell differentiation” and “Th17 cell differentiation”—were similarly overrepresented, and the parallel enrichment of “Osteoclast differentiation” points to coupled inflammatory and bone-destructive programs that underlie synovitis and progressive erosion (Figure 3A and Supplementary Table S7).

In parallel with the over-representation analysis, we used GSEA to capture coordinated pathway shifts without reliance on a fixed differential-expression cutoff. The enrichment pattern recapitulated the GO and KEGG findings, with prominent immune- and inflammation-related hallmarks such as “TNF-α signaling via NF-κB”, “Interferon-γ response”, and the “Inflammatory response”. Notably, the concurrent enrichment of “Complement” and “Hypoxia” gene sets points to a convergence of innate immune activation and the oxygen-limited synovial niche, a microenvironmental context that is increasingly recognized as shaping inflammatory persistence in RA (Figure 3C–F and Supplementary Table S8).

### 2.4. WGCNA Module Identification

**Figure 4:**
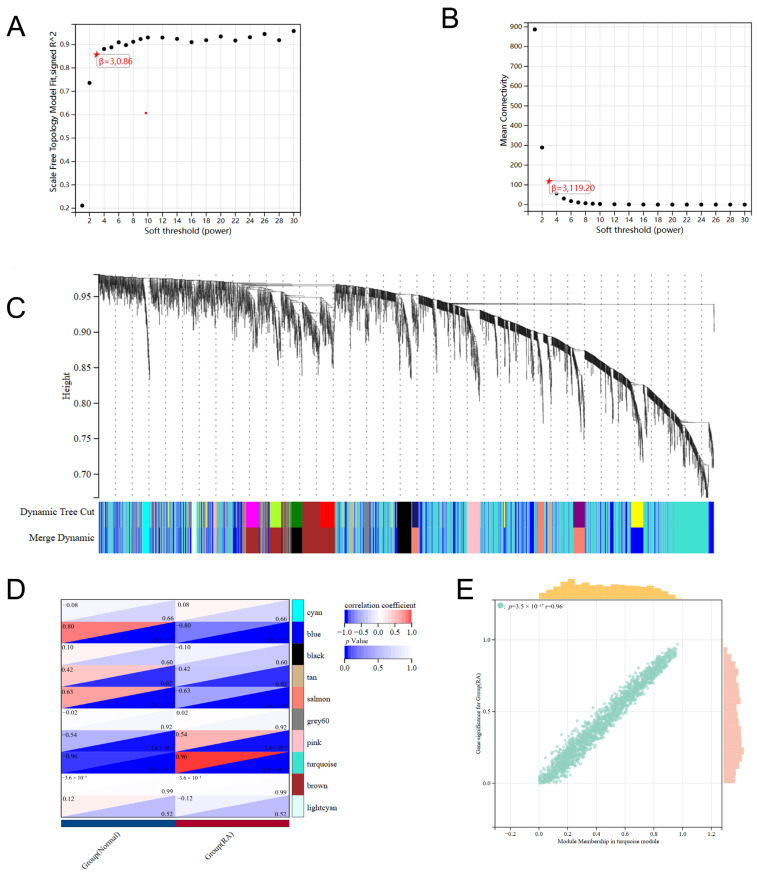
Weighted gene co-expression network analysis. (**A**) Scale-free topology fit index across soft-thresholding powers, with β = 3 identified as the selected threshold. The resulting R^2^ value of 0.86 was considered acceptable given the sample size. (**B**) Average connectivity under different soft-thresholding powers. (**C**) Sample clustering dendrogram and module detection, yielding ten co-expression modules. (**D**) Module–trait correlation heatmap showing associations between modules and clinical features. (**E**) Scatter plot of gene significance (GS) versus module membership (MM). Different colors are used for the scatter points and the marginal distributions solely to improve readability; the colors are not mapped to any variable or category.

We used Weighted Gene Co-expression Network Analysis (WGCNA) to link gene co-expression modules with clinical traits. We selected a soft-thresholding power of β = 3, yielding a scale-free topology fit index of 0.86. Although this value did not exceed 0.90, it was considered acceptable given the limited sample size and preserved sufficient network connectivity for downstream analysis (Figure 4A,B). We identified ten distinct co-expression modules (Figure 4C). Notably, among the identified co-expression modules, the turquoise module showed the most pronounced positive association with the RA phenotype (r = 0.68; Figure 4D). On this basis, we selected 170 genes from this module as RA-relevant hub candidates using the criteria of Module Membership ≥ 0.8 and Gene Significance ≥ 0.1 (Figure 4E; Supplementary Table S9).

### 2.5. Construction of the Herb–Ingredient–Target Network

**Figure 5:**
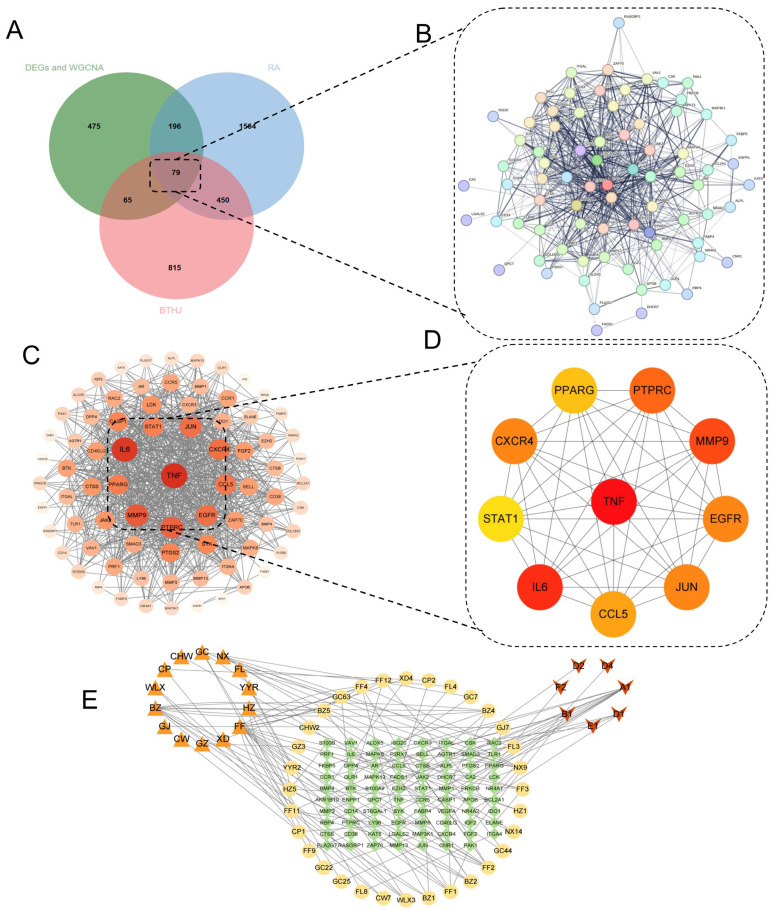
Construction of the protein–protein interaction network and identification of candidate targets. (**A**) Venn diagram showing the overlap between BTM-RA-DEGs and key module genes. (**B**) STRING-based PPI network. (**C**) Cytoscape visualization of the integrated network, where darker colors indicate higher connectivity. (**D**) Top ten candidate targets ranked by degree. (**E**) Herb–ingredient–target network of BTM against RA. Nodes represent herbs, active ingredients, and candidate targets; edges represent ingredient–target associations. Orange triangles denote herbs, yellow circles denote herb-specific active ingredients (present in only one herb), red V-shaped nodes denote shared active ingredients (present in ≥2 herbs), and green diamonds denote candidate targets (overlapping genes).

Identifying the molecular interface between therapeutic compounds and disease-related targets is critical for elucidating the mechanism of action of BTM. By intersecting key gene sets—including the WGCNA turquoise module, DEGs, RA-related targets, and putative BTM targets—we identified 79 overlapping genes (Figure 5A). These genes constitute the core “Drug–Disease” interactome, representing the most promising candidates for therapeutic intervention.

To examine whether these 79 targets might function together, we used the STRING database to build a protein–protein interaction (PPI) network describing their putative connections. We limited the analysis to *Homo sapiens*, and we included only interactions supported by experimental data or curated database annotations. We applied a minimum confidence score of 0.4 to retain associations with reasonable support while avoiding an overly sparse network. We visualized the network using Cytoscape 3.9.1, and the network topology revealed a dense interconnectivity (Figure 5B,C), suggesting that BTM operates through a synergistic multi-target mechanism rather than by modulating isolated proteins. By evaluating the degree centrality, ten hub targets emerged as the most critical nodes within this network: *TNF*, *IL6*, *MMP9*, *PTPRC*, *JUN*, *CXCR4*, *EGFR*, *CCL5*, *PPARG*, and *STAT1* (Figure 5D and Supplementary Table S10). These hubs are centrally positioned in the interactome, indicating their potential roles as master regulators of the BTM-mediated therapeutic response.

To connect the candidate molecular targets with the pharmacologically active constituents of BTM, we assembled a “Herb–Ingredient–Target” network (Figure 5E; Supplementary Table S11). The network contains 132 nodes and 135 edges, linking herbs (orange triangles), active ingredients (yellow circles and red V-shapes), and their corresponding gene targets (green diamonds). Several ingredients—A1, B1, D1, D2, D4, E1, and F2—emerged as shared components across multiple herbs, indicating a substantial degree of overlap in chemical composition. The resulting topology supports the view that BTM exerts therapeutic effects in RA through a coordinated, multi-component and multi-target mode of action rather than via a single dominant constituent.

### 2.6. Functional Expansion via GeneMANIA

We identified ten additional genes for each central target (Supplementary Figure S1), yielding a GMFA Expanded Gene List (GMFA-ED) consisting of 98 genes (Supplementary Table S12). GO and KEGG enrichment analyses of GMFA-ED (Supplementary Tables S13–S16) highlighted functions related to immune regulation, inflammatory response, and tissue repair (Supplementary Figure S1). GO biological process terms were enriched for cell growth, signal transduction, and gene-expression regulation (Supplementary Figure S2A). In the cellular component category, the enriched terms mainly involved membrane raft, membrane microdomain, and the external surface of the plasma membrane (Supplementary Figure S2B), while molecular-function terms were strongly associated with chemokine receptor-mediated signaling (Supplementary Figure S2C). KEGG analysis indicated enrichment in RA-related pathways, particularly TNF and IL-17 signaling (Supplementary Figure S2D). Notably, the cytokine–cytokine receptor interaction signaling pathway exhibited extensive enrichment, a phenomenon that was not apparent in previous analyses of intersecting targets. This finding suggests that GMFA-ED may capture additional pathway-level features. This expansion may indicate that BTM is associated with a broader inflammatory network rather than discrete targets. GeneMANIA expansion may include genes with indirect associations; therefore, these results should be considered as supportive and hypothesis-generating.

### 2.7. Screening of Core Therapeutic Targets and Compounds

**Figure 6:**
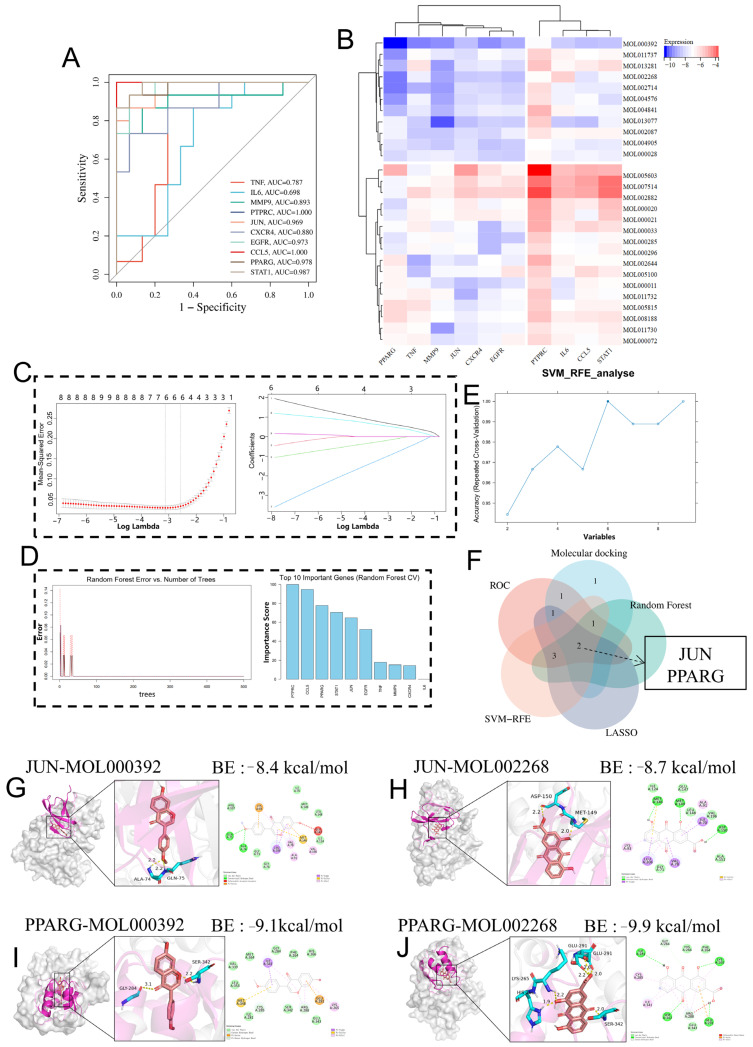
Hub target screening. (**A**) ROC curves of ten candidate targets showing their discriminatory ability in the analyzed datasets. (**B**) Heatmap showing docking scores between 27 BTM active components and the ten targets. (**C**) LASSO regression with 10-fold cross-validation indicating the optimal λ with minimum mean squared error. Colors are for visualization only. (**D**) Random Forest error curve and top 10 genes by importance. (**E**) SVM-RFE accuracy curve for selecting the optimal feature set. (**F**) Venn diagram showing the overlap of candidate genes identified by ROC analysis, molecular docking, and three machine learning methods (LASSO, Random Forest, and SVM-RFE). *JUN* and *PPARG* were identified as common genes across all methods. (**G**–**J**) 3D docking conformations of key compound–target pairs.

To prioritize key targets and compounds, we applied an integrative screening strategy. The ten candidate genes were first evaluated by ROC analysis. As shown in Figure 6A, all genes exhibited some discriminatory ability, and several genes, including *MMP9*, *JUN*, *CXCR4*, *CCL5*, *PTPRC*, and *PPARG*, showed relatively higher AUC values. These genes were therefore retained as candidate features for subsequent prioritization rather than definitive diagnostic markers.

Next, we performed molecular docking analysis to evaluate the interactions between the top 27 active ingredients of BTM (Supplementary Table S17) and these prioritized targets. We retrieved high-resolution crystal structures for the ten hub targets, including *TNF*, *IL6*, *MMP9*, *PTPRC*, *JUN*, *CXCR4*, *EGFR*, *CCL5*, *PPARG*, and *STAT1,* to serve as docking receptors (full PDB list in Supplementary Table S18). The docking results, visualized as a clustered heatmap in Figure 6B, highlighted a group of eleven compounds with relatively favorable binding affinities across six targets, namely *PPARG*, *TNF*, *MMP9*, *JUN*, *CXCR4*, and *EGFR*. Among these compounds, Formononetin (MOL000392) and Rhein (MOL002268) were prioritized because they showed relatively strong and consistent binding patterns. Derived from the principal BTM herbs *Scutellaria baicalensis* and *Polygonum cuspidatum*, respectively, these two compounds were identified as candidate bioactives for subsequent therapeutic analysis.

To prioritize key genes, we applied three machine learning methods. LASSO regression identified six genes with non-zero coefficients (Figure 6C). Random Forest ranked candidate targets based on importance and showed stable performance as the number of trees increased (Figure 6D). SVM-RFE reached optimal accuracy when 7–8 features were retained (Figure 6E).

Ultimately, JUN and PPARG were retained using a stringent intersection strategy. Specifically, we used a Venn diagram to identify genes consistently shared across ROC analysis, molecular docking, and three machine learning algorithms (LASSO, Random Forest, and SVM-RFE). Only genes present in all methods were selected as final candidates to increase robustness while acknowledging the possibility of excluding relevant genes (Figure 6F). These two genes were therefore prioritized as candidate key nodes within the inferred therapeutic network. Further docking visualization showed plausible spatial compatibility between Formononetin, Rhein, and the binding pockets of *JUN* and *PPARG* (Figure 6G–J), providing structural support for these candidate interactions. Detailed performance metrics for the three machine learning models are provided in Supplementary Table S19.

### 2.8. Experimental and Simulation-Based Validation

**Figure 7:**
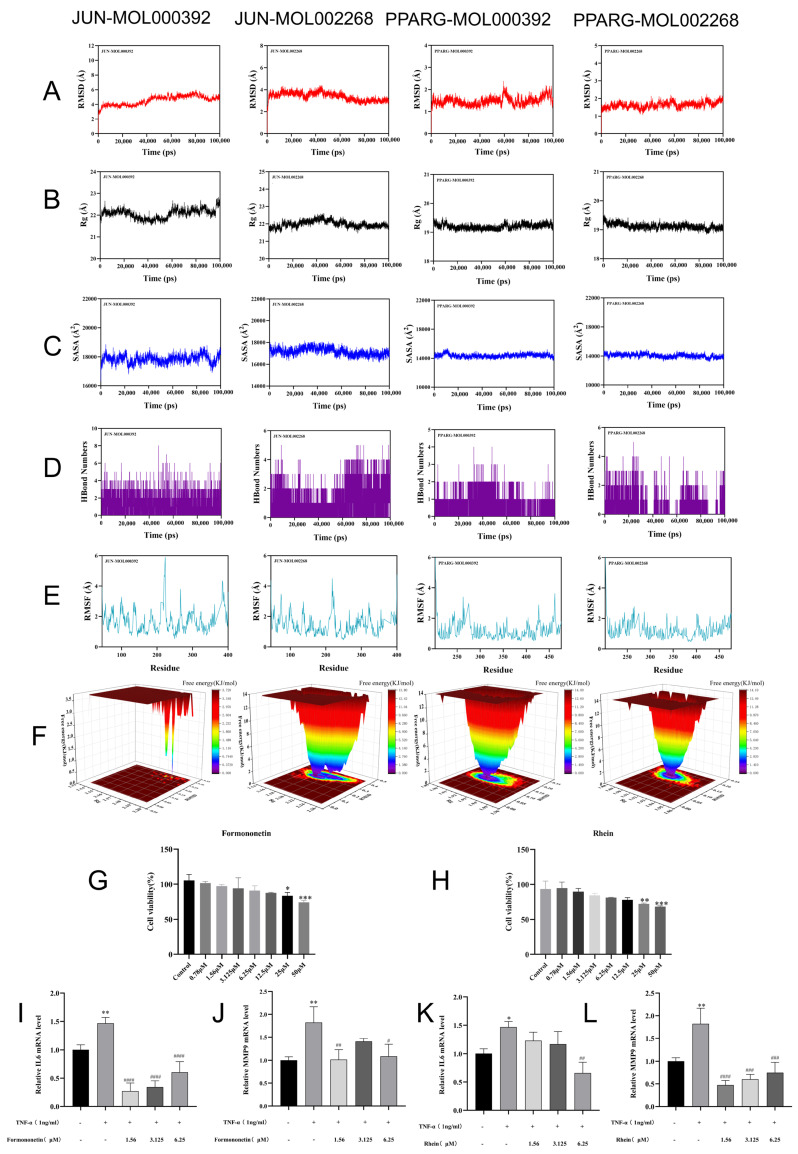
Molecular dynamics simulation and experimental validation of Formononetin and Rhein binding to *JUN* and *PPARG*. (**A**) RMSD trajectories of protein–ligand complexes over 100 ns. (**B**) Radius of gyration (Rg) plots. (**C**) Solvent-accessible surface area (SASA) plots. (**D**) Hydrogen bond interaction profiles during simulation. (**E**) RMSF plots of protein residues. (**F**) Free-energy landscape analysis. (**G**,**H**) Effects of Formononetin and Rhein on MH7A cell viability determined by MTT assay. (**I**–**L**) mRNA expression levels of *IL6* and *MMP9* in TNF-α-induced MH7A cells following treatment with Formononetin or Rhein at 1.56, 3.125, and 6.25 μM. Significant differences relative to the control or model group are indicated. Data are expressed as mean ± SD (*n* = 3). * *p* < 0.05, ** *p* < 0.01, *** *p* < 0.001 vs. control; # *p* < 0.05, ## *p* < 0.01, ### *p* < 0.001, #### *p* < 0.0001 vs. TNF-α model group.

To assess the structural stability and dynamic behavior of the *JUN*–MOL000392 (Formononetin), *JUN*–MOL002268 (Rhein), *PPARG*–MOL000392 (Formononetin), and *PPARG*–MOL002268 (Rhein) complexes, we performed 100 ns molecular dynamics simulations and analyzed RMSD, radius of gyration (Rg), solvent-accessible surface area (SASA), hydrogen-bond occupancy, RMSF, and free-energy landscape (FEL) (Figure 7A–F). The RMSD trajectories indicated that all complexes reached and maintained stable conformations, suggesting a relatively rigid binding environment during the simulations (Figure 7A). *JUN*–MOL000392 showed comparatively larger deviations, fluctuating mainly within 3.5–5.5 Å and approaching a stable plateau after approximately 40 ns. *JUN*–MOL002268 exhibited improved stability, with RMSD values largely confined to 2.5–4.0 Å. In contrast, both *PPARG* complexes displayed consistently lower RMSD values throughout the 100 ns, with *PPARG*–MOL000392 remaining within 1.2–2.2 Å and *PPARG*–MOL002268 within 1.4–2.0 Å, suggesting a more rigid and favorable binding environment in *PPARG* compared with *JUN*.

The Rg profiles further supported these observations by showing no marked global expansion or collapse in any system (Figure 7B). *JUN*–MOL000392 maintained Rg values of 21.8–22.6 Å, while *JUN*–MOL002268 remained within 21.8–22.3 Å, indicating an overall compact fold despite moderate backbone fluctuations. Both *PPARG* systems were even more compact and stable, with Rg values consistently distributed around 19.0–19.5 Å. In agreement with the Rg results, SASA fluctuated within narrow ranges over the trajectories (Figure 7C), including 18,000–20,000 Å^2^ for *JUN*–MOL000392 and 16,500–18,000 Å^2^ for *JUN*–MOL002268, whereas *PPARG*–MOL000392 and *PPARG*–MOL002268 showed lower and more stable SASA values of 14,000–15,000 Å^2^ and 13,500–14,500 Å^2^, respectively, reflecting limited changes in solvent exposure and overall structural integrity.

Hydrogen-bond analysis revealed sustained ligand–protein interactions in all complexes, although with system-dependent occupancy (Figure 7D). *JUN*–MOL000392 maintained approximately 2–4 hydrogen bonds for most of the simulation. *JUN*–MOL002268 typically formed 1–3 hydrogen bonds and displayed an increase to 4–5 hydrogen bonds during the later stage, suggesting enhanced intermolecular contacts over time. *PPARG*–MOL000392 showed 0–2 hydrogen bonds, whereas *PPARG*–MOL002268 maintained 1–2 hydrogen bonds with relatively stable persistence, indicating steady but fewer polar contacts compared with the *JUN* systems.

At the residue level, RMSF profiles showed that the majority of residues in each protein fluctuated mildly (<2 Å), with only localized flexible regions displaying higher peaks (Figure 7E). *JUN* exhibited more pronounced local motions, with peaks reaching 4–6 Å, whereas *PPARG* showed smaller peaks of approximately 3–4 Å, suggesting that *PPARG* remained globally more rigid while *JUN* contained several flexible segments. Finally, the FEL maps for all systems displayed a dominant low-energy basin (Figure 7F), suggesting that each complex predominantly sampled a single stable and energetically favorable conformational state during the simulations. Taken together, these results showed generally stable trajectories over 100 ns, with the *PPARG* complexes showing stronger overall structural stability, while *JUN*–MOL000392 displayed relatively higher flexibility and *JUN*–MOL002268 exhibited improved stabilization, particularly in the later stage of the trajectory.

To complement the in silico analyses, we conducted in vitro experiments in MH7A synovial fibroblasts to evaluate the effects of the selected compounds on cell proliferation and inflammatory responses. The effects of the compounds on cell proliferation were first evaluated using MTT assays, as shown in Figure 7G,H. The results demonstrated a dose-dependent inhibitory effect. However, IC50 values were not formally calculated due to the limited concentration range, which did not allow accurate curve fitting across a full dose–response relationship. At higher concentrations, Formononetin and Rhein significantly suppressed MH7A cell proliferation, suggesting potential activity against synovial hyperplasia-related processes in RA. In contrast, lower concentrations exhibited minimal cytotoxicity and were therefore selected for subsequent inflammatory assays. Using these non-cytotoxic concentrations (1.56, 3.125, and 6.25 μM), we further investigated the anti-inflammatory effects of Formononetin and Rhein in a TNF-α-induced model. As shown in Figure 7I–L, TNF-α stimulation precipitated a dramatic upregulation of *IL6* and *MMP9* mRNA levels compared to the control (*p* < 0.05 or *p* < 0.01). However, intervention with Formononetin and Rhein significantly attenuated this transcriptional surge. Compared with the model group, both compounds significantly reduced the expression of *IL6* and *MMP9* within the tested concentration range (*p <* 0.05 to *p <* 0.0001) without obvious cytotoxicity. These findings support the view that Formononetin and Rhein are candidate active components of BTM with potential anti-inflammatory effects in MH7A cells.

### 2.9. Immune Infiltration and Correlation Among Potential Targets

**Figure 8:**
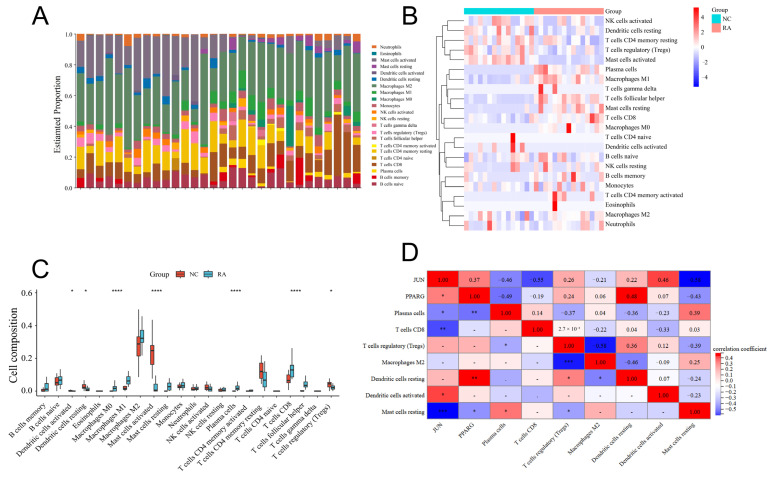
Immune infiltration analysis. (**A**) Stacked bar chart showing the proportional composition of 22 immune cell subsets across all samples, with different colors representing distinct immune cell types. (**B**) Heatmap of immune infiltration levels comparing the NC and RA groups, illustrating differential enrichment patterns among the 22 immune cell subsets. (**C**) Violin plots displaying the distribution of immune-cell infiltration scores between NC and RA samples. Significant differences in specific immune cell subsets are indicated (* *p* < 0.05, **** *p* < 0.0001, values smaller than 1 × 10^−4^ are also denoted as “****”). (**D**) Correlation analysis between significantly altered immune cell and the hub genes PPARG and JUN, suggesting potential immunological relevance in RA. * *p* < 0.05, ** *p* < 0.01, *** *p* < 0.01.

CIBERSORT immune-infiltration analysis revealed distinct immune-cell composition patterns across samples, with higher proportions of Macrophages M2, activated Mast cells, and CD8^+^ T cells (Figure 8A). The heatmap further illustrated differential immune-cell distributions between RA and healthy controls (Figure 8B). Wilcoxon analysis identified seven immune-cell subsets with significant differences (Figure 8C). Correlation analyses revealed that *JUN* exhibited inverse relationships with plasma cells, CD8^+^ T lymphocytes, and quiescent mast cells, whereas positive associations were observed with both resting and activated dendritic cell populations. *PPARG* exhibited negative correlations with plasma cells and resting Mast cells, and a positive correlation with resting dendritic cells (Figure 8D).

## 3. Discussion

Rheumatoid arthritis (RA) is a complex autoimmune disorder characterized by persistent synovial inflammation and progressive joint damage, largely driven by fibroblast-like synoviocytes (FLSs) that adopt an aggressive, tumor-like phenotype [21,22,23,24,25]. Despite the availability of immunomodulatory therapies, effectively targeting the molecular programs that sustain FLS activation remains challenging.

In this study, we used a systems pharmacology approach to identify bioactive compounds with potential therapeutic relevance in RA and to explore their underlying molecular mechanisms. Starting from 242 candidate compounds and 2289 RA-associated targets, we combined network-based target prediction, weighted gene co-expression network analysis (WGCNA), and ensemble machine learning (LASSO, Random Forest, SVM-RFE) with exploratory ROC analysis in the discovery dataset to assess the discriminatory potential of candidate genes. Our analysis identified *JUN* and *PPARG* as candidate key nodes, suggesting their involvement in inflammatory and proliferative pathways in RA. To reduce potential bias arising from database-specific predictions, we focused on overlapping targets identified across multiple databases. The herb–ingredient–target network was primarily constructed to visualize potential relationships rather than to perform detailed topological analysis. Therefore, the network should be interpreted as a descriptive framework rather than a quantitative measure of network structure. *JUN* and *PPARG* were identified as key candidate targets based on network analysis. Importantly, these core targets were identified from the integrated analysis of WGCNA, differential expression, and machine learning results, rather than from the GeneMANIA-expanded network. However, this identification is based on network topology and does not establish causal regulatory roles. Further functional validation is required to confirm their roles in RA pathogenesis. The overlapping strategy was used to focus on consistently identified targets across multiple data sources, which helps to improve robustness. However, this approach may still be influenced by database-specific bias and therefore represents a candidate gene set rather than definitive biological targets. The ROC analysis was performed on the same datasets used for gene identification and therefore reflects internal evaluation rather than independent validation. Thus, the reported AUC values may be overestimated and should be interpreted with caution. In this study, ROC analysis was used to support candidate gene selection rather than to establish a clinically applicable diagnostic model. The three machine learning methods used in this study represent different algorithmic principles, including linear regularization (LASSO), ensemble learning (Random Forest), and recursive feature elimination (SVM-RFE). The consistency of results across these methods improves robustness and reduces the likelihood of method-specific bias. Although the scale-free topology fit index did not reach the commonly recommended threshold of 0.90, the selected soft-thresholding power produced an acceptable scale-free topology fit while preserving network connectivity [26].

Our integrative analysis suggests that *JUN* and *PPARG* may participate in a functionally relevant regulatory relationship in RA pathology. These two factors may play opposite roles in synovial fibroblasts. JUN has been reported to be associated with inflammation and matrix degradation, including the upregulation of MMP-9, which contributes to the invasive behavior of fibroblast-like synoviocytes in RA [27,28]. In contrast, PPARG is associated with anti-inflammatory effects and can suppress the expression of inflammatory genes [9]. These findings suggest that BTM may be associated with rebalancing inflammatory signaling toward a less activated state. Formononetin and Rhein may contribute to this effect by potentially acting on both sides of this regulatory system, as supported by the docking and molecular dynamics results. This observation may help explain the changes observed in immune cell infiltration. It also suggests that BTM may influence both signaling pathways and the immune environment in RA. Importantly, these findings support a network-level mode of action rather than a single-target mechanism. Our KEGG enrichment analysis highlighted the cytokine–cytokine receptor interaction pathway, which is relatively broad. These pathway-level findings are consistent with the immune infiltration patterns observed in the CIBERSORT analysis. The GeneMANIA-expanded network includes indirect associations such as co-expression and genetic interactions. Therefore, these interactions should be interpreted as providing functional context rather than direct mechanistic evidence. In addition, immune infiltration analysis suggested that the observed correlations between JUN/PPARG and specific immune cell populations, particularly dendritic cells, may have potential clinical implications in RA. Dendritic cells play a critical role in antigen presentation and the activation of adaptive immune responses, thereby contributing to chronic inflammation in RA [29]. However, these observations are based on computational inference and require further experimental validation.

Experimental findings for Formononetin and Rhein were consistent with the computational predictions. Although no clinically established JUN inhibitor or PPARG agonist was included as a positive control in the docking analysis, the relative binding patterns observed for Formononetin and Rhein were consistent across multiple targets, suggesting comparative reliability within the screening framework. Our molecular dynamics simulations suggested relatively stable interactions with *JUN* and *PPARG*, and our in vitro assays indicated concentration-dependent effects: higher doses inhibited aberrant FLS proliferation, while lower, non-cytotoxic doses suppressed TNF-α-induced *IL6* and *MMP9* expression. The concentrations used in vitro (0.78–50 μM) were selected to assess dose-dependent responses under controlled conditions. In addition, the present study does not include pharmacokinetic or tissue distribution data; therefore, whether Formononetin and Rhein reach synovial tissue at pharmacologically relevant concentrations remains to be determined. The concentrations used in vitro do not necessarily reflect those achievable in vivo following oral administration. Accordingly, the proposed mechanisms should be interpreted at the cellular level and require further validation in in vivo models. Therefore, the observed dose–response relationship should be interpreted as an indicator of cellular responsiveness rather than a direct measure of clinical efficacy. These findings suggest that specific bioactives can simultaneously modulate synovial hyperplasia and inflammatory mediator production, providing supportive evidence for their potential therapeutic relevance in RA. Previous studies have reported that BTM or its constituents may act through pathways such as RIP140/NF-κB signaling. In this context, the JUN/PPARG axis identified in the present study may partially overlap with these pathways while also providing a complementary perspective that links inflammatory signaling with metabolic regulation at the network level. Thus, rather than representing an entirely independent mechanism, our findings may extend existing knowledge by integrating these pathways within a systems-level framework.

Several limitations should be acknowledged. In this study, an unsigned network was used, which does not distinguish the direction of correlations. Although this approach captures a broader range of gene associations, it may overlook biologically meaningful differences between positive and negative correlations. The ADME-based screening criteria (OB ≥ 30% and DL ≥ 0.18) may exclude certain bioactive compounds with low oral bioavailability but potential local or tissue-specific activity. Therefore, the screening criteria do not necessarily reflect actual in vivo exposure or bioavailability in synovial tissues. We conducted ROC analysis on the discovery dataset without external validation. The use of two transcriptomic datasets (GSE1919 and GSE55235) may limit the generalizability of the findings. These datasets were selected due to comparable platforms and sample characteristics, and batch effects were corrected prior to integration. In addition, the use of overlapping strategies across WGCNA, differential expression, and machine learning was intended to improve internal consistency. Nevertheless, validation in independent cohorts is required to confirm the reproducibility of these findings. Finally, while MD simulations and in vitro experiments provide supportive evidence, in vivo causal relationships remain to be established. Target prediction in this study is based on computational inference and should be interpreted as hypothesis-generating rather than definitive evidence of direct molecular interactions. To reduce potential false-positive results, we integrated multiple databases and analytical methods, including WGCNA and machine learning, and focused on overlapping targets across approaches. However, these thresholds are heuristic and may include genes with varying levels of association, and therefore should be interpreted with caution. In addition, MH7A cells, although widely used, do not fully recapitulate the heterogeneity and behavior of primary fibroblast-like synoviocytes derived from RA patients. Experimental validation focused on two representative compounds and a single cell line. Therefore, the findings support the activity of these selected compounds but do not fully reflect the effects of the entire BTM or potential synergistic interactions among its components. This study did not directly assess JUN/PPARG protein levels, transcriptional activity, or causal dependence; therefore, the proposed “axis” should be interpreted as a candidate regulatory framework at the network level. Future studies should include protein-level validation (e.g., Western blotting, ELISA, or immunofluorescence) to confirm these findings. In parallel, using models lacking JUN or PPARG and applying specific pathway inhibitors or activators will help determine whether the observed effects truly depend on the JUN/PPARG axis.

## 4. Materials and Methods

### 4.1. Bioactive Component Filtering and Target Acquisition for BTM

Candidate constituents of BTM were collected primarily from the Traditional Chinese Medicine Systems Pharmacology (TCMSP) database (http://tcmsp-e.com/, version 2.3, accessed on 1 April 2024). Compounds were filtered using oral bioavailability (OB) ≥ 30% and drug-likeness (DL) ≥ 0.18 as the inclusion thresholds [30]. These thresholds are widely used in network pharmacology studies to prioritize compounds with potential oral bioavailability and drug-like properties. In addition, the Herb database (http://herb.ac.cn/, version 2.0, accessed on 2 April 2024) was consulted to complement the active-component information for *Cibotium barometz* and *Eupolyphaga sinensis* [31]. To improve target coverage, putative protein targets associated with the retained components were compiled from four resources, including TCMSP, SwissTargetPrediction (http://www.swisstargetprediction.ch/, version 2019, accessed on 5 April 2024) [32], the Similarity Ensemble Approach (SEA; https://sea.bkslab.org/, accessed on 5 April 2024) [33], and PharmMapper (http://www.lilab-ecust.cn/pharmmapper/, accessed on 5 April 2024) [34]. All target protein identifiers were subsequently mapped to official gene symbols using UniProt (https://www.uniprot.org/, accessed on 6 April 2024) [35].

### 4.2. Acquisition of RA-Related Therapeutic Targets

Rheumatoid arthritis (RA)-related therapeutic targets were collected by searching DisGeNET (https://www.disgenet.org/, accessed on 10 April 2024) [36], GeneCards (https://www.genecards.org/, accessed on 10 April 2024) [37], DrugBank (https://go.drugbank.com/, accessed on 10 April 2024) [38], and TTD (https://db.idrblab.net/ttd/, accessed on 10 April 2024) [39] with the terms “RA” and “rheumatoid arthritis”. In parallel, the Gene Expression Omnibus (GEO; https://www.ncbi.nlm.nih.gov/geo/, accessed on 15 April 2024) [40] was screened for eligible transcriptomic datasets, and GSE1919 and GSE55235 were retained (Supplementary Table S20). The raw data and series matrix files were retrieved, after which differential expression was assessed in R (v4.3.3, R Foundation for Statistical Computing, Vienna, Austria) using the limma package (v3.58.1). Genes were regarded as differentially expressed when the adjusted criteria met *p* < 0.05 and |log2FC| > 1. PCA plots were produced with ggord (v1.1.6), and heatmaps were generated via pheatmap (v1.0.12). All online databases and web tools were accessed in April 2024; URLs are provided in the text for reference.

### 4.3. Functional Enrichment Analysis

Enrichment analyses were carried out on the Sangerbox platform (http://sangerbox.com/home.html, version 3.0, accessed on 20 April 2024) [41]. For Gene Ontology (GO) and Kyoto Encyclopedia of Genes and Genomes (KEGG) analyses, the permitted gene-set size ranged from 5 to 5000, with significance set at *p* < 0.05 and a false discovery rate (FDR) < 0.25. In addition to DEG-based enrichment, gene set enrichment analysis (GSEA) was conducted to characterize global functional shifts. Enrichment heatmaps were generated using the Bioinformatics platform (https://www.bioinformatics.com.cn, accessed on 20 April 2024) [42]. Significant gene sets were identified based on an FDR < 0.25. For each enrichment analysis (GO, KEGG, and GSEA), multiple testing correction was applied using the Benjamini–Hochberg method, and adjusted *p*-values (FDR) were used to determine statistical significance.

### 4.4. Co-Expression Network Construction and Identification of Pivotal Module

Weighted Gene Co-expression Network Analysis (WGCNA) was performed using the Sangerbox platform (https://www.sangerbox.com/, accessed on 23 April 2024) [41] with the R package WGCNA. The input expression matrix included 30 samples and 8573 genes. After removing genes with zero variance, the top 50% most variable genes ranked by MAD were retained for network construction (*n* = 4287). Hierarchical clustering was used to detect outliers, and no samples were excluded (*n* = 30). An unsigned network was constructed to capture both positive and negative correlations among genes. The soft-thresholding power was selected as β = 3 using the pickSoftThreshold function in the WGCNA package (v1.72-1), and clustering was conducted based on TOM with 1−TOM dissimilarity. The soft-thresholding power was selected as the lowest value that approximates scale-free topology while preserving sufficient network connectivity. Although a higher power may further improve the scale-free topology fit, it may also reduce network connectivity, particularly in datasets with limited sample size. Modules were identified by dynamic tree cut (minModuleSize = 30, deepSplit = 3) and merged by module eigengene similarity (merge cut height = 0.25), yielding 10 modules. Hub genes were screened using MM ≥ 0.8 and GS ≥ 0.1, and edges with weight ≥ 0.1 were retained to extract candidate hub genes [26,43]. These thresholds were selected based on commonly used criteria in WGCNA studies to identify genes with strong module connectivity and biological relevance. However, these thresholds are heuristic and may include genes with varying levels of association, and therefore should be interpreted with caution.

### 4.5. PPI Network Construction and Candidate Target Screening

Key module genes from WGCNA were intersected with DEGs, BTM targets, and RA targets using a Venn diagram. The resulting intersection targets were mapped to the STRING database (http://string-db.org, version 11.5, accessed on 25 April 2024) to analyze protein–protein interactions (PPIs) [44]. A confidence score of 0.4 was selected to retain sufficient network coverage for exploratory analysis. This threshold may include lower-confidence interactions, and therefore the PPI network should be interpreted as a hypothesis-generating framework rather than definitive evidence of molecular interactions. The network was visualized and analyzed using Cytoscape 3.9.1 (https://www.cytoscape.org/, version 3.9.1, accessed on 25 April 2024, Institute for Systems Biology, Seattle, WA, USA). Candidate hub targets were identified based on topological degree centrality.

### 4.6. GeneMANIA Network Analysis

A functional association network for the top 10 candidate targets was constructed using GeneMANIA (accessed on 26 April 2024) [45]. This analysis identifies genes associated with the query targets based on co-expression, genetic interactions, and physical interactions [45]. The resulting network yielded an expanded gene set (GMFA-ED), adding 10 interactors per candidate target [45,46]. Functional enrichment analysis of the GMFA-ED set was performed and visualized using the Bioinformatics platform.

### 4.7. ROC-Based Evaluation of Candidate Targets

We generated ROC curves to assess the discriminatory ability of candidate targets in the analyzed datasets. Genes with relatively higher AUC (AUC > 0.85) values were retained for prioritization. Heatmaps for these targets were generated using the Bioinformatics platform [42].

### 4.8. Molecular Docking

Molecular docking simulations between BTM active ingredients and RA-related targets were conducted using AutoDock Vina (v1.1.2). The three-dimensional structures of target proteins were downloaded from the RCSB PDB repository. Protein receptors and ligands were processed in PyMOL (version 3.1.6.1, Schrödinger, LLC, New York, NY, USA) [47] and AutoDock Tools (version 1.5.7, The Scripps Research Institute, La Jolla, CA, USA) [48], including deletion of crystallographic water molecules, addition of hydrogen atoms, and conversion to the PDBQT format. Docking poses were inspected and rendered in PyMOL, and lower binding energy was used as a relative indicator of binding affinity.

### 4.9. Machine Learning Algorithms

Machine learning analyses were performed using expression levels of 10 candidate genes: *TNF*, *IL6*, *MMP9*, *PTPRC*, *JUN*, *CXCR4*, *EGFR*, *CCL5*, *PPARG*, and *STAT1*. The expression matrix was arranged in a samples-by-genes format, and samples were labeled as Disease or Control based on phenotype annotations. All analyses were conducted in R with a fixed random seed (set.seed = 1234) to ensure reproducibility.

Least absolute shrinkage and selection operator (LASSO) logistic regression was carried out using the glmnet package with a binomial model and alpha set to 1. The optimal penalty parameter λ was determined by 10-fold cross-validation with cv.glmnet, and genes with non-zero coefficients at λmin and λ1se were retained. Support vector machine–recursive feature elimination (SVM-RFE) was performed using the caret package with a radial basis function kernel. Predictors were centered and scaled within the resampling process. Model performance was assessed using repeated cross-validation with 5 folds and 3 repeats, and up-sampling was applied within each training fold to address class imbalance. Feature subset sizes from 2 to 10 were evaluated. Random forest (RF) models were trained in caret with 10-fold cross-validation and 500 trees. To further evaluate the generalizability of the identified gene signature, we validated its classification performance in an independent external dataset (GSE55457), where Accuracy, Sensitivity, Specificity, and F1-score were calculated (Supplementary Table S19). The external dataset (GSE55457) was used exclusively for independent evaluation and was not involved in feature selection, model training, or parameter tuning.

The analysis scripts used in this study are available from the corresponding author upon reasonable request.

### 4.10. Molecular Dynamics Simulation

All molecular dynamics runs were conducted with the GROMACS package (version 2022, University of Groningen, The Netherlands). Ligand topology and parameters were prepared using sobtop_1.0 (dev3.1, Sobereva Lab) with the GAFF2 framework; partial charges were derived via the RESP scheme, whereas the protein was modeled with the AMBER14SB force field. The complex was embedded in a cubic TIP3P water box with a 1.0 nm buffer, followed by addition of NaCl to 0.15 M using gmx genion to neutralize the net charge and approximate physiological salinity. Electrostatics at long range were handled by the particle-mesh Ewald approach with a 1.0 nm real-space cutoff, and all covalent bonds involving hydrogens were constrained through the LINCS method. After energy minimization, the system was sequentially equilibrated under the NVT and NPT ensembles to stabilize temperature and pressure, respectively, followed by a 100 ns production run under the NPT ensemble. These equilibration steps were performed prior to production simulations to ensure system stability. To assess system stability and characterize conformational behavior, the trajectories were examined by calculating RMSD and RMSF, together with hydrogen-bonding patterns, the radius of gyration (Rg), and the solvent-accessible surface area (SASA).

### 4.11. Immune Infiltration Analysis

The CIBERSORT method was applied using the LM22 signature matrix to infer the proportions of infiltrating immune-cell subsets in RA and control samples, and samples with CIBERSORT *p* < 0.05 were retained for further analysis. The estimated immune-cell distributions were summarized using box-and-whisker plots, and between-group differences were evaluated with the Wilcoxon rank-sum test (*p* < 0.05). In addition, relationships between candidate targets and immune infiltration were examined by Spearman correlation analysis in R (v4.3.3).

### 4.12. Experimental Validation In Vitro

#### 4.12.1. Cell Culture and Reagents

MH7A human rheumatoid arthritis synovial fibroblast cells were purchased from Guangzhou Jennio Biotech Co., Ltd. (Guangzhou, China). Cells were grown in DMEM (Gibco; Thermo Fisher Scientific, USA) containing 10% FBS (Gibco; Thermo Fisher Scientific, Waltham, MA, USA) and 1% penicillin/streptomycin (Gibco; Thermo Fisher Scientific, Waltham, MA, USA). The cultures were kept at 37 °C under humidified conditions with 5% CO_2_ in an incubator (Thermo Fisher Scientific, Waltham, MA, USA). Recombinant human TNF-α was obtained from PeproTech (Rocky Hill, NJ, USA). Formononetin (CAS No. 485-72-3, purity ≥ 98%) and Rhein (CAS No. 478-43-3, purity ≥ 98%) were purchased from MedChemExpress (MCE, Monmouth Junction, NJ, USA). The MTT reagent was obtained from Sigma-Aldrich (Merck, Darmstadt, Germany). Primers for key genes were synthesized by Sangon Biotech Co., Ltd. (Shanghai, China).

#### 4.12.2. MTT Analysis

Cell viability was determined by an MTT-based colorimetric assay. MH7A cells were plated into 96-well plates at 5 × 10^4^ cells/mL and allowed to adhere for 12 h. The cells were exposed to Formononetin or Rhein at graded doses (0.78–50 μM) for 24 h. After treatment, 20 μL MTT reagent (5 mg/mL) was added to each well and incubation was continued for 4 h. The supernatant was then carefully discarded, and 150 μL DMSO was introduced to solubilize the resulting formazan. Optical density was measured at 490 nm. Each experiment was independently repeated three times (biological replicates, *n* = 3), with each condition assayed in technical replicates.

#### 4.12.3. RT-qPCR Analysis

Cells were plated in six-well dishes at a density of 2 × 10^5^ cells/mL and cultured for 24 h. To establish the inflammatory model, the model and treatment groups were exposed to TNF-α (1 ng/mL), whereas the control wells were replaced with fresh medium only. This concentration was selected to induce a measurable inflammatory response while minimizing excessive cytotoxic effects. In parallel, the treatment groups received Formononetin or Rhein at 1.56, 3.125, and 6.25 μM. After a further 24 h, total RNA was isolated with the SteadyPure Rapid RNA Extraction Kit. RNA concentration and purity were assessed by a NanoDrop spectrophotometer (Thermo Fisher Scientific, Waltham, MA, USA). Equal amounts of RNA were reverse-transcribed into cDNA using a commercial reverse transcription kit. RT-qPCR was performed using a SYBR Green qPCR Master Mix on a real-time PCR system, with primer sequences listed in Supplementary Table S21. Melt-curve analysis was conducted to support amplification specificity. β-actin was used as the internal reference gene. Each sample was analyzed in technical triplicates, and experiments were independently repeated three times (biological replicates, *n* = 3). Relative mRNA expression levels were calculated using the 2^−ΔΔCt^ method.

## 5. Conclusions

This study identified Formononetin and Rhein as candidate active components of BTM and highlighted *JUN* and *PPARG* as potential targets in rheumatoid arthritis. The results suggest that BTM may regulate inflammatory responses through these targets. Further in vivo studies are needed to confirm these findings and clarify the underlying mechanisms.

## Data Availability

Publicly available datasets were analyzed in this study. Transcriptome data were obtained from the NCBI Gene Expression Omnibus (GEO) under accession numbers GSE1919, GSE55235, and GSE55457 (https://www.ncbi.nlm.nih.gov/geo/, accessed on 10 April 2024). Other data used in this study were retrieved from publicly accessible databases and web tools, including TCMSP (https://tcmsp-e.com/index.php, accessed on 10 April 2024), HERB (http://herb.ac.cn/, accessed on 10 April 2024), SwissTargetPrediction (http://www.swisstargetprediction.ch/, accessed on 10 April 2024), SEA (https://sea.bkslab.org/, accessed on 10 April 2024), PharmMapper (http://www.lilab-ecust.cn/pharmmapper/, accessed on 10 April 2024), UniProt (https://www.uniprot.org/, accessed on 10 April 2024), DisGeNET (https://www.disgenet.org/, accessed on 10 April 2024), GeneCards (https://www.genecards.org/, accessed on 10 April 2024), DrugBank (https://go.drugbank.com/, accessed on 10 April 2024), TTD (https://db.idrblab.net/ttd/, accessed on 10 April 2024), STRING (http://string-db.org, accessed on 10 April 2024), and RCSB PDB (https://www.rcsb.org/, accessed on 10 April 2024). The analysis scripts are available from the corresponding author upon reasonable request.

## Supplementary Materials

The following supporting information can be downloaded at: https://www.mdpi.com/article/10.3390/ph19050735/s1. Table S1. 19-Herb TCM Formula. Table S2-1. Active ingredients (TCMSP). Table S2-2. Active ingredients (HERB). Table S3. The target genes of BTHJ active ingredients. Table S4. The target genes of RA. Table S5. The 798 differentially expressed genes (DEGs). Table S6. GO enrichment analysis of DEGs. Table S7. KEGG enrichment analysis of DEGs. Table S8. Gene set enrichment analysis. Table S9. 170 core genes of the turquoise module. Table S10. 10 candidate targets in the PPI network. Table S11-1. Abbreviated identifiers (ID) of Chinese medicinal herbs in the network diagram. Table S11-2. Abbreviated identifiers (ID) of active ingredients in the network diagram. Table S12. GMFA extended gene list (GMFA-ED). Table S13. BP enrichment analysis of GMFA-ED. Table S14. CC enrichment analysis of GMFA-ED. Table S15. MF enrichment analysis of GMFA-ED. Table S16. KEGG enrichment analysis of GMFA-ED. Table S17. 27 active ingredients of the drug-active ingredient-target network. Table S18. Molecular docking binding energy results for 27 active ingredients and 10 candidate targets. Table S19. Performance comparison of machine learning models. Table S20. Data set information. Table S21. Primers for RT-qPCR. Figure S1. GeneMANIA functional association (GMFA) network analyses illustrated functionally related genes associated with 10 candidate targets and created an expanded database of potential targets for RA (GMFA-ED). Figure S2. GO and KEGG enrichment analysis of intersecting targets and GMFA-ED targets. (A) BP terms of GO enrichment, (B) CC terms of GO enrichment, (C) MF terms of GO enrichment, (D) KEGG enrichment.

## Abbreviations

BTM, Bitong Mixture; RA, rheumatoid arthritis; TCM, traditional Chinese medicine; DEGs, differentially expressed genes; WGCNA, weighted gene co-expression network analysis; ROC, receiver operating characteristic; MTT, 3-(4,5)-dimethylthiazol-2-yl-2,5-diphenyltetrazolium bromide; RT-qPCR, quantitative reverse transcription polymerase chain reaction; TNF-α, tumor necrosis factor-alpha; GEO, gene expression omnibus; IL6, interleukin-6; MMP9, matrix metalloproteinase 9; CCL5, Chemokine (C-C motif) ligand 5; PPARG, peroxisome proliferator-activated receptor gamma; TNF, tumor necrosis factor; JUN, Jun proto-oncogene; PTPRC, protein tyrosine phosphatase receptor type, C; AP-1, activator protein-1; CXCR4, chemokine (C-X-C motif) receptor 4; EGFR, epidermal growth factor receptor; IL-1β, interleukin-1 beta; OMIM, online mendelian inheritance in man; STAT1, signal transducer and activator of transcription 1; TTD, Therapeutic Target Database; PCA, principal component analysis; GO, gene ontology; KEGG, Kyoto encyclopedia of genes and genomes; GSEA, gene set enrichment analysis; FDR, false discovery rate; MAD, median absolute deviation; TOM, topological overlap matrix; AUC, area under the curve; PPI, protein–protein interaction; BP, biological process; CC, Cellular Component; MF, molecular functions.
