# Supplementary material for: Formononetin and Rhein from Bitong Mixture Alleviate Rheumatoid Arthritis-Related Inflammation: An Integrated WGCNA, Machine Learning and In Vitro Study
Source: Pharmaceuticals (Basel). 2026 May 7;19(5):735. doi: 10.3390/ph19050735 (PMC13209788; doi:10.3390/ph19050735)
Supplement: Supplementary file 1 [file pharmaceuticals-19-00735-s001.zip › Supplementary Material-0406.pdf]

Supplementary Material

Formononetin and Rhein from Bitong Mixture Alleviate Rheumatoid Arthritis-Related Inflammation: An Integrated WGCNA, Machine Learning and In Vitro Study

Futing Tan [1,†], Jiangtao Wang [1,†], Keqing Fan [1], Runzi Gao [1,2,*] and Zhibin Yang [1,3,*]

[1] Yunnan Provincial Key Laboratory of Entomological Biopharmaceutical R&D, College of Pharmacy, Dali University, Dali 671003, China; 17869025994@163.com (F.T.); andrevvw@163.com (J.W.); fan18869805251@163.com (K.F.)
[2] Nanjing Hospital of Chinese Medicine Affiliated to Nanjing University of Chinese Medicine, Nanjing 210001, China
[3] National-Local Joint Engineering Research Center of Entomoceutics, Dali 671003, China
* Correspondence: gaoorthopedic@163.com (R.G.); yangzhibin@dali.edu.cn (Z.Y.)
† These authors contributed equally to this work.

Supplementary Table S1. 19-Herb TCM Formula

No.	Latin binomial name
1	*Spatholobus suberectus*
2	*Saposhnikovia divaricata*
3	*Aconitum carmichaelii*
4	*Aconitum kusnezoffii*
5	*Angelica pubescens*
6	*Dipsacus asper*
7	*Achyranthes bidentata*
8	*Cibotium barometz*
9	*Reynoutria japonica*
10	*Glycyrrhiza uralensis*
11	*Atractylodes macrocephala*
12	*Coix lacryma-jobi*
13	*Clematis chinensis*
14	*Citrus reticulata*
15	*Periploca sepium*
16	*Eupolyphaga sinensis*
17	*Cynanchum paniculatum*
18	*Poria cocos*
19	*Cinnamomum cassia*

Supplementary Table S2-1. Active ingredients (TCMSP)

No.	MolID	Molecule Name	ID	OB	DL
1	MOL000296	hederagenin	A1	36.91	0.75
2	MOL000033	(3S,8S,9S,10R,13R,14S,17R)-10,13-dimethyl-17-[(2R, 5S)-5-propan-2-yloctan-2-yl]-2,3,4,7,8,9,11,12,14,15,16 ,17-dodecahydro-1H-cyclopenta[a]phenanthren-3-ol	B1	36.23	0.78
3	MOL000358	beta-sitosterol	C1	36.91	0.75
4	MOL000392	formononetin	D1	69.67	0.21
5	MOL000417	Calycosin	D2	47.75	0.24
6	MOL000449	Stigmasterol	E1	43.83	0.76
7	MOL000461	3,7-dihydroxy-6-methoxy-dihydroflavonol	JXT1	43.8	0.26
8	MOL000468	8-o-Methylreyusi	JXT2	70.32	0.27
9	MOL000469	3-Hydroxystigmast-5-en-7-one	JXT3	40.93	0.78
10	MOL000470	8-C-α-L-arabinosylluteolin	JXT4	35.54	0.66
11	MOL000471	aloe-emodin	JXT5	83.38	0.24
12	MOL000483	(Z)-3-(4-hydroxy-3-methoxy-phenyl)-N-[2-(4-hydroxyp henyl)ethyl]acrylamide	JXT6	118.35	0.26
13	MOL000490	petunidin	JXT7	30.05	0.31
14	MOL000491	Augelicin	JXT8	37.5	0.66
15	MOL000492	(+)-catechin	F1	54.83	0.24
16	MOL000493	campesterol	JXT9	37.58	0.71
17	MOL000497	licochalcone a	D3	40.79	0.29
18	MOL000500	Vestitol	D4	74.66	0.21
19	MOL000501	Consume close grain	JXT10	68.12	0.27
20	MOL000502	Cajinin	JXT11	68.8	0.27
21	MOL000503	Medicagol	JXT12	57.49	0.6
22	MOL000506	Lupinidine	JXT13	61.89	0.21
23	MOL000507	Psi-Baptigenin	JXT14	70.12	0.31
24	MOL000006	luteolin	F2	36.16	0.25
25	MOL000011	(2R,3R)-3-(4-hydroxy-3-methoxy-phenyl)-5-methoxy-2 -methylol-2,3-dihydropyrano[5,6-h][1,4]benzodioxin-9- one	FF1	68.83	0.66
26	MOL011730	11-hydroxy-sec-o-beta-d-glucosylhamaudol_qt	FF2	50.24	0.27
27	MOL011732	anomalin	FF3	59.65	0.66
28	MOL011737	divaricatacid	FF4	87	0.32
29	MOL011740	divaricatol	FF5	31.65	0.38
30	MOL001941	Ammidin	G1	34.55	0.22
31	MOL011747	ledebouriellol	FF6	32.05	0.51
32	MOL011749	phelloptorin	FF7	43.39	0.28
33	MOL011753	5-O-Methylvisamminol	FF8	37.99	0.25
34	MOL002644	Phellopterin	FF9	40.19	0.28
35	MOL000359	sitosterol	H1	36.91	0.75
36	MOL000173	wogonin	I1	30.68	0.23
37	MOL000358	beta-sitosterol	C1	36.91	0.75

38	MOL001494	Mandenol	J1	42	0.19
39	MOL001942	isoimperatorin	G2	45.46	0.23
40	MOL003588	Prangenidin	FF10	36.31	0.22
41	MOL007514	methyl icosa-11,14-dienoate	FF11	39.67	0.23
42	MOL013077	Decursin	FF12	39.27	0.38
43	MOL002086	1-[(5R,8R,9S,10S,12R,13S,14S,17S)-12-hydroxy-10,13-dimethyl-2,3,4,5,6,7,8,9,11,12,14,15,16,17-tetradecahydro-1H-cyclopenta[a]phenanthren-17-yl]ethanone	CHW1	33.47	0.42
44	MOL002087	delta4,16-Androstadien-3-one	CHW2	37.63	0.31
45	MOL000538	hypaconitine	K1	31.39	0.26
46	MOL001941	Ammidin	G1	34.55	0.22
47	MOL001942	isoimperatorin	G2	45.46	0.23
48	MOL000358	beta-sitosterol	C1	36.91	0.75
49	MOL003608	O-Acetylcolumbianetin	DH1	60.04	0.26
50	MOL004777	Angelol D	DH2	34.85	0.34
51	MOL004778	[(1R,2R)-2,3-dihydroxy-1-(7-methoxy-2-oxochromen-6-yl)-3-methylbutyl] (Z)-2-methylbut-2-enoate	DH3	46.03	0.34
52	MOL004780	Angelicone	DH4	30.99	0.19
53	MOL004782	[(1R,2R)-2,3-dihydroxy-1-(7-methoxy-2-oxochromen-6-yl)-3-methylbutyl] 3-methylbutanoate	DH5	45.19	0.34
54	MOL004792	nodakenin	DH6	57.12	0.69
55	MOL002397	karakoline	CW1	51.73	0.73
56	MOL004748	3-deoxyaconitine	CW2	30.96	0.24
57	MOL004749	3-acetylaconitine	CW3	37.05	0.2
58	MOL004756	crassicauline A	CW4	34.13	0.21
59	MOL004757	yunaconitine	CW5	33.56	0.2
60	MOL004759	napelline	CW6	34.48	0.72
61	MOL004763	Izoteolin	CW7	39.53	0.51
62	MOL000538	hypaconitine	K1	31.39	0.26
63	MOL003152	Gentisin	XD1	64.06	0.21
64	MOL000358	beta-sitosterol	C1	36.91	0.75
65	MOL000359	sitosterol	H1	36.91	0.75
66	MOL009312	(E,E)-3,5-Di-O-caffeoylquinic acid	XD2	48.14	0.68
67	MOL009317	Cauloside A_qt	XD3	43.32	0.81
68	MOL008188	Japonine	XD4	44.11	0.25
69	MOL009322	Sylvestroside III	XD5	48.02	0.53
70	MOL009323	Sylvestroside III_qt	XD6	56.47	0.43
71	MOL001006	poriferasta-7,22E-dien-3beta-ol	NX1	42.98	0.76
72	MOL012461	28-norolean-17-en-3-ol	NX2	35.93	0.78
73	MOL012505	bidentatoside,ii_qt	NX3	31.76	0.59
74	MOL012537	Spinoside A	NX4	41.75	0.4
75	MOL012542	β-ecdysterone	NX5	44.23	0.82
76	MOL001454	berberine	NX6	36.86	0.78
77	MOL001458	coptisine	NX7	30.67	0.86

78	MOL000173	wogonin	I1	30.68	0.23
79	MOL002643	delta 7-stigmastenol	NX8	37.42	0.75
80	MOL002714	baicalein	NX9	33.52	0.21
81	MOL002776	Baicalin	NX10	40.12	0.75
82	MOL002897	epiberberine	NX11	43.09	0.78
83	MOL000358	beta-sitosterol	C1	36.91	0.75
84	MOL003847	Inophyllum E	NX12	38.81	0.85
85	MOL000422	kaempferol	L1	41.88	0.24
86	MOL004355	Spinasterol	NX13	42.98	0.76
87	MOL000449	Stigmasterol	E1	43.83	0.76
88	MOL000785	palmatine	NX14	64.6	0.65
89	MOL000085	beta-daucosterol_qt	NX15	36.91	0.75
90	MOL000098	quercetin	P1	46.43	0.28
91	MOL013281	6,8-Dihydroxy-7-methoxyxanthone	HZ1	35.83	0.21
92	MOL013287	Physovenine	HZ2	106.21	0.19
93	MOL013288	Picralinal	HZ3	58.01	0.75
94	MOL002259	Physciondiglucoside	HZ4	41.65	0.63
95	MOL002268	rhein	HZ5	47.07	0.28
96	MOL002280	Torachrysone-8-O-beta-D-(6'-oxayl)-glucoside	HZ6	43.02	0.74
97	MOL000358	beta-sitosterol	C1	36.91	0.75
98	MOL000492	(+)-catechin	G2	54.83	0.24
99	MOL000006	luteolin	F2	36.16	0.25
100	MOL000098	quercetin	P1	46.43	0.28
101	MOL001484	Inermine	GC1	75.18	0.54
102	MOL001792	DFV	GC2	32.76	0.18
103	MOL000211	Mairin	GC3	55.38	0.78
104	MOL002311	Glycyrol	GC4	90.78	0.67
105	MOL000239	Jaranol	GC5	50.83	0.29
106	MOL002565	Medicarpin	GC6	49.22	0.34
107	MOL000354	isorhamnetin	GC7	49.6	0.31
108	MOL000359	sitosterol	H1	36.91	0.75
109	MOL003656	Lupiwighteone	GC8	51.64	0.37
110	MOL003896	7-Methoxy-2-methyl isoflavone	GC9	42.56	0.2
111	MOL000392	formononetin	D1	69.67	0.21
112	MOL000417	Calycosin	D2	47.75	0.24
113	MOL000422	kaempferol	L1	41.88	0.24
114	MOL004328	naringenin	M1	59.29	0.21
115	MOL004805	(2S)-2-[4-hydroxy-3-(3-methylbut-2-enyl)phenyl]-8,8-dimethyl-2,3-dihydropyrano[2,3-f]chromen-4-one	GC10	31.79	0.72
116	MOL004806	euchrenone	GC11	30.29	0.57
117	MOL004808	glyasperin B	GC12	65.22	0.44
118	MOL004810	glyasperin F	GC13	75.84	0.54
119	MOL004811	Glyasperin C	GC14	45.56	0.4
120	MOL004814	Isotrifoliol	GC15	31.94	0.42

121	MOL004815	(E)-1-(2,4-dihydroxyphenyl)-3-(2,2-dimethylchromen-6-yl)prop-2-en-1-one	GC16	39.62	0.35
122	MOL004820	kanzonols W	GC17	50.48	0.52
123	MOL004824	(2S)-6-(2,4-dihydroxyphenyl)-2-(2-hydroxypropan-2-yl)-4-methoxy-2,3-dihydrofuro[3,2-g]chromen-7-one	GC18	60.25	0.63
124	MOL004827	Semilicoisoflavone B	GC19	48.78	0.55
125	MOL004828	Glepidotin A	GC20	44.72	0.35
126	MOL004829	Glepidotin B	GC21	64.46	0.34
127	MOL004833	Phaseolinisoflavan	GC22	32.01	0.45
128	MOL004835	Glypallichalcone	GC23	61.6	0.19
129	MOL004838	8-(6-hydroxy-2-benzofuranyl)-2,2-dimethyl-5-chromenol	GC24	58.44	0.38
130	MOL004841	Licochalcone B	GC25	76.76	0.19
131	MOL004848	licochalcone G	GC26	49.25	0.32
132	MOL004849	3-(2,4-dihydroxyphenyl)-8-(1,1-dimethylprop-2-enyl)-7-hydroxy-5-methoxy-coumarin	GC27	59.62	0.43
133	MOL004855	Licoricone	GC28	63.58	0.47
134	MOL004856	Gancaonin A	GC29	51.08	0.4
135	MOL004857	Gancaonin B	GC30	48.79	0.45
136	MOL004860	licorice glycoside E	GC31	32.89	0.27
137	MOL004863	3-(3,4-dihydroxyphenyl)-5,7-dihydroxy-8-(3-methylbut-2-enyl)chromone	GC32	66.37	0.41
138	MOL004864	5,7-dihydroxy-3-(4-methoxyphenyl)-8-(3-methylbut-2-enyl)chromone	GC33	30.49	0.41
139	MOL004866	2-(3,4-dihydroxyphenyl)-5,7-dihydroxy-6-(3-methylbut-2-enyl)chromone	GC34	44.15	0.41
140	MOL004879	Glycyrin	GC35	52.61	0.47
141	MOL004882	Licocoumarone	GC36	33.21	0.36
142	MOL004883	Licoisoflavone	GC37	41.61	0.42
143	MOL004884	Licoisoflavone B	GC38	38.93	0.55
144	MOL004885	licoisoflavanone	GC39	52.47	0.54
145	MOL004891	shinpterocarpin	GC40	80.3	0.73
146	MOL004898	(E)-3-[3,4-dihydroxy-5-(3-methylbut-2-enyl)phenyl]-1-(2,4-dihydroxyphenyl)prop-2-en-1-one	GC41	46.27	0.31
147	MOL004903	liquiritin	GC42	65.69	0.74
148	MOL004904	licopyranocoumarin	GC43	80.36	0.65
149	MOL004905	3,22-Dihydroxy-11-oxo-delta(12)-oleanene-27-alpha-methoxycarbonyl-29-oic acid	GC44	34.32	0.55
150	MOL004907	Glyzaglabrin	GC45	61.07	0.35
151	MOL004908	Glabridin	GC46	53.25	0.47
152	MOL004910	Glabranin	GC47	52.9	0.31
153	MOL004911	Glabrene	GC48	46.27	0.44
154	MOL004912	Glabrone	GC49	52.51	0.5
155	MOL004913	1,3-dihydroxy-9-methoxy-6-benzofurano[3,2-c]chrome	GC50	48.14	0.43

		none			
156	MOL004914	1,3-dihydroxy-8,9-dimethoxy-6-benzofurano[3,2-c]chromenone	GC51	62.9	0.53
157	MOL004915	Eurycarpin A	GC52	43.28	0.37
158	MOL004917	glycyroside	GC53	37.25	0.79
159	MOL004924	(-)-Medicocarpin	GC54	40.99	0.95
160	MOL004935	Sigmoidin-B	GC55	34.88	0.41
161	MOL004941	(2R)-7-hydroxy-2-(4-hydroxyphenyl)chroman-4-one	GC56	71.12	0.18
162	MOL004945	(2S)-7-hydroxy-2-(4-hydroxyphenyl)-8-(3-methylbut-2-enyl)chroman-4-one	GC57	36.57	0.32
163	MOL004948	Isoglycyrol	GC58	44.7	0.84
164	MOL004949	Isolicoflavonol	GC59	45.17	0.42
165	MOL004957	HMO	GC60	38.37	0.21
166	MOL004959	1-Methoxyphaseollidin	GC61	69.98	0.64
167	MOL004961	Quercetin der.	GC62	46.45	0.33
168	MOL004966	3'-Hydroxy-4'-O-Methylglabridin	GC63	43.71	0.57
169	MOL000497	licochalcone a	D3	40.79	0.29
170	MOL004974	3'-Methoxyglabridin	GC64	46.16	0.57
171	MOL004978	2-[(3R)-8,8-dimethyl-3,4-dihydro-2H-pyrano[6,5-f]chromen-3-yl]-5-methoxyphenol	GC65	36.21	0.52
172	MOL004980	Inflacoumarin A	GC66	39.71	0.33
173	MOL004985	icos-5-enoic acid	GC67	30.7	0.2
174	MOL004988	Kanzonol F	GC68	32.47	0.89
175	MOL004989	6-prenylated eriodictyol	GC69	39.22	0.41
176	MOL004990	7,2',4'-trihydroxy－5-methoxy-3－arylcoumarin	GC70	83.71	0.27
177	MOL004991	7-Acetoxy-2-methylisoflavone	GC71	38.92	0.26
178	MOL004993	8-prenylated eriodictyol	GC72	53.79	0.4
179	MOL004996	gadelaidic acid	GC73	30.7	0.2
180	MOL000500	Vestitol	D4	74.66	0.21
181	MOL005000	Gancaonin G	GC74	60.44	0.39
182	MOL005001	Gancaonin H	GC75	50.1	0.78
183	MOL005003	Licoagrocarpin	GC76	58.81	0.58
184	MOL005007	Glyasperins M	GC77	72.67	0.59
185	MOL005008	Glycyrrhiza flavonol A	GC78	41.28	0.6
186	MOL005012	Licoagroisoflavone	GC79	57.28	0.49
187	MOL005013	18α-hydroxyglycyrrhetic acid	GC84	41.16	0.71
188	MOL005016	Odoratin	GC80	49.95	0.3
189	MOL005017	Phaseol	GC81	78.77	0.58
190	MOL005018	Xambioona	GC82	54.85	0.87
191	MOL005020	dehydroglyasperins C	GC83	53.82	0.37
192	MOL000098	quercetin	P1	46.43	0.28
193	MOL000072	8β-ethoxy atractylenolide III	BZ1	35.95	0.21
194	MOL000033	(3S,8S,9S,10R,13R,14S,17R)-10,13-dimethyl-17-[(2R,5S)-5-propan-2-yloctan-2-yl]-2,3,4,7,8,9,11,12,14,15,16	B1	36.23	0.78

		,17-dodecahydro-1H-cyclopenta[a]phenanthren-3-ol			
195	MOL000028	α-Amyrin	BZ2	39.51	0.76
196	MOL000049	3β-acetoxyatractylone	BZ3	54.07	0.22
197	MOL000021	14-acetyl-12-senecioyl-2E,8E,10E-atractylentriol	BZ4	60.31	0.31
198	MOL000020	12-senecioyl-2E,8E,10E-atractylentriol	BZ5	62.4	0.22
199	MOL000022	14-acetyl-12-senecioyl-2E,8Z,10E-atractylentriol	BZ6	63.37	0.3
200	MOL001323	Sitosterol alpha1	YYR1	43.28	0.78
201	MOL001494	Mandenol	J1	42	0.19
202	MOL002372	(6Z,10E,14E,18E)-2,6,10,15,19,23-hexamethyltetracosa-2,6,10,14,18,22-hexaene	N1	33.55	0.42
203	MOL002882	[(2R)-2,3-dihydroxypropyl] (Z)-octadec-9-enoate	YYR2	34.13	0.3
204	MOL000359	sitosterol	H1	36.91	0.75
205	MOL000449	Stigmasterol	E1	43.83	0.76
206	MOL008118	Coixenolide	YYR3	32.4	0.43
207	MOL008121	2-Monoolein	YYR4	34.23	0.29
208	MOL000953	CLR	YYR5	37.87	0.68
209	MOL001663	(4aS,6aR,6aS,6bR,8aR,10R,12aR,14bS)-10-hydroxy-2,2,6a,6b,9,9,12a-heptamethyl-1,3,4,5,6,6a,7,8,8a,10,11,12,13,14b-tetradecahydropicene-4a-carboxylic acid	WLX1	32.03	0.76
210	MOL002372	(6Z,10E,14E,18E)-2,6,10,15,19,23-hexamethyltetracosa-2,6,10,14,18,22-hexaene	N1	33.55	0.42
211	MOL000358	beta-sitosterol	C1	36.91	0.75
212	MOL000449	Stigmasterol	E1	43.83	0.76
213	MOL005594	ClematosideA'_qt	WLX4	37.51	0.76
214	MOL005598	Embinin	WLX2	33.91	0.73
215	MOL005603	Heptyl phthalate	WLX3	42.26	0.31
216	MOL000359	sitosterol	H1	36.91	0.75
217	MOL004328	naringenin	M1	59.29	0.21
218	MOL005100	5,7-dihydroxy-2-(3-hydroxy-4-methoxyphenyl)chroman-4-one	CP1	47.74	0.27
219	MOL005815	Citromitin	CP2	86.9	0.51
220	MOL005828	nobiletin	CP3	61.67	0.52
221	MOL001771	poriferast-5-en-3beta-ol	XJP1	36.91	0.75
222	MOL000358	beta-sitosterol	C1	36.91	0.75
223	MOL000359	sitosterol	H1	36.91	0.75
224	MOL005645	21-O-Methyl-5,14-pregndiene-3β,14β,17β,21-tetrol-20-one	XJP2	38.52	0.57
225	MOL005646	21-O-Methyl-5-pregnene-3beta,14beta,17beta,20,21-pentol	XJP3	45.12	0.6
226	MOL005652	Glycoside K_qt	XJP4	31.91	0.43
227	MOL005654	Glycoside H2_qt	XJP15	49.95	0.48
228	MOL005656	Glycozolidal	XJP5	78.07	0.2
229	MOL005658	Periplogenin	XJP6	36.61	0.74
230	MOL005664	glycoside E_qt	XJP7	40.57	0.47

231	MOL005666	Periplocoside M_qt	XJP8	32	0.88
232	MOL005675	periplocoside E_qt	XJP14	63.94	0.24
233	MOL005683	delta 5-Pregnenetriol	XJP9	35.94	0.47
234	MOL005686	periplocoside O_qt	XJP10	32	0.88
235	MOL005690	periplocymarin_qt	XJP11	104.15	0.74
236	MOL005692	NERIDIENONE A	XJP12	30.96	0.48
237	MOL005693	xysmalogenin	XJP13	54.41	0.72
238	MOL000359	sitosterol	H1	36.91	0.75
239	MOL005619	Tomentolide A	XCQ1	33.83	0.85
240	MOL005622	tomentogenin	XCQ5	37.84	0.53
241	MOL005623	sarcostin	XCQ2	33.03	0.55
242	MOL005625	cynapanoside C_qt	XCQ3	42.17	0.83
243	MOL005631	cynatratoside B_qt	XCQ4	55.37	0.8
244	MOL000273	(2R)-2-[(3S,5R,10S,13R,14R,16R,17R)-3,16-dihydroxy-4,4,10,13,14-pentamethyl-2,3,5,6,12,15,16,17-octahydro-1H-cyclopenta[a]phenanthren-17-yl]-6-methylhept-5-enoic acid	FL1	30.93	0.81
245	MOL000275	trametenolic acid	FL2	38.71	0.8
246	MOL000276	7,9(11)-dehydropachymic acid	FL3	35.11	0.81
247	MOL000279	Cerevisterol	FL4	37.96	0.77
248	MOL000280	(2R)-2-[(3S,5R,10S,13R,14R,16R,17R)-3,16-dihydroxy-4,4,10,13,14-pentamethyl-2,3,5,6,12,15,16,17-octahydro-1H-cyclopenta[a]phenanthren-17-yl]-5-isopropyl-hex-5-enoic acid	FL5	31.07	0.82
249	MOL000282	ergosta-7,22E-dien-3beta-ol	FL6	43.51	0.72
250	MOL000283	Ergosterol peroxide	FL7	40.36	0.81
251	MOL000285	(2R)-2-[(5R,10S,13R,14R,16R,17R)-16-hydroxy-3-keto-4,4,10,13,14-pentamethyl-1,2,5,6,12,15,16,17-octahydrocyclopenta[a]phenanthren-17-yl]-5-isopropyl-hex-5-enoic acid	FL8	38.26	0.82
252	MOL000287	3beta-Hydroxy-24-methylene-8-lanostene-21-oic acid	FL9	38.7	0.81
253	MOL000289	pachymic acid	FL14	33.63	0.81
254	MOL000290	Poricoic acid A	FL10	30.61	0.76
255	MOL000291	Poricoic acid B	FL11	30.52	0.75
256	MOL000292	poricoic acid C	FL12	38.15	0.75
257	MOL000296	hederagenin	A1	36.91	0.75
258	MOL000300	dehydroeburicoic acid	FL13	44.17	0.83
259	MOL001736	(-)-taxifolin	GZ1	60.51	0.27
260	MOL000358	beta-sitosterol	C1	36.91	0.75
261	MOL000359	sitosterol	H1	36.91	0.75
262	MOL000492	(+)-catechin	F1	54.83	0.24
263	MOL000073	ent-Epicatechin	GZ2	48.96	0.24
264	MOL004576	taxifolin	GZ3	57.84	0.27
265	MOL011169	Peroxyergosterol	GZ4	44.39	0.82

Supplementary Table S2-2. Active ingredients (HERB)

No	Cid	Cmpd Name	ID	MW	Xlogp	Hbond donor	Hbon dacc
1	135398638	Hypoxanthine	TBC1	136.11	-0.5	2	3
2	2355	Bergapten	GJ1	216.19	2.3	0	4
3	5280863	Kaempferol	L1	286.24	1.9	4	6
4	667495	(2R)-5,7-dihydroxy-2-(4-hydroxyphenyl)-2,3-dihydro-4H-chromen-4-one	GJ2	272.25	2.4	3	5
5	5281814	Wighteone	GJ3	338.4	4.6	3	5
6	5490139	Alpinumisoflavone	GJ4	336.3	3.9	2	5
7	5281629	6-Deoxyjacareubin	GJ5	310.3	3.7	2	5
8	5319744	3'-O-Methylorobol	GJ6	300.26	2.6	3	6
9	122841	Aspidinol	GJ7	224.25	2.6	2	4
10	5495920	1,3,7-Trihydroxy-2-prenylxanthone	GJ8	312.3	4.4	3	5
11	5495919	Toxyloxanthone C	GJ9	328.3	3.8	3	6
12	11609510	7-Hydroxy-2-(4-hydroxyphenyl)-8-(3-methylbut-2-enyl)-2,3-dihydrochromen-4-one	GJ10	324.4	4.1	2	4
13	191694	Cudranone	GJ11	328.4	4.6	3	5
14	509244	Cudraflavanone B	GJ12	356.4	4	4	6
15	11948658	gerontoxanthone A	GJ13	394.4	5	2	6
16	5495918	Cudraxanthone S	GJ14	328.3	4	4	6
17	11709692	4',7-Dihydroxy-2',5-dimethoxy-flavonol	GJ15	330.29	1.6	3	7
18	10716607	Cudraxanthone R	GJ16	412.4	4.4	4	7

(Note: ID columns are labelled for each component of the herb to construct a network diagram where A1, B1, C1, D1, D2, E1, F1, D3, D4, F2, G1, H1, I1, J1, G2, K1, L1, M1, N1, P1 belong to the shared constituents present in more than one or two herb）

Supplementary Table S3. The target genes of BTHJ active ingredients

No	MOL ID/Cid	Molecule Name	Target	Symbol
1	MOL000006	luteolin	NF-kappa-B inhibitor alpha	NFKBIA
2	MOL000006	luteolin	Interleukin-10	IL10
3	MOL000006	luteolin	Baculoviral IAP repeat-containing protein 5	BIRC5
4	MOL000006	luteolin	Interferon gamma	IFNG
5	MOL000006	luteolin	Solute carrier family 2, facilitated glucose transporter member 4	SLC2A4
6	MOL000006	luteolin	CD40 ligand	CD40LG
7	MOL000006	luteolin	Kinetochore protein Nuf2	NUF2
8	MOL000006	luteolin	Adenylate cyclase type 2	ADCY2
9	MOL000011	(2R,3R)-3-(4-hydroxy-3-methoxy-phenyl)-5-methoxy-2-methylol-2,3-dihydropyrano[5,6-h][1,4]benzodioxin-9-one	Phosphatidylinositol 4,5-bisphosphate 3-kinase catalytic subunit alpha isoform	PIK3CA
10	MOL000011	(2R,3R)-3-(4-hydroxy-3-methoxy-phenyl)-5-methoxy-2-methylol-2,3-dihydropyrano[5,6-h][1,4]benzodioxin-9-one	Cyclin-dependent kinase 2	CDK2
11	MOL000011	(2R,3R)-3-(4-hydroxy-3-methoxy-phenyl)-5-methoxy-2-methylol-2,3-dihydropyrano[5,6-h][1,4]benzodioxin-9-one	Cyclin-dependent kinase 1	CDK1
12	MOL000011	(2R,3R)-3-(4-hydroxy-3-methoxy-phenyl)-5-methoxy-2-methylol-2,3-dihydropyrano[5,6-h][1,4]benzodioxin-9-one	Protein kinase C delta type	PRKCD
13	MOL000011	(2R,3R)-3-(4-hydroxy-3-methoxy-phenyl)-5-methoxy-2-methylol-2,3-dihydropyrano[5,6-h][1,4]benzodioxin-9-one	Cathepsin L2	CTSV
14	MOL000011	(2R,3R)-3-(4-hydroxy-3-methoxy-phenyl)-5-methoxy-2-methylol-2,3-dihydropyrano[5,6-h][1,4]benzodioxin-9-one	Wee1-like protein kinase	WEE1
15	MOL000011	(2R,3R)-3-(4-hydroxy-3-methoxy-phenyl)-5-methoxy-2-methylol-2,3-dihydropyrano[5,6-h][1,4]benzodioxin-9-one	DNA topoisomerase 1	TOP1
16	MOL000011	(2R,3R)-3-(4-hydroxy-3-methoxy-phenyl)-5-methoxy-2-methylol-2,3-dihydropyrano[5,6-h][1,4]benzodioxin-9-one	Proto-oncogene tyrosine-protein kinase receptor Ret	RET

17	MOL000011	(2R,3R)-3-(4-hydroxy-3-methoxy-phenyl)-5-methoxy-2-methylol-2,3-dihydropyrano[5,6-h][1,4]benzodioxin-9-one	Leucyl-cystinyl aminopeptidase	LNPEP
18	MOL000011	(2R,3R)-3-(4-hydroxy-3-methoxy-phenyl)-5-methoxy-2-methylol-2,3-dihydropyrano[5,6-h][1,4]benzodioxin-9-one	Serine/threonine-protein kinase mTOR	MTOR
19	MOL000011	(2R,3R)-3-(4-hydroxy-3-methoxy-phenyl)-5-methoxy-2-methylol-2,3-dihydropyrano[5,6-h][1,4]benzodioxin-9-one	Serine/threonine-protein kinase B-raf	BRAF
20	MOL000011	(2R,3R)-3-(4-hydroxy-3-methoxy-phenyl)-5-methoxy-2-methylol-2,3-dihydropyrano[5,6-h][1,4]benzodioxin-9-one	Adenosine receptor A3	ADORA3
21	MOL000011	(2R,3R)-3-(4-hydroxy-3-methoxy-phenyl)-5-methoxy-2-methylol-2,3-dihydropyrano[5,6-h][1,4]benzodioxin-9-one	Phosphatidylinositol 4-phosphate 3-kinase C2 domain-containing subunit alpha	PIK3C2A
22	MOL000011	(2R,3R)-3-(4-hydroxy-3-methoxy-phenyl)-5-methoxy-2-methylol-2,3-dihydropyrano[5,6-h][1,4]benzodioxin-9-one	Phosphatidylinositol 3-kinase C2 domain-containing subunit gamma	PIK3C2G
23	MOL000011	(2R,3R)-3-(4-hydroxy-3-methoxy-phenyl)-5-methoxy-2-methylol-2,3-dihydropyrano[5,6-h][1,4]benzodioxin-9-one	Phosphatidylinositol 5-phosphate 4-kinase type-2 gamma	PIP4K2C
24	MOL000011	(2R,3R)-3-(4-hydroxy-3-methoxy-phenyl)-5-methoxy-2-methylol-2,3-dihydropyrano[5,6-h][1,4]benzodioxin-9-one	Receptor-type tyrosine-protein kinase FLT3	FLT3
25	MOL000011	(2R,3R)-3-(4-hydroxy-3-methoxy-phenyl)-5-methoxy-2-methylol-2,3-dihydropyrano[5,6-h][1,4]benzodioxin-9-one	Myosin light chain kinase, smooth muscle	MYLK
26	MOL000011	(2R,3R)-3-(4-hydroxy-3-methoxy-phenyl)-5-methoxy-2-methylol-2,3-dihydropyrano[5,6-h][1,4]benzodioxin-9-one	Death-associated protein kinase 3	DAPK3
27	MOL000011	(2R,3R)-3-(4-hydroxy-3-methoxy-phenyl)-5-methoxy-2-methylol-2,3-dihydropyrano[5,6-h][1,4]benzodioxin-9-one	Tyrosine-protein kinase JAK1	JAK1

28	MOL000011	(2R,3R)-3-(4-hydroxy-3-methoxy-phenyl)-5-methoxy-2-methylol-2,3-dihydropyrano[5,6-h][1,4]benzodioxin-9-one	Cyclin-dependent kinase 7	CDK7
29	MOL000011	(2R,3R)-3-(4-hydroxy-3-methoxy-phenyl)-5-methoxy-2-methylol-2,3-dihydropyrano[5,6-h][1,4]benzodioxin-9-one	Death-associated protein kinase 2	DAPK2
30	MOL000011	(2R,3R)-3-(4-hydroxy-3-methoxy-phenyl)-5-methoxy-2-methylol-2,3-dihydropyrano[5,6-h][1,4]benzodioxin-9-one	Phosphatidylinositol 4,5-bisphosphate 3-kinase catalytic subunit delta isoform	PIK3CD
31	MOL000011	(2R,3R)-3-(4-hydroxy-3-methoxy-phenyl)-5-methoxy-2-methylol-2,3-dihydropyrano[5,6-h][1,4]benzodioxin-9-one	DNA-dependent protein kinase catalytic subunit	PRKDC
32	MOL000011	(2R,3R)-3-(4-hydroxy-3-methoxy-phenyl)-5-methoxy-2-methylol-2,3-dihydropyrano[5,6-h][1,4]benzodioxin-9-one	Phosphatidylinositol 4,5-bisphosphate 3-kinase catalytic subunit beta isoform	PIK3CB
33	MOL000011	(2R,3R)-3-(4-hydroxy-3-methoxy-phenyl)-5-methoxy-2-methylol-2,3-dihydropyrano[5,6-h][1,4]benzodioxin-9-one	Phosphatidylinositol 4,5-bisphosphate 3-kinase catalytic subunit gamma isoform	PIK3CG
34	MOL000011	(2R,3R)-3-(4-hydroxy-3-methoxy-phenyl)-5-methoxy-2-methylol-2,3-dihydropyrano[5,6-h][1,4]benzodioxin-9-one	Phosphatidylinositol 4-kinase beta	PI4KB
35	MOL000011	(2R,3R)-3-(4-hydroxy-3-methoxy-phenyl)-5-methoxy-2-methylol-2,3-dihydropyrano[5,6-h][1,4]benzodioxin-9-one	Interstitial collagenase	MMP1
36	MOL000011	(2R,3R)-3-(4-hydroxy-3-methoxy-phenyl)-5-methoxy-2-methylol-2,3-dihydropyrano[5,6-h][1,4]benzodioxin-9-one	Inhibitor of nuclear factor kappa-B kinase subunit alpha	CHUK
37	MOL000011	(2R,3R)-3-(4-hydroxy-3-methoxy-phenyl)-5-methoxy-2-methylol-2,3-dihydropyrano[5,6-h][1,4]benzodioxin-9-one	Non-receptor tyrosine-protein kinase TYK2	TYK2
38	MOL000011	(2R,3R)-3-(4-hydroxy-3-methoxy-phenyl)-5-methoxy-2-methylol-2,3-dihydropyrano[5,6-h][1,4]benzodioxin-9-one	Casein kinase II subunit alpha	CSNK2A1

39	MOL000011	(2R,3R)-3-(4-hydroxy-3-methoxy-phenyl)-5-methoxy-2-methylol-2,3-dihydropyrano[5,6-h][1,4]benzodioxin-9-one	Casein kinase II subunit alpha'	CSNK2A2
40	MOL000011	(2R,3R)-3-(4-hydroxy-3-methoxy-phenyl)-5-methoxy-2-methylol-2,3-dihydropyrano[5,6-h][1,4]benzodioxin-9-one	Dual specificity protein kinase CLK2	CLK2
41	MOL000011	(2R,3R)-3-(4-hydroxy-3-methoxy-phenyl)-5-methoxy-2-methylol-2,3-dihydropyrano[5,6-h][1,4]benzodioxin-9-one	Dual specificity protein kinase CLK3	CLK3
42	MOL000011	(2R,3R)-3-(4-hydroxy-3-methoxy-phenyl)-5-methoxy-2-methylol-2,3-dihydropyrano[5,6-h][1,4]benzodioxin-9-one	Dual specificity tyrosine-phosphorylation-regulated kinase 2	DYRK2
43	MOL000011	(2R,3R)-3-(4-hydroxy-3-methoxy-phenyl)-5-methoxy-2-methylol-2,3-dihydropyrano[5,6-h][1,4]benzodioxin-9-one	Serine/threonine-protein kinase MRCK alpha	CDC42BPA
44	MOL000011	(2R,3R)-3-(4-hydroxy-3-methoxy-phenyl)-5-methoxy-2-methylol-2,3-dihydropyrano[5,6-h][1,4]benzodioxin-9-one	Homeodomain-interacting protein kinase 2	HIPK2
45	MOL000011	(2R,3R)-3-(4-hydroxy-3-methoxy-phenyl)-5-methoxy-2-methylol-2,3-dihydropyrano[5,6-h][1,4]benzodioxin-9-one	Homeodomain-interacting protein kinase 3	HIPK3
46	MOL000011	(2R,3R)-3-(4-hydroxy-3-methoxy-phenyl)-5-methoxy-2-methylol-2,3-dihydropyrano[5,6-h][1,4]benzodioxin-9-one	Homeodomain-interacting protein kinase 1	HIPK1
47	MOL000011	(2R,3R)-3-(4-hydroxy-3-methoxy-phenyl)-5-methoxy-2-methylol-2,3-dihydropyrano[5,6-h][1,4]benzodioxin-9-one	Dual specificity tyrosine-phosphorylation-regulated kinase 1B	DYRK1B
48	MOL000011	(2R,3R)-3-(4-hydroxy-3-methoxy-phenyl)-5-methoxy-2-methylol-2,3-dihydropyrano[5,6-h][1,4]benzodioxin-9-one	Phosphatidylinositol 4-phosphate 3-kinase C2 domain-containing subunit beta	PIK3C2B
49	MOL000011	(2R,3R)-3-(4-hydroxy-3-methoxy-phenyl)-5-methoxy-2-methylol-2,3-dihydropyrano[5,6-h][1,4]benzodioxin-9-one	LIM domain kinase 2	LIMK2

50	MOL000011	(2R,3R)-3-(4-hydroxy-3-methoxy-phenyl)-5-methoxy-2-methylol-2,3-dihydropyrano[5,6-h][1,4]benzodioxin-9-one	Serine/threonine-protein kinase RIO2	RIOK2
51	MOL000011	(2R,3R)-3-(4-hydroxy-3-methoxy-phenyl)-5-methoxy-2-methylol-2,3-dihydropyrano[5,6-h][1,4]benzodioxin-9-one	Mitogen-activated protein kinase kinase kinase 19	MAP3K19
52	MOL000011	(2R,3R)-3-(4-hydroxy-3-methoxy-phenyl)-5-methoxy-2-methylol-2,3-dihydropyrano[5,6-h][1,4]benzodioxin-9-one	C-X-C chemokine receptor type 2	CXCR2
53	MOL000011	(2R,3R)-3-(4-hydroxy-3-methoxy-phenyl)-5-methoxy-2-methylol-2,3-dihydropyrano[5,6-h][1,4]benzodioxin-9-one	C-X-C chemokine receptor type 1	CXCR1
54	MOL000011	(2R,3R)-3-(4-hydroxy-3-methoxy-phenyl)-5-methoxy-2-methylol-2,3-dihydropyrano[5,6-h][1,4]benzodioxin-9-one	Mitogen-activated protein kinase kinase kinase 14	MAP3K14
55	MOL000011	(2R,3R)-3-(4-hydroxy-3-methoxy-phenyl)-5-methoxy-2-methylol-2,3-dihydropyrano[5,6-h][1,4]benzodioxin-9-one	Eukaryotic initiation factor 4A-I	EIF4A1
56	MOL000011	(2R,3R)-3-(4-hydroxy-3-methoxy-phenyl)-5-methoxy-2-methylol-2,3-dihydropyrano[5,6-h][1,4]benzodioxin-9-one	Serine/threonine-protein kinase PAK 3	PAK3
57	MOL000011	(2R,3R)-3-(4-hydroxy-3-methoxy-phenyl)-5-methoxy-2-methylol-2,3-dihydropyrano[5,6-h][1,4]benzodioxin-9-one	Serine/threonine-protein kinase PAK 2	PAK2
58	MOL000011	(2R,3R)-3-(4-hydroxy-3-methoxy-phenyl)-5-methoxy-2-methylol-2,3-dihydropyrano[5,6-h][1,4]benzodioxin-9-one	Serine/threonine-protein kinase PAK 1	PAK1
59	MOL000011	(2R,3R)-3-(4-hydroxy-3-methoxy-phenyl)-5-methoxy-2-methylol-2,3-dihydropyrano[5,6-h][1,4]benzodioxin-9-one	Tyrosine-protein kinase ABL1	ABL1
60	MOL000011	(2R,3R)-3-(4-hydroxy-3-methoxy-phenyl)-5-methoxy-2-methylol-2,3-dihydropyrano[5,6-h][1,4]benzodioxin-9-one	Alkaline phosphatase, tissue-nonspecific isozyme	ALPL

61	MOL000011	(2R,3R)-3-(4-hydroxy-3-methoxy-phenyl)-5-methoxy-2-methylol-2,3-dihydropyrano[5,6-h][1,4]benzodioxin-9-one	Phospholipase A-2-activating protein	PLAA
62	MOL000011	(2R,3R)-3-(4-hydroxy-3-methoxy-phenyl)-5-methoxy-2-methylol-2,3-dihydropyrano[5,6-h][1,4]benzodioxin-9-one	Disintegrin and metalloproteinase domain-containing protein 17	ADAM17
63	MOL000011	(2R,3R)-3-(4-hydroxy-3-methoxy-phenyl)-5-methoxy-2-methylol-2,3-dihydropyrano[5,6-h][1,4]benzodioxin-9-one	Cystic fibrosis transmembrane conductance regulator	CFTR
64	MOL000011	(2R,3R)-3-(4-hydroxy-3-methoxy-phenyl)-5-methoxy-2-methylol-2,3-dihydropyrano[5,6-h][1,4]benzodioxin-9-one	Adenylate cyclase type 1	ADCY1
65	MOL000011	(2R,3R)-3-(4-hydroxy-3-methoxy-phenyl)-5-methoxy-2-methylol-2,3-dihydropyrano[5,6-h][1,4]benzodioxin-9-one	Coagulation factor IX	F9
66	MOL000011	(2R,3R)-3-(4-hydroxy-3-methoxy-phenyl)-5-methoxy-2-methylol-2,3-dihydropyrano[5,6-h][1,4]benzodioxin-9-one	Vascular endothelial growth factor receptor 1	FLT1
67	MOL000011	(2R,3R)-3-(4-hydroxy-3-methoxy-phenyl)-5-methoxy-2-methylol-2,3-dihydropyrano[5,6-h][1,4]benzodioxin-9-one	Transcription factor Jun	JUN
68	MOL000011	(2R,3R)-3-(4-hydroxy-3-methoxy-phenyl)-5-methoxy-2-methylol-2,3-dihydropyrano[5,6-h][1,4]benzodioxin-9-one	High affinity nerve growth factor receptor	NTRK1
69	MOL000011	(2R,3R)-3-(4-hydroxy-3-methoxy-phenyl)-5-methoxy-2-methylol-2,3-dihydropyrano[5,6-h][1,4]benzodioxin-9-one	Matrilysin	MMP7
70	MOL000011	(2R,3R)-3-(4-hydroxy-3-methoxy-phenyl)-5-methoxy-2-methylol-2,3-dihydropyrano[5,6-h][1,4]benzodioxin-9-one	Disintegrin and metalloproteinase domain-containing protein 10	ADAM10
71	MOL000011	(2R,3R)-3-(4-hydroxy-3-methoxy-phenyl)-5-methoxy-2-methylol-2,3-dihydropyrano[5,6-h][1,4]benzodioxin-9-one	Mitogen-activated protein kinase kinase kinase 7	MAP3K7

72	MOL000011	(2R,3R)-3-(4-hydroxy-3-methoxy-phenyl)-5-methoxy-2-methylol-2,3-dihydropyrano[5,6-h][1,4]benzodioxin-9-one	Endothelin receptor type B	EDNRB
73	MOL000011	(2R,3R)-3-(4-hydroxy-3-methoxy-phenyl)-5-methoxy-2-methylol-2,3-dihydropyrano[5,6-h][1,4]benzodioxin-9-one	Endothelin-1 receptor	EDNRA
74	MOL000011	(2R,3R)-3-(4-hydroxy-3-methoxy-phenyl)-5-methoxy-2-methylol-2,3-dihydropyrano[5,6-h][1,4]benzodioxin-9-one	Sodium/glucose cotransporter 2	SLC5A2
75	MOL000011	(2R,3R)-3-(4-hydroxy-3-methoxy-phenyl)-5-methoxy-2-methylol-2,3-dihydropyrano[5,6-h][1,4]benzodioxin-9-one	Histone deacetylase 6	HDAC6
76	MOL000011	(2R,3R)-3-(4-hydroxy-3-methoxy-phenyl)-5-methoxy-2-methylol-2,3-dihydropyrano[5,6-h][1,4]benzodioxin-9-one	Histone deacetylase 2	HDAC2
77	MOL000011	(2R,3R)-3-(4-hydroxy-3-methoxy-phenyl)-5-methoxy-2-methylol-2,3-dihydropyrano[5,6-h][1,4]benzodioxin-9-one	P2Y purinoceptor 12	P2RY12
78	MOL000011	(2R,3R)-3-(4-hydroxy-3-methoxy-phenyl)-5-methoxy-2-methylol-2,3-dihydropyrano[5,6-h][1,4]benzodioxin-9-one	Histone deacetylase 1	HDAC1
79	MOL000020	12-senecioyl-2E,8E,10E-atractylentriol	DNA topoisomerase 1	TOP1
80	MOL000020	12-senecioyl-2E,8E,10E-atractylentriol	RAC-alpha serine/threonine-protein kinase	AKT1
81	MOL000020	12-senecioyl-2E,8E,10E-atractylentriol	GTPase HRas	HRAS
82	MOL000020	12-senecioyl-2E,8E,10E-atractylentriol	Carbonic anhydrase 1	CA1
83	MOL000020	12-senecioyl-2E,8E,10E-atractylentriol	Carbonic anhydrase 12	CA12
84	MOL000020	12-senecioyl-2E,8E,10E-atractylentriol	Carbonic anhydrase 9	CA9
85	MOL000020	12-senecioyl-2E,8E,10E-atractylentriol	von Hippel-Lindau disease tumor suppressor	VHL
86	MOL000020	12-senecioyl-2E,8E,10E-atractylentriol	Insulin-like growth factor 1 receptor	IGF1R

87	MOL000020	12-senecioyl-2E,8E,10E-atractylent riol	Mitogen-activated protein kinase 14	MAPK14
88	MOL000020	12-senecioyl-2E,8E,10E-atractylent riol	Mitogen-activated protein kinase 1	MAPK1
89	MOL000020	12-senecioyl-2E,8E,10E-atractylent riol	Aurora kinase A	AURKA
90	MOL000020	12-senecioyl-2E,8E,10E-atractylent riol	Aurora kinase B	AURKB
91	MOL000020	12-senecioyl-2E,8E,10E-atractylent riol	Epidermal growth factor receptor	EGFR
92	MOL000020	12-senecioyl-2E,8E,10E-atractylent riol	Glycogen	GYS1
93	MOL000020	12-senecioyl-2E,8E,10E-atractylent riol	Tumor necrosis factor	TNF
94	MOL000020	12-senecioyl-2E,8E,10E-atractylent riol	Adenosine receptor A2a	ADORA2A
95	MOL000020	12-senecioyl-2E,8E,10E-atractylent riol	Sphingosine 1-phosphate receptor 1	S1PR1
96	MOL000020	12-senecioyl-2E,8E,10E-atractylent riol	High affinity nerve growth factor receptor	NTRK1
97	MOL000020	12-senecioyl-2E,8E,10E-atractylent riol	DNA-dependent protein kinase catalytic subunit	PRKDC
98	MOL000020	12-senecioyl-2E,8E,10E-atractylent riol	M-phase inducer phosphatase 2	CDC25B
99	MOL000020	12-senecioyl-2E,8E,10E-atractylent riol	Protein kinase C delta type	PRKCD
100	MOL000020	12-senecioyl-2E,8E,10E-atractylent riol	Vitamin D3 receptor	VDR
101	MOL000020	12-senecioyl-2E,8E,10E-atractylent riol	Aldo-keto reductase family 1 member B1	AKR1B1
102	MOL000020	12-senecioyl-2E,8E,10E-atractylent riol	Fatty-acid amide hydrolase 1	FAAH
103	MOL000020	12-senecioyl-2E,8E,10E-atractylent riol	ATP-dependent translocase ABCB1	ABCB1
104	MOL000020	12-senecioyl-2E,8E,10E-atractylent riol	Transient receptor potential cation channel subfamily V member 1	TRPV1
105	MOL000020	12-senecioyl-2E,8E,10E-atractylent riol	Prostaglandin G/H synthase 2	PTGS2
106	MOL000020	12-senecioyl-2E,8E,10E-atractylent riol	cGMP-inhibited 3',5'-cyclic phosphodiesterase 3A	PDE3A
107	MOL000020	12-senecioyl-2E,8E,10E-atractylent riol	cGMP-inhibited 3',5'-cyclic phosphodiesterase 3B	PDE3B
108	MOL000020	12-senecioyl-2E,8E,10E-atractylent riol	Disintegrin and metalloproteinase domain-containing protein 17	ADAM17

109	MOL000020	12-senecioyl-2E,8E,10E-atractylent riol	Gamma-aminobutyric acid receptor subunit alpha-5	GABRA 5
110	MOL000020	12-senecioyl-2E,8E,10E-atractylent riol	Hexokinase-4	GCK
111	MOL000020	12-senecioyl-2E,8E,10E-atractylent riol	Uridine phosphorylase 1	UPP1
112	MOL000020	12-senecioyl-2E,8E,10E-atractylent riol	1-phosphatidylinositol 4,5-bisphosphate phosphodiesterase gamma-1	PLCG1
113	MOL000020	12-senecioyl-2E,8E,10E-atractylent riol	Tyrosine-protein kinase ABL1	ABL1
114	MOL000020	12-senecioyl-2E,8E,10E-atractylent riol	High affinity cAMP-specific 3',5'-cyclic phosphodiesterase 7A	PDE7A
115	MOL000020	12-senecioyl-2E,8E,10E-atractylent riol	Glycogen phosphorylase, liver form	PYGL
116	MOL000020	12-senecioyl-2E,8E,10E-atractylent riol	Protein kinase C gamma type	PRKCG
117	MOL000020	12-senecioyl-2E,8E,10E-atractylent riol	Protein kinase C beta type	PRKCB
118	MOL000020	12-senecioyl-2E,8E,10E-atractylent riol	Proto-oncogene vav	VAV1
119	MOL000020	12-senecioyl-2E,8E,10E-atractylent riol	Protein kinase C epsilon type	PRKCE
120	MOL000020	12-senecioyl-2E,8E,10E-atractylent riol	Protein kinase C eta type	PRKCH
121	MOL000020	12-senecioyl-2E,8E,10E-atractylent riol	Protein kinase C theta type	PRKCQ
122	MOL000020	12-senecioyl-2E,8E,10E-atractylent riol	Activated CDC42 kinase 1	TNK2
123	MOL000020	12-senecioyl-2E,8E,10E-atractylent riol	Prostate-specific antigen	KLK3
124	MOL000020	12-senecioyl-2E,8E,10E-atractylent riol	Sphingosine 1-phosphate receptor 3	S1PR3
125	MOL000020	12-senecioyl-2E,8E,10E-atractylent riol	Mitogen-activated protein kinase 8	MAPK8
126	MOL000020	12-senecioyl-2E,8E,10E-atractylent riol	C-X-C chemokine receptor type 2	CXCR2
127	MOL000020	12-senecioyl-2E,8E,10E-atractylent riol	Voltage-dependent T-type calcium channel subunit alpha-1H	CACNA 1H
128	MOL000020	12-senecioyl-2E,8E,10E-atractylent riol	Cyclin-T1	CCNT1
129	MOL000020	12-senecioyl-2E,8E,10E-atractylent riol	Adenosine receptor A2b	ADORA 2B
130	MOL000020	12-senecioyl-2E,8E,10E-atractylent	Gastrin/cholecystokinin type B	CCKBR

		riol	receptor	
131	MOL000021	14-acetyl-12-senecioyl-2E,8E,10E-atractylentriol	Sodium- and chloride-dependent GABA transporter 1	SLC6A1
132	MOL000021	14-acetyl-12-senecioyl-2E,8E,10E-atractylentriol	Geranylgeranyl transferase type-1 subunit beta	PGGT1B
133	MOL000021	14-acetyl-12-senecioyl-2E,8E,10E-atractylentriol	Adenosine receptor A1	ADORA1
134	MOL000021	14-acetyl-12-senecioyl-2E,8E,10E-atractylentriol	Prothrombin	F2
135	MOL000021	14-acetyl-12-senecioyl-2E,8E,10E-atractylentriol	Histone-lysine N-methyltransferase EZH2	EZH2
136	MOL000021	14-acetyl-12-senecioyl-2E,8E,10E-atractylentriol	Orexin receptor type 2	HCRTR2
137	MOL000021	14-acetyl-12-senecioyl-2E,8E,10E-atractylentriol	Orexin/Hypocretin receptor type 1	HCRTR1
138	MOL000021	14-acetyl-12-senecioyl-2E,8E,10E-atractylentriol	Sterol O-acyltransferase 1	SOAT1
139	MOL000021	14-acetyl-12-senecioyl-2E,8E,10E-atractylentriol	Neuromedin-K receptor	TACR3
140	MOL000021	14-acetyl-12-senecioyl-2E,8E,10E-atractylentriol	cAMP and cAMP-inhibited cGMP 3',5'-cyclic phosphodiesterase 10A	PDE10A
141	MOL000021	14-acetyl-12-senecioyl-2E,8E,10E-atractylentriol	Procathepsin L	CTSL
142	MOL000021	14-acetyl-12-senecioyl-2E,8E,10E-atractylentriol	Casein kinase I isoform alpha	CSNK1A1
143	MOL000021	14-acetyl-12-senecioyl-2E,8E,10E-atractylentriol	Cyclin-dependent kinase 5	CDK5
144	MOL000021	14-acetyl-12-senecioyl-2E,8E,10E-atractylentriol	Sonic hedgehog protein	SHH
145	MOL000021	14-acetyl-12-senecioyl-2E,8E,10E-atractylentriol	Scavenger receptor class B member 1	SCARB1
146	MOL000021	14-acetyl-12-senecioyl-2E,8E,10E-atractylentriol	Kinesin-like protein KIF11	KIF11
147	MOL000021	14-acetyl-12-senecioyl-2E,8E,10E-atractylentriol	Translocator protein	TSPO
148	MOL000021	14-acetyl-12-senecioyl-2E,8E,10E-atractylentriol	Interstitial collagenase	MMP1
149	MOL000021	14-acetyl-12-senecioyl-2E,8E,10E-atractylentriol	Tyrosine-protein kinase Mer	MERTK
150	MOL000021	14-acetyl-12-senecioyl-2E,8E,10E-atractylentriol	Amyloid-beta precursor protein	APP
151	MOL000021	14-acetyl-12-senecioyl-2E,8E,10E-atractylentriol	Peptidyl-prolyl cis-trans isomerase FKBP5	FKBP5
152	MOL000021	14-acetyl-12-senecioyl-2E,8E,10E-	N-lysine methyltransferase	SMYD2

		atractylentriol	SMYD2	
153	MOL000021	14-acetyl-12-senecioyl-2E,8E,10E-atractylentriol	cAMP-specific 3',5'-cyclic phosphodiesterase 4B	PDE4B
154	MOL000021	14-acetyl-12-senecioyl-2E,8E,10E-atractylentriol	Protein smoothened	SMO
155	MOL000021	14-acetyl-12-senecioyl-2E,8E,10E-atractylentriol	E3 ubiquitin-protein ligase Mdm2	MDM2
156	MOL000021	14-acetyl-12-senecioyl-2E,8E,10E-atractylentriol	Kappa-type opioid receptor	OPRK1
157	MOL000021	14-acetyl-12-senecioyl-2E,8E,10E-atractylentriol	3-phosphoinositide-dependent protein kinase 1	PDPK1
158	MOL000021	14-acetyl-12-senecioyl-2E,8E,10E-atractylentriol	Receptor tyrosine-protein kinase erbB-2	ERBB2
159	MOL000021	14-acetyl-12-senecioyl-2E,8E,10E-atractylentriol	Gastrin/cholecystokinin type B receptor	CCKBR
160	MOL000021	14-acetyl-12-senecioyl-2E,8E,10E-atractylentriol	Proto-oncogene tyrosine-protein kinase Src	SRC
161	MOL000028	α-Amyrin	Nuclear receptor subfamily 1 group I member 3	NR1I3
162	MOL000028	α-Amyrin	NPC1-like intracellular cholesterol transporter 1	NPC1L1
163	MOL000028	α-Amyrin	3-hydroxy-3-methylglutaryl-coenzyme A reductase	HMGCR
164	MOL000028	α-Amyrin	Lanosterol 14-alpha demethylase	CYP51A1
165	MOL000028	α-Amyrin	Sex hormone-binding globulin	SHBG
166	MOL000028	α-Amyrin	Steroid 17-alpha-hydroxylase/17,20 lyase	CYP17A1
167	MOL000028	α-Amyrin	Oxysterols receptor LXR-alpha	NR1H3
168	MOL000028	α-Amyrin	Tyrosine-protein phosphatase non-receptor type 6	PTPN6
169	MOL000028	α-Amyrin	Tyrosine-protein phosphatase non-receptor type 2	PTPN2
170	MOL000028	α-Amyrin	Fatty acid-binding protein, adipocyte	FABP4
171	MOL000028	α-Amyrin	Fatty acid-binding protein, heart	FABP3
172	MOL000028	α-Amyrin	Fatty acid-binding protein 5	FABP5
173	MOL000028	α-Amyrin	M-phase inducer phosphatase 2	CDC25B
174	MOL000028	α-Amyrin	Stearoyl-CoA desaturase	SCD
175	MOL000028	α-Amyrin	Corticosteroid-binding globulin	SERPINA6
176	MOL000028	α-Amyrin	cAMP-specific 3',5'-cyclic phosphodiesterase 4D	PDE4D
177	MOL000028	α-Amyrin	Receptor-type tyrosine-protein	PTPRF

			phosphatase F	
178	MOL000028	α-Amyrin	Phospholipase A2	PLA2G1B
179	MOL000028	α-Amyrin	Cocaine esterase	CES2
180	MOL000028	α-Amyrin	M-phase inducer phosphatase 1	CDC25A
181	MOL000028	α-Amyrin	11-beta-hydroxysteroid dehydrogenase type 2	HSD11B2
182	MOL000028	α-Amyrin	Mitogen-activated protein kinase 3	MAPK3
183	MOL000028	α-Amyrin	Tyrosine-protein phosphatase non-receptor type 11	PTPN11
184	MOL000028	α-Amyrin	Cannabinoid receptor 1	CNR1
185	MOL000028	α-Amyrin	Polyunsaturated fatty acid 5-lipoxygenase	ALOX5
186	MOL000028	α-Amyrin	11-beta-hydroxysteroid dehydrogenase type 2	HSD11B2
187	MOL000028	α-Amyrin	11-beta-hydroxysteroid dehydrogenase 1	HSD11B1
188	MOL000028	α-Amyrin	Peptidyl-prolyl cis-trans isomerase NIMA-interacting 1	PIN1
189	MOL000028	α-Amyrin	Ubiquitin carboxyl-terminal hydrolase 7	USP7
190	MOL000033	(3S,8S,9S,10R,13R,14S,17R)-10,13-dimethyl-17-[(2R,5S)-5-propan-2-yloctan-2-yl]-2,3,4,7,8,9,11,12,14,15,16,17-dodecahydro-1H-cyclopenta[a]phenanthren-3-ol	Lanosterol 14-alpha demethylase	CYP51A1
191	MOL000033	(3S,8S,9S,10R,13R,14S,17R)-10,13-dimethyl-17-[(2R,5S)-5-propan-2-yloctan-2-yl]-2,3,4,7,8,9,11,12,14,15,16,17-dodecahydro-1H-cyclopenta[a]phenanthren-3-ol	Androgen receptor	AR
192	MOL000033	(3S,8S,9S,10R,13R,14S,17R)-10,13-dimethyl-17-[(2R,5S)-5-propan-2-yloctan-2-yl]-2,3,4,7,8,9,11,12,14,15,16,17-dodecahydro-1H-cyclopenta[a]phenanthren-3-ol	NPC1-like intracellular cholesterol transporter 1	NPC1L1
193	MOL000033	(3S,8S,9S,10R,13R,14S,17R)-10,13-dimethyl-17-[(2R,5S)-5-propan-2-yloctan-2-yl]-2,3,4,7,8,9,11,12,14,15,16,17-dodecahydro-1H-cyclopenta[a]phenanthren-3-ol	Nuclear receptor ROR-gamma	RORC
194	MOL000033	(3S,8S,9S,10R,13R,14S,17R)-10,13-dimethyl-17-[(2R,5S)-5-propan-2	Steroid 17-alpha-hydroxylase/17,20 lyase	CYP17A1

		-yloctan-2-yl]-2,3,4,7,8,9,11,12,14,15,16,17-dodecahydro-1H-cyclopenta[a]phenanthren-3-ol		
195	MOL000033	(3S,8S,9S,10R,13R,14S,17R)-10,13-dimethyl-17-[(2R,5S)-5-propan-2-yloctan-2-yl]-2,3,4,7,8,9,11,12,14,15,16,17-dodecahydro-1H-cyclopenta[a]phenanthren-3-ol	Aromatase	CYP19A1
196	MOL000033	(3S,8S,9S,10R,13R,14S,17R)-10,13-dimethyl-17-[(2R,5S)-5-propan-2-yloctan-2-yl]-2,3,4,7,8,9,11,12,14,15,16,17-dodecahydro-1H-cyclopenta[a]phenanthren-3-ol	Sterol regulatory element-binding protein 2	SREBF2
197	MOL000033	(3S,8S,9S,10R,13R,14S,17R)-10,13-dimethyl-17-[(2R,5S)-5-propan-2-yloctan-2-yl]-2,3,4,7,8,9,11,12,14,15,16,17-dodecahydro-1H-cyclopenta[a]phenanthren-3-ol	Estrogen receptor	ESR1
198	MOL000033	(3S,8S,9S,10R,13R,14S,17R)-10,13-dimethyl-17-[(2R,5S)-5-propan-2-yloctan-2-yl]-2,3,4,7,8,9,11,12,14,15,16,17-dodecahydro-1H-cyclopenta[a]phenanthren-3-ol	Sex hormone-binding globulin	SHBG
199	MOL000033	(3S,8S,9S,10R,13R,14S,17R)-10,13-dimethyl-17-[(2R,5S)-5-propan-2-yloctan-2-yl]-2,3,4,7,8,9,11,12,14,15,16,17-dodecahydro-1H-cyclopenta[a]phenanthren-3-ol	Estrogen receptor beta	ESR2
200	MOL000033	(3S,8S,9S,10R,13R,14S,17R)-10,13-dimethyl-17-[(2R,5S)-5-propan-2-yloctan-2-yl]-2,3,4,7,8,9,11,12,14,15,16,17-dodecahydro-1H-cyclopenta[a]phenanthren-3-ol	Tyrosine-protein phosphatase non-receptor type 1	PTPN1
201	MOL000033	(3S,8S,9S,10R,13R,14S,17R)-10,13-dimethyl-17-[(2R,5S)-5-propan-2-yloctan-2-yl]-2,3,4,7,8,9,11,12,14,15,16,17-dodecahydro-1H-cyclopenta[a]phenanthren-3-ol	Corticosteroid-binding globulin	SERPINA6
202	MOL000033	(3S,8S,9S,10R,13R,14S,17R)-10,13-dimethyl-17-[(2R,5S)-5-propan-2-yloctan-2-yl]-2,3,4,7,8,9,11,12,14,15,16,17-dodecahydro-1H-cyclopenta[a]phenanthren-3-ol	Cholinesterase	BCHE
203	MOL000033	(3S,8S,9S,10R,13R,14S,17R)-10,1	Sodium-dependent serotonin	SLC6A4

		3-dimethyl-17-[(2R,5S)-5-propan-2-yloctan-2-yl]-2,3,4,7,8,9,11,12,14,15,16,17-dodecahydro-1H-cyclopenta[a]phenanthren-3-ol	transporter	
204	MOL000033	(3S,8S,9S,10R,13R,14S,17R)-10,13-dimethyl-17-[(2R,5S)-5-propan-2-yloctan-2-yl]-2,3,4,7,8,9,11,12,14,15,16,17-dodecahydro-1H-cyclopenta[a]phenanthren-3-ol	Acetylcholinesterase	ACHE
205	MOL000033	(3S,8S,9S,10R,13R,14S,17R)-10,13-dimethyl-17-[(2R,5S)-5-propan-2-yloctan-2-yl]-2,3,4,7,8,9,11,12,14,15,16,17-dodecahydro-1H-cyclopenta[a]phenanthren-3-ol	Glucose-6-phosphate 1-dehydrogenase	G6PD
206	MOL000033	(3S,8S,9S,10R,13R,14S,17R)-10,13-dimethyl-17-[(2R,5S)-5-propan-2-yloctan-2-yl]-2,3,4,7,8,9,11,12,14,15,16,17-dodecahydro-1H-cyclopenta[a]phenanthren-3-ol	Cytochrome P450 2C19	CYP2C19
207	MOL000033	(3S,8S,9S,10R,13R,14S,17R)-10,13-dimethyl-17-[(2R,5S)-5-propan-2-yloctan-2-yl]-2,3,4,7,8,9,11,12,14,15,16,17-dodecahydro-1H-cyclopenta[a]phenanthren-3-ol	M-phase inducer phosphatase 1	CDC25A
208	MOL000033	(3S,8S,9S,10R,13R,14S,17R)-10,13-dimethyl-17-[(2R,5S)-5-propan-2-yloctan-2-yl]-2,3,4,7,8,9,11,12,14,15,16,17-dodecahydro-1H-cyclopenta[a]phenanthren-3-ol	11-beta-hydroxysteroid dehydrogenase 1	HSD11B1
209	MOL000033	(3S,8S,9S,10R,13R,14S,17R)-10,13-dimethyl-17-[(2R,5S)-5-propan-2-yloctan-2-yl]-2,3,4,7,8,9,11,12,14,15,16,17-dodecahydro-1H-cyclopenta[a]phenanthren-3-ol	Cocaine esterase	CES2
210	MOL000033	(3S,8S,9S,10R,13R,14S,17R)-10,13-dimethyl-17-[(2R,5S)-5-propan-2-yloctan-2-yl]-2,3,4,7,8,9,11,12,14,15,16,17-dodecahydro-1H-cyclopenta[a]phenanthren-3-ol	Prostaglandin E2 receptor EP1 subtype	PTGER1
211	MOL000033	(3S,8S,9S,10R,13R,14S,17R)-10,13-dimethyl-17-[(2R,5S)-5-propan-2-yloctan-2-yl]-2,3,4,7,8,9,11,12,14,15,16,17-dodecahydro-1H-cyclopenta[a]phenanthren-3-ol	Prostaglandin E2 receptor EP2 subtype	PTGER2

212	MOL000033	(3S,8S,9S,10R,13R,14S,17R)-10,13-dimethyl-17-[(2R,5S)-5-propan-2-yloctan-2-yl]-2,3,4,7,8,9,11,12,14,15,16,17-dodecahydro-1H-cyclopenta[a]phenanthren-3-ol	Glycine receptor subunit alpha-1	GLRA1
213	MOL000033	(3S,8S,9S,10R,13R,14S,17R)-10,13-dimethyl-17-[(2R,5S)-5-propan-2-yloctan-2-yl]-2,3,4,7,8,9,11,12,14,15,16,17-dodecahydro-1H-cyclopenta[a]phenanthren-3-ol	7-dehydrocholesterol reductase	DHCR7
214	MOL000033	(3S,8S,9S,10R,13R,14S,17R)-10,13-dimethyl-17-[(2R,5S)-5-propan-2-yloctan-2-yl]-2,3,4,7,8,9,11,12,14,15,16,17-dodecahydro-1H-cyclopenta[a]phenanthren-3-ol	Squalene monooxygenase	SQLE
215	MOL000033	(3S,8S,9S,10R,13R,14S,17R)-10,13-dimethyl-17-[(2R,5S)-5-propan-2-yloctan-2-yl]-2,3,4,7,8,9,11,12,14,15,16,17-dodecahydro-1H-cyclopenta[a]phenanthren-3-ol	Tyrosine-protein phosphatase non-receptor type 6	PTPN6
216	MOL000033	(3S,8S,9S,10R,13R,14S,17R)-10,13-dimethyl-17-[(2R,5S)-5-propan-2-yloctan-2-yl]-2,3,4,7,8,9,11,12,14,15,16,17-dodecahydro-1H-cyclopenta[a]phenanthren-3-ol	Nitric oxide synthase, inducible	NOS2
217	MOL000033	(3S,8S,9S,10R,13R,14S,17R)-10,13-dimethyl-17-[(2R,5S)-5-propan-2-yloctan-2-yl]-2,3,4,7,8,9,11,12,14,15,16,17-dodecahydro-1H-cyclopenta[a]phenanthren-3-ol	Sigma non-opioid intracellular receptor 1	SIGMAR1
218	MOL000033	(3S,8S,9S,10R,13R,14S,17R)-10,13-dimethyl-17-[(2R,5S)-5-propan-2-yloctan-2-yl]-2,3,4,7,8,9,11,12,14,15,16,17-dodecahydro-1H-cyclopenta[a]phenanthren-3-ol	UDP-glucuronosyltransferase 2B7	UGT2B7
219	MOL000033	(3S,8S,9S,10R,13R,14S,17R)-10,13-dimethyl-17-[(2R,5S)-5-propan-2-yloctan-2-yl]-2,3,4,7,8,9,11,12,14,15,16,17-dodecahydro-1H-cyclopenta[a]phenanthren-3-ol	C-C chemokine receptor type 1	CCR1
220	MOL000033	(3S,8S,9S,10R,13R,14S,17R)-10,13-dimethyl-17-[(2R,5S)-5-propan-2-yloctan-2-yl]-2,3,4,7,8,9,11,12,14,15,16,17-dodecahydro-1H-cyclope	Prostaglandin E synthase	PTGES

		nta[a]phenanthren-3-ol		
221	MOL000033	(3S,8S,9S,10R,13R,14S,17R)-10,13-dimethyl-17-[(2R,5S)-5-propan-2-yloctan-2-yl]-2,3,4,7,8,9,11,12,14,15,16,17-dodecahydro-1H-cyclopenta[a]phenanthren-3-ol	cAMP-specific 3',5'-cyclic phosphodiesterase 4A	PDE4A
222	MOL000033	(3S,8S,9S,10R,13R,14S,17R)-10,13-dimethyl-17-[(2R,5S)-5-propan-2-yloctan-2-yl]-2,3,4,7,8,9,11,12,14,15,16,17-dodecahydro-1H-cyclopenta[a]phenanthren-3-ol	cAMP-specific 3',5'-cyclic phosphodiesterase 4B	PDE4B
223	MOL000033	(3S,8S,9S,10R,13R,14S,17R)-10,13-dimethyl-17-[(2R,5S)-5-propan-2-yloctan-2-yl]-2,3,4,7,8,9,11,12,14,15,16,17-dodecahydro-1H-cyclopenta[a]phenanthren-3-ol	cAMP-specific 3',5'-cyclic phosphodiesterase 4C	PDE4C
224	MOL000033	(3S,8S,9S,10R,13R,14S,17R)-10,13-dimethyl-17-[(2R,5S)-5-propan-2-yloctan-2-yl]-2,3,4,7,8,9,11,12,14,15,16,17-dodecahydro-1H-cyclopenta[a]phenanthren-3-ol	DNA polymerase beta	POLB
225	MOL000033	(3S,8S,9S,10R,13R,14S,17R)-10,13-dimethyl-17-[(2R,5S)-5-propan-2-yloctan-2-yl]-2,3,4,7,8,9,11,12,14,15,16,17-dodecahydro-1H-cyclopenta[a]phenanthren-3-ol	Prolyl endopeptidase	PREP
226	MOL000033	(3S,8S,9S,10R,13R,14S,17R)-10,13-dimethyl-17-[(2R,5S)-5-propan-2-yloctan-2-yl]-2,3,4,7,8,9,11,12,14,15,16,17-dodecahydro-1H-cyclopenta[a]phenanthren-3-ol	Delta	DHCR24
227	MOL000033	(3S,8S,9S,10R,13R,14S,17R)-10,13-dimethyl-17-[(2R,5S)-5-propan-2-yloctan-2-yl]-2,3,4,7,8,9,11,12,14,15,16,17-dodecahydro-1H-cyclopenta[a]phenanthren-3-ol	Alpha-crystallin B chain	CRYAB
228	MOL000033	(3S,8S,9S,10R,13R,14S,17R)-10,13-dimethyl-17-[(2R,5S)-5-propan-2-yloctan-2-yl]-2,3,4,7,8,9,11,12,14,15,16,17-dodecahydro-1H-cyclopenta[a]phenanthren-3-ol	G-protein coupled bile acid receptor 1	GPBAR1
229	MOL000033	(3S,8S,9S,10R,13R,14S,17R)-10,13-dimethyl-17-[(2R,5S)-5-propan-2-yloctan-2-yl]-2,3,4,7,8,9,11,12,14,	3-oxo-5-alpha-steroid 4-dehydrogenase 2	SRD5A2

		15,16,17-dodecahydro-1H-cyclopenta[a]phenanthren-3-ol		
230	MOL000033	(3S,8S,9S,10R,13R,14S,17R)-10,13-dimethyl-17-[(2R,5S)-5-propan-2-yloctan-2-yl]-2,3,4,7,8,9,11,12,14,15,16,17-dodecahydro-1H-cyclopenta[a]phenanthren-3-ol	Hepatic sodium/bile acid cotransporter	SLC10A1
231	MOL000033	(3S,8S,9S,10R,13R,14S,17R)-10,13-dimethyl-17-[(2R,5S)-5-propan-2-yloctan-2-yl]-2,3,4,7,8,9,11,12,14,15,16,17-dodecahydro-1H-cyclopenta[a]phenanthren-3-ol	ATP-binding cassette sub-family C member 4	ABCC4
232	MOL000033	(3S,8S,9S,10R,13R,14S,17R)-10,13-dimethyl-17-[(2R,5S)-5-propan-2-yloctan-2-yl]-2,3,4,7,8,9,11,12,14,15,16,17-dodecahydro-1H-cyclopenta[a]phenanthren-3-ol	Cell division control protein 45 homolog	CDC45
233	MOL000033	(3S,8S,9S,10R,13R,14S,17R)-10,13-dimethyl-17-[(2R,5S)-5-propan-2-yloctan-2-yl]-2,3,4,7,8,9,11,12,14,15,16,17-dodecahydro-1H-cyclopenta[a]phenanthren-3-ol	3-oxo-5-alpha-steroid 4-dehydrogenase 1	SRD5A1
234	MOL000033	(3S,8S,9S,10R,13R,14S,17R)-10,13-dimethyl-17-[(2R,5S)-5-propan-2-yloctan-2-yl]-2,3,4,7,8,9,11,12,14,15,16,17-dodecahydro-1H-cyclopenta[a]phenanthren-3-ol	Ileal sodium/bile acid cotransporter	SLC10A2
235	MOL000033	(3S,8S,9S,10R,13R,14S,17R)-10,13-dimethyl-17-[(2R,5S)-5-propan-2-yloctan-2-yl]-2,3,4,7,8,9,11,12,14,15,16,17-dodecahydro-1H-cyclopenta[a]phenanthren-3-ol	NPC intracellular cholesterol transporter 1	NPC1
236	MOL000033	(3S,8S,9S,10R,13R,14S,17R)-10,13-dimethyl-17-[(2R,5S)-5-propan-2-yloctan-2-yl]-2,3,4,7,8,9,11,12,14,15,16,17-dodecahydro-1H-cyclopenta[a]phenanthren-3-ol	Fibroblast growth factor 2	FGF2
237	MOL000033	(3S,8S,9S,10R,13R,14S,17R)-10,13-dimethyl-17-[(2R,5S)-5-propan-2-yloctan-2-yl]-2,3,4,7,8,9,11,12,14,15,16,17-dodecahydro-1H-cyclopenta[a]phenanthren-3-ol	T-cell surface glycoprotein CD4	CD4
238	MOL000033	(3S,8S,9S,10R,13R,14S,17R)-10,13-dimethyl-17-[(2R,5S)-5-propan-2	CMP-N-acetylneuraminate-beta-galactosamide-alpha-2,3-sialyltrans	ST3GAL1

		-yloctan-2-yl]-2,3,4,7,8,9,11,12,14,15,16,17-dodecahydro-1H-cyclopenta[a]phenanthren-3-ol	ferase 1	
239	MOL000033	(3S,8S,9S,10R,13R,14S,17R)-10,13-dimethyl-17-[(2R,5S)-5-propan-2-yloctan-2-yl]-2,3,4,7,8,9,11,12,14,15,16,17-dodecahydro-1H-cyclopenta[a]phenanthren-3-ol	DNA polymerase alpha catalytic subunit	POLA1
240	MOL000033	(3S,8S,9S,10R,13R,14S,17R)-10,13-dimethyl-17-[(2R,5S)-5-propan-2-yloctan-2-yl]-2,3,4,7,8,9,11,12,14,15,16,17-dodecahydro-1H-cyclopenta[a]phenanthren-3-ol	1,25-dihydroxyvitamin D	CYP24A1
241	MOL000033	(3S,8S,9S,10R,13R,14S,17R)-10,13-dimethyl-17-[(2R,5S)-5-propan-2-yloctan-2-yl]-2,3,4,7,8,9,11,12,14,15,16,17-dodecahydro-1H-cyclopenta[a]phenanthren-3-ol	3-beta-hydroxysteroid-Delta	EBP
242	MOL000033	(3S,8S,9S,10R,13R,14S,17R)-10,13-dimethyl-17-[(2R,5S)-5-propan-2-yloctan-2-yl]-2,3,4,7,8,9,11,12,14,15,16,17-dodecahydro-1H-cyclopenta[a]phenanthren-3-ol	Bile salt export pump	ABCB11
243	MOL000033	(3S,8S,9S,10R,13R,14S,17R)-10,13-dimethyl-17-[(2R,5S)-5-propan-2-yloctan-2-yl]-2,3,4,7,8,9,11,12,14,15,16,17-dodecahydro-1H-cyclopenta[a]phenanthren-3-ol	Ephrin type-A receptor 7	EPHA7
244	MOL000033	(3S,8S,9S,10R,13R,14S,17R)-10,13-dimethyl-17-[(2R,5S)-5-propan-2-yloctan-2-yl]-2,3,4,7,8,9,11,12,14,15,16,17-dodecahydro-1H-cyclopenta[a]phenanthren-3-ol	Ephrin type-A receptor 5	EPHA5
245	MOL000033	(3S,8S,9S,10R,13R,14S,17R)-10,13-dimethyl-17-[(2R,5S)-5-propan-2-yloctan-2-yl]-2,3,4,7,8,9,11,12,14,15,16,17-dodecahydro-1H-cyclopenta[a]phenanthren-3-ol	Ephrin type-A receptor 8	EPHA8
246	MOL000033	(3S,8S,9S,10R,13R,14S,17R)-10,13-dimethyl-17-[(2R,5S)-5-propan-2-yloctan-2-yl]-2,3,4,7,8,9,11,12,14,15,16,17-dodecahydro-1H-cyclopenta[a]phenanthren-3-ol	Ephrin type-B receptor 3	EPHB3
247	MOL000033	(3S,8S,9S,10R,13R,14S,17R)-10,1	Ephrin type-A receptor 4	EPHA4

		3-dimethyl-17-[(2R,5S)-5-propan-2-yloctan-2-yl]-2,3,4,7,8,9,11,12,14,15,16,17-dodecahydro-1H-cyclopenta[a]phenanthren-3-ol		
248	MOL000033	(3S,8S,9S,10R,13R,14S,17R)-10,13-dimethyl-17-[(2R,5S)-5-propan-2-yloctan-2-yl]-2,3,4,7,8,9,11,12,14,15,16,17-dodecahydro-1H-cyclopenta[a]phenanthren-3-ol	Ephrin type-A receptor 1	EPHA1
249	MOL000033	(3S,8S,9S,10R,13R,14S,17R)-10,13-dimethyl-17-[(2R,5S)-5-propan-2-yloctan-2-yl]-2,3,4,7,8,9,11,12,14,15,16,17-dodecahydro-1H-cyclopenta[a]phenanthren-3-ol	Ephrin type-B receptor 1	EPHB1
250	MOL000033	(3S,8S,9S,10R,13R,14S,17R)-10,13-dimethyl-17-[(2R,5S)-5-propan-2-yloctan-2-yl]-2,3,4,7,8,9,11,12,14,15,16,17-dodecahydro-1H-cyclopenta[a]phenanthren-3-ol	Ephrin type-A receptor 6	EPHA6
251	MOL000033	(3S,8S,9S,10R,13R,14S,17R)-10,13-dimethyl-17-[(2R,5S)-5-propan-2-yloctan-2-yl]-2,3,4,7,8,9,11,12,14,15,16,17-dodecahydro-1H-cyclopenta[a]phenanthren-3-ol	Ephrin type-B receptor 2	EPHB2
252	MOL000033	(3S,8S,9S,10R,13R,14S,17R)-10,13-dimethyl-17-[(2R,5S)-5-propan-2-yloctan-2-yl]-2,3,4,7,8,9,11,12,14,15,16,17-dodecahydro-1H-cyclopenta[a]phenanthren-3-ol	Carbonic anhydrase 1	CA1
253	MOL000033	(3S,8S,9S,10R,13R,14S,17R)-10,13-dimethyl-17-[(2R,5S)-5-propan-2-yloctan-2-yl]-2,3,4,7,8,9,11,12,14,15,16,17-dodecahydro-1H-cyclopenta[a]phenanthren-3-ol	Ephrin type-A receptor 2	EPHA2
254	MOL000033	(3S,8S,9S,10R,13R,14S,17R)-10,13-dimethyl-17-[(2R,5S)-5-propan-2-yloctan-2-yl]-2,3,4,7,8,9,11,12,14,15,16,17-dodecahydro-1H-cyclopenta[a]phenanthren-3-ol	Ephrin type-B receptor 6	EPHB6
255	MOL000033	(3S,8S,9S,10R,13R,14S,17R)-10,13-dimethyl-17-[(2R,5S)-5-propan-2-yloctan-2-yl]-2,3,4,7,8,9,11,12,14,15,16,17-dodecahydro-1H-cyclopenta[a]phenanthren-3-ol	25-hydroxyvitamin D-1 alpha hydroxylase, mitochondrial	CYP27B1

256	MOL000033	(3S,8S,9S,10R,13R,14S,17R)-10,13-dimethyl-17-[(2R,5S)-5-propan-2-yloctan-2-yl]-2,3,4,7,8,9,11,12,14,15,16,17-dodecahydro-1H-cyclopenta[a]phenanthren-3-ol	Ectonucleotide pyrophosphatase/phosphodiesterase family member 2	ENPP2
257	MOL000033	(3S,8S,9S,10R,13R,14S,17R)-10,13-dimethyl-17-[(2R,5S)-5-propan-2-yloctan-2-yl]-2,3,4,7,8,9,11,12,14,15,16,17-dodecahydro-1H-cyclopenta[a]phenanthren-3-ol	Ephrin type-A receptor 3	EPHA3
258	MOL000033	(3S,8S,9S,10R,13R,14S,17R)-10,13-dimethyl-17-[(2R,5S)-5-propan-2-yloctan-2-yl]-2,3,4,7,8,9,11,12,14,15,16,17-dodecahydro-1H-cyclopenta[a]phenanthren-3-ol	Zinc finger protein GLI1	GLI1
259	MOL000033	(3S,8S,9S,10R,13R,14S,17R)-10,13-dimethyl-17-[(2R,5S)-5-propan-2-yloctan-2-yl]-2,3,4,7,8,9,11,12,14,15,16,17-dodecahydro-1H-cyclopenta[a]phenanthren-3-ol	Sonic hedgehog protein	SHH
260	MOL000033	(3S,8S,9S,10R,13R,14S,17R)-10,13-dimethyl-17-[(2R,5S)-5-propan-2-yloctan-2-yl]-2,3,4,7,8,9,11,12,14,15,16,17-dodecahydro-1H-cyclopenta[a]phenanthren-3-ol	Solute carrier family 22 member 3	SLC22A3
261	MOL000033	(3S,8S,9S,10R,13R,14S,17R)-10,13-dimethyl-17-[(2R,5S)-5-propan-2-yloctan-2-yl]-2,3,4,7,8,9,11,12,14,15,16,17-dodecahydro-1H-cyclopenta[a]phenanthren-3-ol	Non-lysosomal glucosylceramidase	GBA2
262	MOL000049	3β-acetoxyatractylone	Vesicular acetylcholine transporter	SLC18A3
263	MOL000049	3β-acetoxyatractylone	Histone-binding protein RBBP4	RBBP4
264	MOL000049	3β-acetoxyatractylone	Histone-binding protein RBBP7	RBBP7
265	MOL000049	3β-acetoxyatractylone	Polycomb protein SUZ12	SUZ12
266	MOL000049	3β-acetoxyatractylone	Ubiquitin-conjugating enzyme E2 D3	UBE2D3
267	MOL000072	8β-ethoxy atractylenolide III	Ribosomal protein S6 kinase alpha-5	RPS6KA5
268	MOL000072	8β-ethoxy atractylenolide III	Glycine receptor subunit alpha-2	GLRA2
269	MOL000072	8β-ethoxy atractylenolide III	Tubulin--tyrosine ligase	TTL
270	MOL000072	8β-ethoxy atractylenolide III	Melatonin receptor type 1A	MTNR1A
271	MOL000072	8β-ethoxy atractylenolide III	Melatonin receptor type 1B	MTNR1

				B
272	MOL000072	8β-ethoxy atractylenolide III	TGF-beta receptor type-1	TGFBR1
273	MOL000072	8β-ethoxy atractylenolide III	Collagenase 3	MMP13
274	MOL000072	8β-ethoxy atractylenolide III	Voltage-dependent N-type calcium channel subunit alpha-1B	CACNA 1B
275	MOL000072	8β-ethoxy atractylenolide III	Receptor-interacting serine/threonine-protein kinase 3	RIPK3
276	MOL000072	8β-ethoxy atractylenolide III	Cathepsin B	CTSB
277	MOL000072	8β-ethoxy atractylenolide III	Receptor-interacting serine/threonine-protein kinase 2	RIPK2
278	MOL000072	8β-ethoxy atractylenolide III	Macrophage colony-stimulating factor 1 receptor	CSF1R
279	MOL000072	8β-ethoxy atractylenolide III	Phospholipase A2, membrane associated	PLA2G2 A
280	MOL000072	8β-ethoxy atractylenolide III	Ribosyldihydronicotinamide dehydrogenase	NQO2
281	MOL000072	8β-ethoxy atractylenolide III	Potassium voltage-gated channel subfamily A member 5	KCNA5
282	MOL000098	quercetin	D	DRD4
283	MOL000173	wogonin	Cyclin-O	CCNO
284	MOL000173	wogonin	G1/S-specific cyclin-E2	CCNE2
285	MOL000173	wogonin	Poly	TNKS
286	MOL000173	wogonin	Serine/threonine-protein kinase VRK2	VRK2
287	MOL000173	wogonin	CDK-activating kinase assembly factor MAT1	MNAT1
288	MOL000173	wogonin	Serine/threonine-protein kinase MAK	MAK
289	MOL000173	wogonin	Activin receptor type-2A	ACVR2 A
290	MOL000173	wogonin	Cyclin-K	CCNK
291	MOL000173	wogonin	Cyclin-dependent kinase-like 5	CDKL5
292	MOL000173	wogonin	Calcium/calmodulin-dependent protein kinase type IV	CAMK4
293	MOL000173	wogonin	Cyclin-dependent kinase 13	CDK13
294	MOL000173	wogonin	Casein kinase I isoform alpha-like	CSNK1 A1L
295	MOL000173	wogonin	Serine/threonine-protein kinase MRCK beta	CDC42B PB
296	MOL000173	wogonin	Serine/threonine-protein kinase 32A	STK32A
297	MOL000173	wogonin	Phosphatidylinositol 4-phosphate 5-kinase type-1 gamma	PIP5K1C
298	MOL000173	wogonin	Poly	TNKS2

299	MOL000173	wogonin	Cyclin-dependent kinase 15	CDK15
300	MOL000173	wogonin	Cyclin-dependent kinase 5	CDK5
301	MOL000173	wogonin	Amine oxidase	MAOA
302	MOL000173	wogonin	Activin receptor type-2B	ACVR2 B
303	MOL000173	wogonin	Cyclin-dependent kinase 18	CDK18
304	MOL000173	wogonin	Serine/threonine-protein kinase ICK	CILK1
305	MOL000173	wogonin	Cyclin-dependent kinase 14	CDK14
306	MOL000173	wogonin	Cyclin-dependent kinase 17	CDK17
307	MOL000173	wogonin	Serine/threonine-protein kinase 35	STK35
308	MOL000173	wogonin	Calcium/calmodulin-dependent protein kinase kinase 1	CAMKK 1
309	MOL000173	wogonin	G1/S-specific cyclin-E1	CCNE1
310	MOL000173	wogonin	Cyclin-H	CCNH
311	MOL000173	wogonin	Myotonin-protein kinase	DMPK
312	MOL000173	wogonin	Leukocyte tyrosine kinase receptor	LTK
313	MOL000173	wogonin	Rhodopsin kinase GRK1	GRK1
314	MOL000173	wogonin	Serine/threonine-protein kinase DCLK3	DCLK3
315	MOL000173	wogonin	Mitogen-activated protein kinase 15	MAPK1 5
316	MOL000173	wogonin	NAD	NQO1
317	MOL000173	wogonin	Phosphorylase b kinase gamma catalytic chain, skeletal muscle/heart isoform	PHKG1
318	MOL000173	wogonin	Serine/threonine-protein kinase ULK2	ULK2
319	MOL000173	wogonin	Rhodopsin kinase GRK7	GRK7
320	MOL000173	wogonin	Mitogen-activated protein kinase kinase kinase 3	MAP3K 3
321	MOL000173	wogonin	Prostaglandin G/H synthase 2	PTGS2
322	MOL000173	wogonin	Inhibitor of nuclear factor kappa-B kinase subunit beta	IKBKB
323	MOL000173	wogonin	BDNF/NT-3 growth factors receptor	NTRK2
324	MOL000173	wogonin	Lysine-specific demethylase 4E	KDM4E
325	MOL000173	wogonin	Xanthine dehydrogenase/oxidase	XDH
326	MOL000173	wogonin	Adenosine receptor A2a	ADORA 2A
327	MOL000173	wogonin	ADP-ribosyl cyclase/cyclic ADP-ribose hydrolase 1	CD38
328	MOL000173	wogonin	DNA topoisomerase 1	TOP1

329	MOL000173	wogonin	Arginase-1	ARG1
330	MOL000173	wogonin	Phosphatidylinositol 3-kinase regulatory subunit alpha	PIK3R1
331	MOL000173	wogonin	Carbonic anhydrase 13	CA13
332	MOL000173	wogonin	Calcium/calmodulin-dependent protein kinase type II subunit beta	CAMK2 B
333	MOL000173	wogonin	Aldo-keto reductase family 1 member C2	AKR1C2
334	MOL000173	wogonin	Aldo-keto reductase family 1 member C1	AKR1C1
335	MOL000173	wogonin	Aldo-keto reductase family 1 member C3	AKR1C3
336	MOL000173	wogonin	Aldo-keto reductase family 1 member C4	AKR1C4
337	MOL000173	wogonin	Aldo-keto reductase family 1 member A1	AKR1A1
338	MOL000173	wogonin	C-X-C chemokine receptor type 1	CXCR1
339	MOL000211	Mairin	SUMO-activating enzyme subunit 2	UBA2
340	MOL000211	Mairin	SUMO-activating enzyme subunit 1	SAE1
341	MOL000211	Mairin	CD81 antigen	CD81
342	MOL000211	Mairin	Interleukin-1 beta	IL1B
343	MOL000211	Mairin	1-phosphatidylinositol 4,5-bisphosphate phosphodiesterase gamma-1	PLCG1
344	MOL000239	Jaranol	Anoctamin-1	ANO1
345	MOL000239	Jaranol	Serine/threonine-protein kinase N1	PKN1
346	MOL000273	(2R)-2-[(3S,5R,10S,13R,14R,16R,17R)-3,16-dihydroxy-4,4,10,13,14-pentamethyl-2,3,5,6,12,15,16,17-octahydro-1H-cyclopenta[a]phenanthren-17-yl]-6-methylhept-5-enoic acid	Prostaglandin D2 receptor 2	PTGDR2
347	MOL000273	(2R)-2-[(3S,5R,10S,13R,14R,16R,17R)-3,16-dihydroxy-4,4,10,13,14-pentamethyl-2,3,5,6,12,15,16,17-octahydro-1H-cyclopenta[a]phenanthren-17-yl]-6-methylhept-5-enoic acid	Arachidonate 5-lipoxygenase-activating protein	ALOX5 AP
348	MOL000273	(2R)-2-[(3S,5R,10S,13R,14R,16R,17R)-3,16-dihydroxy-4,4,10,13,14-pentamethyl-2,3,5,6,12,15,16,17-oc	Prostaglandin D2 receptor	PTGDR

		tahydro-1H-cyclopenta[a]phenanthren-17-yl]-6-methylhept-5-enoic acid		
349	MOL000273	(2R)-2-[(3S,5R,10S,13R,14R,16R,17R)-3,16-dihydroxy-4,4,10,13,14-pentamethyl-2,3,5,6,12,15,16,17-octahydro-1H-cyclopenta[a]phenanthren-17-yl]-6-methylhept-5-enoic acid	Polyunsaturated fatty acid lipoxygenase ALOX12	ALOX12
350	MOL000273	(2R)-2-[(3S,5R,10S,13R,14R,16R,17R)-3,16-dihydroxy-4,4,10,13,14-pentamethyl-2,3,5,6,12,15,16,17-octahydro-1H-cyclopenta[a]phenanthren-17-yl]-6-methylhept-5-enoic acid	Thyroid hormone receptor beta	THRB
351	MOL000273	(2R)-2-[(3S,5R,10S,13R,14R,16R,17R)-3,16-dihydroxy-4,4,10,13,14-pentamethyl-2,3,5,6,12,15,16,17-octahydro-1H-cyclopenta[a]phenanthren-17-yl]-6-methylhept-5-enoic acid	Ras guanyl-releasing protein 3	RASGRP3
352	MOL000273	(2R)-2-[(3S,5R,10S,13R,14R,16R,17R)-3,16-dihydroxy-4,4,10,13,14-pentamethyl-2,3,5,6,12,15,16,17-octahydro-1H-cyclopenta[a]phenanthren-17-yl]-6-methylhept-5-enoic acid	Sphingosine 1-phosphate receptor 2	S1PR2
353	MOL000273	(2R)-2-[(3S,5R,10S,13R,14R,16R,17R)-3,16-dihydroxy-4,4,10,13,14-pentamethyl-2,3,5,6,12,15,16,17-octahydro-1H-cyclopenta[a]phenanthren-17-yl]-6-methylhept-5-enoic acid	Prostaglandin F2-alpha receptor	PTGFR
354	MOL000273	(2R)-2-[(3S,5R,10S,13R,14R,16R,17R)-3,16-dihydroxy-4,4,10,13,14-pentamethyl-2,3,5,6,12,15,16,17-octahydro-1H-cyclopenta[a]phenanthren-17-yl]-6-methylhept-5-enoic acid	Delta-type opioid receptor	OPRD1
355	MOL000273	(2R)-2-[(3S,5R,10S,13R,14R,16R,17R)-3,16-dihydroxy-4,4,10,13,14-pentamethyl-2,3,5,6,12,15,16,17-octahydro-1H-cyclopenta[a]phenanthren-17-yl]-6-methylhept-5-enoic	Lysosomal protective protein	CTSA

		acid		
356	MOL000273	(2R)-2-[(3S,5R,10S,13R,14R,16R,17R)-3,16-dihydroxy-4,4,10,13,14-pentamethyl-2,3,5,6,12,15,16,17-octahydro-1H-cyclopenta[a]phenanthren-17-yl]-6-methylhept-5-enoic acid	Endothelin-1 receptor	EDNRA
357	MOL000273	(2R)-2-[(3S,5R,10S,13R,14R,16R,17R)-3,16-dihydroxy-4,4,10,13,14-pentamethyl-2,3,5,6,12,15,16,17-octahydro-1H-cyclopenta[a]phenanthren-17-yl]-6-methylhept-5-enoic acid	Prostaglandin E2 receptor EP3 subtype	PTGER3
358	MOL000273	(2R)-2-[(3S,5R,10S,13R,14R,16R,17R)-3,16-dihydroxy-4,4,10,13,14-pentamethyl-2,3,5,6,12,15,16,17-octahydro-1H-cyclopenta[a]phenanthren-17-yl]-6-methylhept-5-enoic acid	Endothelin receptor type B	EDNRB
359	MOL000273	(2R)-2-[(3S,5R,10S,13R,14R,16R,17R)-3,16-dihydroxy-4,4,10,13,14-pentamethyl-2,3,5,6,12,15,16,17-octahydro-1H-cyclopenta[a]phenanthren-17-yl]-6-methylhept-5-enoic acid	AMP deaminase 2	AMPD2
360	MOL000276	7,9(11)-dehydropachymic acid	Substance-K receptor	TACR2
361	MOL000276	7,9(11)-dehydropachymic acid	Chymotrypsin-C	CTRC
362	MOL000276	7,9(11)-dehydropachymic acid	Peptidyl-prolyl cis-trans isomerase FKBP1A	FKBP1A
363	MOL000276	7,9(11)-dehydropachymic acid	Peptidyl-prolyl cis-trans isomerase FKBP4	FKBP4
364	MOL000276	7,9(11)-dehydropachymic acid	Type-1 angiotensin II receptor	AGTR1
365	MOL000276	7,9(11)-dehydropachymic acid	Signal transducer and activator of transcription 5B	STAT5B
366	MOL000276	7,9(11)-dehydropachymic acid	Extracellular calcium-sensing receptor	CASR
367	MOL000276	7,9(11)-dehydropachymic acid	Ileal sodium/bile acid cotransporter	SLC10A2
368	MOL000279	Cerevisterol	Mitogen-activated protein kinase kinase kinase 20	MAP3K20
369	MOL000279	Cerevisterol	TGF-beta receptor type-2	TGFBR2
370	MOL000279	Cerevisterol	Atypical chemokine receptor 3	ACKR3
371	MOL000279	Cerevisterol	Isocitrate dehydrogenase	IDH1
372	MOL000279	Cerevisterol	Corticotropin-releasing factor	CRHR1

			receptor 1	
373	MOL000279	Cerevisterol	Mitogen-activated protein kinase 13	MAPK13
374	MOL000279	Cerevisterol	cGMP-dependent 3',5'-cyclic phosphodiesterase	PDE2A
375	MOL000279	Cerevisterol	Interleukin-6 receptor subunit beta	IL6ST
376	MOL000279	Cerevisterol	Mast/stem cell growth factor receptor Kit	KIT
377	MOL000279	Cerevisterol	Glutamate receptor 2	GRIA2
378	MOL000279	Cerevisterol	D	DRD2
379	MOL000279	Cerevisterol	Proprotein convertase subtilisin/kexin type 7	PCSK7
380	MOL000279	Cerevisterol	Coagulation factor X	F10
381	MOL000279	Cerevisterol	Phosphatidylinositol 4,5-bisphosphate 3-kinase catalytic subunit alpha isoform	PIK3CA
382	MOL000279	Cerevisterol	Prostaglandin E2 receptor EP4 subtype	PTGER4
383	MOL000280	(2R)-2-[(3S,5R,10S,13R,14R,16R,17R)-3,16-dihydroxy-4,4,10,13,14-pentamethyl-2,3,5,6,12,15,16,17-octahydro-1H-cyclopenta[a]phenanthren-17-yl]-5-isopropyl-hex-5-enoic acid	Thymidylate synthase	TYMS
384	MOL000280	(2R)-2-[(3S,5R,10S,13R,14R,16R,17R)-3,16-dihydroxy-4,4,10,13,14-pentamethyl-2,3,5,6,12,15,16,17-octahydro-1H-cyclopenta[a]phenanthren-17-yl]-5-isopropyl-hex-5-enoic acid	Leucyl-cystinyl aminopeptidase	LNPEP
385	MOL000285	(2R)-2-[(5R,10S,13R,14R,16R,17R)-16-hydroxy-3-keto-4,4,10,13,14-pentamethyl-1,2,5,6,12,15,16,17-octahydrocyclopenta[a]phenanthren-17-yl]-5-isopropyl-hex-5-enoic acid	Prostaglandin E2 receptor EP2 subtype	PTGER2
386	MOL000285	(2R)-2-[(5R,10S,13R,14R,16R,17R)-16-hydroxy-3-keto-4,4,10,13,14-pentamethyl-1,2,5,6,12,15,16,17-octahydrocyclopenta[a]phenanthren-17-yl]-5-isopropyl-hex-5-enoic acid	Neurotensin receptor type 1	NTSR1
387	MOL000285	(2R)-2-[(5R,10S,13R,14R,16R,17R)-16-hydroxy-3-keto-4,4,10,13,14-pentamethyl-1,2,5,6,12,15,16,17-octahydrocyclopenta[a]phenanthren-1	P2Y purinoceptor 12	P2RY12

		7-yl]-5-isopropyl-hex-5-enoic acid		
388	MOL000285	(2R)-2-[(5R,10S,13R,14R,16R,17R)-16-hydroxy-3-keto-4,4,10,13,14-pentamethyl-1,2,5,6,12,15,16,17-octahydrocyclopenta[a]phenanthren-17-yl]-5-isopropyl-hex-5-enoic acid	Integrin alpha-4	ITGA4
389	MOL000287	3beta-Hydroxy-24-methylene-8-lanostene-21-oic acid	17-beta-hydroxysteroid dehydrogenase type 3	HSD17B3
390	MOL000287	3beta-Hydroxy-24-methylene-8-lanostene-21-oic acid	Solute carrier family 22 member 6	SLC22A6
391	MOL000291	Poricoic acid B	Cytochrome P450 26A1	CYP26A1
392	MOL000291	Poricoic acid B	Cytochrome P450 26B1	CYP26B1
393	MOL000292	poricoic acid C	Oxytocin receptor	OXTR
394	MOL000296	hederagenin	Progesterone receptor	PGR
395	MOL000296	hederagenin	Muscarinic acetylcholine receptor M3	CHRM3
396	MOL000296	hederagenin	Muscarinic acetylcholine receptor M1	CHRM1
397	MOL000296	hederagenin	Muscarinic acetylcholine receptor M2	CHRM2
398	MOL000296	hederagenin	Alpha-1B adrenergic receptor	ADRA1B
399	MOL000296	hederagenin	Gamma-aminobutyric acid receptor subunit alpha-1	GABRA1
400	MOL000296	hederagenin	Glutamate receptor 2	GRIA2
401	MOL000296	hederagenin	Nuclear receptor coactivator 2	NCOA2
402	MOL000296	hederagenin	Alcohol dehydrogenase 1C	ADH1C
403	MOL000296	hederagenin	Prostaglandin G/H synthase 1	PTGS1
404	MOL000296	hederagenin	Sodium channel protein type 5 subunit alpha	SCN5A
405	MOL000296	hederagenin	Prostaglandin G/H synthase 2	PTGS2
406	MOL000296	hederagenin	Retinoic acid receptor RXR-alpha	RXRA
407	MOL000296	hederagenin	Sodium-dependent noradrenaline transporter	SLC6A2
408	MOL000296	hederagenin	Aldo-keto reductase family 1 member C2	AKR1C2
409	MOL000296	hederagenin	MAP kinase-activated protein kinase 2	MAPKAPK2
410	MOL000296	hederagenin	Bone morphogenetic protein 2	BMP2
411	MOL000296	hederagenin	Peptidyl-prolyl cis-trans isomerase A	PPIA
412	MOL000296	hederagenin	Steryl-sulfatase	STS

413	MOL000296	hederagenin	Caspase-7	CASP7
414	MOL000296	hederagenin	Thyroid hormone receptor beta	THRB
415	MOL000296	hederagenin	Carbonic anhydrase 2	CA2
416	MOL000296	hederagenin	Kinesin-like protein KIF11	KIF11
417	MOL000296	hederagenin	Mitogen-activated protein kinase 1	MAPK1
418	MOL000296	hederagenin	Apolipoprotein A-II	APOA2
419	MOL000296	hederagenin	Vitamin D-binding protein	GC
420	MOL000296	hederagenin	Nuclear receptor ROR-alpha	RORA
421	MOL000296	hederagenin	SEC14-like protein 2	SEC14L 2
422	MOL000296	hederagenin	Bile acid receptor	NR1H4
423	MOL000296	hederagenin	Tyrosine-protein phosphatase non-receptor type 11	PTPN11
424	MOL000296	hederagenin	Gastrotropin	FABP6
425	MOL000296	hederagenin	TGF-beta receptor type-1	TGFBR1
426	MOL000296	hederagenin	Mitogen-activated protein kinase 14	MAPK1 4
427	MOL000296	hederagenin	Epidermal growth factor receptor	EGFR
428	MOL000296	hederagenin	Mitogen-activated protein kinase 8	MAPK8
429	MOL000296	hederagenin	Vascular endothelial growth factor receptor 2	KDR
430	MOL000296	hederagenin	Proto-oncogene tyrosine-protein kinase Src	SRC
431	MOL000296	hederagenin	Phenylethanolamine N-methyltransferase	PNMT
432	MOL000296	hederagenin	3-phosphoinositide-dependent protein kinase 1	PDPK1
433	MOL000296	hederagenin	Serine/threonine-protein phosphatase 5	PPP5C
434	MOL000296	hederagenin	Heat shock cognate 71 kDa protein	HSPA8
435	MOL000296	hederagenin	Liver carboxylesterase 1	CES1
436	MOL000296	hederagenin	Peroxisome proliferator-activated receptor gamma	PPARG
437	MOL000296	hederagenin	Trafficking protein particle complex subunit 3	TRAPPC 3
438	MOL000296	hederagenin	Prothrombin	F2
439	MOL000296	hederagenin	Histone deacetylase 8	HDAC8
440	MOL000296	hederagenin	Mineralocorticoid receptor	NR3C2
441	MOL000296	hederagenin	Troponin C, slow skeletal and cardiac muscles	TNNC1
442	MOL000296	hederagenin	Aldo-keto reductase family 1	AKR1C1

			member C1	
443	MOL000296	hederagenin	Serine/threonine-protein kinase PLK1	PLK1
444	MOL000296	hederagenin	cAMP-dependent protein kinase catalytic subunit alpha	PRKACA
445	MOL000296	hederagenin	Glutathione S-transferase P	GSTP1
446	MOL000296	hederagenin	Ephrin type-B receptor 4	EPHB4
447	MOL000296	hederagenin	Alpha-tocopherol transfer protein	TTPA
448	MOL000296	hederagenin	Steroid hormone receptor ERR1	ESRRA
449	MOL000296	hederagenin	Dipeptidyl peptidase 4	DPP4
450	MOL000296	hederagenin	Alcohol dehydrogenase class-3	ADH5
451	MOL000296	hederagenin	Retinol-binding protein 4	RBP4
452	MOL000296	hederagenin	Glutathione S-transferase A1	GSTA1
453	MOL000296	hederagenin	Adenosine kinase	ADK
454	MOL000296	hederagenin	Aldo-keto reductase family 1 member C3	AKR1C3
455	MOL000296	hederagenin	Lanosterol synthase	LSS
456	MOL000296	hederagenin	Oxysterols receptor LXR-beta	NR1H2
457	MOL000296	hederagenin	Glycogen synthase kinase-3 beta	GSK3B
458	MOL000296	hederagenin	Cytochrome P450 2C8	CYP2C8
459	MOL000296	hederagenin	Receptor tyrosine-protein kinase erbB-4	ERBB4
460	MOL000296	hederagenin	Flavin reductase	BLVRB
461	MOL000296	hederagenin	Macrophage metalloelastase	MMP12
462	MOL000296	hederagenin	Fibroblast growth factor receptor 2	FGFR2
463	MOL000296	hederagenin	Renin	REN
464	MOL000296	hederagenin	E3 ubiquitin-protein ligase Mdm2	MDM2
465	MOL000296	hederagenin	Ferrochelatase, mitochondrial	FECH
466	MOL000296	hederagenin	Mitogen-activated protein kinase 10	MAPK10
467	MOL000296	hederagenin	Oxysterols receptor LXR-alpha	NR1H3
468	MOL000296	hederagenin	Catenin alpha-1	CTNNA1
469	MOL000296	hederagenin	Tyrosine-protein kinase JAK3	JAK3
470	MOL000296	hederagenin	Tyrosine-protein kinase ITK/TSK	ITK
471	MOL000296	hederagenin	Aldehyde dehydrogenase, mitochondrial	ALDH2
472	MOL000296	hederagenin	Peroxisome proliferator-activated receptor delta	PPARD
473	MOL000296	hederagenin	Nuclear receptor subfamily 1 group I member 3	NR1I3
474	MOL000296	hederagenin	Alpha-1-antitrypsin	SERPINA1

475	MOL000296	hederagenin	Tyrosine-protein kinase SYK	SYK
476	MOL000296	hederagenin	Fatty acid-binding protein, brain	FABP7
477	MOL000296	hederagenin	Fatty acid-binding protein, heart	FABP3
478	MOL000296	hederagenin	72 kDa type IV collagenase	MMP2
479	MOL000296	hederagenin	Dual specificity mitogen-activated protein kinase kinase 1	MAP2K 1
480	MOL000296	hederagenin	Peroxisome proliferator-activated receptor alpha	PPARA
481	MOL000296	hederagenin	Histo-blood group ABO system transferase	ABO
482	MOL000296	hederagenin	Caspase-3	CASP3
483	MOL000296	hederagenin	Ganglioside GM2 activator	GM2A
484	MOL000296	hederagenin	Hepatocyte nuclear factor 4-gamma	HNF4G
485	MOL000296	hederagenin	Retinoic acid receptor gamma	RARG
486	MOL000296	hederagenin	Deoxycytidine kinase	DCK
487	MOL000296	hederagenin	Coagulation factor VII	F7
488	MOL000296	hederagenin	Stromelysin-1	MMP3
489	MOL000296	hederagenin	Peptidyl-prolyl cis-trans isomerase FKBP1A	FKBP1A
490	MOL000296	hederagenin	Protein kinase C theta type	PRKCQ
491	MOL000296	hederagenin	Interleukin-2	IL2
492	MOL000296	hederagenin	Tyrosine-protein kinase ZAP-70	ZAP70
493	MOL000296	hederagenin	Cellular retinoic acid-binding protein 2	CRABP2
494	MOL000296	hederagenin	Glucocorticoid receptor	NR3C1
495	MOL000296	hederagenin	Angiopoietin-1 receptor	TEK
496	MOL000296	hederagenin	Neutrophil gelatinase-associated lipocalin	LCN2
497	MOL000296	hederagenin	Heat shock protein HSP 90-beta	HSP90A B1
498	MOL000296	hederagenin	Histamine N-methyltransferase	HNMT
499	MOL000296	hederagenin	Retinoic acid receptor beta	RARB
500	MOL000296	hederagenin	Retinoic acid receptor alpha	RARA
501	MOL000296	hederagenin	Protein S100-A9	S100A9
502	MOL000296	hederagenin	Vitamin D3 receptor	VDR
503	MOL000296	hederagenin	3-hydroxy-3-methylglutaryl-coenz yme A reductase	HMGCR
504	MOL000296	hederagenin	Phosphatidylinositol 3-kinase regulatory subunit alpha	PIK3R1
505	MOL000296	hederagenin	Retinoic acid receptor RXR-beta	RXRB
506	MOL000296	hederagenin	Growth factor receptor-bound protein 2	GRB2
507	MOL000296	hederagenin	Lactoylglutathione lyase	GLO1

508	MOL000296	hederagenin	Baculoviral IAP repeat-containing protein 7	BIRC7
509	MOL000296	hederagenin	Serine/threonine-protein phosphatase PP1-gamma catalytic subunit	PPP1CC
510	MOL000296	hederagenin	Heme oxygenase 1	HMOX1
511	MOL000296	hederagenin	Phosphatidylcholine transfer protein	PCTP
512	MOL000296	hederagenin	Endothelial protein C receptor	PROCR
513	MOL000300	dehydroeburicoic acid	Glycine amidinotransferase, mitochondrial	GATM
514	MOL000300	dehydroeburicoic acid	Angiotensin-converting enzyme 2	ACE2
515	MOL000354	isorhamnetin	Neutrophil cytosol factor 1	NCF1
516	MOL000354	isorhamnetin	Oxidized low-density lipoprotein receptor 1	OLR1
517	MOL000354	isorhamnetin	Calcium/calmodulin-dependent protein kinase type II subunit beta	CAMK2B
518	MOL000354	isorhamnetin	Calcium/calmodulin-dependent protein kinase type II subunit alpha	CAMK2A
519	MOL000358	beta-sitosterol	Potassium voltage-gated channel subfamily H member 2	KCNH2
520	MOL000358	beta-sitosterol	Muscarinic acetylcholine receptor M4	CHRM4
521	MOL000358	beta-sitosterol	Alpha-1A adrenergic receptor	ADRA1A
522	MOL000358	beta-sitosterol	Beta-2 adrenergic receptor	ADRB2
523	MOL000358	beta-sitosterol	Neuronal acetylcholine receptor subunit alpha-2	CHRNA2
524	MOL000358	beta-sitosterol	Mu-type opioid receptor	OPRM1
525	MOL000358	beta-sitosterol	Apoptosis regulator Bcl-2	BCL2
526	MOL000358	beta-sitosterol	Apoptosis regulator BAX	BAX
527	MOL000358	beta-sitosterol	Caspase-9	CASP9
528	MOL000358	beta-sitosterol	Caspase-8	CASP8
529	MOL000358	beta-sitosterol	Protein kinase C alpha type	PRKCA
530	MOL000358	beta-sitosterol	Serum paraoxonase/arylesterase 1	PON1
531	MOL000358	beta-sitosterol	Microtubule-associated protein 2	MAP2
532	MOL000358	beta-sitosterol	Squalene synthase	FDFT1
533	MOL000358	beta-sitosterol	M-phase inducer phosphatase 2	CDC25B
534	MOL000358	beta-sitosterol	11-beta-hydroxysteroid dehydrogenase type 2	HSD11B2
535	MOL000358	beta-sitosterol	D	DRD2
536	MOL000392	formononetin	Sodium-dependent dopamine transporter	SLC6A3

537	MOL000392	formononetin	Serine/threonine-protein kinase Chk1	CHEK1
538	MOL000392	formononetin	Cyclin-A2	CCNA2
539	MOL000392	formononetin	cAMP-dependent protein kinase inhibitor alpha	PKIA
540	MOL000392	formononetin	Interleukin-4	IL4
541	MOL000392	formononetin	ATP synthase subunit beta, mitochondrial	ATP5F1 B
542	MOL000392	formononetin	NADH-ubiquinone oxidoreductase chain 6	MT-ND6
543	MOL000392	formononetin	3 beta-hydroxysteroid dehydrogenase/Delta 5-->4-isomerase type 2	HSD3B2
544	MOL000392	formononetin	Angiogenin	ANG
545	MOL000392	formononetin	Neutrophil collagenase	MMP8
546	MOL000392	formononetin	Macrophage migration inhibitory factor	MIF
547	MOL000392	formononetin	Glycogen phosphorylase, liver form	PYGL
548	MOL000392	formononetin	Purine nucleoside phosphorylase	PNP
549	MOL000392	formononetin	Estrogen-related receptor gamma	ESRRG
550	MOL000392	formononetin	Hexokinase-1	HK1
551	MOL000392	formononetin	Adenosylhomocysteinase	AHCY
552	MOL000392	formononetin	Lithostathine-1-alpha	REG1A
553	MOL000392	formononetin	Cytochrome P450 2C9	CYP2C9
554	MOL000392	formononetin	Insulin-like growth factor 1 receptor	IGF1R
555	MOL000392	formononetin	Collagenase 3	MMP13
556	MOL000392	formononetin	Protein-arginine deiminase type-4	PADI4
557	MOL000392	formononetin	Coagulation factor X	F10
558	MOL000392	formononetin	T-cell surface glycoprotein CD1a	CD1A
559	MOL000392	formononetin	Uridine-cytidine kinase 2	UCK2
560	MOL000392	formononetin	C-C motif chemokine 5	CCL5
561	MOL000392	formononetin	Thymidylate synthase	TYMS
562	MOL000392	formononetin	Glucose-6-phosphate isomerase	GPI
563	MOL000392	formononetin	Glutathione S-transferase theta-2	GSTT2
564	MOL000392	formononetin	Angiotensin-converting enzyme	ACE
565	MOL000392	formononetin	Dihydrofolate reductase	DHFR
566	MOL000392	formononetin	Chitotriosidase-1	CHIT1
567	MOL000392	formononetin	Methionine aminopeptidase 2	METAP 2
568	MOL000392	formononetin	Leukotriene A-4 hydrolase	LTA4H
569	MOL000392	formononetin	Cathepsin S	CTSS
570	MOL000392	formononetin	Matrix metalloproteinase-9	MMP9

571	MOL000392	formononetin	Hepatocyte growth factor receptor	MET
572	MOL000392	formononetin	Betaine--homocysteine S-methyltransferase 1	BHMT
573	MOL000392	formononetin	Neprilysin	MME
574	MOL000392	formononetin	Coagulation factor XI	F11
575	MOL000392	formononetin	Glutamate carboxypeptidase 2	FOLH1
576	MOL000392	formononetin	Acetyl-CoA acetyltransferase, mitochondrial	ACAT1
577	MOL000392	formononetin	Glutaryl-CoA dehydrogenase, mitochondrial	GCDH
578	MOL000392	formononetin	Eukaryotic translation initiation factor 4E	EIF4E
579	MOL000392	formononetin	Fibrinogen gamma chain	FGG
580	MOL000392	formononetin	Urokinase-type plasminogen activator	PLAU
581	MOL000392	formononetin	Tryptase beta-2	TPSB2
582	MOL000392	formononetin	Ras-related protein Rab-5A	RAB5A
583	MOL000392	formononetin	Riboflavin kinase	RFK
584	MOL000392	formononetin	Carbonic anhydrase 12	CA12
585	MOL000392	formononetin	Adenosine receptor A1	ADORA 1
586	MOL000392	formononetin	Adenosine receptor A2a	ADORA 2A
587	MOL000392	formononetin	Thromboxane-A synthase	TBXAS1
588	MOL000392	formononetin	Amine oxidase	MAOA
589	MOL000392	formononetin	Maltase-glucoamylase	MGAM
590	MOL000392	formononetin	5-hydroxytryptamine receptor 2A	HTR2A
591	MOL000392	formononetin	5-hydroxytryptamine receptor 2C	HTR2C
592	MOL000392	formononetin	Steroid hormone receptor ERR2	ESRRB
593	MOL000392	formononetin	Broad substrate specificity ATP-binding cassette transporter ABCG2	ABCG2
594	MOL000392	formononetin	Carbonic anhydrase 7	CA7
595	MOL000392	formononetin	17-beta-hydroxysteroid dehydrogenase type 2	HSD17B 2
596	MOL000392	formononetin	Carbonic anhydrase 4	CA4
597	MOL000392	formononetin	Carbonyl reductase	CBR1
598	MOL000392	formononetin	Polyunsaturated fatty acid lipoxygenase ALOX12	ALOX12
599	MOL000392	formononetin	Tyrosinase	TYR
600	MOL000392	formononetin	Xanthine dehydrogenase/oxidase	XDH
601	MOL000392	formononetin	6-phosphofructo-2-kinase/fructose -2,6-bisphosphatase 3	PFKFB3
602	MOL000392	formononetin	Receptor-type tyrosine-protein	PTPRS

			phosphatase S	
603	MOL000392	formononetin	17-beta-hydroxysteroid dehydrogenase type 1	HSD17B 1
604	MOL000392	formononetin	ATP-dependent translocase ABCB1	ABCB1
605	MOL000392	formononetin	Polyunsaturated fatty acid lipoxygenase ALOX15	ALOX15
606	MOL000392	formononetin	Toll-like receptor 9	TLR9
607	MOL000392	formononetin	NADPH oxidase 4	NOX4
608	MOL000392	formononetin	Poly	TNKS2
609	MOL000392	formononetin	Carbonic anhydrase 3	CA3
610	MOL000392	formononetin	Carbonic anhydrase 6	CA6
611	MOL000392	formononetin	Carbonic anhydrase 14	CA14
612	MOL000392	formononetin	Carbonic anhydrase 13	CA13
613	MOL000392	formononetin	Carbonic anhydrase 5B, mitochondrial	CA5B
614	MOL000392	formononetin	Carbonic anhydrase 5A, mitochondrial	CA5A
615	MOL000392	formononetin	Dihydroorotate dehydrogenase	DHODH
616	MOL000392	formononetin	DNA excision repair protein ERCC-5	ERCC5
617	MOL000392	formononetin	Flap endonuclease 1	FEN1
618	MOL000392	formononetin	RAF proto-oncogene serine/threonine-protein kinase	RAF1
619	MOL000392	formononetin	Carbonic anhydrase 9	CA9
620	MOL000392	formononetin	Beta-secretase 1	BACE1
621	MOL000392	formononetin	17-beta-hydroxysteroid dehydrogenase type 3	HSD17B 3
622	MOL000392	formononetin	Aldo-keto reductase family 1 member B10	AKR1B1 0
623	MOL000392	formononetin	Cystathionine beta-synthase	CBS
624	MOL000392	formononetin	Aldehyde dehydrogenase X, mitochondrial	ALDH1 B1
625	MOL000392	formononetin	Low molecular weight phosphotyrosine protein phosphatase	ACP1
626	MOL000392	formononetin	Potassium voltage-gated channel subfamily D member 3	KCND3
627	MOL000392	formononetin	DNA-3-methyladenine glycosylase	MPG
628	MOL000392	formononetin	Retinal dehydrogenase 2	ALDH1 A2
629	MOL000392	formononetin	Calmodulin-1	CALM1
630	MOL000392	formononetin	Taste receptor type 2 member 31	TAS2R3

				1
631	MOL000392	formononetin	Kallikrein-7	KLK7
632	MOL000392	formononetin	ELAV-like protein 3	ELAVL3
633	MOL000392	formononetin	Melatonin receptor type 1A	MTNR1A
634	MOL000392	formononetin	Gamma-aminobutyric acid receptor subunit alpha-3	GABRA3
635	MOL000392	formononetin	NAD	NQO1
636	MOL000392	formononetin	Gamma-aminobutyric acid receptor subunit beta-3	GABRB3
637	MOL000392	formononetin	Beta-glucuronidase	GUSB
638	MOL000392	formononetin	Endoplasmic reticulum aminopeptidase 1	ERAP1
639	MOL000392	formononetin	Aldo-keto reductase family 1 member B1	AKR1B1
640	MOL000392	formononetin	Gamma-aminobutyric acid receptor subunit gamma-2	GABRG2
641	MOL000392	formononetin	Monocarboxylate transporter 4	SLC16A3
642	MOL000392	formononetin	Sentrin-specific protease 7	SENP7
643	MOL000392	formononetin	CAAX prenyl protease 2	RCE1
644	MOL000392	formononetin	Melatonin receptor type 1B	MTNR1B
645	MOL000392	formononetin	Cytochrome P450 11B1, mitochondrial	CYP11B1
646	MOL000392	formononetin	Amyloid-beta precursor protein	APP
647	MOL000392	formononetin	Hepatocyte nuclear factor 4-alpha	HNF4A
648	MOL000392	formononetin	DNA topoisomerase 2-beta	TOP2B
649	MOL000392	formononetin	Transthyretin	TTR
650	MOL000392	formononetin	Matrix metalloproteinase-26	MMP26
651	MOL000392	formononetin	Nuclear receptor subfamily 4 group A member 2	NR4A2
652	MOL000392	formononetin	M-phase inducer phosphatase 3	CDC25C
653	MOL000392	formononetin	Chymotrypsin-like elastase family member 1	CELA1
654	MOL000392	formononetin	5'-AMP-activated protein kinase subunit gamma-1	PRKAG1
655	MOL000392	formononetin	Solute carrier family 22 member 12	SLC22A12
656	MOL000392	formononetin	Cytochrome P450 11B2, mitochondrial	CYP11B2
657	MOL000392	formononetin	Potassium voltage-gated channel subfamily A member 3	KCNA3
658	MOL000392	formononetin	Heat shock protein HSP 90-alpha	HSP90A

				A1
659	MOL000392	formononetin	Serine/threonine-protein kinase/endoribonuclease IRE1	ERN1
660	MOL000392	formononetin	Multidrug resistance-associated protein 1	ABCC1
661	MOL000392	formononetin	5'-AMP-activated protein kinase subunit beta-1	PRKAB1
662	MOL000392	formononetin	Polyunsaturated fatty acid 5-lipoxygenase	ALOX5
663	MOL000392	formononetin	Ribosyldihydronicotinamide dehydrogenase	NQO2
664	MOL000392	formononetin	Matrix metalloproteinase-15	MMP15
665	MOL000392	formononetin	Stromal cell-derived factor 1	CXCL12
666	MOL000417	Calycosin	Phenylalanine-4-hydroxylase	PAH
667	MOL000417	Calycosin	Complement factor B	CFB
668	MOL000417	Calycosin	RAC-alpha serine/threonine-protein kinase	AKT1
669	MOL000417	Calycosin	Activated CDC42 kinase 1	TNK2
670	MOL000417	Calycosin	Transforming protein RhoA	RHOA
671	MOL000417	Calycosin	Serine hydroxymethyltransferase, cytosolic	SHMT1
672	MOL000417	Calycosin	Eosinophil cationic protein	RNASE3
673	MOL000417	Calycosin	Triosephosphate isomerase	TPI1
674	MOL000417	Calycosin	Fatty acid-binding protein, adipocyte	FABP4
675	MOL000417	Calycosin	Thymidine kinase, cytosolic	TK1
676	MOL000417	Calycosin	Medium-chain specific acyl-CoA dehydrogenase, mitochondrial	ACADM
677	MOL000417	Calycosin	Dual specificity protein kinase CLK1	CLK1
678	MOL000417	Calycosin	RAC-beta serine/threonine-protein kinase	AKT2
679	MOL000417	Calycosin	Hypoxanthine-guanine phosphoribosyltransferase	HPRT1
680	MOL000417	Calycosin	Hydroxyacyl-coenzyme A dehydrogenase, mitochondrial	HADH
681	MOL000417	Calycosin	Thymidylate kinase	DTYMK
682	MOL000417	Calycosin	Platelet glycoprotein Ib alpha chain	GP1BA
683	MOL000417	Calycosin	Ornithine aminotransferase, mitochondrial	OAT
684	MOL000417	Calycosin	C-type lectin domain family 4 member M	CLEC4 M
685	MOL000417	Calycosin	Glutathione S-transferase A3	GSTA3

686	MOL000417	Calycosin	Glutathione S-transferase Mu 2	GSTM2
687	MOL000417	Calycosin	Pancreatic alpha-amylase	AMY2A
688	MOL000417	Calycosin	Glutathione reductase, mitochondrial	GSR
689	MOL000417	Calycosin	Mitogen-activated protein kinase 12	MAPK12
690	MOL000417	Calycosin	E-selectin	SELE
691	MOL000417	Calycosin	Inositol-trisphosphate 3-kinase A	ITPKA
692	MOL000417	Calycosin	Glutathione S-transferase Mu 1	GSTM1
693	MOL000417	Calycosin	Death-associated protein kinase 1	DAPK1
694	MOL000417	Calycosin	Maleylacetoacetate isomerase	GSTZ1
695	MOL000417	Calycosin	UDP-N-acetylhexosamine pyrophosphorylase	UAP1
696	MOL000417	Calycosin	Quinone oxidoreductase	CRYZ
697	MOL000417	Calycosin	Cytochrome P450 1B1	CYP1B1
698	MOL000417	Calycosin	Tubulin beta-1 chain	TUBB1
699	MOL000417	Calycosin	G1/S-specific cyclin-D3	CCND3
700	MOL000417	Calycosin	Beta-galactoside alpha-2,6-sialyltransferase 1	ST6GAL1
701	MOL000417	Calycosin	G1/S-specific cyclin-D1	CCND1
702	MOL000417	Calycosin	Protein disulfide-isomerase	P4HB
703	MOL000417	Calycosin	Dihydropteridine reductase	QDPR
704	MOL000417	Calycosin	Cytochrome P450 1A1	CYP1A1
705	MOL000417	Calycosin	Tyrosine-protein kinase HCK	HCK
706	MOL000417	Calycosin	Microtubule-associated protein tau	MAPT
707	MOL000417	Calycosin	Serine/threonine-protein kinase Nek6	NEK6
708	MOL000417	Calycosin	ELAV-like protein 1	ELAVL1
709	MOL000422	kaempferol	Aldo-keto reductase family 1 member B1	AKR1B1
710	MOL000422	kaempferol	Steroid hormone receptor ERR1	ESRRA
711	MOL000422	kaempferol	Lactoylglutathione lyase	GLO1
712	MOL000422	kaempferol	Casein kinase II subunit alpha	CSNK2A1
713	MOL000422	kaempferol	Serine/threonine-protein kinase N1	PKN1
714	MOL000422	kaempferol	Alpha-amylase 1A	AMY1A
715	MOL000422	kaempferol	Guanine nucleotide-binding protein G	GNB1
716	MOL000422	kaempferol	Guanine nucleotide-binding protein G	GNG2
717	MOL000449	Stigmasterol	Nuclear receptor coactivator 1	NCOA1
718	MOL000449	Stigmasterol	Alpha-2A adrenergic receptor	ADRA2A

719	MOL000449	Stigmasterol	Chymotrypsinogen B	CTRB1
720	MOL000449	Stigmasterol	Beta-1 adrenergic receptor	ADRB1
721	MOL000449	Stigmasterol	Estradiol 17-beta-dehydrogenase 11	HSD17B 11
722	MOL000449	Stigmasterol	Protein farnesyltransferase/geranylgeranyl transferase type-1 subunit alpha	FNTA
723	MOL000449	Stigmasterol	Placenta growth factor	PGF
724	MOL000449	Stigmasterol	Tyrosine-protein kinase CSK	CSK
725	MOL000449	Stigmasterol	Nuclear receptor subfamily 1 group I member 2	NR1I2
726	MOL000449	Stigmasterol	Phospholipase A2, membrane associated	PLA2G2 A
727	MOL000449	Stigmasterol	Dipeptidase 1	DPEP1
728	MOL000449	Stigmasterol	Protein-glutamine gamma-glutamyltransferase E	TGM3
729	MOL000449	Stigmasterol	Cathepsin B	CTSB
730	MOL000492	(+)-catechin	Hyaluronan synthase 2	HAS2
731	MOL000497	licochalcone a	Retinoblastoma-associated protein	RB1
732	MOL000500	Vestitol	Polyunsaturated fatty acid lipoxygenase ALOX15B	ALOX15 B
733	MOL000500	Vestitol	Serine/threonine-protein kinase pim-3	PIM3
734	MOL000500	Vestitol	MAP kinase-interacting serine/threonine-protein kinase 1	MKNK1
735	MOL000500	Vestitol	Ribosomal protein S6 kinase alpha-2	RPS6KA 2
736	MOL000500	Vestitol	Matrix metalloproteinase-25	MMP25
737	MOL000500	Vestitol	Alkaline phosphatase, germ cell type	ALPG
738	MOL000500	Vestitol	Nuclear receptor subfamily 4immunitygroup A member 1	NR4A1
739	MOL000500	Vestitol	Aldo-keto reductase family 1 member A1	AKR1A1
740	MOL000538	hypaconitine	Muscarinic acetylcholine receptor M3	CHRM3
741	MOL000538	hypaconitine	5-hydroxytryptamine receptor 3A	HTR3A
742	MOL000538	hypaconitine	High affinity cGMP-specific 3',5'-cyclic phosphodiesterase 9A	PDE9A
743	MOL000538	hypaconitine	Dual specificity calcium/calmodulin-dependent 3',5'-cyclic nucleotide phosphodiesterase 1C	PDE1C
744	MOL000538	hypaconitine	NAD	NQO1

745	MOL000538	hypaconitine	High affinity cAMP-specific and IBMX-insensitive 3',5'-cyclic phosphodiesterase 8B	PDE8B
746	MOL000538	hypaconitine	Baculoviral IAP repeat-containing protein 2	BIRC2
747	MOL000785	palmatine	Alpha-2B adrenergic receptor	ADRA2B
748	MOL000785	palmatine	Cytochrome P450 2D6	CYP2D6
749	MOL000785	palmatine	Phosphoglycerate kinase 1	PGK1
750	MOL000785	palmatine	Short transient receptor potential channel 6	TRPC6
751	MOL000785	palmatine	Perforin-1	PRF1
752	MOL000785	palmatine	Serine/threonine-protein kinase MARK1	MARK1
753	MOL000785	palmatine	Tyrosine-protein kinase Lyn	LYN
754	MOL000785	palmatine	Excitatory amino acid transporter 1	SLC1A3
755	MOL000785	palmatine	Macrophage-stimulating protein receptor	MST1R
756	MOL000785	palmatine	Tubulin beta chain	TUBB
757	MOL000785	palmatine	Nuclear receptor subfamily 0 group B member 1	NR0B1
758	MOL000953	CLR	1,25-dihydroxyvitamin D	CYP24A1
759	MOL000953	CLR	Delta	DHCR24
760	MOL000953	CLR	3-beta-hydroxysteroid-Delta	EBP
761	MOL000953	CLR	ATP-binding cassette sub-family C member 4	ABCC4
762	MOL000953	CLR	Hepatic sodium/bile acid cotransporter	SLC10A1
763	MOL000953	CLR	Bifunctional purine biosynthesis protein ATIC	ATIC
764	MOL001323	Sitosterol alpha1	7-dehydrocholesterol reductase	DHCR7
765	MOL001323	Sitosterol alpha1	Nitric oxide synthase, inducible	NOS2
766	MOL001323	Sitosterol alpha1	Oxysterols receptor LXR-beta	NR1H2
767	MOL001323	Sitosterol alpha1	DNA polymerase beta	POLB
768	MOL001323	Sitosterol alpha1	DNA topoisomerase 2-alpha	TOP2A
769	MOL001323	Sitosterol alpha1	Prostaglandin E2 receptor EP1 subtype	PTGER1
770	MOL001323	Sitosterol alpha1	Prostaglandin E2 receptor EP2 subtype	PTGER2
771	MOL001484	Inermine	5-hydroxytryptamine receptor 3A	HTR3A
772	MOL001484	Inermine	Diacylglycerol O-acyltransferase 1	DGAT1

773	MOL001484	Inermine	Tubulin beta-3 chain	TUBB3
774	MOL001484	Inermine	Serine/threonine-protein kinase TBK1	TBK1
775	MOL001494	Mandenol	C5a anaphylatoxin chemotactic receptor 1	C5AR1
776	MOL001494	Mandenol	Glutamate receptor ionotropic, NMDA 2B	GRIN2B
777	MOL001494	Mandenol	Retinoic acid receptor RXR-alpha	RXRA
778	MOL001494	Mandenol	Retinoic acid receptor RXR-gamma	RXRG
779	MOL001494	Mandenol	Neuropeptide Y receptor type 2	NPY2R
780	MOL001494	Mandenol	Sodium- and chloride-dependent glycine transporter 1	SLC6A9
781	MOL001494	Mandenol	Acetyl-CoA carboxylase 1	ACACA
782	MOL001494	Mandenol	Poly	PARP2
783	MOL001494	Mandenol	Platelet-activating factor receptor	PTAFR
784	MOL001494	Mandenol	Alpha-1,6-mannosyl-glycoprotein 2-beta-N-acetylglucosaminyltransf erase	MGAT2
785	MOL001494	Mandenol	Membrane primary amine oxidase	AOC3
786	MOL001494	Mandenol	Retinal rod rhodopsin-sensitive cGMP 3',5'-cyclic phosphodiesterase subunit delta	PDE6D
787	MOL001494	Mandenol	Sodium-dependent neutral amino acid transporter B	SLC6A1 5
788	MOL001494	Mandenol	Cholesteryl ester transfer protein	CETP
789	MOL001494	Mandenol	Mitogen-activated protein kinase kinase kinase 5	MAP3K 5
790	MOL001663	(4aS,6aR,6aS,6bR,8aR,10R,12aR,1 4bS)-10-hydroxy-2,2,6a,6b,9,9,12a -heptamethyl-1,3,4,5,6,6a,7,8,8a,10 ,11,12,13,14b-tetradecahydropicen e-4a-carboxylic acid	Prostaglandin G/H synthase 1	PTGS1
791	MOL001663	(4aS,6aR,6aS,6bR,8aR,10R,12aR,1 4bS)-10-hydroxy-2,2,6a,6b,9,9,12a -heptamethyl-1,3,4,5,6,6a,7,8,8a,10 ,11,12,13,14b-tetradecahydropicen e-4a-carboxylic acid	Prostaglandin E2 receptor EP4 subtype	PTGER4
792	MOL001663	(4aS,6aR,6aS,6bR,8aR,10R,12aR,1 4bS)-10-hydroxy-2,2,6a,6b,9,9,12a -heptamethyl-1,3,4,5,6,6a,7,8,8a,10 ,11,12,13,14b-tetradecahydropicen e-4a-carboxylic acid	Glutamate receptor ionotropic, kainate 1	GRIK1
793	MOL001663	(4aS,6aR,6aS,6bR,8aR,10R,12aR,1	Glutamate receptor ionotropic,	GRIK2

		4bS)-10-hydroxy-2,2,6a,6b,9,9,12a-heptamethyl-1,3,4,5,6,6a,7,8,8a,10,11,12,13,14b-tetradecahydropicene-4a-carboxylic acid	kainate 2	
794	MOL001663	(4aS,6aR,6aS,6bR,8aR,10R,12aR,14bS)-10-hydroxy-2,2,6a,6b,9,9,12a-heptamethyl-1,3,4,5,6,6a,7,8,8a,10,11,12,13,14b-tetradecahydropicene-4a-carboxylic acid	Prostacyclin receptor	PTGIR
795	MOL001663	(4aS,6aR,6aS,6bR,8aR,10R,12aR,14bS)-10-hydroxy-2,2,6a,6b,9,9,12a-heptamethyl-1,3,4,5,6,6a,7,8,8a,10,11,12,13,14b-tetradecahydropicene-4a-carboxylic acid	Bile acid receptor	NR1H4
796	MOL001663	(4aS,6aR,6aS,6bR,8aR,10R,12aR,14bS)-10-hydroxy-2,2,6a,6b,9,9,12a-heptamethyl-1,3,4,5,6,6a,7,8,8a,10,11,12,13,14b-tetradecahydropicene-4a-carboxylic acid	Sodium-dependent dopamine transporter	SLC6A3
797	MOL001663	(4aS,6aR,6aS,6bR,8aR,10R,12aR,14bS)-10-hydroxy-2,2,6a,6b,9,9,12a-heptamethyl-1,3,4,5,6,6a,7,8,8a,10,11,12,13,14b-tetradecahydropicene-4a-carboxylic acid	Liver carboxylesterase 1	CES1
798	MOL001663	(4aS,6aR,6aS,6bR,8aR,10R,12aR,14bS)-10-hydroxy-2,2,6a,6b,9,9,12a-heptamethyl-1,3,4,5,6,6a,7,8,8a,10,11,12,13,14b-tetradecahydropicene-4a-carboxylic acid	Prostaglandin E2 receptor EP1 subtype	PTGER1
799	MOL001792	DFV	17-beta-hydroxysteroid dehydrogenase 14	HSD17B14
800	MOL001792	DFV	Cytochrome P450 3A4	CYP3A4
801	MOL001942	isoimperatorin	Aldehyde dehydrogenase 1A1	ALDH1A1
802	MOL002087	delta4,16-Androstadien-3-one	C-C chemokine receptor type 5	CCR5
803	MOL002259	Physciondiglucoside	Acidic mammalian chitinase	CHIA
804	MOL002259	Physciondiglucoside	Galectin-9	LGALS9
805	MOL002259	Physciondiglucoside	Mitogen-activated protein kinase kinase kinase 9	MAP3K9
806	MOL002259	Physciondiglucoside	Solute carrier family 28 member 3	SLC28A3
807	MOL002259	Physciondiglucoside	Beta-1,4-galactosyltransferase 1	B4GALT1
808	MOL002259	Physciondiglucoside	P2Y purinoceptor 14	P2RY14

809	MOL002259	Physciondiglucoside	Galectin-1	LGALS1
810	MOL002259	Physciondiglucoside	B-cell receptor CD22	CD22
811	MOL002259	Physciondiglucoside	Protein tyrosine phosphatase type IVA 3	PTP4A3
812	MOL002259	Physciondiglucoside	P2Y purinoceptor 6	P2RY6
813	MOL002268	rhein	Alpha-ketoglutarate-dependent dioxygenase FTO	FTO
814	MOL002268	rhein	Adenosine deaminase	ADA
815	MOL002268	rhein	Hypoxia-inducible factor 1-alpha inhibitor	HIF1AN
816	MOL002268	rhein	5'-AMP-activated protein kinase subunit beta-2	PRKAB2
817	MOL002268	rhein	DNA ligase 1	LIG1
818	MOL002268	rhein	Hexokinase HKDC1	HKDC1
819	MOL002268	rhein	Protein S100-B	S100B
820	MOL002268	rhein	Receptor-type tyrosine-protein phosphatase C	PTPRC
821	MOL002268	rhein	Bcl-2-related protein A1	BCL2A1
822	MOL002268	rhein	Histone-lysine N-methyltransferase NSD2	NSD2
823	MOL002268	rhein	NAD-dependent protein deacylase sirtuin-5, mitochondrial	SIRT5
824	MOL002268	rhein	Lysine-specific demethylase 2A	KDM2A
825	MOL002268	rhein	Lysine-specific demethylase 5C	KDM5C
826	MOL002268	rhein	Glyceraldehyde-3-phosphate dehydrogenase	GAPDH
827	MOL002311	Glycyrol	G-protein coupled receptor 84	GPR84
828	MOL002311	Glycyrol	Histone acetyltransferase p300	EP300
829	MOL002372	(6Z,10E,14E,18E)-2,6,10,15,19,23-hexamethyltetracosa-2,6,10,14,18,22-hexaene	Butyrophilin subfamily 3 member A1	BTN3A1
830	MOL002397	karakoline	Plasminogen	PLG
831	MOL002397	karakoline	Chloride intracellular channel protein 1	CLIC1
832	MOL002397	karakoline	Early endosome antigen 1	EEA1
833	MOL002397	karakoline	Phosphoserine phosphatase	PSPH
834	MOL002397	karakoline	A disintegrin and metalloproteinase with thrombospondin motifs 4	ADAMTS4
835	MOL002397	karakoline	ADP-ribosylation factor 4	ARF4
836	MOL002397	karakoline	Glycogen debranching enzyme	AGL
837	MOL002397	karakoline	Tissue alpha-L-fucosidase	FUCA1
838	MOL002397	karakoline	Sigma non-opioid intracellular receptor 1	SIGMAR1

839	MOL002397	karakoline	Dipeptidyl peptidase 2	DPP7
840	MOL002397	karakoline	cGMP-specific 3',5'-cyclic phosphodiesterase	PDE5A
841	MOL002397	karakoline	Dual 3',5'-cyclic-AMP and -GMP phosphodiesterase 11A	PDE11A
842	MOL002397	karakoline	cAMP and cAMP-inhibited cGMP 3',5'-cyclic phosphodiesterase 10A	PDE10A
843	MOL002397	karakoline	Neuronal acetylcholine receptor subunit alpha-4	CHRNA 4
844	MOL002397	karakoline	Beta-2 adrenergic receptor	ADRB2
845	MOL002397	karakoline	Beta-3 adrenergic receptor	ADRB3
846	MOL002397	karakoline	D	DRD1
847	MOL002565	Medicarpin	Huntingtin	HTT
848	MOL002565	Medicarpin	Methyl-CpG-binding domain protein 2	MBD2
849	MOL002643	delta 7-stigmastenol	Potassium-transporting ATPase alpha chain 2	ATP12A
850	MOL002644	Phellopterin	Cyclin-dependent kinase 5 activator 1	CDK5R1
851	MOL002644	Phellopterin	Oxidized purine nucleoside triphosphate hydrolase	NUDT1
852	MOL002644	Phellopterin	Melanin-concentrating hormone receptor 1	MCHR1
853	MOL002644	Phellopterin	Deoxyuridine 5'-triphosphate nucleotidohydrolase, mitochondrial	DUT
854	MOL002644	Phellopterin	High affinity cAMP-specific and IBMX-insensitive 3',5'-cyclic phosphodiesterase 8B	PDE8B
855	MOL002644	Phellopterin	Glycogen	GYS1
856	MOL002644	Phellopterin	Adenosine receptor A2b	ADORA 2B
857	MOL002644	Phellopterin	Trace amine-associated receptor 1	TAAR1
858	MOL002644	Phellopterin	Hydroxycarboxylic acid receptor 2	HCAR2
859	MOL002644	Phellopterin	Heat shock factor protein 1	HSF1
860	MOL002644	Phellopterin	Cytochrome P450 1A2	CYP1A2
861	MOL002644	Phellopterin	Sodium/hydrogen exchanger 1	SLC9A1
862	MOL002644	Phellopterin	N-acylethanolamine-hydrolyzing acid amidase	NAAA
863	MOL002644	Phellopterin	DNA	DNMT3 A
864	MOL002644	Phellopterin	Ribosomal protein S6 kinase beta-1	RPS6KB 1

865	MOL002644	Phellopterin	Potassium voltage-gated channel subfamily A member 5	KCNA5
866	MOL002644	Phellopterin	Platelet-activating factor acetylhydrolase	PLA2G7
867	MOL002644	Phellopterin	ADP-ribosyl cyclase/cyclic ADP-ribose hydrolase 1	CD38
868	MOL002644	Phellopterin	Cell division cycle 7-related protein kinase	CDC7
869	MOL002644	Phellopterin	Histamine H3 receptor	HRH3
870	MOL002644	Phellopterin	Prostaglandin E2 receptor EP3 subtype	PTGER3
871	MOL002644	Phellopterin	Histamine H4 receptor	HRH4
872	MOL002644	Phellopterin	Serine/threonine-protein kinase pim-1	PIM1
873	MOL002644	Phellopterin	Corticotropin-releasing factor receptor 1	CRHR1
874	MOL002644	Phellopterin	cGMP-dependent 3',5'-cyclic phosphodiesterase	PDE2A
875	MOL002644	Phellopterin	Dual specificity tyrosine-phosphorylation-regulate d kinase 1A	DYRK1 A
876	MOL002644	Phellopterin	Dual specificity protein kinase CLK4	CLK4
877	MOL002644	Phellopterin	Potassium voltage-gated channel subfamily A member 2	KCNA2
878	MOL002644	Phellopterin	Potassium voltage-gated channel subfamily A member 1	KCNA1
879	MOL002644	Phellopterin	Potassium voltage-gated channel subfamily A member 4	KCNA4
880	MOL002644	Phellopterin	Potassium voltage-gated channel subfamily A member 7	KCNA7
881	MOL002644	Phellopterin	Potassium voltage-gated channel subfamily A member 6	KCNA6
882	MOL002644	Phellopterin	Exportin-1	XPO1
883	MOL002644	Phellopterin	Small conductance calcium-activated potassium channel protein 1	KCNN1
884	MOL002644	Phellopterin	Small conductance calcium-activated potassium channel protein 2	KCNN2
885	MOL002644	Phellopterin	Nuclear factor NF-kappa-B p105 subunit	NFKB1
886	MOL002714	baicalein	Cellular tumor antigen p53	TP53
887	MOL002714	baicalein	Fos-related antigen 1	FOSL1

888	MOL002714	baicalein	Fos-related antigen 2	FOSL2
889	MOL002714	baicalein	G2/mitotic-specific cyclin-B1	CCNB1
890	MOL002714	baicalein	Myeloperoxidase	MPO
891	MOL002714	baicalein	Insulin-like growth factor II	IGF2
892	MOL002714	baicalein	Cytochrome c	CYCS
893	MOL002714	baicalein	Nuclear factor of activated T-cells, cytoplasmic 1	NFATC1
894	MOL002714	baicalein	Tudor domain-containing protein 7	TDRD7
895	MOL002714	baicalein	NADPH oxidase 5	NOX5
896	MOL002714	baicalein	Apolipoprotein D	APOD
897	MOL002714	baicalein	SPARC	SPARC
898	MOL002714	baicalein	Cyclin-dependent kinase 6	CDK6
899	MOL002714	baicalein	Tyrosine-protein kinase Lck	LCK
900	MOL002714	baicalein	NEDD8-activating enzyme E1 regulatory subunit	NAE1
901	MOL002714	baicalein	Tyrosine-protein kinase Fyn	FYN
902	MOL002714	baicalein	Tyrosine-protein phosphatase non-receptor type 9	PTPN9
903	MOL002714	baicalein	DNA polymerase iota	POLI
904	MOL002714	baicalein	Dopamine beta-hydroxylase	DBH
905	MOL002714	baicalein	L-selectin	SELL
906	MOL002714	baicalein	Proprotein convertase subtilisin/kexin type 7	PCSK7
907	MOL002714	baicalein	Troponin I, cardiac muscle	TNNI3
908	MOL002714	baicalein	Troponin T, cardiac muscle	TNNT2
909	MOL002714	baicalein	Tyrosine-protein phosphatase non-receptor type 22	PTPN22
910	MOL002714	baicalein	Mothers against decapentaplegic homolog 3	SMAD3
911	MOL002714	baicalein	Receptor-type tyrosine-protein phosphatase gamma	PTPRG
912	MOL002714	baicalein	D-amino-acid oxidase	DAO
913	MOL002714	baicalein	Dual specificity protein phosphatase 22	DUSP22
914	MOL002776	Baicalin	Intercellular adhesion molecule 2	ICAM2
915	MOL002776	Baicalin	Galactokinase	GALK1
916	MOL002776	Baicalin	Glutathione synthetase	GSS
917	MOL002776	Baicalin	Solute carrier organic anion transporter family member 1B1	SLCO1B1
918	MOL002882	[(2R)-2,3-dihydroxypropyl] (Z)-octadec-9-enoate	Protein kinase C alpha type	PRKCA
919	MOL002882	[(2R)-2,3-dihydroxypropyl] (Z)-octadec-9-enoate	Protein kinase C delta type	PRKCD

920	MOL002882	[(2R)-2,3-dihydroxypropyl] (Z)-octadec-9-enoate	3-hydroxy-3-methylglutaryl-coenzyme A reductase	HMGCR
921	MOL002882	[(2R)-2,3-dihydroxypropyl] (Z)-octadec-9-enoate	Ectonucleotide pyrophosphatase/phosphodiesterase family member 2	ENPP2
922	MOL002882	[(2R)-2,3-dihydroxypropyl] (Z)-octadec-9-enoate	Glucose-dependent insulinotropic receptor	GPR119
923	MOL002882	[(2R)-2,3-dihydroxypropyl] (Z)-octadec-9-enoate	Lysophosphatidic acid receptor 3	LPAR3
924	MOL002882	[(2R)-2,3-dihydroxypropyl] (Z)-octadec-9-enoate	Lysophosphatidic acid receptor 2	LPAR2
925	MOL002882	[(2R)-2,3-dihydroxypropyl] (Z)-octadec-9-enoate	Lysophosphatidic acid receptor 1	LPAR1
926	MOL002882	[(2R)-2,3-dihydroxypropyl] (Z)-octadec-9-enoate	17-beta-hydroxysteroid dehydrogenase type 2	HSD17B2
927	MOL002882	[(2R)-2,3-dihydroxypropyl] (Z)-octadec-9-enoate	Bcl-2-like protein 1	BCL2L1
928	MOL002882	[(2R)-2,3-dihydroxypropyl] (Z)-octadec-9-enoate	NAD-dependent protein deacetylase sirtuin-2	SIRT2
929	MOL002882	[(2R)-2,3-dihydroxypropyl] (Z)-octadec-9-enoate	Serine protease 1	PRSS1
930	MOL002882	[(2R)-2,3-dihydroxypropyl] (Z)-octadec-9-enoate	Diacylglycerol lipase-alpha	DAGLA
931	MOL002882	[(2R)-2,3-dihydroxypropyl] (Z)-octadec-9-enoate	NADH dehydrogenase	NDUFA1
932	MOL002882	[(2R)-2,3-dihydroxypropyl] (Z)-octadec-9-enoate	NADH dehydrogenase	NDUFA2
933	MOL002882	[(2R)-2,3-dihydroxypropyl] (Z)-octadec-9-enoate	NADH dehydrogenase	NDUFA3
934	MOL002882	[(2R)-2,3-dihydroxypropyl] (Z)-octadec-9-enoate	NADH dehydrogenase	NDUFA5
935	MOL002882	[(2R)-2,3-dihydroxypropyl] (Z)-octadec-9-enoate	NADH dehydrogenase	NDUFA6
936	MOL002882	[(2R)-2,3-dihydroxypropyl] (Z)-octadec-9-enoate	NADH dehydrogenase	NDUFA7
937	MOL002882	[(2R)-2,3-dihydroxypropyl] (Z)-octadec-9-enoate	NADH dehydrogenase	NDUFA8
938	MOL002882	[(2R)-2,3-dihydroxypropyl] (Z)-octadec-9-enoate	NADH dehydrogenase	NDUFA10
939	MOL002882	[(2R)-2,3-dihydroxypropyl] (Z)-octadec-9-enoate	NADH dehydrogenase	NDUFA9
940	MOL002882	[(2R)-2,3-dihydroxypropyl] (Z)-octadec-9-enoate	NADH dehydrogenase	NDUFA11
941	MOL002882	[(2R)-2,3-dihydroxypropyl]	NADH dehydrogenase	NDUFA

		(Z)-octadec-9-enoate		12
942	MOL002882	[(2R)-2,3-dihydroxypropyl] (Z)-octadec-9-enoate	NADH dehydrogenase	NDUFA 13
943	MOL002882	[(2R)-2,3-dihydroxypropyl] (Z)-octadec-9-enoate	NADH dehydrogenase	NDUFB 1
944	MOL002882	[(2R)-2,3-dihydroxypropyl] (Z)-octadec-9-enoate	NADH dehydrogenase	NDUFB 2
945	MOL002882	[(2R)-2,3-dihydroxypropyl] (Z)-octadec-9-enoate	NADH dehydrogenase	NDUFB 3
946	MOL002882	[(2R)-2,3-dihydroxypropyl] (Z)-octadec-9-enoate	NADH dehydrogenase	NDUFB 4
947	MOL002882	[(2R)-2,3-dihydroxypropyl] (Z)-octadec-9-enoate	NADH dehydrogenase	NDUFB 5
948	MOL002882	[(2R)-2,3-dihydroxypropyl] (Z)-octadec-9-enoate	NADH dehydrogenase	NDUFB 7
949	MOL002882	[(2R)-2,3-dihydroxypropyl] (Z)-octadec-9-enoate	NADH dehydrogenase	NDUFB 6
950	MOL002882	[(2R)-2,3-dihydroxypropyl] (Z)-octadec-9-enoate	NADH dehydrogenase	NDUFB 8
951	MOL002882	[(2R)-2,3-dihydroxypropyl] (Z)-octadec-9-enoate	NADH dehydrogenase	NDUFB 10
952	MOL002882	[(2R)-2,3-dihydroxypropyl] (Z)-octadec-9-enoate	NADH dehydrogenase	NDUFB 9
953	MOL002882	[(2R)-2,3-dihydroxypropyl] (Z)-octadec-9-enoate	NADH dehydrogenase	NDUFB 11
954	MOL002882	[(2R)-2,3-dihydroxypropyl] (Z)-octadec-9-enoate	NADH dehydrogenase	NDUFC 1
955	MOL002882	[(2R)-2,3-dihydroxypropyl] (Z)-octadec-9-enoate	NADH dehydrogenase	NDUFA F2
956	MOL002882	[(2R)-2,3-dihydroxypropyl] (Z)-octadec-9-enoate	NADH dehydrogenase	NDUFC 2
957	MOL002882	[(2R)-2,3-dihydroxypropyl] (Z)-octadec-9-enoate	NADH dehydrogenase	NDUFA F4
958	MOL002882	[(2R)-2,3-dihydroxypropyl] (Z)-octadec-9-enoate	NADH dehydrogenase	NDUFA F3
959	MOL002882	[(2R)-2,3-dihydroxypropyl] (Z)-octadec-9-enoate	NADH dehydrogenase	NDUFS2
960	MOL002882	[(2R)-2,3-dihydroxypropyl] (Z)-octadec-9-enoate	NADH dehydrogenase	NDUFS3
961	MOL002882	[(2R)-2,3-dihydroxypropyl] (Z)-octadec-9-enoate	NADH dehydrogenase	NDUFS4
962	MOL002882	[(2R)-2,3-dihydroxypropyl] (Z)-octadec-9-enoate	NADH dehydrogenase	NDUFS5
963	MOL002882	[(2R)-2,3-dihydroxypropyl]	NADH dehydrogenase	NDUFS6

		(Z)-octadec-9-enoate		
964	MOL002882	[(2R)-2,3-dihydroxypropyl] (Z)-octadec-9-enoate	NADH dehydrogenase	NDUFS7
965	MOL002882	[(2R)-2,3-dihydroxypropyl] (Z)-octadec-9-enoate	NADH dehydrogenase	NDUFS8
966	MOL002882	[(2R)-2,3-dihydroxypropyl] (Z)-octadec-9-enoate	NADH dehydrogenase	NDUFV 1
967	MOL002882	[(2R)-2,3-dihydroxypropyl] (Z)-octadec-9-enoate	NADH dehydrogenase	NDUFV 2
968	MOL002882	[(2R)-2,3-dihydroxypropyl] (Z)-octadec-9-enoate	NADH dehydrogenase	NDUFV 3
969	MOL002882	[(2R)-2,3-dihydroxypropyl] (Z)-octadec-9-enoate	NADH dehydrogenase	NDUFA 4L2
970	MOL002882	[(2R)-2,3-dihydroxypropyl] (Z)-octadec-9-enoate	Phospholipase A2 group V	PLA2G5
971	MOL002882	[(2R)-2,3-dihydroxypropyl] (Z)-octadec-9-enoate	Protein phosphatase 1A	PPM1A
972	MOL002882	[(2R)-2,3-dihydroxypropyl] (Z)-octadec-9-enoate	Organic anion transporter 3	SLC22A 8
973	MOL002882	[(2R)-2,3-dihydroxypropyl] (Z)-octadec-9-enoate	Vascular endothelial growth factor A, long form	VEGFA
974	MOL003152	Gentisin	Calcium-activated potassium channel subunit alpha-1	KCNMA 1
975	MOL003152	Gentisin	14-3-3 protein gamma	YWHAG
976	MOL003152	Gentisin	Platelet-derived growth factor receptor alpha	PDGFR A
977	MOL003152	Gentisin	Histone deacetylase 9	HDAC9
978	MOL003152	Gentisin	Low-density lipoprotein receptor-related protein 6	LRP6
979	MOL003152	Gentisin	Intestinal-type alkaline phosphatase	ALPI
980	MOL003152	Gentisin	Transcription factor 4	TCF4
981	MOL003152	Gentisin	Neuromedin-U receptor 2	NMUR2
982	MOL003152	Gentisin	Lysine-specific demethylase 4E	KDM4E
983	MOL003152	Gentisin	Serine/threonine-protein kinase 17B	STK17B
984	MOL003152	Gentisin	Telomerase reverse transcriptase	TERT
985	MOL003152	Gentisin	C-C chemokine receptor type 4	CCR4
986	MOL003152	Gentisin	Casein kinase II subunit beta	CSNK2B
987	MOL003588	Prangenidin	Alpha-2C adrenergic receptor	ADRA2 C
988	MOL003588	Prangenidin	Muscarinic acetylcholine receptor M5	CHRM5
989	MOL003588	Prangenidin	Kappa-type opioid receptor	OPRK1

990	MOL003588	Prangenidin	Ribosomal protein S6 kinase alpha-3	RPS6KA3
991	MOL003588	Prangenidin	cAMP-specific 3',5'-cyclic phosphodiesterase 4D	PDE4D
992	MOL003588	Prangenidin	Heat shock 70 kDa protein 1A	HSPA1A
993	MOL003608	O-Acetylcolumbianetin	Bone morphogenetic protein 7	BMP7
994	MOL003608	O-Acetylcolumbianetin	Adenylate kinase isoenzyme 1	AK1
995	MOL003608	O-Acetylcolumbianetin	Leucine-rich repeat serine/threonine-protein kinase 2	LRRK2
996	MOL003608	O-Acetylcolumbianetin	Kinesin-like protein KIF20A	KIF20A
997	MOL003608	O-Acetylcolumbianetin	ALK tyrosine kinase receptor	ALK
998	MOL003608	O-Acetylcolumbianetin	Transmembrane domain-containing protein TMIGD3	TMIGD3
999	MOL003847	Inophyllum E	Probable G-protein coupled receptor 139	GPR139
1000	MOL003847	Inophyllum E	Endothelial lipase	LIPG
1001	MOL003847	Inophyllum E	Ezrin	EZR
1002	MOL003847	Inophyllum E	Serine/threonine-protein kinase TAO1	TAOK1
1003	MOL003847	Inophyllum E	Eukaryotic translation initiation factor 4H	EIF4H
1004	MOL003896	7-Methoxy-2-methyl isoflavone	Monoglyceride lipase	MGLL
1005	MOL003896	7-Methoxy-2-methyl isoflavone	G-protein coupled receptor 55	GPR55
1006	MOL003896	7-Methoxy-2-methyl isoflavone	Lysyl oxidase homolog 3	LOXL3
1007	MOL003896	7-Methoxy-2-methyl isoflavone	Carboxy-terminal domain RNA polymerase II polypeptide A small phosphatase 1	CTDSP1
1008	MOL003896	7-Methoxy-2-methyl isoflavone	Natural resistance-associated macrophage protein 2	SLC11A2
1009	MOL003896	7-Methoxy-2-methyl isoflavone	Prostaglandin E2 receptor EP4 subtype	PTGER4
1010	MOL004328	naringenin	Dual specificity protein kinase CLK1	CLK1
1011	MOL004328	naringenin	Steroid hormone receptor ERR2	ESRRB
1012	MOL004328	naringenin	Insulin-like growth factor-binding protein 3	IGFBP3
1013	MOL004328	naringenin	Tissue factor	F3
1014	MOL004576	taxifolin	Diacylglycerol O-acyltransferase 2	DGAT2
1015	MOL004576	taxifolin	Microsomal triglyceride transfer protein large subunit	MTTP
1016	MOL004576	taxifolin	Apolipoprotein B-100	APOB
1017	MOL004748	3-deoxyaconitine	Sodium-dependent dopamine	SLC6A3

			transporter	
1018	MOL004748	3-deoxyaconitine	Potassium voltage-gated channel subfamily H member 2	KCNH2
1019	MOL004748	3-deoxyaconitine	Sodium-dependent serotonin transporter	SLC6A4
1020	MOL004748	3-deoxyaconitine	Cholinesterase	BCHE
1021	MOL004748	3-deoxyaconitine	Neuronal acetylcholine receptor subunit alpha-7	CHRNA 7
1022	MOL004748	3-deoxyaconitine	Sodium-dependent noradrenaline transporter	SLC6A2
1023	MOL004748	3-deoxyaconitine	ATP-sensitive inward rectifier potassium channel 1	KCNJ1
1024	MOL004748	3-deoxyaconitine	Beta-secretase 2	BACE2
1025	MOL004748	3-deoxyaconitine	Adenosine receptor A3	ADORA 3
1026	MOL004748	3-deoxyaconitine	Dolichyl-diphosphooligosaccharide--protein glycosyltransferase 48 kDa subunit	DDOST
1027	MOL004748	3-deoxyaconitine	Phosphatidylinositol 4,5-bisphosphate 3-kinase catalytic subunit alpha isoform	PIK3CA
1028	MOL004748	3-deoxyaconitine	5-hydroxytryptamine receptor 2B	HTR2B
1029	MOL004748	3-deoxyaconitine	5-hydroxytryptamine receptor 2C	HTR2C
1030	MOL004748	3-deoxyaconitine	Adenylate cyclase type 1	ADCY1
1031	MOL004748	3-deoxyaconitine	Non-lysosomal glucosylceramidase	GBA2
1032	MOL004748	3-deoxyaconitine	5-hydroxytryptamine receptor 2A	HTR2A
1033	MOL004748	3-deoxyaconitine	Carbonic anhydrase 2	CA2
1034	MOL004748	3-deoxyaconitine	Phosphatidylinositol 4,5-bisphosphate 3-kinase catalytic subunit delta isoform	PIK3CD
1035	MOL004748	3-deoxyaconitine	Phosphatidylinositol 4,5-bisphosphate 3-kinase catalytic subunit beta isoform	PIK3CB
1036	MOL004748	3-deoxyaconitine	Phosphatidylinositol 4,5-bisphosphate 3-kinase catalytic subunit gamma isoform	PIK3CG
1037	MOL004749	3-acetylaconitine	Tyrosine-protein kinase receptor TYRO3	TYRO3
1038	MOL004749	3-acetylaconitine	Copper transport protein ATOX1	ATOX1
1039	MOL004749	3-acetylaconitine	72 kDa type IV collagenase	MMP2
1040	MOL004749	3-acetylaconitine	Macrophage colony-stimulating factor 1 receptor	CSF1R
1041	MOL004749	3-acetylaconitine	Receptor-type tyrosine-protein	FLT3

			kinase FLT3	
1042	MOL004749	3-acetylaconitine	Cyclin-dependent kinase 9	CDK9
1043	MOL004749	3-acetylaconitine	Non-receptor tyrosine-protein kinase TYK2	TYK2
1044	MOL004749	3-acetylaconitine	Ceramide glucosyltransferase	UGCG
1045	MOL004749	3-acetylaconitine	Solute carrier family 2, facilitated glucose transporter member 1	SLC2A1
1046	MOL004749	3-acetylaconitine	Transketolase	TKT
1047	MOL004756	crassicauline A	Peroxiredoxin-2	PRDX2
1048	MOL004756	crassicauline A	Deoxyhypusine synthase	DHPS
1049	MOL004763	Izoteolin	Delta-type opioid receptor	OPRD1
1050	MOL004763	Izoteolin	Alpha-1D adrenergic receptor	ADRA1D
1051	MOL004763	Izoteolin	Matrilysin	MMP7
1052	MOL004763	Izoteolin	Tyrosine-protein phosphatase non-receptor type 1	PTPN1
1053	MOL004763	Izoteolin	Neutrophil collagenase	MMP8
1054	MOL004763	Izoteolin	Platelet-activating factor acetylhydrolase	PLA2G7
1055	MOL004763	Izoteolin	Disintegrin and metalloproteinase domain-containing protein 9	ADAM9
1056	MOL004763	Izoteolin	MAP kinase-interacting serine/threonine-protein kinase 1	MKNK1
1057	MOL004763	Izoteolin	Corticotropin-releasing factor receptor 1	CRHR1
1058	MOL004763	Izoteolin	Histone deacetylase 5	HDAC5
1059	MOL004763	Izoteolin	Histone deacetylase 7	HDAC7
1060	MOL004763	Izoteolin	Histone deacetylase 4	HDAC4
1061	MOL004763	Izoteolin	Ephrin type-B receptor 4	EPHB4
1062	MOL004763	Izoteolin	RAF proto-oncogene serine/threonine-protein kinase	RAF1
1063	MOL004763	Izoteolin	Dual specificity tyrosine-phosphorylation-regulated kinase 1A	DYRK1A
1064	MOL004763	Izoteolin	Matrix metalloproteinase-16	MMP16
1065	MOL004763	Izoteolin	Matrix metalloproteinase-14	MMP14
1066	MOL004763	Izoteolin	Macrophage metalloelastase	MMP12
1067	MOL004763	Izoteolin	Peptide deformylase, mitochondrial	PDF
1068	MOL004763	Izoteolin	Glutamate receptor 1	GRIA1
1069	MOL004763	Izoteolin	D	DRD1
1070	MOL004763	Izoteolin	Tyrosine 3-monooxygenase	TH
1071	MOL004763	Izoteolin	D	DRD2
1072	MOL004763	Izoteolin	Synaptic vesicular amine	SLC18A

			transporter	2
1073	MOL004763	Izoteolin	D	DRD5
1074	MOL004763	Izoteolin	D	DRD3
1075	MOL004763	Izoteolin	Tissue factor	F3
1076	MOL004763	Izoteolin	DNA repair protein RAD52 homolog	RAD52
1077	MOL004763	Izoteolin	5-hydroxytryptamine receptor 2B	HTR2B
1078	MOL004763	Izoteolin	Sigma intracellular receptor 2	TMEM97
1079	MOL004763	Izoteolin	Multidrug and toxin extrusion protein 1	SLC47A1
1080	MOL004763	Izoteolin	Neuronal acetylcholine receptor subunit beta-2	CHRNB2
1081	MOL004763	Izoteolin	Dual specificity calcium/calmodulin-dependent 3',5'-cyclic nucleotide phosphodiesterase 1A	PDE1A
1082	MOL004777	Angelol D	Methionine aminopeptidase 1	METAP1
1083	MOL004777	Angelol D	Serum amyloid P-component	APCS
1084	MOL004777	Angelol D	BAG family molecular chaperone regulator 1	BAG1
1085	MOL004777	Angelol D	Carnitine O-acetyltransferase	CRAT
1086	MOL004777	Angelol D	Serine/threonine-protein kinase PAK 6	PAK6
1087	MOL004777	Angelol D	Arginase-2, mitochondrial	ARG2
1088	MOL004780	Angelicone	Neuronal calcium sensor 1	NCS1
1089	MOL004780	Angelicone	Antigen peptide transporter 1	TAP1
1090	MOL004780	Angelicone	Protein arginine N-methyltransferase 3	PRMT3
1091	MOL004780	Angelicone	Dual specificity protein phosphatase 3	DUSP3
1092	MOL004780	Angelicone	Palmitoleoyl-protein carboxylesterase NOTUM	NOTUM
1093	MOL004780	Angelicone	Cyclin-dependent kinase 4	CDK4
1094	MOL004780	Angelicone	Small conductance calcium-activated potassium channel protein 3	KCNN3
1095	MOL004780	Angelicone	Vascular cell adhesion protein 1	VCAM1
1096	MOL004780	Angelicone	Tumor necrosis factor receptor superfamily member 1A	TNFRSF1A
1097	MOL004780	Angelicone	Potassium voltage-gated channel subfamily B member 1	KCNB1
1098	MOL004780	Angelicone	ERO1-like protein alpha	ERO1A

1099	MOL004780	Angelicone	Anthrax toxin receptor 2	ANTXR 2
1100	MOL004780	Angelicone	Nuclear factor erythroid 2-related factor 2	NFE2L2
1101	MOL004780	Angelicone	NF-kappa-B essential modulator	IKBKG
1102	MOL004782	[(1R,2R)-2,3-dihydroxy-1-(7-methoxy-2-oxochromen-6-yl)-3-methylbutyl] 3-methylbutanoate	Zinc-alpha-2-glycoprotein	AZGP1
1103	MOL004782	[(1R,2R)-2,3-dihydroxy-1-(7-methoxy-2-oxochromen-6-yl)-3-methylbutyl] 3-methylbutanoate	UDP-glucose 4-epimerase	GALE
1104	MOL004782	[(1R,2R)-2,3-dihydroxy-1-(7-methoxy-2-oxochromen-6-yl)-3-methylbutyl] 3-methylbutanoate	Carboxypeptidase B	CPB1
1105	MOL004792	nodakenin	ADP-ribose pyrophosphatase, mitochondrial	NUDT9
1106	MOL004792	nodakenin	TGF-beta receptor type-2	TGFBR2
1107	MOL004806	euchrenone	Proliferating cell nuclear antigen	PCNA
1108	MOL004808	glyasperin B	Bone morphogenetic protein 1	BMP1
1109	MOL004808	glyasperin B	Tyrosine-protein kinase TXK	TXK
1110	MOL004808	glyasperin B	Histone deacetylase 11	HDAC11
1111	MOL004808	glyasperin B	NADP-dependent malic enzyme	ME1
1112	MOL004810	glyasperin F	Serine/threonine-protein kinase Sgk1	SGK1
1113	MOL004811	Glyasperin C	Hematopoietic prostaglandin D synthase	HPGDS
1114	MOL004815	(E)-1-(2,4-dihydroxyphenyl)-3-(2,2-dimethylchromen-6-yl)prop-2-en-1-one	MAP kinase-activated protein kinase 5	MAPKAPK5
1115	MOL004820	kanzonols W	Malate dehydrogenase, mitochondrial	MDH2
1116	MOL004833	Phaseolinisoflavan	Homeodomain-interacting protein kinase 4	HIPK4
1117	MOL004833	Phaseolinisoflavan	Serine/threonine-protein kinase TAO2	TAOK2
1118	MOL004833	Phaseolinisoflavan	Peripheral plasma membrane protein CASK	CASK
1119	MOL004833	Phaseolinisoflavan	Dual serine/threonine and tyrosine protein kinase	DSTYK
1120	MOL004833	Phaseolinisoflavan	Tyrosine-protein kinase Yes	YES1
1121	MOL004833	Phaseolinisoflavan	Dual specificity mitogen-activated protein kinase kinase 3	MAP2K 3
1122	MOL004833	Phaseolinisoflavan	Tyrosine-protein kinase Blk	BLK
1123	MOL004833	Phaseolinisoflavan	Fibroblast growth factor receptor	FGFR3

			3	
1124	MOL004833	Phaseolinisoflavan	cGMP-dependent protein kinase 2	PRKG2
1125	MOL004833	Phaseolinisoflavan	Dual specificity mitogen-activated protein kinase kinase 4	MAP2K 4
1126	MOL004833	Phaseolinisoflavan	Dual specificity mitogen-activated protein kinase kinase 2	MAP2K 2
1127	MOL004833	Phaseolinisoflavan	Interleukin-1 receptor-associated kinase 1	IRAK1
1128	MOL004833	Phaseolinisoflavan	Serine/threonine-protein kinase 16	STK16
1129	MOL004833	Phaseolinisoflavan	Mitogen-activated protein kinase kinase kinase 1	MAP3K 1
1130	MOL004833	Phaseolinisoflavan	Fibroblast growth factor receptor 4	FGFR4
1131	MOL004833	Phaseolinisoflavan	Serine/threonine-protein kinase 10	STK10
1132	MOL004835	Glypallichalcone	Nuclear receptor subfamily 1 group D member 1	NR1D1
1133	MOL004835	Glypallichalcone	Succinate-semialdehyde dehydrogenase, mitochondrial	ALDH5 A1
1134	MOL004835	Glypallichalcone	4-aminobutyrate aminotransferase, mitochondrial	ABAT
1135	MOL004835	Glypallichalcone	G-protein coupled receptor 183	GPR183
1136	MOL004835	Glypallichalcone	Kallikrein-14	KLK14
1137	MOL004835	Glypallichalcone	Protein mono-ADP-ribosyltransferase PARP10	PARP10
1138	MOL004835	Glypallichalcone	NACHT, LRR and PYD domains-containing protein 1	NLRP1
1139	MOL004835	Glypallichalcone	Taste receptor type 2 member 14	TAS2R1 4
1140	MOL004835	Glypallichalcone	3-hydroxyacyl-CoA dehydrogenase type-2	HSD17B 10
1141	MOL004835	Glypallichalcone	Krueppel-like factor 5	KLF5
1142	MOL004835	Glypallichalcone	Serine hydrolase RBBP9	RBBP9
1143	MOL004835	Glypallichalcone	4-hydroxyphenylpyruvate dioxygenase	HPD
1144	MOL004835	Glypallichalcone	Sentrin-specific protease 6	SENP6
1145	MOL004835	Glypallichalcone	Taste receptor type 1 member 3	TAS1R3
1146	MOL004835	Glypallichalcone	Kallikrein-5	KLK5
1147	MOL004835	Glypallichalcone	Sentrin-specific protease 1	SENP1
1148	MOL004835	Glypallichalcone	FAD-linked sulfhydryl oxidase ALR	GFER
1149	MOL004841	Licochalcone B	C-X-C chemokine receptor type 4	CXCR4
1150	MOL004841	Licochalcone B	Zinc finger protein GLI2	GLI2
1151	MOL004856	Gancaonin A	Calcitonin	CALCA

1152	MOL004860	licorice glycoside E	Fibroblast growth factor 1	FGF1
1153	MOL004879	Glycyrin	Lysine-specific demethylase 4B	KDM4B
1154	MOL004879	Glycyrin	Splicing factor 3B subunit 3	SF3B3
1155	MOL004885	licoisoflavanone	Transient receptor potential cation channel subfamily M member 8	TRPM8
1156	MOL004885	licoisoflavanone	Serine-protein kinase ATM	ATM
1157	MOL004885	licoisoflavanone	Sphingosine kinase 2	SPHK2
1158	MOL004903	liquiritin	GTPase NRas	NRAS
1159	MOL004903	liquiritin	Sodium/myo-inositol cotransporter 2	SLC5A11
1160	MOL004903	liquiritin	Tyrosine--tRNA ligase, cytoplasmic	YARS1
1161	MOL004905	3,22-Dihydroxy-11-oxo-delta(12)-oleanene-27-alpha-methoxycarbonyl-29-oic acid	Endothelial PAS domain-containing protein 1	EPAS1
1162	MOL004905	3,22-Dihydroxy-11-oxo-delta(12)-oleanene-27-alpha-methoxycarbonyl-29-oic acid	Vasopressin V1a receptor	AVPR1A
1163	MOL004905	3,22-Dihydroxy-11-oxo-delta(12)-oleanene-27-alpha-methoxycarbonyl-29-oic acid	Lysosomal Pro-X carboxypeptidase	PRCP
1164	MOL004905	3,22-Dihydroxy-11-oxo-delta(12)-oleanene-27-alpha-methoxycarbonyl-29-oic acid	C-X-C chemokine receptor type 3	CXCR3
1165	MOL004905	3,22-Dihydroxy-11-oxo-delta(12)-oleanene-27-alpha-methoxycarbonyl-29-oic acid	Monocarboxylate transporter 1	SLC16A1
1166	MOL004905	3,22-Dihydroxy-11-oxo-delta(12)-oleanene-27-alpha-methoxycarbonyl-29-oic acid	Microtubule-associated serine/threonine-protein kinase 3	MAST3
1167	MOL004905	3,22-Dihydroxy-11-oxo-delta(12)-oleanene-27-alpha-methoxycarbonyl-29-oic acid	Protein Mdm4	MDM4
1168	MOL004908	Glabridin	C-C chemokine receptor type 3	CCR3
1169	MOL004935	Sigmoidin-B	Phospholipase A2	PLA2G1B
1170	MOL004948	Isoglycyrol	Mucosa-associated lymphoid tissue lymphoma translocation protein 1	MALT1
1171	MOL004959	1-Methoxyphaseollidin	Serine/threonine-protein kinase PAK 4	PAK4
1172	MOL004959	1-Methoxyphaseollidin	Serine/threonine-protein kinase TNNI3K	TNNI3K
1173	MOL004961	Quercetin der.	Testis-specific	TSSK2

			serine/threonine-protein kinase 2	
1174	MOL004961	Quercetin der.	PAS domain-containing serine/threonine-protein kinase	PASK
1175	MOL004966	3'-Hydroxy-4'-O-Methylglabridin	Probable global transcription activator SNF2L2	SMARCA2
1176	MOL004966	3'-Hydroxy-4'-O-Methylglabridin	Serine/threonine-protein kinase Nek1	NEK1
1177	MOL004966	3'-Hydroxy-4'-O-Methylglabridin	Ectonucleotide pyrophosphatase/phosphodiesterase family member 1	ENPP1
1178	MOL004966	3'-Hydroxy-4'-O-Methylglabridin	NADPH oxidase 1	NOX1
1179	MOL004966	3'-Hydroxy-4'-O-Methylglabridin	C-C chemokine receptor type 9	CCR9
1180	MOL004974	3'-Methoxyglabridin	Fibrinogen beta chain	FGB
1181	MOL004978	2-[(3R)-8,8-dimethyl-3,4-dihydro-2H-pyrano[6,5-f]chromen-3-yl]-5-methoxyphenol	Cytochrome b-c1 complex subunit 7	UQCRB
1182	MOL004978	2-[(3R)-8,8-dimethyl-3,4-dihydro-2H-pyrano[6,5-f]chromen-3-yl]-5-methoxyphenol	Protein polybromo-1	PBRM1
1183	MOL004978	2-[(3R)-8,8-dimethyl-3,4-dihydro-2H-pyrano[6,5-f]chromen-3-yl]-5-methoxyphenol	Transcription activator BRG1	SMARCA4
1184	MOL004978	2-[(3R)-8,8-dimethyl-3,4-dihydro-2H-pyrano[6,5-f]chromen-3-yl]-5-methoxyphenol	Activin receptor type-1	ACVR1
1185	MOL004980	Inflacoumarin A	Signal transducer and activator of transcription 6	STAT6
1186	MOL004980	Inflacoumarin A	dCTP pyrophosphatase 1	DCTPP1
1187	MOL004985	icos-5-enoic acid	Gastric inhibitory polypeptide receptor	GIPR
1188	MOL004985	icos-5-enoic acid	Gamma-butyrobetaine dioxygenase	BBOX1
1189	MOL004985	icos-5-enoic acid	Gamma-aminobutyric acid receptor subunit theta	GABRQ
1190	MOL004985	icos-5-enoic acid	Cytochrome P450 4F2	CYP4F2
1191	MOL004985	icos-5-enoic acid	Platelet-activating factor acetylhydrolase IB subunit beta	PAFAH1B1
1192	MOL004985	icos-5-enoic acid	Glutathione S-transferase kappa 1	GSTK1
1193	MOL004985	icos-5-enoic acid	Sodium- and chloride-dependent GABA transporter 3	SLC6A11
1194	MOL004985	icos-5-enoic acid	Lysine-specific demethylase 7A	KDM7A
1195	MOL004985	icos-5-enoic acid	Geranylgeranyl pyrophosphate synthase	GGPS1
1196	MOL004985	icos-5-enoic acid	Prostaglandin F2-alpha receptor	PTGFR

1197	MOL004985	icos-5-enoic acid	Prostaglandin D2 receptor	PTGDR
1198	MOL004985	icos-5-enoic acid	Farnesyl pyrophosphate synthase	FDPS
1199	MOL004985	icos-5-enoic acid	Sphingosine 1-phosphate receptor 2	S1PR2
1200	MOL004985	icos-5-enoic acid	Glutamyl aminopeptidase	ENPEP
1201	MOL004985	icos-5-enoic acid	Leukotriene B4 receptor 1	LTB4R
1202	MOL004985	icos-5-enoic acid	Gamma-aminobutyric acid type B receptor subunit 2	GABBR 2
1203	MOL004985	icos-5-enoic acid	Gamma-aminobutyric acid type B receptor subunit 1	GABBR 1
1204	MOL004985	icos-5-enoic acid	Histone lysine demethylase PHF8	PHF8
1205	MOL004985	icos-5-enoic acid	Gamma-aminobutyric acid receptor subunit rho-1	GABRR 1
1206	MOL004985	icos-5-enoic acid	Sphingosine 1-phosphate receptor 3	S1PR3
1207	MOL004985	icos-5-enoic acid	Sphingosine 1-phosphate receptor 4	S1PR4
1208	MOL004985	icos-5-enoic acid	Free fatty acid receptor 4	FFAR4
1209	MOL004991	7-Acetoxy-2-methylisoflavone	Prokineticin receptor 1	PROKR1
1210	MOL004991	7-Acetoxy-2-methylisoflavone	Ubiquitin carboxyl-terminal hydrolase 4	USP4
1211	MOL004991	7-Acetoxy-2-methylisoflavone	Ubiquitin carboxyl-terminal hydrolase 5	USP5
1212	MOL004991	7-Acetoxy-2-methylisoflavone	Dual specificity protein phosphatase CDC14A	CDC14A
1213	MOL005003	Licoagrocarpin	Proto-oncogene tyrosine-protein kinase ROS	ROS1
1214	MOL005003	Licoagrocarpin	Disintegrin and metalloproteinase domain-containing protein 33	ADAM3 3
1215	MOL005016	Odoratin	Bromodomain-containing protein 9	BRD9
1216	MOL005020	dehydroglyasperins C	Gonadotropin-releasing hormone receptor	GNRHR
1217	MOL005100	5,7-dihydroxy-2-(3-hydroxy-4-met hoxyphenyl)chroman-4-one	Carbonic anhydrase 7	CA7
1218	MOL005100	5,7-dihydroxy-2-(3-hydroxy-4-met hoxyphenyl)chroman-4-one	Carbonic anhydrase 4	CA4
1219	MOL005100	5,7-dihydroxy-2-(3-hydroxy-4-met hoxyphenyl)chroman-4-one	Broad substrate specificity ATP-binding cassette transporter ABCG2	ABCG2
1220	MOL005100	5,7-dihydroxy-2-(3-hydroxy-4-met hoxyphenyl)chroman-4-one	17-beta-hydroxysteroid dehydrogenase type 1	HSD17B 1
1221	MOL005100	5,7-dihydroxy-2-(3-hydroxy-4-met hoxyphenyl)chroman-4-one	Amine oxidase	MAOB

1222	MOL005100	5,7-dihydroxy-2-(3-hydroxy-4-methoxyphenyl)chroman-4-one	Carbonyl reductase	CBR1
1223	MOL005100	5,7-dihydroxy-2-(3-hydroxy-4-methoxyphenyl)chroman-4-one	Kallikrein-1	KLK1
1224	MOL005100	5,7-dihydroxy-2-(3-hydroxy-4-methoxyphenyl)chroman-4-one	Kallikrein-2	KLK2
1225	MOL005100	5,7-dihydroxy-2-(3-hydroxy-4-methoxyphenyl)chroman-4-one	Carbonic anhydrase 3	CA3
1226	MOL005100	5,7-dihydroxy-2-(3-hydroxy-4-methoxyphenyl)chroman-4-one	Carbonic anhydrase 6	CA6
1227	MOL005100	5,7-dihydroxy-2-(3-hydroxy-4-methoxyphenyl)chroman-4-one	Carbonic anhydrase 5A, mitochondrial	CA5A
1228	MOL005100	5,7-dihydroxy-2-(3-hydroxy-4-methoxyphenyl)chroman-4-one	Plasminogen activator inhibitor 1	SERPINE1
1229	MOL005100	5,7-dihydroxy-2-(3-hydroxy-4-methoxyphenyl)chroman-4-one	Group 10 secretory phospholipase A2	PLA2G10
1230	MOL005100	5,7-dihydroxy-2-(3-hydroxy-4-methoxyphenyl)chroman-4-one	Carbonic anhydrase 13	CA13
1231	MOL005100	5,7-dihydroxy-2-(3-hydroxy-4-methoxyphenyl)chroman-4-one	Carbonic anhydrase 5B, mitochondrial	CA5B
1232	MOL005100	5,7-dihydroxy-2-(3-hydroxy-4-methoxyphenyl)chroman-4-one	Retinoic acid receptor RXR-alpha	RXRA
1233	MOL005100	5,7-dihydroxy-2-(3-hydroxy-4-methoxyphenyl)chroman-4-one	Metabotropic glutamate receptor 2	GRM2
1234	MOL005100	5,7-dihydroxy-2-(3-hydroxy-4-methoxyphenyl)chroman-4-one	DNA	DNMT1
1235	MOL005100	5,7-dihydroxy-2-(3-hydroxy-4-methoxyphenyl)chroman-4-one	6-phosphogluconate dehydrogenase, decarboxylating	PGD
1236	MOL005100	5,7-dihydroxy-2-(3-hydroxy-4-methoxyphenyl)chroman-4-one	Alpha-	FUT4
1237	MOL005100	5,7-dihydroxy-2-(3-hydroxy-4-methoxyphenyl)chroman-4-one	Squalene monooxygenase	SQLE
1238	MOL005100	5,7-dihydroxy-2-(3-hydroxy-4-methoxyphenyl)chroman-4-one	Serine/threonine-protein kinase pim-1	PIM1
1239	MOL005100	5,7-dihydroxy-2-(3-hydroxy-4-methoxyphenyl)chroman-4-one	Serine/threonine-protein kinase pim-2	PIM2
1240	MOL005100	5,7-dihydroxy-2-(3-hydroxy-4-methoxyphenyl)chroman-4-one	Serine/threonine-protein kinase pim-3	PIM3
1241	MOL005100	5,7-dihydroxy-2-(3-hydroxy-4-methoxyphenyl)chroman-4-one	Matrix metalloproteinase-9	MMP9
1242	MOL005100	5,7-dihydroxy-2-(3-hydroxy-4-methoxyphenyl)chroman-4-one	Poly	PARP1
1243	MOL005100	5,7-dihydroxy-2-(3-hydroxy-4-methoxyphenyl)chroman-4-one	Poly	TNKS2

1244	MOL005100	5,7-dihydroxy-2-(3-hydroxy-4-methoxyphenyl)chroman-4-one	Poly	TNKS
1245	MOL005100	5,7-dihydroxy-2-(3-hydroxy-4-methoxyphenyl)chroman-4-one	Protein arginine N-methyltransferase 1	PRMT1
1246	MOL005100	5,7-dihydroxy-2-(3-hydroxy-4-methoxyphenyl)chroman-4-one	Endothelin-1 receptor	EDNRA
1247	MOL005100	5,7-dihydroxy-2-(3-hydroxy-4-methoxyphenyl)chroman-4-one	Amine oxidase	MAOB
1248	MOL005100	5,7-dihydroxy-2-(3-hydroxy-4-methoxyphenyl)chroman-4-one	Alpha-	FUT4
1249	MOL005100	5,7-dihydroxy-2-(3-hydroxy-4-methoxyphenyl)chroman-4-one	Polyunsaturated fatty acid lipoxygenase ALOX12	ALOX12
1250	MOL005100	5,7-dihydroxy-2-(3-hydroxy-4-methoxyphenyl)chroman-4-one	ATP-binding cassette sub-family C member 2	ABCC2
1251	MOL005594	ClematosideA'_qt	Nucleoside diphosphate kinase B	NME2
1252	MOL005598	Embinin	Cellular tumor antigen p53	TP53
1253	MOL005598	Embinin	Alpha-amylase 1A	AMY1A
1254	MOL005598	Embinin	Probable glucose sensor protein SLC5A4	SLC5A4
1255	MOL005603	Heptyl phthalate	Androgen receptor	AR
1256	MOL005603	Heptyl phthalate	Potassium channel subfamily K member 2	KCNK2
1257	MOL005603	Heptyl phthalate	5-hydroxytryptamine receptor 4	HTR4
1258	MOL005603	Heptyl phthalate	Acrosin	ACR
1259	MOL005603	Heptyl phthalate	Cyclic AMP-dependent transcription factor ATF-1	ATF1
1260	MOL005603	Heptyl phthalate	Voltage-dependent L-type calcium channel subunit alpha-1D	CACNA1D
1261	MOL005603	Heptyl phthalate	Calpain-2 catalytic subunit	CAPN2
1262	MOL005603	Heptyl phthalate	Monocyte differentiation antigen CD14	CD14
1263	MOL005603	Heptyl phthalate	Peptide deformylase, mitochondrial	PDF
1264	MOL005603	Heptyl phthalate	Alpha-	FUT7
1265	MOL005603	Heptyl phthalate	Gamma-aminobutyric acid receptor subunit alpha-6	GABRA6
1266	MOL005603	Heptyl phthalate	Gamma-aminobutyric acid receptor subunit beta-1	GABRB1
1267	MOL005603	Heptyl phthalate	Gamma-aminobutyric acid receptor subunit beta-2	GABRB2
1268	MOL005603	Heptyl phthalate	Metabotropic glutamate receptor 6	GRM6
1269	MOL005603	Heptyl phthalate	Metabotropic glutamate receptor 7	GRM7
1270	MOL005603	Heptyl phthalate	Metabotropic glutamate receptor 8	GRM8
1271	MOL005603	Heptyl phthalate	RAS guanyl-releasing protein 1	RASGR

				P1
1272	MOL005603	Heptyl phthalate	Histamine H2 receptor	HRH2
1273	MOL005603	Heptyl phthalate	Protein-S-isoprenylcysteine O-methyltransferase	ICMT
1274	MOL005603	Heptyl phthalate	Polyunsaturated fatty acid lipoxygenase ALOX15	ALOX15
1275	MOL005603	Heptyl phthalate	Neutral cholesterol ester hydrolase 1	NCEH1
1276	MOL005603	Heptyl phthalate	Group IID secretory phospholipase A2	PLA2G2D
1277	MOL005603	Heptyl phthalate	Myeloblastin	PRTN3
1278	MOL005603	Heptyl phthalate	Sphingosine 1-phosphate receptor 5	S1PR5
1279	MOL005603	Heptyl phthalate	Sphingosine 1-phosphate receptor 1	S1PR1
1280	MOL005603	Heptyl phthalate	Long-chain fatty acid transport protein 4	SLC27A4
1281	MOL005603	Heptyl phthalate	Multidrug and toxin extrusion protein 2	SLC47A2
1282	MOL005603	Heptyl phthalate	Diamine acetyltransferase 1	SAT1
1283	MOL005603	Heptyl phthalate	Sodium channel protein type 8 subunit alpha	SCN8A
1284	MOL005603	Heptyl phthalate	Steroidogenic factor 1	NR5A1
1285	MOL005603	Heptyl phthalate	Toll-like receptor 8	TLR8
1286	MOL005603	Heptyl phthalate	Transient receptor potential cation channel subfamily M member 2	TRPM2
1287	MOL005603	Heptyl phthalate	Ubiquitin-like domain-containing CTD phosphatase 1	UBLCP1
1288	MOL005603	Heptyl phthalate	DNA repair protein complementing XP-A cells	XPA
1289	MOL005656	Glycozolidal	Angiopoietin-1 receptor	TEK
1290	MOL005656	Glycozolidal	N-acylethanolamine-hydrolyzing acid amidase	NAAA
1291	MOL005656	Glycozolidal	Cell division cycle 7-related protein kinase	CDC7
1292	MOL005656	Glycozolidal	Phosphatidylinositol 4-kinase beta	PI4KB
1293	MOL005656	Glycozolidal	Sodium channel protein type 4 subunit alpha	SCN4A
1294	MOL005656	Glycozolidal	Ribosomal protein S6 kinase alpha-3	RPS6KA3
1295	MOL005656	Glycozolidal	Sodium channel protein type 9 subunit alpha	SCN9A
1296	MOL005656	Glycozolidal	Proteasome subunit beta type-5	PSMB5
1297	MOL005656	Glycozolidal	Intercellular adhesion molecule 1	ICAM1

1298	MOL005656	Glycozolidal	E-selectin	SELE
1299	MOL005656	Glycozolidal	2-Hydroxyacid oxidase 2	HAO2
1300	MOL005656	Glycozolidal	Neprilysin	MME
1301	MOL005656	Glycozolidal	cAMP-dependent protein kinase catalytic subunit alpha	PRKAC A
1302	MOL005656	Glycozolidal	Mitogen-activated protein kinase 9	MAPK9
1303	MOL005656	Glycozolidal	Serine/threonine-protein phosphatase PP1-alpha catalytic subunit	PPP1CA
1304	MOL005656	Glycozolidal	Trace amine-associated receptor 1	TAAR1
1305	MOL005656	Glycozolidal	NAD-dependent protein deacetylase sirtuin-1	SIRT1
1306	MOL005656	Glycozolidal	15-hydroxyprostaglandin dehydrogenase	HPGD
1307	MOL005656	Glycozolidal	Ribosomal protein S6 kinase alpha-2	RPS6KA 2
1308	MOL005656	Glycozolidal	Protein mono-ADP-ribosyltransferase PARP10	PARP10
1309	MOL005656	Glycozolidal	MAP kinase-interacting serine/threonine-protein kinase 2	MKNK2
1310	MOL005656	Glycozolidal	Queuine tRNA-ribosyltransferase catalytic subunit 1	QTRT1
1311	MOL005656	Glycozolidal	Sodium channel protein type 10 subunit alpha	SCN10A
1312	MOL005656	Glycozolidal	Acid ceramidase	ASAH1
1313	MOL005656	Glycozolidal	D	DRD5
1314	MOL005656	Glycozolidal	RecQ-like DNA helicase BLM	BLM
1315	MOL005656	Glycozolidal	5-hydroxytryptamine receptor 3A	HTR3A
1316	MOL005656	Glycozolidal	Beta-adrenergic receptor kinase 2	GRK3
1317	MOL005656	Glycozolidal	B2 bradykinin receptor	BDKRB 2
1318	MOL005656	Glycozolidal	Urotensin-2 receptor	UTS2R
1319	MOL005656	Glycozolidal	Beta-adrenergic receptor kinase 1	GRK2
1320	MOL005656	Glycozolidal	G protein-coupled receptor kinase 5	GRK5
1321	MOL005656	Glycozolidal	5-hydroxytryptamine receptor 1E	HTR1E
1322	MOL005656	Glycozolidal	Serine/threonine-protein kinase haspin	HASPIN
1323	MOL005656	Glycozolidal	Ribosyldihydronicotinamide dehydrogenase	NQO2
1324	MOL005658	Periplogenin	Sodium/potassium-transporting ATPase subunit alpha-1	ATP1A1

1325	MOL005658	Periplogenin	Mitogen-activated protein kinase 10	MAPK10
1326	MOL005658	Periplogenin	Proteinase-activated receptor 2	F2RL1
1327	MOL005658	Periplogenin	Mitogen-activated protein kinase 11	MAPK11
1328	MOL005658	Periplogenin	Biotin--protein ligase	HLCS
1329	MOL005658	Periplogenin	Retinol-binding protein 4	RBP4
1330	MOL005658	Periplogenin	Nuclear receptor subfamily 1 group I member 2	NR1I2
1331	MOL005658	Periplogenin	Dipeptidyl peptidase 4	DPP4
1332	MOL005658	Periplogenin	Proto-oncogene tyrosine-protein kinase receptor Ret	RET
1333	MOL005658	Periplogenin	Sodium/potassium-transporting ATPase subunit alpha-1	ATP1A1
1334	MOL005658	Periplogenin	Neurogenic locus notch homolog protein 1	NOTCH1
1335	MOL005658	Periplogenin	Paired box protein Pax-8	PAX8
1336	MOL005658	Periplogenin	Solute carrier organic anion transporter family member 4C1	SLCO4C1
1337	MOL005675	periplocoside E_qt	ADP-ribosylation factor 1	ARF1
1338	MOL005683	delta 5-Pregnenetriol	Lanosterol 14-alpha demethylase	CYP51A1
1339	MOL005683	delta 5-Pregnenetriol	DNA polymerase alpha catalytic subunit	POLA1
1340	MOL005683	delta 5-Pregnenetriol	Serine/threonine-protein kinase MRCK alpha	CDC42BPA
1341	MOL005683	delta 5-Pregnenetriol	Cyclin-dependent kinase 8	CDK8
1342	MOL005683	delta 5-Pregnenetriol	Wee1-like protein kinase	WEE1
1343	MOL005692	NERIDIENONE A	Indoleamine 2,3-dioxygenase 1	IDO1
1344	MOL005692	NERIDIENONE A	2-Hydroxyacid oxidase 1	HAO1
1345	MOL005692	NERIDIENONE A	Protein-S-isoprenylcysteine O-methyltransferase	ICMT
1346	MOL005692	NERIDIENONE A	Stromelysin-1	MMP3
1347	MOL005692	NERIDIENONE A	Cytochrome P450 11B1, mitochondrial	CYP11B1
1348	MOL005692	NERIDIENONE A	Cytochrome P450 11B2, mitochondrial	CYP11B2
1349	MOL005692	NERIDIENONE A	D	DRD4
1350	MOL005692	NERIDIENONE A	3-oxo-5-alpha-steroid 4-dehydrogenase 2	SRD5A2
1351	MOL005692	NERIDIENONE A	Hormone-sensitive lipase	LIPE
1352	MOL005692	NERIDIENONE A	Delta-type opioid receptor	OPRD1
1353	MOL005692	NERIDIENONE A	Kappa-type opioid receptor	OPRK1
1354	MOL005692	NERIDIENONE A	CREB-binding protein	CREBBP

1355	MOL005692	NERIDIENONE A	Leukotriene A-4 hydrolase	LTA4H
1356	MOL005692	NERIDIENONE A	Heme oxygenase 1	HMOX1
1357	MOL005692	NERIDIENONE A	Squalene synthase	FDFT1
1358	MOL005692	NERIDIENONE A	Carbonic anhydrase 14	CA14
1359	MOL005692	NERIDIENONE A	RAS guanyl-releasing protein 1	RASGRP1
1360	MOL005692	NERIDIENONE A	ATP-binding cassette sub-family C member 9	ABCC9
1361	MOL005692	NERIDIENONE A	D	DRD3
1362	MOL005692	NERIDIENONE A	Hypoxia-inducible factor 1-alpha	HIF1A
1363	MOL005692	NERIDIENONE A	Solute carrier family 22 member 1	SLC22A1
1364	MOL005815	Citromitin	Adenosine receptor A1	ADORA1
1365	MOL005815	Citromitin	Acyl-CoA	FADS1
1366	MOL005815	Citromitin	Acyl-protein thioesterase 1	LYPLA1
1367	MOL005815	Citromitin	Acyl-protein thioesterase 2	LYPLA2
1368	MOL005828	nobiletin	Squalene synthase	FDFT1
1369	MOL005828	nobiletin	D	DRD2
1370	MOL007514	methyl icosa-11,14-dienoate	Integrin alpha-L	ITGAL
1371	MOL007514	methyl icosa-11,14-dienoate	Stearoyl-CoA desaturase	SCD
1372	MOL007514	methyl icosa-11,14-dienoate	Fatty acid-binding protein 5	FABP5
1373	MOL007514	methyl icosa-11,14-dienoate	Fatty acid-binding protein, liver	FABP1
1374	MOL007514	methyl icosa-11,14-dienoate	Oxytocin receptor	OXTR
1375	MOL007514	methyl icosa-11,14-dienoate	Induced myeloid leukemia cell differentiation protein Mcl-1	MCL1
1376	MOL007514	methyl icosa-11,14-dienoate	Proteinase-activated receptor 1	F2R
1377	MOL007514	methyl icosa-11,14-dienoate	Sterol O-acyltransferase 1	SOAT1
1378	MOL007514	methyl icosa-11,14-dienoate	Somatostatin receptor type 4	SSTR4
1379	MOL007514	methyl icosa-11,14-dienoate	Calcitonin gene-related peptide type 1 receptor	CALCRL
1380	MOL007514	methyl icosa-11,14-dienoate	DNA topoisomerase 2-alpha	TOP2A
1381	MOL007514	methyl icosa-11,14-dienoate	Platelet-derived growth factor receptor beta	PDGFRB
1382	MOL007514	methyl icosa-11,14-dienoate	15-hydroxyprostaglandin dehydrogenase	HPGD
1383	MOL007514	methyl icosa-11,14-dienoate	Aurora kinase B	AURKB
1384	MOL007514	methyl icosa-11,14-dienoate	Platelet-activating factor receptor	PTAFR
1385	MOL007514	methyl icosa-11,14-dienoate	Rho-associated protein kinase 2	ROCK2
1386	MOL007514	methyl icosa-11,14-dienoate	Acetyl-CoA carboxylase 2	ACACB
1387	MOL007514	methyl icosa-11,14-dienoate	Arachidonate 5-lipoxygenase-activating protein	ALOX5AP
1388	MOL007514	methyl icosa-11,14-dienoate	Receptor-type tyrosine-protein phosphatase F	PTPRF

1389	MOL007514	methyl icosa-11,14-dienoate	Epoxide hydrolase 1	EPHX1
1390	MOL007514	methyl icosa-11,14-dienoate	Rho-associated protein kinase 1	ROCK1
1391	MOL007514	methyl icosa-11,14-dienoate	Protein smoothened	SMO
1392	MOL007514	methyl icosa-11,14-dienoate	Tyrosine-protein phosphatase non-receptor type 2	PTPN2
1393	MOL007514	methyl icosa-11,14-dienoate	Nicotinamide phosphoribosyltransferase	NAMPT
1394	MOL007514	methyl icosa-11,14-dienoate	Peptidyl-prolyl cis-trans isomerase D	PPID
1395	MOL007514	methyl icosa-11,14-dienoate	C5a anaphylatoxin chemotactic receptor 1	C5AR1
1396	MOL007514	methyl icosa-11,14-dienoate	Sphingosine-1-phosphate lyase 1	SGPL1
1397	MOL007514	methyl icosa-11,14-dienoate	Tyrosine-protein kinase Mer	MERTK
1398	MOL007514	methyl icosa-11,14-dienoate	Acid ceramidase	ASAH1
1399	MOL007514	methyl icosa-11,14-dienoate	Lysophosphatidic acid receptor 3	LPAR3
1400	MOL007514	methyl icosa-11,14-dienoate	Putative P2Y purinoceptor 10	P2RY10
1401	MOL007514	methyl icosa-11,14-dienoate	Lysophosphatidic acid receptor 4	LPAR4
1402	MOL007514	methyl icosa-11,14-dienoate	Toll-like receptor 2	TLR2
1403	MOL007514	methyl icosa-11,14-dienoate	Probable G-protein coupled receptor 174	GPR174
1404	MOL007514	methyl icosa-11,14-dienoate	Lysophosphatidic acid receptor 6	LPAR6
1405	MOL007514	methyl icosa-11,14-dienoate	Lysophosphatidic acid receptor 1	LPAR1
1406	MOL007514	methyl icosa-11,14-dienoate	Probable G-protein coupled receptor 34	GPR34
1407	MOL007514	methyl icosa-11,14-dienoate	Oxoeicosanoid receptor 1	OXER1
1408	MOL007514	methyl icosa-11,14-dienoate	Lysophosphatidic acid receptor 2	LPAR2
1409	MOL007514	methyl icosa-11,14-dienoate	DNA-directed DNA/RNA polymerase mu	POLM
1410	MOL007514	methyl icosa-11,14-dienoate	Mitochondrial carnitine/acylcarnitine carrier protein	SLC25A20
1411	MOL007514	methyl icosa-11,14-dienoate	DNA polymerase eta	POLH
1412	MOL007514	methyl icosa-11,14-dienoate	Alkaline ceramidase 2	ACER2
1413	MOL007514	methyl icosa-11,14-dienoate	Serine palmitoyltransferase 2	SPTLC2
1414	MOL007514	methyl icosa-11,14-dienoate	Putative inactive group IIC secretory phospholipase A2	PLA2G2C
1415	MOL007514	methyl icosa-11,14-dienoate	Ceramide transfer protein	CERT1
1416	MOL007514	methyl icosa-11,14-dienoate	All-trans-retinol dehydrogenase	ADH7
1417	MOL007514	methyl icosa-11,14-dienoate	Platelet-activating factor acetylhydrolase IB subunit alpha2	PAFAH1B2
1418	MOL007514	methyl icosa-11,14-dienoate	Serine palmitoyltransferase 1	SPTLC1
1419	MOL007514	methyl icosa-11,14-dienoate	DNA polymerase kappa	POLK
1420	MOL007514	methyl icosa-11,14-dienoate	Phospholipase A2 group V	PLA2G5
1421	MOL007514	methyl icosa-11,14-dienoate	Peptidyl-glycine alpha-amidating	PAM

			monooxygenase	
1422	MOL007514	methyl icosa-11,14-dienoate	Programmed cell death protein 4	PDCD4
1423	MOL007514	methyl icosa-11,14-dienoate	Macrophage-expressed gene 1 protein	MPEG1
1424	MOL007514	methyl icosa-11,14-dienoate	Sphingomyelin phosphodiesterase 2	SMPD2
1425	MOL007514	methyl icosa-11,14-dienoate	Histone acetyltransferase KAT5	KAT5
1426	MOL007514	methyl icosa-11,14-dienoate	Bifunctional epoxide hydrolase 2	EPHX2
1427	MOL007514	methyl icosa-11,14-dienoate	Acyl carrier protein, mitochondrial	NDUFA B1
1428	MOL007514	methyl icosa-11,14-dienoate	Complex I intermediate-associated protein 30, mitochondrial	NDUFA F1
1429	MOL007514	methyl icosa-11,14-dienoate	NADH dehydrogenase	NDUFA 1
1430	MOL007514	methyl icosa-11,14-dienoate	NADH-ubiquinone oxidoreductase 75 kDa subunit, mitochondrial	NDUFS1
1431	MOL007514	methyl icosa-11,14-dienoate	NADH-ubiquinone oxidoreductase chain 1	MT-ND1
1432	MOL007514	methyl icosa-11,14-dienoate	NADH-ubiquinone oxidoreductase chain 2	MT-ND2
1433	MOL007514	methyl icosa-11,14-dienoate	NADH-ubiquinone oxidoreductase chain 3	MT-ND3
1434	MOL007514	methyl icosa-11,14-dienoate	NADH-ubiquinone oxidoreductase chain 4L	MT-ND4 L
1435	MOL007514	methyl icosa-11,14-dienoate	NADH-ubiquinone oxidoreductase chain 5	MT-ND5
1436	MOL007514	methyl icosa-11,14-dienoate	Solute carrier organic anion transporter family member 2A1	SLCO2A 1
1437	MOL007514	methyl icosa-11,14-dienoate	Lymphocyte antigen 96	LY96
1438	MOL007514	methyl icosa-11,14-dienoate	Cytochrome c oxidase subunit NDUFA4	NDUFA 4
1439	MOL007514	methyl icosa-11,14-dienoate	NADH-ubiquinone oxidoreductase chain 4	MT-ND4
1440	MOL007514	methyl icosa-11,14-dienoate	Isoleucine--tRNA ligase, cytoplasmic	IARS1
1441	MOL007514	methyl icosa-11,14-dienoate	N-arachidonyl glycine receptor	GPR18
1442	MOL007514	methyl icosa-11,14-dienoate	Dynamin-1	DNM1
1443	MOL007514	methyl icosa-11,14-dienoate	DNA polymerase lambda	POLL
1444	MOL007514	methyl icosa-11,14-dienoate	Diacylglycerol lipase-alpha	DAGLA
1445	MOL007514	methyl icosa-11,14-dienoate	Nucleotide-binding oligomerization domain-containing protein 1	NOD1
1446	MOL007514	methyl icosa-11,14-dienoate	Histone acetyltransferase KAT2B	KAT2B

1447	MOL007514	methyl icosa-11,14-dienoate	Cytosolic phospholipase A2 beta	PLA2G4B
1448	MOL007514	methyl icosa-11,14-dienoate	Protein kinase C epsilon type	PRKCE
1449	MOL007514	methyl icosa-11,14-dienoate	Lysine-specific demethylase 5A	KDM5A
1450	MOL007514	methyl icosa-11,14-dienoate	Organic anion transporter 3	SLC22A8
1451	MOL007514	methyl icosa-11,14-dienoate	Toll-like receptor 4	TLR4
1452	MOL007514	methyl icosa-11,14-dienoate	Tyrosine-protein phosphatase non-receptor type 13	PTPN13
1453	MOL007514	methyl icosa-11,14-dienoate	DNA-	APEX1
1454	MOL007514	methyl icosa-11,14-dienoate	Solute carrier family 22 member 6	SLC22A6
1455	MOL007514	methyl icosa-11,14-dienoate	Sphingosine kinase 1	SPHK1
1456	MOL007514	methyl icosa-11,14-dienoate	Lysophosphatidic acid receptor 5	LPAR5
1457	MOL007514	methyl icosa-11,14-dienoate	Alcohol dehydrogenase 1A	ADH1A
1458	MOL008118	Coixenolide	Cannabinoid receptor 2	CNR2
1459	MOL008118	Coixenolide	Protein kinase C gamma type	PRKCG
1460	MOL008118	Coixenolide	Protein kinase C eta type	PRKCH
1461	MOL008118	Coixenolide	All-trans-retinol dehydrogenase	ADH1B
1462	MOL008118	Coixenolide	DNA-	APEX1
1463	MOL008118	Coixenolide	17-beta-hydroxysteroid dehydrogenase type 3	HSD17B3
1464	MOL008121	2-Monoolein	Aldo-keto reductase family 1 member C3	AKR1C3
1465	MOL008188	Japonine	Rho-related GTP-binding protein RhoE	RND3
1466	MOL008188	Japonine	Sterol 26-hydroxylase, mitochondrial	CYP27A1
1467	MOL008188	Japonine	Peregrin	BRPF1
1468	MOL008188	Japonine	Bromodomain-containing protein 1	BRD1
1469	MOL008188	Japonine	Transcription intermediary factor 1-alpha	TRIM24
1470	MOL008188	Japonine	Kynurenine--oxoglutarate transaminase 3	KYAT3
1471	MOL008188	Japonine	Cytochrome c oxidase subunit 2	MT-CO2
1472	MOL008188	Japonine	Serine/threonine-protein kinase WNK1	WNK1
1473	MOL008188	Japonine	Bromodomain and PHD finger-containing protein 3	BRPF3
1474	MOL008188	Japonine	Low affinity immunoglobulin epsilon Fc receptor	FCER2
1475	MOL008188	Japonine	Glutamate receptor ionotropic, kainate 2	GRIK2

1476	MOL008188	Japonine	Sphingomyelin phosphodiesterase	SMPD1
1477	MOL008188	Japonine	Cytosolic phospholipase A2	PLA2G4A
1478	MOL008188	Japonine	Neuropeptides B/W receptor type 1	NPBWR1
1479	MOL008188	Japonine	Interleukin-8	CXCL8
1480	MOL008188	Japonine	1-phosphatidylinositol 4,5-bisphosphate phosphodiesterase gamma-2	PLCG2
1481	MOL008188	Japonine	NACHT, LRR and PYD domains-containing protein 3	NLRP3
1482	MOL008188	Japonine	Aryl hydrocarbon receptor	AHR
1483	MOL008188	Japonine	NUAK family SNF1-like kinase 1	NUAK1
1484	MOL008188	Japonine	Taste receptor type 1 member 1	TAS1R1
1485	MOL008188	Japonine	Catechol O-methyltransferase	COMT
1486	MOL008188	Japonine	Nuclear receptor coactivator 3	NCOA3
1487	MOL008188	Japonine	Bone morphogenetic protein 4	BMP4
1488	MOL009312	(E,E)-3,5-Di-O-caffeoylquinic acid	Reticulon-4 receptor	RTN4R
1489	MOL009312	(E,E)-3,5-Di-O-caffeoylquinic acid	Peroxiredoxin-5, mitochondrial	PRDX5
1490	MOL009312	(E,E)-3,5-Di-O-caffeoylquinic acid	Immunoglobulin alpha Fc receptor	FCAR
1491	MOL009312	(E,E)-3,5-Di-O-caffeoylquinic acid	Nuclear receptor subfamily 0 group B member 2	NR0B2
1492	MOL009312	(E,E)-3,5-Di-O-caffeoylquinic acid	Myocilin	MYOC
1493	MOL009312	(E,E)-3,5-Di-O-caffeoylquinic acid	Glucose-6-phosphate exchanger SLC37A4	SLC37A4
1494	MOL009312	(E,E)-3,5-Di-O-caffeoylquinic acid	Aldo-keto reductase family 1 member C4	AKR1C4
1495	MOL009312	(E,E)-3,5-Di-O-caffeoylquinic acid	10 kDa heat shock protein, mitochondrial	HSPE1
1496	MOL009312	(E,E)-3,5-Di-O-caffeoylquinic acid	60 kDa heat shock protein, mitochondrial	HSPD1
1497	MOL009322	Sylvestroside III	Bactericidal permeability-increasing protein	BPI
1498	MOL011730	11-hydroxy-sec-o-beta-d-glucosylhamaudol_qt	Group 10 secretory phospholipase A2	PLA2G10
1499	MOL011730	11-hydroxy-sec-o-beta-d-glucosylhamaudol_qt	Triggering receptor expressed on myeloid cells 1	TREM1
1500	MOL011730	11-hydroxy-sec-o-beta-d-glucosylhamaudol_qt	Glutaminyl-peptide cyclotransferase	QPCT
1501	MOL011730	11-hydroxy-sec-o-beta-d-glucosylhamaudol_qt	Complement factor D	CFD
1502	MOL011730	11-hydroxy-sec-o-beta-d-glucosylhamaudol_qt	Branched-chain-amino-acid aminotransferase, mitochondrial	BCAT2
1503	MOL011730	11-hydroxy-sec-o-beta-d-glucosylh	Complement C1r subcomponent	C1R

		amaudol_qt		
1504	MOL011730	11-hydroxy-sec-o-beta-d-glucosylh amaudol_qt	Sorbitol dehydrogenase	SORD
1505	MOL011730	11-hydroxy-sec-o-beta-d-glucosylh amaudol_qt	Interferon-stimulated gene 20 kDa protein	ISG20
1506	MOL011730	11-hydroxy-sec-o-beta-d-glucosylh amaudol_qt	Serine protease hepsin	HPN
1507	MOL011730	11-hydroxy-sec-o-beta-d-glucosylh amaudol_qt	Cyclin-T1	CCNT1
1508	MOL011730	11-hydroxy-sec-o-beta-d-glucosylh amaudol_qt	Cathepsin F	CTSF
1509	MOL011730	11-hydroxy-sec-o-beta-d-glucosylh amaudol_qt	Arylsulfatase A	ARSA
1510	MOL011730	11-hydroxy-sec-o-beta-d-glucosylh amaudol_qt	Cathepsin G	CTSG
1511	MOL011730	11-hydroxy-sec-o-beta-d-glucosylh amaudol_qt	Beta-hexosaminidase subunit beta	HEXB
1512	MOL011730	11-hydroxy-sec-o-beta-d-glucosylh amaudol_qt	Thyroid hormone receptor alpha	THRA
1513	MOL011730	11-hydroxy-sec-o-beta-d-glucosylh amaudol_qt	C-1-tetrahydrofolate synthase, cytoplasmic	MTHFD 1
1514	MOL011730	11-hydroxy-sec-o-beta-d-glucosylh amaudol_qt	Lysozyme C	LYZ
1515	MOL011730	11-hydroxy-sec-o-beta-d-glucosylh amaudol_qt	Cathepsin K	CTSK
1516	MOL011730	11-hydroxy-sec-o-beta-d-glucosylh amaudol_qt	Insulin receptor	INSR
1517	MOL011730	11-hydroxy-sec-o-beta-d-glucosylh amaudol_qt	P-selectin	SELP
1518	MOL011730	11-hydroxy-sec-o-beta-d-glucosylh amaudol_qt	Tyrosine-protein kinase JAK2	JAK2
1519	MOL011730	11-hydroxy-sec-o-beta-d-glucosylh amaudol_qt	Spermidine synthase	SRM
1520	MOL011730	11-hydroxy-sec-o-beta-d-glucosylh amaudol_qt	Matrix metalloproteinase-16	MMP16
1521	MOL011730	11-hydroxy-sec-o-beta-d-glucosylh amaudol_qt	Caspase-1	CASP1
1522	MOL011730	11-hydroxy-sec-o-beta-d-glucosylh amaudol_qt	Peptidyl-prolyl cis-trans isomerase FKBP1B	FKBP1B
1523	MOL011730	11-hydroxy-sec-o-beta-d-glucosylh amaudol_qt	Bcl-2-like protein 1	BCL2L1
1524	MOL011732	anomalin	Cathepsin D	CTSD
1525	MOL011732	anomalin	Annexin A5	ANXA5
1526	MOL011732	anomalin	Galectin-7	LGALS7

1527	MOL011732	anomalin	Cytidine deaminase	CDA
1528	MOL011732	anomalin	Prostatic acid phosphatase	ACP3
1529	MOL011732	anomalin	Endoplasmic reticulum mannosyl-oligosaccharide 1,2-alpha-mannosidase	MAN1B 1
1530	MOL011732	anomalin	Galectin-3	LGALS3
1531	MOL011732	anomalin	CD209 antigen	CD209
1532	MOL011732	anomalin	Cannabinoid receptor 1	CNR1
1533	MOL011732	anomalin	Neutrophil elastase	ELANE
1534	MOL011732	anomalin	Cannabinoid receptor 2	CNR2
1535	MOL011732	anomalin	Macrophage colony-stimulating factor 1 receptor	CSF1R
1536	MOL011732	anomalin	Sodium channel protein type 2 subunit alpha	SCN2A
1537	MOL011732	anomalin	Orexin receptor type 2	HCRTR2
1538	MOL011732	anomalin	Sodium channel protein type 10 subunit alpha	SCN10A
1539	MOL011732	anomalin	P2X purinoceptor 3	P2RX3
1540	MOL011732	anomalin	Sodium channel protein type 9 subunit alpha	SCN9A
1541	MOL011732	anomalin	Procathepsin L	CTSL
1542	MOL011732	anomalin	Elongation of very long chain fatty acids protein 6	ELOVL6
1543	MOL011732	anomalin	Metabotropic glutamate receptor 5	GRM5
1544	MOL011732	anomalin	Translocator protein	TSPO
1545	MOL011732	anomalin	P2X purinoceptor 7	P2RX7
1546	MOL011732	anomalin	Potassium channel subfamily K member 3	KCNK3
1547	MOL011732	anomalin	Potassium channel subfamily K member 9	KCNK9
1548	MOL011732	anomalin	Transitional endoplasmic reticulum ATPase	VCP
1549	MOL011732	anomalin	Isocitrate dehydrogenase	IDH1
1550	MOL011732	anomalin	B1 bradykinin receptor	BDKRB 1
1551	MOL011732	anomalin	Neuropeptide Y receptor type 5	NPY5R
1552	MOL011732	anomalin	5-hydroxytryptamine receptor 1A	HTR1A
1553	MOL011732	anomalin	5-hydroxytryptamine receptor 7	HTR7
1554	MOL011732	anomalin	5-hydroxytryptamine receptor 6	HTR6
1555	MOL011732	anomalin	Mitogen-activated protein kinase 11	MAPK1 1
1556	MOL011732	anomalin	Orexin/Hypocretin receptor type 1	HCRTR1
1557	MOL011732	anomalin	Neuromedin-K receptor	TACR3
1558	MOL011732	anomalin	Transient receptor potential cation	TRPA1

			channel subfamily A member 1	
1559	MOL011732	anomalin	Protein-glutamine gamma-glutamyltransferase 2	TGM2
1560	MOL011732	anomalin	Protein-glutamine gamma-glutamyltransferase K	TGM1
1561	MOL011732	anomalin	Chymase	CMA1
1562	MOL011732	anomalin	Coagulation factor XIII A chain	F13A1
1563	MOL011732	anomalin	Polyadenylate-binding protein 1	PABPC1
1564	MOL011732	anomalin	Carnitine O-palmitoyltransferase 1, liver isoform	CPT1A
1565	MOL011732	anomalin	Inhibitor of nuclear factor kappa-B kinase subunit beta	IKBKB
1566	MOL011732	anomalin	Fatty-acid amide hydrolase 1	FAAH
1567	MOL011732	anomalin	Metabotropic glutamate receptor 4	GRM4
1568	MOL011732	anomalin	High affinity cAMP-specific 3',5'-cyclic phosphodiesterase 7A	PDE7A
1569	MOL011732	anomalin	Nitric oxide synthase 1	NOS1
1570	MOL011732	anomalin	cAMP and cAMP-inhibited cGMP 3',5'-cyclic phosphodiesterase 10A	PDE10A
1571	MOL011732	anomalin	Transient receptor potential cation channel subfamily V member 1	TRPV1
1572	MOL011732	anomalin	Protein kinase C gamma type	PRKCG
1573	MOL011732	anomalin	Proteasome subunit beta type-5	PSMB5
1574	MOL011732	anomalin	Hexokinase-4	GCK
1575	MOL011732	anomalin	Focal adhesion kinase 1	PTK2
1576	MOL011732	anomalin	Gastrin/cholecystokinin type B receptor	CCKBR
1577	MOL011732	anomalin	Metabotropic glutamate receptor 2	GRM2
1578	MOL011732	anomalin	Receptor tyrosine-protein kinase erbB-2	ERBB2
1579	MOL011737	divaricatacid	Probable ATP-dependent RNA helicase DDX6	DDX6
1580	MOL011737	divaricatacid	U1 small nuclear ribonucleoprotein A	SNRPA
1581	MOL011737	divaricatacid	Galactosylgalactosylxylosylprotein 3-beta-glucuronosyltransferase 1	B3GAT1
1582	MOL011737	divaricatacid	Kynurenine--oxoglutarate transaminase 1	KYAT1
1583	MOL011737	divaricatacid	S-adenosylmethionine decarboxylase proenzyme	AMD1
1584	MOL011737	divaricatacid	NAD-dependent malic enzyme, mitochondrial	ME2
1585	MOL011737	divaricatacid	Casein kinase I isoform gamma-2	CSNK1G2

1586	MOL011737	divaricatacid	L-lactate dehydrogenase B chain	LDHB
1587	MOL011737	divaricatacid	Ras-related C3 botulinum toxin substrate 2	RAC2
1588	MOL011737	divaricatacid	Ribonuclease 4	RNASE4
1589	MOL011737	divaricatacid	Complement component C8 gamma chain	C8G
1590	MOL011737	divaricatacid	Glutaredoxin-1	GLRX
1591	MOL011737	divaricatacid	GTP-binding protein Rheb	RHEB
1592	MOL011737	divaricatacid	Catalase	CAT
1593	MOL011737	divaricatacid	Complement C1s subcomponent	C1S
1594	MOL011737	divaricatacid	Tryptophan 5-hydroxylase 1	TPH1
1595	MOL011737	divaricatacid	Galectin-2	LGALS2
1596	MOL011737	divaricatacid	GTPase HRas	HRAS
1597	MOL011737	divaricatacid	ADP-ribosylation factor-like protein 5A	ARL5A
1598	MOL011737	divaricatacid	Arginase-1	ARG1
1599	MOL011737	divaricatacid	Hydroxyacylglutathione hydrolase, mitochondrial	HAGH
1600	MOL011737	divaricatacid	Glutathione S-transferase omega-1	GSTO1
1601	MOL011737	divaricatacid	Pleckstrin homology domain-containing family A member 4	PLEKH A4
1602	MOL011737	divaricatacid	Delta-aminolevulinic acid dehydratase	ALAD
1603	MOL011737	divaricatacid	Phosphatidylinositol transfer protein alpha isoform	PITPNA
1604	MOL011737	divaricatacid	Phosphopantothenoylcysteine decarboxylase	PPCDC
1605	MOL011737	divaricatacid	GMP reductase 1	GMPR
1606	MOL011737	divaricatacid	Signal transducer and activator of transcription 1-alpha/beta	STAT1
1607	MOL011737	divaricatacid	L-xylulose reductase	DCXR
1608	MOL011737	divaricatacid	Non-secretory ribonuclease	RNASE2
1609	MOL011737	divaricatacid	Thiopurine S-methyltransferase	TPMT
1610	MOL011737	divaricatacid	G-protein coupled receptor 35	GPR35
1611	MOL011737	divaricatacid	Hypoxia-inducible factor 1-alpha	HIF1A
1612	MOL011737	divaricatacid	Cyclic AMP-responsive element-binding protein 1	CREB1
1613	MOL011737	divaricatacid	Ornithine decarboxylase	ODC1
1614	MOL011737	divaricatacid	Malate dehydrogenase, cytoplasmic	MDH1
1615	MOL011737	divaricatacid	MAP kinase-interacting serine/threonine-protein kinase 2	MKNK2
1616	MOL011737	divaricatacid	Lysine-specific demethylase 4A	KDM4A

1617	MOL011737	divaricatacid	cGMP-specific 3',5'-cyclic phosphodiesterase	PDE5A
1618	MOL011737	divaricatacid	Cysteinyl leukotriene receptor 1	CYSLTR 1
1619	MOL011737	divaricatacid	Lysine-specific demethylase 4C	KDM4C
1620	MOL011737	divaricatacid	G protein-coupled receptor kinase 6	GRK6
1621	MOL011737	divaricatacid	Mannose-6-phosphate isomerase	MPI
1622	MOL011737	divaricatacid	Inosine-5'-monophosphate dehydrogenase 2	IMPDH2
1623	MOL011737	divaricatacid	Inosine-5'-monophosphate dehydrogenase 1	IMPDH1
1624	MOL011737	divaricatacid	Egl nine homolog 1	EGLN1
1625	MOL011737	divaricatacid	Mast/stem cell growth factor receptor Kit	KIT
1626	MOL011737	divaricatacid	Aminopeptidase N	ANPEP
1627	MOL011737	divaricatacid	Endothelin-converting enzyme 1	ECE1
1628	MOL011737	divaricatacid	Lysine-specific demethylase 4D	KDM4D
1629	MOL011737	divaricatacid	2-Hydroxyacid oxidase 2	HAO2
1630	MOL011737	divaricatacid	AMP deaminase 3	AMPD3
1631	MOL011737	divaricatacid	ATP-citrate synthase	ACLY
1632	MOL011737	divaricatacid	Insulin-like growth factor-binding protein 3	IGFBP3
1633	MOL011737	divaricatacid	Caspase-6	CASP6
1634	MOL011737	divaricatacid	Free fatty acid receptor 1	FFAR1
1635	MOL011737	divaricatacid	Caspase-2	CASP2
1636	MOL011737	divaricatacid	Prostaglandin D2 receptor 2	PTGDR2
1637	MOL011737	divaricatacid	Glycogen phosphorylase, muscle form	PYGM
1638	MOL011737	divaricatacid	Prolyl hydroxylase EGLN3	EGLN3
1639	MOL011737	divaricatacid	Glycogen synthase kinase-3 alpha	GSK3A
1640	MOL011737	divaricatacid	Lysine-specific demethylase 3A	KDM3A
1641	MOL011737	divaricatacid	Lysine-specific demethylase 5B	KDM5B
1642	MOL011737	divaricatacid	Sialidase-2	NEU2
1643	MOL011737	divaricatacid	Prostacyclin receptor	PTGIR
1644	MOL011737	divaricatacid	Insulin-degrading enzyme	IDE
1645	MOL011740	divaricatol	Histone-lysine N-methyltransferase SETD7	SETD7
1646	MOL011740	divaricatol	Fructose-bisphosphate aldolase A	ALDOA
1647	MOL011740	divaricatol	Ras-related protein Rab-11A	RAB11A
1648	MOL011740	divaricatol	Adenine phosphoribosyltransferase	APRT
1649	MOL011740	divaricatol	Trifunctional purine biosynthetic protein adenosine-3	GART

1650	MOL011740	divaricatol	Ras-related C3 botulinum toxin substrate 1	RAC1
1651	MOL011747	ledebouriellol	Thymidine phosphorylase	TYMP
1652	MOL011747	ledebouriellol	Rho GTPase-activating protein 1	ARHGAP1
1653	MOL011747	ledebouriellol	Transcription factor p65	RELA
1654	MOL011749	phelloptorin	Dual specificity protein phosphatase 6	DUSP6
1655	MOL011749	phelloptorin	Tissue-type plasminogen activator	PLAT
1656	MOL011753	5-O-Methylvisamminol	Cell division control protein 42 homolog	CDC42
1657	MOL011753	5-O-Methylvisamminol	Glycolipid transfer protein	GLTP
1658	MOL011753	5-O-Methylvisamminol	GMP reductase 2	GMPR2
1659	MOL011753	5-O-Methylvisamminol	Sulfotransferase 1A1	SULT1A1
1660	MOL011753	5-O-Methylvisamminol	Ras-related protein Rap-2a	RAP2A
1661	MOL011753	5-O-Methylvisamminol	ADP-ribosylation factor-like protein 5B	ARL5B
1662	MOL011753	5-O-Methylvisamminol	GTP-binding nuclear protein Ran	RAN
1663	MOL011753	5-O-Methylvisamminol	Isovaleryl-CoA dehydrogenase, mitochondrial	IVD
1664	MOL011753	5-O-Methylvisamminol	Sepiapterin reductase	SPR
1665	MOL012537	Spinoside A	Tyrosine-protein kinase receptor UFO	AXL
1666	MOL012537	Spinoside A	Signal transducer and activator of transcription 3	STAT3
1667	MOL012542	β-ecdysterone	Serine/threonine-protein kinase receptor R3	ACVRL1
1668	MOL012542	β-ecdysterone	Equilibrative nucleoside transporter 1	SLC29A1
1669	MOL012542	β-ecdysterone	Interleukin-1 receptor-associated kinase 4	IRAK4
1670	MOL013077	Decursin	Apoptotic protease-activating factor 1	APAF1
1671	MOL013077	Decursin	Tyrosine-protein kinase BTK	BTK
1672	MOL013077	Decursin	Alpha-synuclein	SNCA
1673	MOL013077	Decursin	Glucagon receptor	GCGR
1674	MOL013077	Decursin	UDP-N-acetylglucosamine--peptide N-acetylglucosaminyltransferase 110 kDa subunit	OGT
1675	MOL013077	Decursin	Mitogen-activated protein kinase kinase kinase kinase 4	MAP4K4
1676	MOL013077	Decursin	Aurora kinase A	AURKA

1677	MOL013077	Decursin	cGMP-inhibited 3',5'-cyclic phosphodiesterase 3A	PDE3A
1678	MOL013077	Decursin	cGMP-inhibited 3',5'-cyclic phosphodiesterase 3B	PDE3B
1679	MOL013077	Decursin	Pyruvate kinase PKM	PKM
1680	MOL013077	Decursin	A disintegrin and metalloproteinase with thrombospondin motifs 5	ADAMTS5
1681	MOL013077	Decursin	Metabotropic glutamate receptor 1	GRM1
1682	MOL013077	Decursin	Sodium/glucose cotransporter 1	SLC5A1
1683	MOL013077	Decursin	Bromodomain-containing protein 4	BRD4
1684	MOL013077	Decursin	Tyrosyl-DNA phosphodiesterase 2	TDP2
1685	MOL013077	Decursin	Indoleamine 2,3-dioxygenase 1	IDO1
1686	MOL013077	Decursin	Serine/threonine-protein kinase Chk2	CHEK2
1687	MOL013077	Decursin	Serine/threonine-protein kinase PLK3	PLK3
1688	MOL013077	Decursin	Serine/threonine-protein kinase PLK2	PLK2
1689	MOL013077	Decursin	Vascular endothelial growth factor receptor 3	FLT4
1690	MOL013077	Decursin	Neuronal acetylcholine receptor subunit alpha-7	CHRNA7
1691	MOL013077	Decursin	Cyclin-dependent kinase 8	CDK8
1692	MOL013077	Decursin	NAD-dependent protein deacetylase sirtuin-2	SIRT2
1693	MOL013077	Decursin	Intercellular adhesion molecule 1	ICAM1
1694	MOL013077	Decursin	Serine/threonine-protein kinase pim-2	PIM2
1695	MOL013077	Decursin	Dual specificity protein kinase TTK	TTK
1696	MOL013281	6,8-Dihydroxy-7-methoxyxanthone	Fibroblast growth factor receptor 1	FGFR1
1697	MOL013281	6,8-Dihydroxy-7-methoxyxanthone	Branched-chain-amino-acid aminotransferase, cytosolic	BCAT1
1698	MOL013281	6,8-Dihydroxy-7-methoxyxanthone	Interleukin-6	IL6
1699	MOL013287	Physovenine	Bombesin receptor subtype-3	BRS3
1700	MOL013287	Physovenine	Long-chain fatty acid transport protein 1	SLC27A1
1701	MOL013287	Physovenine	Bromodomain-containing protein 2	BRD2
1702	MOL013287	Physovenine	Bromodomain-containing protein	BRD3

			3	
1703	MOL013287	Physovenine	Thromboxane A2 receptor	TBXA2R
1704	MOL013288	Picralinal	Histone-lysine N-methyltransferase, H3 lysine-79 specific	DOT1L
1705	MOL013288	Picralinal	Protoporphyrinogen oxidase	PPOX
1706	2355	Bergapten	Serine/threonine-protein kinase PLK4	PLK4
1707	2355	Bergapten	Mitogen-activated protein kinase kinase kinase 8	MAP3K8
1708	2355	Bergapten	Intermediate conductance calcium-activated potassium channel protein 4	KCNN4
1709	122841	Aspidinol	Histone chaperone ASF1A	ASF1A
1710	122841	Aspidinol	Myoglobin	MB
1711	122841	Aspidinol	Arylamine N-acetyltransferase 1	NAT1
1712	122841	Aspidinol	Plectin	PLEC
1713	122841	Aspidinol	Nischarin	NISCH
1714	122841	Aspidinol	Toll-like receptor 1	TLR1
1715	122841	Aspidinol	Pituitary adenylate cyclase-activating polypeptide type I receptor	ADCYAP1R1
1716	122841	Aspidinol	L-lactate dehydrogenase A chain	LDHA
1717	122841	Aspidinol	Sodium- and chloride-dependent glycine transporter 2	SLC6A5
1718	191694	Cudranone	LIM domain kinase 1	LIMK1
1719	191694	Cudranone	Protein kinase C eta type	PRKCH
1720	191694	Cudranone	Protein kinase C beta type	PRKCB
1721	191694	Cudranone	C-terminal-binding protein 2	CTBP2
1722	509244	Cudraflavanone B	Histone deacetylase 3	HDAC3
1723	509244	Cudraflavanone B	Voltage-dependent T-type calcium channel subunit alpha-1H	CACNA1H
1724	667495	(2R)-5,7-dihydroxy-2-(4-hydroxyphenyl)-2,3-dihydro-4H-chromen-4-one	Pyruvate dehydrogenase E1 component subunit beta, mitochondrial	PDHB
1725	667495	(2R)-5,7-dihydroxy-2-(4-hydroxyphenyl)-2,3-dihydro-4H-chromen-4-one	Tyrosyl-DNA phosphodiesterase 1	TDP1
1726	667495	(2R)-5,7-dihydroxy-2-(4-hydroxyphenyl)-2,3-dihydro-4H-chromen-4-one	CDGSH iron-sulfur domain-containing protein 1	CISD1
1727	667495	(2R)-5,7-dihydroxy-2-(4-hydroxyphenyl)-2,3-dihydro-4H-chromen-4-	6-phosphogluconate dehydrogenase, decarboxylating	PGD

		one		
1728	667495	(2R)-5,7-dihydroxy-2-(4-hydroxyphenyl)-2,3-dihydro-4H-chromen-4-one	CMP-N-acetylneuraminate-beta-1,4-galactoside alpha-2,3-sialyltransferase	ST3GAL3
1729	667495	(2R)-5,7-dihydroxy-2-(4-hydroxyphenyl)-2,3-dihydro-4H-chromen-4-one	Kallikrein-2	KLK2
1730	667495	(2R)-5,7-dihydroxy-2-(4-hydroxyphenyl)-2,3-dihydro-4H-chromen-4-one	Phosphoglycerate mutase 1	PGAM1
1731	5281814	Wighteone	Endoplasmin	HSP90B1
1732	5281814	Wighteone	Tyrosine-protein phosphatase non-receptor type 12	PTPN12
1733	5319744	3'-O-Methylorobol	Bifunctional heparan sulfate N-deacetylase/N-sulfotransferase 1	NDST1
1734	5495919	Toxyloxanthone C	PH domain leucine-rich repeat-containing protein phosphatase 2	PHLPP2
1735	5495920	1,3,7-Trihydroxy-2-prenylxanthone	Fatty acid synthase	FASN
1736	10716607	Cudraxanthone R	Soluble calcium-activated nucleotidase 1	CANT1
1737	10716607	Cudraxanthone R	Maternal embryonic leucine zipper kinase	MELK
1738	10716607	Cudraxanthone R	Type-1 angiotensin II receptor	AGTR1
1739	10716607	Cudraxanthone R	CCR4-NOT transcription complex subunit 7	CNOT7
1740	11609510	7-Hydroxy-2-(4-hydroxyphenyl)-8-(3-methylbut-2-enyl)-2,3-dihydrochromen-4-one	Serine/threonine-protein kinase TAO3	TAOK3
1741	11609510	7-Hydroxy-2-(4-hydroxyphenyl)-8-(3-methylbut-2-enyl)-2,3-dihydrochromen-4-one	Presequence protease, mitochondrial	PITRM1
1742	11709692	4',7-Dihydroxy-2',5-dimethoxy-flavonol	Vasopressin V2 receptor	AVPR2
1743	11709692	4',7-Dihydroxy-2',5-dimethoxy-flavonol	Serine/threonine-protein kinase Nek2	NEK2

Supplementary Table S4. The target genes of RA

No	Gene	No	Gene
1	PTPN22	1146	TRA
2	SLC22A4	1147	TFF3
3	TNF	1148	TIE1
4	CRP	1149	TIMP2
5	IL6ST	1150	TIMP3
6	CCR6	1151	ACTG1
7	FCGR2A	1152	TPI1
8	PADI4	1153	CRISP2
9	HLA-DPB1	1154	ACTG2
10	HLA-DRB1	1155	MUC5B
11	IL2RA	1156	TNFSF4
12	IL6R	1157	TXNRD1
13	IL10	1158	VIL1
14	IRF5	1159	VTN
15	CIITA	1160	YY1
16	STAT4	1161	VTCN1
17	TNFAIP3	1162	SCD5
18	TRAF1	1163	PDCD1LG2
19	TRAF6	1164	SLC7A5
20	CD28	1165	ADAMTS12
21	CD40	1166	TLR10
22	PTPN2	1167	CAMP
23	TYK2	1168	H4C9
24	RUNX1	1169	H4C1
25	PTPRC	1170	H4C4
26	AGER	1171	H4C6
27	IL2RB	1172	H4C12
28	NFKBIL1	1173	H4C11
29	AFF3	1174	H4C3
30	REL	1175	H4C8
31	BLK	1176	H4C2
32	CDK6	1177	H4C5
33	CD244	1178	H4C13
34	MMEL1	1179	H4C14
35	ANKRD55	1180	PRAM1
36	GATA3	1181	SPZ1
37	NFKBIE	1182	FCRLA
38	PLD4	1183	ORAI1
39	DNASE1L3	1184	PIK3R3
40	KIF5A	1185	ABCC11
41	ARID5B	1186	BECN1
42	RASGRP1	1187	FADD

43	CSF2	1188	CD84
44	CTLA4	1189	HDAC3
45	ACAN	1190	CCK
46	RCAN1	1191	APLN
47	AHR	1192	PER2
48	SPRED2	1193	CCRL2
49	ENO1	1194	FCGR2C
50	ANXA3	1195	LPAR2
51	HLA-DQA2	1196	XPR1
52	IFNG	1197	CD6
53	CCN1	1198	ASIC3
54	IL1B	1199	CD163
55	IL1RN	1200	KL
56	IL6	1201	FHL5
57	CXCL8	1202	ENTPD1
58	IL18	1203	BMS1
59	MIF	1204	CDC42
60	MMP2	1205	SCO2
61	MPO	1206	MIR760
62	MTHFR	1207	SH2B3
63	NCF2	1208	LINC01672
64	TNFRSF11B	1209	RPL17-C18orf32
65	FOXP3	1210	LINC-ROR
66	IL23A	1211	ADAM8
67	ABCB1	1212	CDK2
68	PON1	1213	CALCRL
69	PTGS2	1214	PRG4
70	CCL21	1215	SPRY1
71	SLC11A1	1216	RAMP2
72	STAT1	1217	CCL26
73	TLR2	1218	WARS2
74	VEGFA	1219	LINC02210-CRHR1
75	CXCR4	1220	SEMA4D
76	CAT	1221	CEBPD
77	ADIPOQ	1222	KAT5
78	CCN2	1223	IVNS1ABP
79	TNFSF14	1224	EBP
80	DHFR	1225	CTSC
81	TNFRSF14	1226	CFTR
82	TAGAP	1227	CCR9
83	IRAK1	1228	CHAT
84	COL2A1	1229	PSIP1
85	ALOX5	1230	IRAK3
86	GC	1231	KLF12

87	HOXD13	1232	SAAL1
88	NCF1	1233	TIRAP
89	TXNDC5	1234	MIR155HG
90	GRK2	1235	CHUK
91	FPGS	1236	C1QTNF3
92	IGFBP3	1237	C1QTNF6
93	FASLG	1238	TADA1
94	ATIC	1239	LTB4R
95	PRKCQ	1240	CNR1
96	PTGS1	1241	PPARGC1B
97	CCL8	1242	COX8A
98	SOD2	1243	CPB1
99	GGH	1244	CR1
100	B3GNT2	1245	ATF2
101	CP	1246	CREBBP
102	PLB1	1247	CRHR1
103	MMP12	1248	CSF3
104	ACKR3	1249	CSK
105	PRDM1	1250	CST3
106	CD83	1251	ADRA1A
107	ADORA2A	1252	CTSB
108	CLEC12A	1253	ADRA2B
109	IKZF3	1254	BTLA
110	FKBP5	1255	CYP2D6
111	CXCL2	1256	CYP3A5
112	HSD11B1	1257	CYP17A1
113	RBPJ	1258	CYP19A1
114	STS	1259	CYP26A1
115	MMP10	1260	DNAJB1P1
116	BGN	1261	NLRP6
117	BMP4	1262	DHCR7
118	SMS	1263	AFP
119	ABCC5	1264	DNASE1
120	ABCC4	1265	DNTT
121	CTSD	1266	DR1
122	DDX6	1267	DRD2
123	GRK6	1268	JAG1
124	HCLS1	1269	TSC22D3
125	HOXD10	1270	DUSP1
126	PXK	1271	EDN1
127	BCL2A1	1272	EFNB1
128	BMP6	1273	EIF4EBP1
129	NR4A3	1274	ELAVL1
130	GDF5	1275	ELF3

131	CAV2	1276	ELK3
132	ABCC3	1277	EPAS1
133	GPRC5A	1278	FBL
134	CD2	1279	ETS2
135	LHX2	1280	F2R
136	MAB21L2	1281	ALDH1A1
137	TXNIP	1282	PTK2B
138	ABCC2	1283	FCER2
139	HAPLN1	1284	FCN2
140	GSDME	1285	FGB
141	F2	1286	FGF1
142	LY96	1287	FGF13
143	GART	1288	CASC3
144	RGMB	1289	COG2
145	NR3C1	1290	FOXO1
146	HOXD11	1291	KDM6B
147	IRF8	1292	FLT4
148	ITGA6	1293	BRD4
149	LCN2	1294	ALPP
150	MARCKS	1295	TMEM158
151	RAB8A	1296	ASPM
152	PLEK	1297	GAPDH
153	POU3F1	1298	ATRNL1
154	DDIT4	1299	GAS6
155	GIN1	1300	HSPB8
156	BAIAP2L1	1301	GH1
157	PSG5	1302	CBLIF
158	NDUFA4L2	1303	LAMP3
159	PTK2	1304	HPLH1
160	RAP2A	1305	PDLIM3
161	BDKRB2	1306	ABO
162	CXCL6	1307	CCR10
163	TLE3	1308	GPR15
164	TMPO	1309	VSTM1
165	TNFAIP2	1310	DLL1
166	ZFP36	1311	TRAV29DV5
167	TFPI2	1312	GPR42
168	CALD1	1313	TRAJ60
169	ST6GALNAC5	1314	TRAC
170	MGARP	1315	GPX1
171	B3GNT9	1316	ANPEP
172	RUNX2	1317	SETD2
173	SLC25A12	1318	GRP
174	CD3E	1319	GSK3B

175	CD5	1320	MSH6
176	ABCG2	1321	SENP1
177	MMP14	1322	A1CF
178	ZAP70	1323	TBX21
179	DNASE2	1324	SLC40A1
180	HNRNPA2B1	1325	HDAC1
181	CD226	1326	HDGF
182	PADI2	1327	ADGRE2
183	FCRL3	1328	HGF
184	HLA-A	1329	ANXA6
185	HLA-B	1330	HIP1
186	HLA-C	1331	NR4A1
187	HLA-DQA1	1332	HRAS
188	HLA-DQB1	1333	HSD11B2
189	ISG20	1334	HSPA8
190	TAP2	1335	BIRC5
191	DPP4	1336	DNAJB1
192	AIRE	1337	ICA1
193	MICA	1338	ID1
194	HLA-DMB	1339	IFNGR1
195	ATG5	1340	IGFBP1
196	AIF1	1341	IGHA1
197	C5	1342	APP
198	MECP2	1343	APRT
199	NOTCH4	1344	KLK3
200	TNIP1	1345	IL1RAP
201	CCR3	1346	IL5RA
202	HLA-DOA	1347	IL9R
203	HLA-DPA1	1348	IL10RB
204	PTPN11	1349	IL11
205	CFB	1350	IDO1
206	SFTPD	1351	INHBA
207	TSBP1	1352	INSR
208	CCL27	1353	INSRR
209	PSORS1C1	1354	AQP9
210	MICB	1355	ITGA2
211	APOM	1356	ITGAE
212	RAD51B	1357	ITGAX
213	BACH2	1358	AREG
214	PRRC2A	1359	KCNMA1
215	DDX39B	1360	MALAT1
216	LST1	1361	KIR2DL3
217	CDSN	1362	KIR2DS5
218	RPP14	1363	KLK1

219	ICOSLG	1364	KNG1
220	PHF19	1365	ARG1
221	UBASH3A	1366	KPNA1
222	PRKCH	1367	CCL4L1
223	GSDMB	1368	LDLR
224	BTNL2	1369	LGALS2
225	COG6	1370	LGALS3BP
226	C2	1371	LGALS8
227	UBE2L3	1372	LIG4
228	NELFE	1373	LRPAP1
229	KIAA1109	1374	SH2D1A
230	CASP10	1375	MIR132
231	MACIR	1376	MIR152
232	SYNGR1	1377	MIR17
233	CD247	1378	MIR192
234	COL4A1	1379	MIR210
235	COL11A2	1380	MIR214
236	DAXX	1381	MIR222
237	ETFA	1382	MIR29A
238	RTKN2	1383	MIR30A
239	HSPA1L	1384	MIR98
240	ICAM3	1385	SMAD7
241	SMTNL2	1386	MCAM
242	IL12RB2	1387	MET
243	FADS1	1388	MFAP1
244	MBP	1389	MFAP4
245	EMCN	1390	MKI67
246	ATP6V1G2	1391	ASIP
247	SUPT20H	1392	MOG
248	PSMB8	1393	MRC1
249	PSMB9	1394	MRE11
250	WDFY4	1395	ZFAS1
251	RARB	1396	MIR346
252	GPSM3	1397	ASPA
253	CARD9	1398	MST1
254	TNXB	1399	COX1
255	TPD52	1400	MUTYH
256	MPIG6B	1401	MYO9B
257	TMEM187	1402	ATHS
258	MYO18B	1403	ATM
259	NTN1	1404	NGF
260	SH3PXD2A	1405	NHS
261	ELMO1	1406	NME1
262	NAT2	1407	NOS1

263	ZDHHC20P2	1408	NOTCH3
264	TMPOP1	1409	NPPA
265	WAKMAR2	1410	NT5E
266	HNRNPA1P2	1411	ODC1
267	NONOP2	1412	OXA1L
268	UBDP1	1413	G0S2
269	AP4B1-AS1	1414	NOX4
270	CYP4F23P	1415	PAK1
271	PSMB8-AS1	1416	PAM
272	MUC22	1417	PAX5
273	MSH5-SAPCD1	1418	ASCC1
274	ATP6V1G2-DDX39B	1419	ADIPOR1
275	PPT2-EGFL8	1420	PCNA
276	CDH11	1421	PLEKHO1
277	TSPAN5	1422	PDC
278	TRIM10	1423	TNFRSF12A
279	LINC01934	1424	TRPV2
280	LNCPRESS1	1425	PDE4A
281	LINC00824	1426	PDE7A
282	TSBP1-AS1	1427	PDGFRA
283	C3orf67-AS1	1428	ENPP2
284	C2-AS1	1429	ADA2
285	HCG9	1430	PGR
286	LOC102723407	1431	PITX2
287	ASB15-AS1	1432	PLA2G4A
288	LOC102724971	1433	TREM2
289	STAG1	1434	POU2AF1
290	NEBL	1435	ARL15
291	LINC02356	1436	AHI1
292	LINC02357	1437	ATG16L1
293	LINC02571	1438	NAT10
294	ETV7-AS1	1439	PRELP
295	LINC01748	1440	CHD7
296	AGPAT1	1441	WDR11
297	CXCL13	1442	MAP2K1
298	AHSA1	1443	CCL28
299	CDC42EP3	1444	SLC12A9
300	EHMT2-AS1	1445	CEMIP
301	MDC1-AS1	1446	AICDA
302	CELF2	1447	MIR410
303	TNFSF13B	1448	KIDINS220
304	MTCO2P12	1449	MIB1
305	PHTF1	1450	PTN
306	LINC02196	1451	CIP2A

307	HCP5	1452	PHRF1
308	PAPOLA	1453	BAK1
309	EHMT2	1454	RAC2
310	OS9	1455	MOK
311	HTD2	1456	RAF1
312	TRIM31	1457	BCHE
313	CHI3L1	1458	PLAAT4
314	FAM107A	1459	RB1
315	NRM	1460	UBL5
316	IL17F	1461	RBP2
317	SMIM40	1462	RDX
318	NLRP3	1463	RNASE2
319	CSMD3	1464	BCL6
320	PHACTR3	1465	RNF2
321	RPL3P2	1466	IGAN1
322	IP6K3	1467	RORC
323	CLIC1	1468	RPL17
324	CCR5	1469	SAA3P
325	ZPBP2	1470	SAT1
326	RAVER1	1471	CCL4
327	CNTFR	1472	CCL17
328	RBM45	1473	CCL19
329	FLACC1	1474	CCL22
330	TRIM40	1475	CCL25
331	MUCL3	1476	SDC2
332	SGCZ	1477	TINAGL1
333	ATF6B	1478	SEMA4A
334	CRH	1479	CLEC7A
335	CRK	1480	AGXT2
336	CRYGD	1481	SLC2A3
337	ASB15	1482	SLC6A2
338	MAPK14	1483	SUMO3
339	DRAIC	1484	SUMO2
340	CSNK2B	1485	SNCA
341	IL34	1486	SOD1
342	CSTF3	1487	SOD3
343	IL23R	1488	SPARC
344	LINC01104	1489	SSRP1
345	ZNF595	1490	SSTR4
346	ERICH1	1491	STAT5A
347	CYP21A2	1492	STAT6
348	DGKQ	1493	BRS3
349	MDGA2	1494	CNTN2
350	ZNF679	1495	TBCA

351	CLYBL	1496	GCFC2
352	DLG2	1497	TG
353	EGFR	1498	TGFA
354	C3orf67	1499	TGM2
355	TRIM39-RPP21	1500	THBD
356	EPHB2	1501	THOP1
357	AKT1	1502	THY1
358	ESR1	1503	TIA1
359	ALB	1504	TLR1
360	FCGR3A	1505	TTR
361	FCGR3B	1506	POTEF
362	JAZF1	1507	TNFRSF4
363	NKAPL	1508	TYROBP
364	FGF2	1509	UQCRFS1
365	GPC5	1510	VPREB1
366	DKK1	1511	WARS1
367	ABCF1	1512	WNT3
368	PLCL2	1513	WNT10B
369	PDS5A	1514	YWHAZ
370	FN1	1515	CA1
371	SIRT1	1516	IL1R2
372	TNPO3	1517	IRX1
373	SDF2L1	1518	ADIPOR2
374	IL27	1519	RHBDF2
375	HCG27	1520	ASRGL1
376	WDR27	1521	TAM
377	GABBR1	1522	CAPG
378	ASF1A	1523	FCRL4
379	RNF19A	1524	JAM3
380	RCHY1	1525	LOH19CR1
381	LY6G6F	1526	CASP5
382	MAGI3	1527	ROPN1L
383	POLDIP2	1528	CASP7
384	FBXW8	1529	NLRC5
385	GCH1	1530	MINDY4
386	GEM	1531	CUL1
387	HLA-S	1532	IL17RC
388	GFRA1	1533	SEMA7A
389	DKK3	1534	UBASH3B
390	IL37	1535	CAV1
391	IL17B	1536	SERPINH1
392	HPGDS	1537	TNFSF12
393	GNL1	1538	TNFRSF10B
394	GPI	1539	IL18RAP

395	LINC00294	1540	NRP1
396	CXCR3	1541	CCN6
397	HECTD4	1542	SPHK1
398	TMEM235	1543	MBD4
399	HCG22	1544	MBD2
400	GSTM1	1545	PGLYRP1
401	GSTT1	1546	PSTPIP1
402	GUCY1B2	1547	CD1D
403	HIF1A	1548	PDCD5
404	HLA-DOB	1549	INTS4
405	HLA-DPB2	1550	CD8A
406	HLA-DQB2	1551	DNER
407	HLA-DQB3	1552	KLF4
408	HLA-DRA	1553	CD22
409	HLA-DRB4	1554	TNFRSF8
410	HLA-DRB9	1555	ADAMTS3
411	HLA-G	1556	TBPL1
412	HMGB1	1557	CCL4L2
413	HSPA4	1558	CD48
414	HSPD1	1559	IKBKE
415	ICAM1	1560	SDC3
416	IFNA1	1561	FAM53B
417	IFNA13	1562	CD70
418	IGF1	1563	HDAC9
419	FAS	1564	ADGRE5
420	IL1A	1565	PIEZO1
421	IL2	1566	KEAP1
422	IL4	1567	SPATA2
423	IL7	1568	CYP2C9
424	IL9	1569	CYP2E1
425	IL13	1570	CYP2A6
426	IL15	1571	CYP3A4
427	IL17A	1572	UGT1A6
428	ILF3	1573	UGT2B15
429	CXCL10	1574	SULT1A1
430	AR	1575	PTGES3
431	ITPR3	1576	TRPV1
432	JAK1	1577	FAAH
433	JAK2	1578	AKR1C1
434	JAK3	1579	SLC22A6
435	GALNT18	1580	CYP2C19
436	KDR	1581	TNFAIP6
437	KIR3DL1	1582	NEU1
438	ZKSCAN4	1583	PRDX5

439	TMEM179	1584	XDH
440	SFTA2	1585	GSTA1
441	COL11A2P1	1586	GSTA2
442	LEP	1587	HPRT1
443	LGALS3	1588	NUDT15
444	LINC02649	1589	SLC29A2
445	LINC02656	1590	SLC28A3
446	GATA3-AS1	1591	IMPDH1
447	LMNA	1592	IMPDH2
448	LINC01185	1593	ITPA
449	LINC00243	1594	CYP3A7
450	MCCD1	1595	CYP1B1
451	TRIM26BP	1596	CYP2B6
452	MTCO3P1	1597	CYP2C8
453	LTA	1598	SERPINA6
454	MIR146A	1599	SLCO1A2
455	MIR155	1600	SCN10A
456	MIR223	1601	PTGER1
457	DDR1-DT	1602	PDPK1
458	HCG18	1603	CA2
459	HCG17	1604	CA3
460	MBL2	1605	ABCB11
461	MEFV	1606	CYP1A1
462	MAP3K4	1607	ACE2
463	MMP1	1608	MC2R
464	MMP3	1609	HSD3B2
465	MMP9	1610	CYP27B1
466	MMP13	1611	PPP3R2
467	BCL2L15	1612	SLC10A2
468	MYL8P	1613	SLCO1B1
469	MSH5	1614	PPIF
470	MSI1	1615	SLCO1B3
471	COX2	1616	NR0B1
472	MTF1	1617	CYP3A43
473	MTX1	1618	CYP4A11
474	LRRC18	1619	CYP11B1
475	NFKB1	1620	ABCC1
476	NM	1621	SLC22A11
477	NOS3	1622	UGT2B7
478	ACR	1623	CYP2C18
479	NUP88	1624	UGT2B4
480	IL20	1625	SCN4A
481	IL22	1626	ASIC1
482	SNHG32	1627	KCNQ2

483	PBX2	1628	KCNQ3
484	CPA4	1629	PLA2G2A
485	PDCD1	1630	RXRA
486	PDE2A	1631	SERPINA7
487	WNT16	1632	PPARA
488	RAB14	1633	CYP11B2
489	CYRIB	1634	SRD5A2
490	SERPINA1	1635	HSD3B1
491	PIK3CA	1636	SLCO2B1
492	PIK3CB	1637	FABP2
493	PIK3CD	1638	GP1BA
494	PIK3CG	1639	S100A7
495	PLCL1	1640	AMACR
496	IL17D	1641	SLC22A7
497	PLG	1642	PGD
498	GDAP1	1643	AOX1
499	KRT20	1644	SLC16A1
500	CCHCR1	1645	SLCO3A1
501	NECAB2	1646	SLC46A1
502	POU5F1	1647	SLCO4C1
503	RSBN1	1648	FOLR1
504	RBFOX1	1649	SLC15A1
505	PPIAP9	1650	SLC36A1
506	VPS37C	1651	AKR1C2
507	ENOX1	1652	AKR1C4
508	KIF26B	1653	CLCNKA
509	PPP1R10	1654	SLC16A7
510	ACOXL	1655	ATP4A
511	TCP11L1	1656	HRH2
512	CDK5RAP2	1657	ACHE
513	ZNF302	1658	SLC22A2
514	MAPK1	1659	ACAT1
515	MAPK8	1660	SLC7A11
516	PRL	1661	NFKB2
517	PRTN3	1662	PPARD
518	TRIM39	1663	PTGDR2
519	RETN	1664	AKR1B10
520	PSMA4	1665	FKBP1A
521	PRDM10	1666	ORM1
522	VARS2	1667	GLRA1
523	MIR499A	1668	TDO2
524	PRR12	1669	H19
525	DENND1A	1670	COPA
526	ANO8	1671	SNHG29

527	NCOA5	1672	MIR150
528	C6orf47	1673	LACC1
529	LY6G5B	1674	CHRNG
530	LY6G6D	1675	UCA1
531	IL21	1676	SNHG28
532	RARA	1677	FBXL19-AS1
533	BCL2	1678	SYK
534	RELA	1679	IL1RAPL2
535	ACTB	1680	MIF-AS1
536	RIT2	1681	IL6-AS1
537	RNF5	1682	LOC126859963
538	RPL37A	1683	LOC100287329
539	RPS18	1684	CD79A
540	RPS19	1685	CD4
541	S100A8	1686	RELN
542	SAA1	1687	GAS5
543	VPS52	1688	MAFB
544	CCL2	1689	OLAH
545	CCL5	1690	RIPK1
546	CCL20	1691	LTF
547	CXCL12	1692	RELB
548	PRDM16	1693	HSPA5
549	SELE	1694	TFRC
550	CLSTN2	1695	SIAE
551	NOD2	1696	CD36
552	LINC00452	1697	APOH
553	HLA-DPA2	1698	BST1
554	GORASP1	1699	ELANE
555	SKIV2L	1700	SAA4
556	WNK1	1701	ANXA5
557	PCDH15	1702	CPT2
558	CASTOR1	1703	B2M
559	SLC6A11	1704	PPP1CA
560	SLC19A1	1705	LIN54
561	SOAT1	1706	ZNF354A
562	SPP1	1707	USP50
563	STAT3	1708	FRG2C
564	SNORA38	1709	CILP
565	BSG	1710	PPP1CB
566	SUOX	1711	MAP3K8
567	SYT1	1712	PPP1CC
568	TAPBP	1713	LRRK2
569	BTF3P11	1714	CTSG
570	TCF19	1715	GNAQ

571	BTK	1716	ADORA3
572	TRBV20OR9-2	1717	HAS1
573	TEC	1718	SRF
574	TGFB1	1719	PRSS2
575	TIMP1	1720	LECT2
576	TLR3	1721	LTBR
577	TLR4	1722	DEK
578	TNFRSF1A	1723	ANKH
579	TNFRSF1B	1724	ERAP1
580	TP53	1725	PLA2G10
581	TRPS1	1726	SIGLEC1
582	DCDC2C	1727	ZNF334
583	TYMS	1728	GOLGB1
584	VARS1	1729	IL22RA1
585	VCAM1	1730	TNFRSF18
586	VDR	1731	IL36RN
587	VIM	1732	WAS
588	VIP	1733	XIST
589	TRIM26	1734	INS
590	ZNF175	1735	CERNA3
591	ZSCAN26	1736	HMGB2
592	DDR1	1737	ERAP2
593	DDA1	1738	HGD
594	FTO	1739	RAG2
595	BAG6	1740	MIR146B
596	GPANK1	1741	PLA2G7
597	HSD17B8	1742	MIR125A
598	OR2H2	1743	MVK
599	RNASEH2B	1744	TRPV4
600	AIMP2	1745	MMP7
601	AGBL2	1746	TMSB4X
602	RPP21	1747	IFNG-AS1
603	ATAT1	1748	APOA1
604	SPSB1	1749	PRKCD
605	ALPK1	1750	TNFRSF13B
606	CUL5	1751	CALCA
607	SLC44A4	1752	CD58
608	VWA7	1753	NLRP12
609	LY6G6C	1754	CARD14
610	LY6G5C	1755	GHRL
611	ZNRD1ASP	1756	FGFR1
612	PRRT1	1757	PVT1
613	EGFL8	1758	RAG1
614	CDR3	1759	COL9A2

615	OR5V1	1760	FBN1
616	OR12D3	1761	BDNF-AS
617	CASP3	1762	COX5A
618	UQCC2	1763	TF
619	DHX16	1764	MIR142
620	PGBD1	1765	POMC
621	HAVCR2	1766	IGKC
622	AIFM2	1767	SRC
623	CEP89	1768	COL1A1
624	PPIL4	1769	BLTP1
625	TNFSF11	1770	SLC17A5
626	TNFRSF11A	1771	MMP15
627	SYNGAP1	1772	PSORS1C3
628	STK19	1773	XIAP
629	NAV2	1774	TMX2-CTNND1
630	TRIM15	1775	ENG
631	CCNG2	1776	BPI
632	SOCS3	1777	MIR381
633	IL33	1778	MIR151A
634	RABEP1	1779	NEAT1
635	IL32	1780	MIR133B
636	CD14	1781	PLAUR
637	MS4A1	1782	ACTA2
638	PPT2	1783	CSN1S1
639	GRAP2	1784	MIR23A
640	FADS2	1785	LBR
641	GABBR2	1786	EPO
642	CLOCK	1787	LINC00426
643	CD40LG	1788	LINC02384
644	CD44	1789	MIR494
645	MDC1	1790	OPRM1
646	CD68	1791	BCL2L1
647	N4BP1	1792	MIR320A
648	PRORP	1793	COL9A3
649	RIPOR2	1794	CFI
650	CTIF	1795	CXCR2P1
651	SMG7	1796	CCL11
652	TNFSF15	1797	FAM167A
653	EBI3	1798	LRBA
654	CDKN2A	1799	DCN
655	CNTRL	1800	MIR99B
656	CDC42EP1	1801	ALOX5AP
657	COMP	1802	LERFS
658	PARP1	1803	TTC7A

659	CX3CR1	1804	MIR197
660	ACE	1805	TGFB2
661	FCGR2B	1806	IGHM
662	FOXO3	1807	TREX1
663	FOS	1808	ADAMTS7
664	HAVCR1	1809	CALCR
665	HLA-DMA	1810	PTGER4
666	IL3	1811	KIT
667	JUN	1812	CEACAM8
668	CX3CL1	1813	PRF1
669	THBS1	1814	KCNJ11
670	CCR2	1815	COL11A1
671	LRPPRC	1816	COL5A2
672	FSTL1	1817	P4HA2
673	LINC02605	1818	ATXN2
674	CSF1	1819	PICSAR
675	CARD8	1820	MATN1
676	IL4R	1821	FCAR
677	IL7R	1822	TACR3
678	MMP8	1823	NPY
679	WG	1824	PLAT
680	PRKN	1825	MMP17
681	SOST	1826	IBSP
682	TREM1	1827	PARN
683	MAPK3	1828	CENPB
684	MYDGF	1829	H2AC18
685	PTX3	1830	BRD2
686	RFC1	1831	HJV
687	CXCR5	1832	UNC13D
688	ADAM17	1833	GBA1
689	TPO	1834	UBA1
690	C3	1835	NLRC4
691	ARHGEF5	1836	TFR2
692	SYVN1	1837	ALPL
693	TNFRSF6B	1838	MYOM2
694	IL18R1	1839	NPPB
695	TIMELESS	1840	PNP
696	PTGES	1841	NTAN1
697	SEMA3A	1842	IRAK4
698	ANP32B	1843	TGFBR2
699	CDAN1	1844	MIR106A
700	CTSK	1845	KITLG
701	CBLL2	1846	LSM2
702	ETS1	1847	AMPD1

703	F2RL1	1848	MTHFD1
704	IFNL1	1849	LEPQTL1
705	ANGPT2	1850	SLX1A-SULT1A3
706	CD274	1851	RNPC3
707	ICOS	1852	LPIN2
708	CFH	1853	LBH
709	IL12B	1854	NAGLU
710	KIR2DS1	1855	CYP21A1P
711	LPA	1856	AIM2
712	MIR21	1857	EMSLR
713	NFE2L2	1858	MRAP
714	NOS2	1859	BCL2L11
715	OSM	1860	LCK
716	P2RX7	1861	IL15RA
717	SERPINE1	1862	TGFBR1
718	MBL3P	1863	ACP1
719	PML	1864	ELN
720	PPARG	1865	CYLD-AS1
721	S100A9	1866	RHOA
722	SAA2	1867	C5-OT1
723	CCL3	1868	DCLRE1C
724	TRIM21	1869	SOD2-OT1
725	TAC1	1870	MIR498
726	TLR5	1871	ENPP1
727	C4B	1872	SMAD2
728	MUL1	1873	TRH
729	CALR	1874	SERPINC1
730	CASP1	1875	C1QB
731	TNFSF13	1876	PIP4K2C
732	CD19	1877	MIR31
733	CD34	1878	IL12RB1
734	CD69	1879	MIR769
735	C4B_2	1880	PDGFRB
736	KLRC4-KLRK1	1881	MIR141
737	ADM	1882	LOC126862264
738	HT	1883	RO60
739	DMD	1884	MIR382
740	ESR2	1885	CD209
741	KLRK1	1886	IFNA2
742	TBC1D9	1887	YDJC
743	FLT1	1888	GSR
744	FOLR2	1889	SPTAN1
745	IL17RA	1890	MIR16-1
746	GABPA	1891	IFNGR2

747	GCG	1892	ADAD1
748	GLP1R	1893	CD81
749	ANGPT1	1894	TGIF1
750	IGHV3-69-1	1895	ASAH1
751	GZMB	1896	ELP1
752	HP	1897	COL9A1
753	AGBL3	1898	XK
754	IL16	1899	SNORD44
755	IRF4	1900	MIR629
756	ITGAV	1901	HSP90AB1
757	KIR3DL2	1902	MIR575
758	LAIR1	1903	MIR543
759	LGALS9	1904	MIR483
760	LIF	1905	TH
761	MDK	1906	MIR486-1
762	MYD88	1907	U2AF1
763	NOTCH1	1908	MIR203A
764	IL21R	1909	MIR122
765	CLEC4A	1910	CD59
766	TLR7	1911	GSN
767	GDE1	1912	PERP
768	TLR9	1913	WIPF1
769	BANK1	1914	ELMO2
770	MAP2K7	1915	GPX3
771	HAMP	1916	TAB1
772	BDNF	1917	NTRK1
773	CCL7	1918	TRA-TGC7-1
774	BRAF	1919	IRF3
775	TEK	1920	SLAMF1
776	C4A	1921	MIR99A
777	SUMO1	1922	PDGFB
778	BEST1	1923	GGT1
779	WNT5A	1924	IL1RL1
780	XRCC1	1925	RBP4
781	ZBTB16	1926	FLI1
782	CAST	1927	CYTOR
783	CASP8	1928	PIK3R1
784	TNFRSF25	1929	PGR-AS1
785	ADAM15	1930	IL1RL2
786	NR1I2	1931	CD101
787	CD86	1932	CEBPB
788	ADA	1933	HLA-DRB6
789	KIR2DS2	1934	MIR15B
790	CDKN1A	1935	COL10A1

791	NFAT5	1936	MIR199B
792	LILRB1	1937	COL5A1
793	BTG3	1938	IL24
794	CISH	1939	TYR
795	TNFRSF13C	1940	LEPR
796	CCR7	1941	MIR92B
797	CNR2	1942	MAPK10
798	SLCO6A1	1943	AKT2
799	CREB1	1944	ENSG00000083622
800	CTSL	1945	MAP3K14
801	ADRB2	1946	AXL
802	CYP1A2	1947	IL36A
803	OLIG3	1948	SLC2A9
804	DHODH	1949	VIPR1
805	DNMT1	1950	FLT3
806	EGR1	1951	ERBB3
807	FCGR1A	1952	RPLP1
808	NLRP1	1953	KCNQ1
809	FOSB	1954	MIRLET7E
810	MTOR	1955	BMP7
811	ACAD8	1956	YWHAH
812	B3GAT1	1957	IGHE
813	GSTM2	1958	MSN
814	GSTP1	1959	HNF1A-AS1
815	TNC	1960	CDK4
816	APOE	1961	PADI6
817	IGF1R	1962	MIR500A
818	LINC01193	1963	DUSP22
819	IGHG3	1964	TAB2
820	IL12A	1965	ANXA2
821	ITGAM	1966	TRAF2
822	ITGB1	1967	FCN3
823	JUNB	1968	SOX9
824	JUND	1969	NCF4
825	GSTK1	1970	ADRB3
826	KLRB1	1971	MB
827	RPSA	1972	MIR663A
828	LGALS1	1973	HNRNPC
829	MIR126	1974	MAP3K1
830	MIR22	1975	FAP
831	MIR221	1976	SPI1
832	MIR34A	1977	AHSG
833	MCL1	1978	HLA-DQB1-AS1
834	MDM2	1979	IL2RG

835	CXCL9	1980	TTN
836	NFATC1	1981	PPBP
837	NR4A2	1982	IREB2
838	HSPA14	1983	SSB
839	PLA2G1B	1984	ZNF774
840	PLAU	1985	THPO
841	PTEN	1986	PLOD2
842	PTH	1987	MMP11
843	CXCL16	1988	RIPK2
844	RAC1	1989	CAMLG
845	RARRES2	1990	SNORD15A
846	TNFRSF17	1991	F5
847	ROS1	1992	GNAS
848	S100A4	1993	IFI16
849	S100A12	1994	ACTR2
850	SP1	1995	ICAM2
851	MAP3K7	1996	PABIR2
852	TRB	1997	TSIX
853	TSPO	1998	MIR326
854	TM7SF2	1999	GHSR
855	VPS51	2000	GATA4
856	VEGFC	2001	IL36G
857	DHX40	2002	MIR154
858	TSLP	2003	GFAP
859	SOCS1	2004	SLC22A12
860	TNFSF10	2005	CDKN3
861	TNFRSF10A	2006	MIR379
862	CFLAR	2007	HDAC4
863	SQSTM1	2008	SHMT1
864	CD80	2009	PSORS1C2
865	ADAMTS4	2010	MAP2K6
866	GDF15	2011	COL1A2
867	CD38	2012	FCGRT
868	HOTAIR	2013	HSPA1A
869	ZGLP1	2014	A2M
870	IL18BP	2015	MAPKAPK2
871	NAMPT	2016	ADAR
872	RABEPK	2017	XBP1
873	LANCL1	2018	ITGA5
874	CDKN2D	2019	TYRO3
875	NXF1	2020	HLA-E
876	LOC105379528	2021	PROCR
877	KHDRBS1	2022	KRT18
878	CXCR6	2023	EP300

879	MALT1	2024	REL-DT
880	EBNA1BP2	2025	MIR26B
881	H3P13	2026	TRIP11
882	H3P28	2027	FAS-AS1
883	LRG1	2028	GLA
884	CLU	2029	VAV1
885	SERPINA3	2030	SULT1A3
886	CCR4	2031	FGFR2
887	CR2	2032	SALL3
888	CRABP2	2033	MIR125B1
889	CSF1R	2034	MIR204
890	CTNNB1	2035	MTNR1B
891	CYBB	2036	PTGER2
892	DECR1	2037	RNU6-1
893	ATN1	2038	RIGI
894	S1PR1	2039	TCN1
895	LPAR1	2040	ZNF438
896	EDNRA	2041	RPS6KA4
897	EGF	2042	TAC3
898	F3	2043	CDKAL1
899	F9	2044	FGFR3
900	MMRN1	2045	CHST11
901	FOXC1	2046	MIR124-1
902	FLNB	2047	TERT
903	SEC14L2	2048	CHRM3
904	FPR2	2049	PRKG1-AS1
905	BBC3	2050	CD1A
906	GLB1	2051	CYCS
907	IGHV3OR16-7	2052	ITLN1
908	IGHV3-52	2053	INPP5B
909	TRBV16	2054	MIR181D
910	TRBV7-9	2055	MIR212
911	TRBC1	2056	MIR211
912	GPT	2057	ATRIP
913	GRN	2058	ATRIP-TREX1
914	RMC1	2059	TREH
915	IL19	2060	STING1
916	ANXA1	2061	MIR215
917	ACACA	2062	MIR7-3HG
918	HLA-DRB3	2063	ZFAT-AS1
919	HOXD9	2064	MIR130A
920	APC	2065	CHRNA7
921	HTR2A	2066	LSP1
922	ARMH1	2067	ROCR

923	IFNB1	2068	BMP5
924	IGFBP5	2069	SEMA3C
925	LCE3B	2070	KRT18P39
926	LCE3C	2071	PFKM
927	IKBKB	2072	TGFB3
928	IL5	2073	UGT1A1
929	CXCR2	2074	IGHG2
930	IL10RA	2075	MIR199A1
931	ITGA1	2076	NGFR
932	KIR2DL2	2077	SPATA31F1
933	KLRC1	2078	C1QA
934	SUMO4	2079	SHH
935	LOC390714	2080	CYB5A
936	LBP	2081	TERC
937	LOX	2082	MIR100
938	MIR10A	2083	IL36B
939	MIR145	2084	MIR5196
940	CD46	2085	HAS2
941	MIP	2086	WNT1
942	MMP19	2087	MC3R
943	PAEP	2088	CLEC3B
944	ANGPTL4	2089	CLNK
945	PECAM1	2090	F13A1
946	PGF	2091	ARAP1
947	PRKCA	2092	MAP2K3
948	IL26	2093	BAX
949	PSMD7	2094	RNF182
950	MIR451A	2095	ALDH2
951	CCND1	2096	PPP3CA
952	REG1A	2097	RPA3
953	REN	2098	ANGEL2
954	S100A1	2099	PTPN12
955	S100B	2100	MIR600
956	CCL3L1	2101	MMP16
957	CXCL5	2102	TAX1BP1
958	SELL	2103	MIR27B
959	SELP	2104	SNRNP70
960	MAP2K4	2105	IL17C
961	SFRP1	2106	ABHD16A
962	SHBG	2107	CCN4
963	SLC22A5	2108	TLR6
964	FSCN1	2109	IL20RB
965	SOX5	2110	YPEL5
966	TAP1	2111	MMP20

967	TBX5	2112	MAPK9
968	TPMT	2113	QDPR
969	C5AR1	2114	MIRLET7C
970	TXN	2115	LPL
971	VWF	2116	MIR423
972	STEAP4	2117	MIR106B
973	EHMT1	2118	GNRHR
974	CD276	2119	NLGN3
975	IL1F10	2120	IRF2BP2
976	USO1	2121	ATOD1
977	TP63	2122	RNGTT
978	CD1C	2123	BATF
979	ARHGEF2	2124	MIR22HG
980	TIMD4	2125	UGDH
981	MSC	2126	MC4R
982	CD27	2127	MIR191
983	ADAM10	2128	PWAR4
984	DDX39A	2129	NRXN1
985	SPRY2	2130	ARHGEF3
986	SIK1B	2131	DCAF5
987	KLF2	2132	CCL15
988	NOD1	2133	SLAMF7
989	PDPN	2134	HSPG2
990	TRAF3IP2	2135	MAP3K2
991	HPSE	2136	CASP9
992	LILRA3	2137	COL3A1
993	ADRM1	2138	MIR766
994	ADAMTS5	2139	PCSK9
995	CD160	2140	EOMES
996	SLC7A9	2141	TRD
997	POLG2	2142	RPS12P4
998	PRRT2	2143	MIR199A2
999	ACOT7	2144	PRKDC
1000	CTHRC1	2145	JDP2
1001	H4-16	2146	NTF3
1002	CCR1	2147	MIR34C
1003	OSCAR	2148	ENSG00000274029
1004	SIK1	2149	MT-CYB
1005	CD55	2150	C1S
1006	DBP	2151	IAPP
1007	GADD45A	2152	EZR
1008	DNMT3B	2153	AGTR1
1009	DPEP1	2154	MAPK13
1010	E2F2	2155	QPCT

1011	TYMP	2156	GAST
1012	ELAVL2	2157	NFKBIB
1013	PRSS55	2158	BST2
1014	EPHB1	2159	CCL1
1015	ERBB2	2160	HARS1
1016	ERG	2161	MIR638
1017	EZH2	2162	CASR
1018	FCGR1B	2163	DNMT3A
1019	FCN1	2164	TXNDC11
1020	FHL1	2165	SCGB1A1
1021	FLG	2166	RPL7
1022	ACSBG1	2167	CD200R1
1023	FLT3LG	2168	NEDD9
1024	CLEC16A	2169	CASC2
1025	DICER1	2170	FMOD
1026	SMUG1	2171	PLA2G5
1027	NUP62	2172	TNFSF18
1028	SH3BP4	2173	SLAMF6
1029	ALOX15	2174	CGAS
1030	FRZB	2175	WDR26
1031	AOC1	2176	CPEB4
1032	COPD	2177	MIR532
1033	MSTN	2178	ADAMTSL1
1034	GDNF	2179	IL31
1035	SGSM3	2180	CUTALP
1036	GLS	2181	FNDC1
1037	PYCARD	2182	SMIM20
1038	CXCL1	2183	MIR26A1
1039	GTF2H1	2184	IFNAR1
1040	GTF2I	2185	MIR373
1041	GUSB	2186	MIR149
1042	GZMA	2187	TUG1
1043	HFE	2188	IKBKG
1044	HLA-DRB5	2189	MOB3B
1045	HMOX1	2190	BMX
1046	HOXC6	2191	HCCAT5
1047	HSP90AA1	2192	PWAR1
1048	APOB	2193	HTR3A
1049	IDDM8	2194	SFTA3
1050	ACTBL2	2195	CLEC4E
1051	H3P44	2196	UCN
1052	IGF2	2197	MIR9-1
1053	IL1R1	2198	TCF7L2
1054	CXCR1	2199	MIR425

1055	AQP1	2200	GYPC
1056	TNFRSF9	2201	TNNT2
1057	IRF1	2202	PFKL
1058	IRS1	2203	TRHDE
1059	ITGA4	2204	ZNF311
1060	ABCC6	2205	LINC00158
1061	ITGAL	2206	HNF4A
1062	ITGB2	2207	LOC100506023
1063	KIR2DL1	2208	MIR200C
1064	KLRC2	2209	CNMD
1065	KLRD1	2210	G3BP1
1066	NPSR1	2211	BMS1P20
1067	LRP5	2212	HSPA1B
1068	LTB	2213	EFEMP1
1069	ARNTL	2214	ATP1A1
1070	MIR140	2215	XCR1
1071	MIR143	2216	MIR127
1072	MIR20A	2217	PSMD5
1073	MIR23B	2218	TYRP1
1074	MIR27A	2219	MIR30E
1075	MIR17HG	2220	FGF23
1076	SMAD3	2221	CMKLR1
1077	MAP3K5	2222	GHR
1078	FOXO4	2223	TCN2
1079	MPG	2224	SERPINF2
1080	MPP1	2225	HDAC2
1081	POTEKP	2226	LALBA
1082	MIR323A	2227	DRD1
1083	MIR338	2228	DNM1L
1084	MSRA	2229	ITGB7
1085	MTR	2230	IKZF1
1086	MTRR	2231	MIR522
1087	MUC1	2232	TSPAN33
1088	MYC	2233	MIR29C
1089	GADD45B	2234	FABP4
1090	NCAM1	2235	MIRLET7B
1091	NFKBIA	2236	FURIN
1092	DDR2	2237	PTPN3
1093	OGG1	2238	VEGFD
1094	OLR1	2239	MIR103A1
1095	P4HB	2240	C1QC
1096	PCSK6	2241	CEP57
1097	PAK3	2242	GRHL2
1098	DCTN4	2243	DGUOK-AS1

1099	GP6	2244	TANK
1100	TLR8	2245	MTNR1A
1101	PDE3A	2246	MLKL
1102	NCKIPSD	2247	CHIT1
1103	SIRT6	2248	PTPRT
1104	PADI3	2249	C1R
1105	SERPINF1	2250	IGES
1106	PF4	2251	MERTK
1107	PFKFB3	2252	EXOSC10
1108	PIK3R2	2253	TACR2
1109	SLCO1C1	2254	FLNA
1110	ACP5	2255	MIRLET7G
1111	PPIA	2256	MSMB
1112	ASPN	2257	HBEGF
1113	SLC52A1	2258	ADAMTS13
1114	ADI1	2259	CD52
1115	MEG3	2260	KCP
1116	H4C15	2261	VSIG4
1117	PRKCB	2262	CDH13
1118	PROC	2263	LETR1
1119	HTRA1	2264	MIR370
1120	PSMD12	2265	COMT
1121	PTCH1	2266	MEG8
1122	PTHLH	2267	MX1
1123	SEMA6A	2268	NOTCH2
1124	SIGIRR	2269	CBL
1125	HRH4	2270	PGK1
1126	RHO	2271	CNTF
1127	BCR	2272	DSG1
1128	RPS6KA3	2273	AAK1
1129	BGLAP	2274	GAK
1130	CCL13	2275	AKR1B1
1131	CCL18	2276	HMGCR
1132	CXCL11	2277	Malaria DHOdehase
1133	SDC1	2278	G6PD
1134	IFIH1	2279	HRH1
1135	POTEM	2280	MAPK12
1136	ST6GAL1	2281	OPRD1
1137	BMP2	2282	PDE4D
1138	RAPH1	2283	CSF2RA
1139	SLC22A1	2284	CELA1
1140	SLPI	2285	HIV tat
1141	SST	2286	ITGA4-ITGB7
1142	STAT5B	2287	PDE4B

1143	SULT2A1	2288	MAPK11
1144	ABCC8	2289	CTSS
1145	TACR1		

Supplementary Table S5. The 798 differentially expressed genes (DEGs)

No	Smybol	Up/Down	No	Smybol	Up/Down
1	ANGPTL7	Up	400	TRAF3IP3	Down
2	MAOA	Up	401	LCK	Down
3	ADH1B	Up	402	CD2	Down
4	ZBTB16	Up	403	PLEK	Down
5	GADD45A	Up	404	CD27	Down
6	KLF9	Up	405	PSMB9	Down
7	C6	Up	406	GFRA2	Down
8	AGTR1	Up	407	VOPP1	Down
9	PLIN1	Up	408	PTPRC	Down
10	FABP4	Up	409	ITGAL	Down
11	WASF3	Up	410	HK3	Down
12	PCK1	Up	411	CSK	Down
13	ADH1C	Up	412	DOCK10	Down
14	APOD	Up	413	CORO1A	Down
15	FOSL2	Up	414	LAMP3	Down
16	NFIL3	Up	415	IL32	Down
17	NOVA1	Up	416	MICB	Down
18	ATF3	Up	417	HLA-DMB	Down
19	LAMA2	Up	418	PLXNC1	Down
20	TIMP4	Up	419	ALOX5	Down
21	ABLIM1	Up	420	SELPLG	Down
22	MAFF	Up	421	CD48	Down
23	SPRY2	Up	422	LILRB2	Down
24	FAM107A	Up	423	APOBEC3F	Down
25	MAGI2	Up	424	NID2	Down
26	KLF4	Up	425	RUNX3	Down
27	TGFBR3	Up	426	ADCY7	Down
28	GSN	Up	427	OAS1	Down
29	CYP4B1	Up	428	AIF1	Down
30	MYOC	Up	429	OSBPL3	Down
31	FOXO3	Up	430	MARCKS	Down
32	RRAD	Up	431	FCN1	Down
33	MAP1B	Up	432	NREP	Down
34	VEGFD	Up	433	THY1	Down
35	BCL6	Up	434	CD79A	Down
36	TCF7L2	Up	435	LILRB3	Down
37	SVEP1	Up	436	SELL	Down

38	MTUS1	Up	437	LGALS2	Down
39	NPAS2	Up	438	PLCG2	Down
40	SMAD3	Up	439	RFX5	Down
41	GABARAPL1	Up	440	GZMB	Down
42	GADD45B	Up	441	MMP1	Down
43	SERPINA3	Up	442	CXCL10	Down
44	ZFP36	Up	443	COL1A2	Down
45	GDF15	Up	444	PTTG1	Down
46	TNS2	Up	445	NMI	Down
47	ADM	Up	446	CD86	Down
48	ITPKC	Up	447	KLRB1	Down
49	PPARG	Up	448	LST1	Down
50	LPL	Up	449	ICAM3	Down
51	AKR1B10	Up	450	ITGB2	Down
52	PPP1R15A	Up	451	AIM2	Down
53	TSPYL2	Up	452	MX2	Down
54	EDNRB	Up	453	LRRC15	Down
55	ZEB1	Up	454	NDC80	Down
56	DAAM2	Up	455	STAT1	Down
57	LTBP4	Up	456	CRIP1	Down
58	FMO2	Up	457	ARHGAP25	Down
59	SLC19A2	Up	458	SLC7A7	Down
60	ABTB2	Up	459	PLAAT4	Down
61	ADIPOQ	Up	460	MNDA	Down
62	JUNB	Up	461	HLA-DMA	Down
63	NTRK2	Up	462	GZMA	Down
64	IL1R1	Up	463	BMP2K	Down
65	AASS	Up	464	CXCL6	Down
66	MYH11	Up	465	CXCL9	Down
67	MYC	Up	466	CD8A	Down
68	RBPMS	Up	467	ARHGAP45	Down
69	PER1	Up	468	ACP2	Down
70	FOXO1	Up	469	ADAM28	Down
71	RND3	Up	470	LILRB4	Down
72	CX3CL1	Up	471	P2RX5	Down
73	SDC4	Up	472	CD6	Down
74	LMOD1	Up	473	RASSF2	Down
75	C1ORF21	Up	474	LCP1	Down
76	SOCS2	Up	475	PRF1	Down
77	RHOBTB3	Up	476	ITGA4	Down
78	TFPI	Up	477	IL7R	Down
79	FOSB	Up	478	SYK	Down
80	DCAF1	Up	479	VAV1	Down
81	CDH19	Up	480	ARHGDIB	Down

82	PLPP3	Up	481	CTSS	Down
83	CCN1	Up	482	SORL1	Down
84	WEE1	Up	483	SPOCK2	Down
85	IGFBP6	Up	484	NUP210	Down
86	PIGA	Up	485	C2	Down
87	PPL	Up	486	UBE2L6	Down
88	EIF1	Up	487	ITGB7	Down
89	LAMA3	Up	488	TRIM14	Down
90	SCD	Up	489	LEF1	Down
91	C7	Up	490	CCL18	Down
92	YTHDC1	Up	491	P2RY6	Down
93	AOC3	Up	492	ISG20	Down
94	CNN1	Up	493	RHOH	Down
95	NR4A1	Up	494	CST7	Down
96	ADIRF	Up	495	CASP1	Down
97	SOCS3	Up	496	SDC3	Down
98	EGR1	Up	497	ST6GAL1	Down
99	PTGS2	Up	498	KYNU	Down
100	DDR2	Up	499	ITK	Down
101	FSTL3	Up	500	LY86	Down
102	SORBS1	Up	501	DOK2	Down
103	DIO3	Up	502	PTPRE	Down
104	ACACB	Up	503	LPXN	Down
105	GLRX	Up	504	HCK	Down
106	KHDRBS3	Up	505	NCF2	Down
107	BMP5	Up	506	SLC46A3	Down
108	PLN	Up	507	GZMK	Down
109	JUND	Up	508	IL2RG	Down
110	DEPP1	Up	509	PPP1R16B	Down
111	NOL3	Up	510	SMPDL3A	Down
112	SFRP1	Up	511	LTB	Down
113	REV3L	Up	512	MSX2	Down
114	OLFM1	Up	513	RTN1	Down
115	GPD1	Up	514	CYFIP2	Down
116	SERPINA5	Up	515	GZMH	Down
117	FADS1	Up	516	AOAH	Down
118	ITGA7	Up	517	ADGRE2	Down
119	ADCY2	Up	518	BTN3A1	Down
120	TMEM47	Up	519	PTPN7	Down
121	ABCA8	Up	520	SEMA4D	Down
122	SLPI	Up	521	COL1A1	Down
123	IFRD1	Up	522	CR1	Down
124	STBD1	Up	523	GNLY	Down
125	GBE1	Up	524	SLC31A1	Down

126	CHRDL1	Up	525	DAZL	Down
127	SLC6A3	Up	526	SRGN	Down
128	CDC42EP4	Up	527	CYBA	Down
129	CBS	Up	528	GPR65	Down
130	FASN	Up	529	IKZF1	Down
131	TIPARP	Up	530	CPVL	Down
132	SRPX	Up	531	TK1	Down
133	HBEGF	Up	532	BTN3A2	Down
134	AMPH	Up	533	PRSS23	Down
135	EGFR	Up	534	CCR2	Down
136	NID1	Up	535	CTSH	Down
137	ZFHX3	Up	536	SH2D1A	Down
138	JUN	Up	537	ARPC5	Down
139	GPX3	Up	538	DPYSL3	Down
140	FAH	Up	539	PRDX4	Down
141	BTG2	Up	540	CTSC	Down
142	ALDH6A1	Up	541	CLEC10A	Down
143	PNPLA2	Up	542	WNT5A	Down
144	ATP9A	Up	543	NCF4	Down
145	LMO3	Up	544	BTK	Down
146	CEBPD	Up	545	THEMIS2	Down
147	FADS3	Up	546	GAP43	Down
148	AOX1	Up	547	AGA	Down
149	DUSP8	Up	548	SECTM1	Down
150	CTH	Up	549	ORAI2	Down
151	IER2	Up	550	S100A9	Down
152	DHCR7	Up	551	CD38	Down
153	NR4A2	Up	552	PRKCB	Down
154	ETS2	Up	553	CD14	Down
155	FKBP5	Up	554	CD53	Down
156	P2RY2	Up	555	TNFSF11	Down
157	ARID5B	Up	556	FCGR3B	Down
158	TMOD1	Up	557	FLRT2	Down
159	MTHFD2	Up	558	PAQR4	Down
160	INHBB	Up	559	COL5A2	Down
161	PTX3	Up	560	DOCK4	Down
162	KAT5	Up	561	LCP2	Down
163	ADH1A	Up	562	GMFG	Down
164	LIPE	Up	563	PSMB8	Down
165	GPRC5A	Up	564	CRLF3	Down
166	CDKN1A	Up	565	DOCK2	Down
167	DYRK3	Up	566	CSF2RB	Down
168	TOM1L1	Up	567	TNFSF10	Down
169	AOC2	Up	568	PLEKHB2	Down

170	EHBP1	Up	569	CRYBG1	Down
171	AR	Up	570	RRM2	Down
172	SASH1	Up	571	FYB1	Down
173	GGT5	Up	572	GBP1	Down
174	GOLGA2	Up	573	STK10	Down
175	APOB	Up	574	CD300C	Down
176	HES1	Up	575	FAP	Down
177	RB1CC1	Up	576	CCR7	Down
178	ATP1A2	Up	577	APLNR	Down
179	GRAMD1B	Up	578	CCR1	Down
180	PRG2	Up	579	HPS5	Down
181	ING1	Up	580	LAT	Down
182	NPY1R	Up	581	ST8SIA4	Down
183	CLDN5	Up	582	APOBEC3G	Down
184	LEP	Up	583	HLA-DPA1	Down
185	SPEG	Up	584	ZAP70	Down
186	SLC16A7	Up	585	OLFML1	Down
187	FGF2	Up	586	CA2	Down
188	ANG	Up	587	DCK	Down
189	TRIM2	Up	588	FCMR	Down
190	PTPRG	Up	589	CYBB	Down
191	PEG3	Up	590	FCGR3A	Down
192	TLN2	Up	591	PLA2G7	Down
193	CORO2B	Up	592	MAPK13	Down
194	RAMP2	Up	593	IDO1	Down
195	LDLR	Up	594	PSME2	Down
196	GPRASP1	Up	595	IL15	Down
197	SOX9	Up	596	RASGRP1	Down
198	PEX5	Up	597	FZD2	Down
199	PDE9A	Up	598	MAP4K1	Down
200	CDO1	Up	599	MAST1	Down
201	WNT11	Up	600	LTA4H	Down
202	RND1	Up	601	CX3CR1	Down
203	AQP7	Up	602	CD3E	Down
204	SEMA3E	Up	603	CD37	Down
205	SPOCK1	Up	604	S100A8	Down
206	SLC6A8	Up	605	FCGR2A	Down
207	PC	Up	606	MATK	Down
208	KCNK3	Up	607	ME2	Down
209	TNFSF9	Up	608	SH2D2A	Down
210	GEM	Up	609	IL10RA	Down
211	KCNAB1	Up	610	CXCL11	Down
212	HAS1	Up	611	CSF2RA	Down
213	DUSP1	Up	612	SLAMF1	Down

214	GPC3	Up	613	TPX2	Down
215	FOXC1	Up	614	GNG7	Down
216	VLDLR	Up	615	BATF	Down
217	NR1D1	Up	616	CCR6	Down
218	RNASE4	Up	617	ATP2A3	Down
219	HMGB2	Up	618	OAS2	Down
220	NPR1	Up	619	KIF11	Down
221	BMP4	Up	620	ADAM12	Down
222	ZFP36L2	Up	621	KCNN4	Down
223	CNR1	Up	622	LY96	Down
224	PNMT	Up	623	APOBEC3B	Down
225	FBLN2	Up	624	JAK2	Down
226	MPPED2	Up	625	NRGN	Down
227	GHR	Up	626	PNOC	Down
228	PCDH9	Up	627	BCL2A1	Down
229	ASS1	Up	628	TLR1	Down
230	EFHD1	Up	629	MOXD1	Down
231	KLHL21	Up	630	TRIM22	Down
232	ABHD5	Up	631	MS4A1	Down
233	GULP1	Up	632	CD72	Down
234	OSR2	Up	633	CCL13	Down
235	LAS1L	Up	634	CD180	Down
236	IER3	Up	635	COL3A1	Down
237	LHCGR	Up	636	MMP13	Down
238	PLP1	Up	637	CFP	Down
239	CRY2	Up	638	SKAP2	Down
240	G0S2	Up	639	ABHD3	Down
241	PLIN2	Up	640	SPP1	Down
242	HSPB2	Up	641	OLFML2B	Down
243	FXYD1	Up	642	TCL1A	Down
244	FNBP1L	Up	643	VNN2	Down
245	PDE8B	Up	644	CAPG	Down
246	ELL2	Up	645	PSMB10	Down
247	HSPB6	Up	646	ITGAX	Down
248	AGPAT2	Up	647	MDK	Down
249	NEUROG3	Up	648	ALPL	Down
250	ADGRG2	Up	649	NFE2L3	Down
251	ELANE	Up	650	CHN1	Down
252	RHOB	Up	651	VAMP8	Down
253	ACADL	Up	652	APOL1	Down
254	DDX3Y	Up	653	CREBL2	Down
255	SYN2	Up	654	FPR3	Down
256	RBP4	Up	655	QPCT	Down
257	TOX3	Up	656	CD33	Down

258	STON1	Up	657	BCL11A	Down
259	ZFAND5	Up	658	GATA3	Down
260	GJA4	Up	659	GGH	Down
261	ENPP1	Up	660	VEGFC	Down
262	FOXC2	Up	661	LAIR1	Down
263	PDK4	Up	662	SPINT2	Down
264	TF	Up	663	P2RX7	Down
265	DUSP4	Up	664	FCGR2B	Down
266	TAGLN	Up	665	RHOBTB1	Down
267	COBL	Up	666	CCNB2	Down
268	CRYAB	Up	667	SIT1	Down
269	ERI3	Up	668	CYTIP	Down
270	MAPK8	Up	669	CDK1	Down
271	HP	Up	670	IFI27	Down
272	LIFR	Up	671	CBR1	Down
273	PRKG1	Up	672	CDC20	Down
274	EFNB2	Up	673	LYZ	Down
275	ID4	Up	674	TCF7	Down
276	KLF5	Up	675	ARAP2	Down
277	PEMT	Up	676	ARHGAP4	Down
278	IRS2	Up	677	ST8SIA1	Down
279	PPFIA2	Up	678	LY9	Down
280	PITX1	Up	679	IL7	Down
281	GABRA2	Up	680	MMP9	Down
282	RGS16	Up	681	SLC39A8	Down
283	TPO	Up	682	GINS1	Down
284	SIX3	Up	683	CXCR4	Down
285	PPP1R3C	Up	684	CD19	Down
286	DES	Up	685	FBP1	Down
287	COL4A5	Up	686	MARCO	Down
288	MT1X	Up	687	GPR171	Down
289	SNED1	Up	688	CXADR	Down
290	PHKA1	Up	689	TBC1D31	Down
291	DIAPH2	Up	690	TMEM158	Down
292	TENM1	Up	691	TYROBP	Down
293	NLGN1	Up	692	CD3G	Down
294	ZNF230	Up	693	DLGAP5	Down
295	RETREG1	Up	694	ENTPD1	Down
296	SCNN1B	Up	695	CD58	Down
297	CRLF1	Up	696	MLLT11	Down
298	RPS4Y1	Up	697	SLC16A3	Down
299	VEGFA	Up	698	ROR2	Down
300	PLS1	Up	699	CTSB	Down
301	ARC	Up	700	LILRB1	Down

302	FOS	Up	701	LRRC17	Down
303	CCL25	Up	702	C3AR1	Down
304	AZGP1	Up	703	GSAP	Down
305	S100B	Up	704	CDK5	Down
306	FOXO4	Up	705	PCLAF	Down
307	CP	Up	706	CLDN7	Down
308	DPP4	Up	707	OLR1	Down
309	ANKRD28	Up	708	FOXM1	Down
310	EREG	Up	709	CEMIP	Down
311	EMX2	Up	710	STXBP2	Down
312	GPR20	Up	711	ATXN1	Down
313	GPC5	Up	712	TPD52	Down
314	CHST7	Up	713	ARRB2	Down
315	GATA6	Up	714	CD55	Down
316	FADS2	Up	715	CRTAM	Down
317	DDIT4	Up	716	PLXDC1	Down
318	MYOM1	Up	717	NUDT1	Down
319	YAP1	Up	718	EVI2A	Down
320	CASQ2	Up	719	FMNL1	Down
321	GYG2	Up	720	SLC31A2	Down
322	ACKR1	Up	721	TRAT1	Down
323	TSPAN8	Up	722	IL2RA	Down
324	TNF	Up	723	PKIA	Down
325	ASIC3	Up	724	EVI2B	Down
326	USP19	Up	725	CD7	Down
327	TSPAN1	Up	726	CNPY3	Down
328	USP9Y	Up	727	CXCR3	Down
329	BTC	Up	728	CD28	Down
330	PMAIP1	Up	729	BIRC3	Down
331	MYL9	Up	730	BUB1	Down
332	PDE3B	Up	731	DPT	Down
333	LIF	Up	732	LYL1	Down
334	PDZK1IP1	Up	733	TIMP1	Down
335	ACTG2	Up	734	NAIP	Down
336	EPHA7	Up	735	RASGRP3	Down
337	MAB21L1	Up	736	ICOS	Down
338	OLFM4	Up	737	COL5A1	Down
339	SGCA	Up	738	CCL19	Down
340	ADRA2C	Up	739	SCN9A	Down
341	TNFRSF10D	Up	740	KLRD1	Down
342	OVGP1	Up	741	FGF9	Down
343	WIF1	Up	742	IL18	Down
344	SAA4	Up	743	LOXL1	Down
345	EIF1AY	Up	744	P2RY10	Down

346	GAS1	Up	745	MELK	Down
347	HSD17B3	Up	746	PAK1	Down
348	CCL20	Up	747	TENM4	Down
349	CLUL1	Up	748	TFRC	Down
350	ODF2	Up	749	RSAD2	Down
351	IRX5	Up	750	SPI1	Down
352	GPM6B	Up	751	HLA-DRA	Down
353	FOSL1	Up	752	POSTN	Down
354	AREG	Up	753	TDO2	Down
355	SLC2A3	Up	754	CD40LG	Down
356	ZIC1	Up	755	NDP	Down
357	CXCL2	Up	756	PF4V1	Down
358	HLF	Up	757	IGLL1	Down
359	CLIC5	Up	758	IFIT1	Down
360	NLGN4Y	Up	759	TNFAIP6	Down
361	HOXA9	Up	760	SLCO2B1	Down
362	SOX5	Up	761	TEP1	Down
363	IL6	Up	762	C11ORF80	Down
364	GNRH1	Up	763	PRDM1	Down
365	MPP6	Up	764	SAC3D1	Down
366	MDM4	Up	765	MYBL1	Down
367	MATN3	Up	766	APOC4	Down
368	IGFBP5	Up	767	SFRP4	Down
369	SIM1	Up	768	MTM1	Down
370	PPP1R1A	Up	769	DCLRE1A	Down
371	ZNF674	Up	770	IL2RB	Down
372	IGF2	Up	771	CD24	Down
373	GSTT1	Up	772	CXCL1	Down
374	NFIX	Up	773	TFEC	Down
375	JCHAIN	Down	774	XAF1	Down
376	PTPRCAP	Down	775	TTC9	Down
377	ADAMDEC1	Down	776	LMNB1	Down
378	SEL1L3	Down	777	XCL1	Down
379	HLA-DOB	Down	778	MAP3K1	Down
380	PTPN6	Down	779	LAG3	Down
381	RAC2	Down	780	CYP27A1	Down
382	CD52	Down	781	EZH2	Down
383	CXCL13	Down	782	CAMTA1	Down
384	SDC1	Down	783	RNASE6	Down
385	CCL5	Down	784	DFFB	Down
386	CD247	Down	785	HLA-DRB4	Down
387	UCP2	Down	786	OMD	Down
388	CCR5	Down	787	CDH11	Down
389	BLNK	Down	788	C4BPB	Down

390	NKG7	Down	789	SLC16A4	Down
391	CD3D	Down	790	RAD17	Down
392	TNFRSF17	Down	791	GPR18	Down
393	TOP2A	Down	792	CCDC88C	Down
394	NCKAP1L	Down	793	CD79B	Down
395	MMP3	Down	794	FMO1	Down
396	MXRA5	Down	795	KIF23	Down
397	AQP9	Down	796	TSPAN2	Down
398	PIM2	Down	797	VNN1	Down
399	BTN3A3	Down	798	TAC1	Down

Note: Up/Down, RA group vs control group.

Supplementary Table S9. 170 core genes of the turquoise module

No	Gene	Module	MM_R	MM p value
1	ABLIM1	turquoise	-0.830750501	1.33E-08
2	ACP2	turquoise	0.85259493	2.22E-09
3	ADAMDEC1	turquoise	0.926937933	1.93E-13
4	ADH1B	turquoise	-0.859677585	1.17E-09
5	ADH1C	turquoise	-0.826615481	1.82E-08
6	AGTR1	turquoise	-0.864554404	7.34E-10
7	AIF1	turquoise	0.838068208	7.53E-09
8	AIM2	turquoise	0.832165544	1.20E-08
9	AKR1B10	turquoise	-0.837327039	7.99E-09
10	ALOX5	turquoise	0.895505283	2.37E-11
11	ANGPTL7	turquoise	-0.916405583	1.19E-12
12	APOBEC3C	turquoise	0.856283113	1.60E-09
13	APOBEC3F	turquoise	0.850180228	2.74E-09
14	AQP9	turquoise	0.870543165	4.05E-10
15	ARHGAP25	turquoise	0.871657196	3.61E-10
16	ARHGDIB	turquoise	0.819836554	2.98E-08
17	ARHGEF18	turquoise	0.834778727	9.77E-09
18	ARPC5	turquoise	0.801288312	1.04E-07
19	ATF3	turquoise	-0.869637575	4.44E-10
20	BCL6	turquoise	-0.868444512	5.00E-10
21	BLNK	turquoise	0.896945115	1.97E-11
22	BMP2K	turquoise	0.822500706	2.46E-08
23	BTN3A3	turquoise	0.884544501	8.92E-11
24	C1ORF21	turquoise	-0.80228217	9.74E-08
25	C2	turquoise	0.815753883	3.97E-08
26	C6	turquoise	-0.840230317	6.33E-09
27	CCL5	turquoise	0.904159465	7.46E-12

28	CCR5	turquoise	0.907712794	4.49E-12
29	CD2	turquoise	0.915920111	1.28E-12
30	CD247	turquoise	0.89199732	3.68E-11
31	CD27	turquoise	0.815658575	3.99E-08
32	CD3D	turquoise	0.892458153	3.47E-11
33	CD48	turquoise	0.831961192	1.22E-08
34	CD52	turquoise	0.946764916	2.59E-15
35	CD6	turquoise	0.877034694	2.05E-10
36	CD79A	turquoise	0.814192043	4.42E-08
37	CD8A	turquoise	0.818631254	3.24E-08
38	CMKLR1	turquoise	0.828868549	1.54E-08
39	COL1A1	turquoise	0.805362345	7.98E-08
40	CORO1A	turquoise	0.899981811	1.32E-11
41	CRIP1	turquoise	0.880071426	1.48E-10
42	CSK	turquoise	0.910737895	2.87E-12
43	CST7	turquoise	0.853448434	2.06E-09
44	CTSC	turquoise	0.811003394	5.49E-08
45	CTSS	turquoise	0.833488595	1.08E-08
46	CXCL13	turquoise	0.928782002	1.36E-13
47	CXCL6	turquoise	0.800885258	1.07E-07
48	CYBA	turquoise	0.834357747	1.01E-08
49	CYP4B1	turquoise	-0.863506584	8.12E-10
50	DOCK10	turquoise	0.872250703	3.40E-10
51	DOK2	turquoise	0.825733818	1.94E-08
52	DPYSL3	turquoise	0.806924543	7.20E-08
53	EDNRB	turquoise	-0.84569753	4.03E-09
54	EGR1	turquoise	-0.813129467	4.75E-08
55	EIF1	turquoise	-0.849902837	2.81E-09
56	FAM107A	turquoise	-0.854066461	1.95E-09
57	FOSL2	turquoise	-0.921434495	5.15E-13
58	FOXO3	turquoise	-0.865561088	6.65E-10
59	GABARAPL1	turquoise	-0.890030498	4.67E-11
60	GADD45A	turquoise	-0.921823969	4.81E-13
61	GADD45B	turquoise	-0.867878217	5.30E-10
62	GFRA2	turquoise	0.823577501	2.27E-08
63	GSN	turquoise	-0.864390343	7.46E-10
64	GZMB	turquoise	0.843299032	4.92E-09
65	HK3	turquoise	0.909369244	3.52E-12
66	HLA-DMA	turquoise	0.833399568	1.09E-08
67	HLA-DMB	turquoise	0.820333972	2.87E-08
68	HLA-DOB	turquoise	0.94725904	2.28E-15
69	HSPB1	turquoise	-0.846487169	3.77E-09
70	ICAM3	turquoise	0.888649516	5.52E-11
71	IGHM	turquoise	0.965288317	7.30E-18

72	IGKC	turquoise	0.954803486	2.75E-16
73	IGLC1	turquoise	0.903275216	8.43E-12
74	IGLV1-44	turquoise	0.943026188	6.55E-15
75	IGLV6-57	turquoise	0.837053527	8.17E-09
76	IL32	turquoise	0.868583005	4.94E-10
77	ISG20	turquoise	0.843354946	4.90E-09
78	ITGAL	turquoise	0.906363345	5.46E-12
79	ITGB7	turquoise	0.815168773	4.13E-08
80	JCHAIN	turquoise	0.911557994	2.54E-12
81	JUNB	turquoise	-0.81845738	3.28E-08
82	KLF4	turquoise	-0.886874465	6.81E-11
83	KLF9	turquoise	-0.94184014	8.68E-15
84	LAMA2	turquoise	-0.845768425	4.00E-09
85	LAMP3	turquoise	0.837096534	8.14E-09
86	LCK	turquoise	0.936117444	3.12E-14
87	LGALS2	turquoise	0.827538038	1.70E-08
88	LILRB2	turquoise	0.817137936	3.60E-08
89	LILRB3	turquoise	0.879148399	1.63E-10
90	LMOD1	turquoise	-0.819179074	3.12E-08
91	LST1	turquoise	0.837079534	8.15E-09
92	LTB	turquoise	0.8348526	9.71E-09
93	MAFF	turquoise	-0.889715475	4.86E-11
94	MAGI2	turquoise	-0.858560017	1.29E-09
95	MAN2B1	turquoise	0.810003328	5.87E-08
96	MAOA	turquoise	-0.902242376	9.72E-12
97	MAP1B	turquoise	-0.829364525	1.48E-08
98	MARCKS	turquoise	0.84828671	3.23E-09
99	MICB	turquoise	0.871058282	3.84E-10
100	MMP1	turquoise	0.851003995	2.55E-09
101	MMP3	turquoise	0.836073427	8.83E-09
102	MTUS1	turquoise	-0.810812082	5.56E-08
103	MX2	turquoise	0.880056272	1.48E-10
104	MXRA5	turquoise	0.904593653	7.02E-12
105	MYC	turquoise	-0.803955485	8.75E-08
106	MYO1F	turquoise	0.876386492	2.20E-10
107	NAGA	turquoise	0.881141358	1.31E-10
108	NCKAP1L	turquoise	0.893757609	2.95E-11
109	NDC80	turquoise	0.808739597	6.39E-08
110	NFIL3	turquoise	-0.864197956	7.60E-10
111	NID2	turquoise	0.841500226	5.71E-09
112	NKG7	turquoise	0.953471218	4.10E-16
113	NOVA1	turquoise	-0.841247284	5.83E-09
114	NPAS2	turquoise	-0.855472172	1.72E-09
115	OAS1	turquoise	0.858517722	1.30E-09

116	P2RX5	turquoise	0.848653133	3.13E-09
117	PARD3	turquoise	-0.853302048	2.09E-09
118	PCK1	turquoise	-0.854711156	1.84E-09
119	PIGA	turquoise	-0.833334861	1.09E-08
120	PIM2	turquoise	0.878768961	1.70E-10
121	PLAAT4	turquoise	0.882300585	1.15E-10
122	PLCG2	turquoise	0.906847991	5.09E-12
123	PLIN1	turquoise	-0.836134737	8.78E-09
124	PLXNC1	turquoise	0.833785483	1.06E-08
125	PRDX4	turquoise	0.821432183	2.66E-08
126	PRF1	turquoise	0.829672353	1.45E-08
127	PRKCB	turquoise	0.815915799	3.92E-08
128	PSMB8	turquoise	0.804143961	8.64E-08
129	PSMB9	turquoise	0.917668227	9.68E-13
130	PSTPIP1	turquoise	0.828637997	1.56E-08
131	PTGS2	turquoise	-0.800823705	1.07E-07
132	PTPN6	turquoise	0.943471804	5.89E-15
133	PTPRC	turquoise	0.854063725	1.95E-09
134	PTPRCAP	turquoise	0.902309834	9.63E-12
135	PTTG1	turquoise	0.853839687	1.99E-09
136	RAC2	turquoise	0.913929456	1.76E-12
137	RASSF2	turquoise	0.837754913	7.72E-09
138	RFX5	turquoise	0.86212306	9.27E-10
139	RGS19	turquoise	0.824271763	2.16E-08
140	RND3	turquoise	-0.800311078	1.10E-07
141	RPS6KA1	turquoise	0.868396264	5.03E-10
142	RUNX3	turquoise	0.856216838	1.61E-09
143	SDC1	turquoise	0.952048745	6.20E-16
144	SDC3	turquoise	0.824134076	2.18E-08
145	SEL1L3	turquoise	0.928120044	1.55E-13
146	SELPLG	turquoise	0.858534083	1.30E-09
147	SEMA4D	turquoise	0.869819863	4.36E-10
148	SLC19A2	turquoise	-0.850189345	2.74E-09
149	SLC7A7	turquoise	0.82776785	1.67E-08
150	SMAD3	turquoise	-0.822747344	2.42E-08
151	SPRY2	turquoise	-0.868482976	4.99E-10
152	STK10	turquoise	0.826948351	1.77E-08
153	SVEP1	turquoise	-0.822010023	2.55E-08
154	SYK	turquoise	0.809555982	6.05E-08
155	TCF7L2	turquoise	-0.831592885	1.25E-08
156	TFPI	turquoise	-0.847974213	3.32E-09
157	TGFBR3	turquoise	-0.828666529	1.56E-08
158	THY1	turquoise	0.862839332	8.66E-10
159	TNFRSF17	turquoise	0.940229191	1.26E-14

160	TOP2A	turquoise	0.881104666	1.32E-10
161	TRAF3IP3	turquoise	0.927946602	1.60E-13
162	TRBC1	turquoise	0.939510265	1.48E-14
163	UBE2L6	turquoise	0.871618465	3.63E-10
164	UCP2	turquoise	0.940813796	1.10E-14
165	VAV1	turquoise	0.838795532	7.11E-09
166	VEGFD	turquoise	-0.819198481	3.11E-08
167	VOPP1	turquoise	0.906022911	5.73E-12
168	WASF3	turquoise	-0.823610574	2.27E-08
169	ZEB1	turquoise	-0.834438151	1.00E-08
170	ZFP36	turquoise	-0.852609588	2.22E-09

Supplementary Table S10. 10 candidate targets in the PPI network

No	Rank	Name	Degree
1	1	TNF	62
2	2	IL6	59
3	3	MMP9	48
4	4	PTPRC	45
5	5	JUN	41
6	5	CXCR4	41
7	5	EGFR	41
8	8	CCL5	38
9	9	PPARG	36
10	10	STAT1	35

Supplementary Table S11-1. Abbreviated identifiers (ID) of chinese medicinal herbs in the network diagram.

No	Latin name	Pingyin name	Identifiers (ID)
1	*Citrus reticulata*	Chengpi	CP
2	*Clematis chinensis*	Weilingxian	WLX
3	*Atractylodes macrocephala*	Baizhu	BZ
4	*Cibotium barometz*	Gouji	GJ
5	*Aconitum kusnezoffii*	Caowu	CW
6	*Cinnamomum cassia*	Guizhi	GZ
7	*Dipsacus asper*	Xuduan	XD
8	*Saposhnikovia divaricata*	Fangfeng	FF
9	*Reynoutria japonica*	Huzhang	HZ
10	*Coix lacryma-jobi*	Yiyiren	YYR
11	*Poria cocos*	Fuling	FL
12	*Achyranthes bidentata*	Niuxi	NX
13	*Cibotium barometz*	Gouji	GC
14	*Aconitum carmichaelii*	Chuanwu	CHW

Supplementary Table S11-2. Abbreviated identifiers (ID) of active ingredients in the network diagram.

No	Molecular name	Identifiers (ID)
1	5,7-dihydroxy-2-(3-hydroxy-4-methoxyphenyl) chroman-4-one	CP1
2	Citromitin	CP2
3	Phellopterin	FF9
4	methyl icosa-11,14-dienoate	FF11
5	(2R,3R)-3-(4-hydroxy-3-methoxy-phenyl)-5-methoxy-2-methylol-2,3-dihydropyrano[5,6-h] [1,4] benzodioxin-9-one	FF1
6	11-hydroxy-sec-o-beta-d-glucosylhamaudol_qt	FF2
7	anomalin	FF3
8	Decursin	FF12
9	Phaseolinisoflavan	GC22
10	Licochalcone B	GC25
11	3,22-Dihydroxy-11-oxo-delta (12)-oleanene-27-alpha-methoxycarbonyl-29-oic acid	GC44
12	isorhamnetin	GC7
13	3'-Hydroxy-4'-O-Methylglabridin	GC63
14	(2R)-2-[(5R,10S,13R,14R,16R,17R)-16-hydroxy-3-keto-4,4,10,13,14-pentamethyl-1,2,5,6,12,15,16,17-octahydrocyclopenta[a]phenanthren-17-yl]-5-isopropyl-hex-5-enoic acid	FL8
15	7,9(11)-dehydropachymic acid	FL3
16	Cerevisterol	FL4
17	Izoteolin	CW7
18	Heptyl phthalate	WLX3
19	8β-ethoxy atractylenolide III	BZ1
20	α-Amyrin	BZ2
21	12-senecioyl-2E,8E,10E-atractylentriol	BZ5
22	palmatine	NX14
23	6,8-Dihydroxy-7-methoxyxanthone	HZ1
24	rhein	HZ5
25	baicalein	NX9
26	Aspidinol	GJ7
27	14-acetyl-12-senecioyl-2E,8E,10E-atractylentriol	BZ4
28	Japonine	XD4
29	delta4,16-Androstadien-3-one	CHW2
30	taxifolin	GZ3
31	[(2R)-2,3-dihydroxypropyl] (Z)-octadec-9-enoate	YYR2
32	hederagenin	A1
33	(3S,8S,9S,10R,13R,14S,17R)-10,13-dimethyl-17-[(2R,5S)-5-propan-2-yloctan-2-yl]-2,3,4,7,8,9,11,12,14,15,16,17-dodecahydro-1H-cyclopenta[a]phenanthren-3-ol	B1
34	formononetin	D1
35	Calycosin	D2

36	Vestitol	D4
37	Stigmasterol	E1
38	luteolin	F2

Supplementary Table S12. GMFA extended gene list (GMFA-ED)

No	Symbol	No	Symbol
1	TNFRSF1A	50	CREB5
2	TRADD	51	MAPK8
3	TNFRSF1B	52	ATF2
4	ADAM17	53	FOSB
5	RIPK1	54	MAPK9
6	TRAF2	55	SENP2
7	CHUK	56	CXCR4
8	HNRNPA1	57	PSIP1
9	TNFAIP3	58	CD164
10	FADD	59	CXCL12
11	TNF	60	RNF113A
12	IL6R	61	TIR2
13	IL6ST	62	SLC1A1
14	SOCS3	63	RGS1
15	ZC3H12B	64	ADRA1A
16	OSM	65	ADRB1
17	PTGS2	66	CCR5
18	CXCR2	67	EGFR
19	CXCL8	68	PIK3CA
20	SELE	69	ANXA2
21	IL1B	70	PIGS
22	IL6	71	ADAM12
23	MMP9	72	LRIG1
24	TIMP1	73	EGF
25	LCN2	74	CAV2
26	CXCL5	75	ABL1
27	CXCR6	76	BLK
28	COL12A1	77	TGFA
29	MMP12	78	CCL5
30	THBS2	79	CCR1
31	CD44	80	CCL16
32	MEP1A	81	CCL27
33	ELANE	82	CCL2
34	PTPRC	83	CCL24
35	PTPRCAP	84	CCL25

36	SEMA4D	85	CCL20
37	ITGAL	86	ACKR1
38	LCK	87	CXCL9
39	DPP4	88	PPARG
40	PAG1	89	NR2F2
41	CD2	90	NCOA1
42	CD22	91	NCOA2
43	CD3D	92	HELZ2
44	SKAP1	93	RXRA
45	JUN	94	ACTN4
46	FOS	95	FABP4
47	ATF3	96	KLF5
48	IGFBP7	97	GSTA2
49	KDM6B	98	KLF15

Supplementary Table S17. 27 active ingredients of the drug-active ingredient-target network

No	MOL ID	Molecular name	CAS/ Compound CID	Structure	Degree	Source
1	MOL000296	hederagenin	142211602	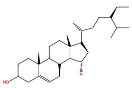	11	*Spatholobus suberectus*, *Poria cocos*
2	MOL011730	11-hydroxy-sec-o-beta-d-glucosylhamaudol_qt	163014355	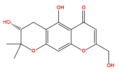	8	*Saposhnikovia divaricata*
3	MOL002714	Baicalein	491-67-8	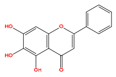	8	*Achyranthes bidentata*
4	MOL000020	12-senecioyl-2E,8E,10E-atractylentriol	113269-39-9	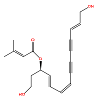	6	*Atractylodes macrocephala*
5	MOL002268	Rhein	478-43-3	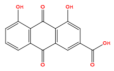	6	*Reynoutria japonica*

6	MOL011737	divaricatacid	163024659	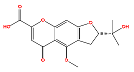	6	*Saposhnikovia divaricata*
7	MOL000028	α-Amyrin	638-95-9	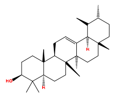	6	*Atractylodes macrocephala*
8	MOL000021	14-acetyl-12-senecioyl-2E,8E,10E-atractylentriol	113269-37-7	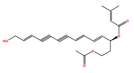	6	*Atractylodes macrocephala*
9	MOL007514	Methyl icosa-11,14-dienoate	2463-02-7		6	*Saposhnikovia divaricata*
10	MOL000011	(2R,3R)-3-(4-hydroxy-3-methoxy-phenyl)-5-methoxy-2-methylol-2,3-dihydropyrano[5,6-h][1,4]benzodioxin-9-one	76948-72-6	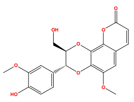	6	*Saposhnikovia divaricata*
11	MOL005603	Heptyl phthalate	3648-21-3	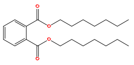	4	*Clematis chinensis*
12	MOL000033	(3S,8S,9S,10R,13R,14S,17R)-10,13-dimethyl-17-[(2R,5S)-5-propan-2-yloctan-2-yl]-2,3,4,7,8,9,11,12,14,15,16,17-dodecahydro-1H-cyclopenta[a]phenanthren-3-ol	64997-52-0	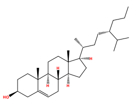	4	*Spatholobus suberectus, Atractylodes macrocephala*
13	MOL000072	8β-ethoxy atractylenolide III	113269-35-5	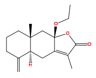	4	*Atractylodes macrocephala*
14	MOL000392	formononetin	485-72-3	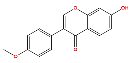	4	*Spatholobus suberectus, Glycyrrhiza uralensis*
15	MOL013077	Decursin	5928-25-6	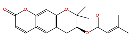	4	*Saposhnikovia divaricata*

16	MOL011732	anomalin	81740-07-0	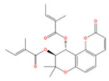	4	*Saposhnikovia divaricata*
17	MOL013281	6,8-Dihydroxy-7-methoxyxanthone	87339-74-0	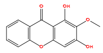	2	*Reynoutria japonica*
18	MOL002882	[(2R)-2,3-dihydroxypropyl] (Z)-octadec-9-enoate	111-03-59	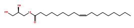	2	*Coix lacryma-jobi*
19	MOL004841	Licochalcone B	58749-23-8	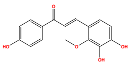	2	*Glycyrrhiza uralensis*
20	MOL008188	Japonine	30426-61-0	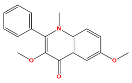	2	*Dipsacus asper*
21	MOL005815	Citromitin	12303287	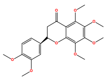	2	*Citrus reticulata*
22	MOL002087	delta4,16-Androstadien-3-one	794-58-9	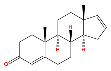	2	*Aconitum carmichaelii*
23	MOL004905	3,22-Dihydroxy-11-oxo-delta(12)-oleanene-27-alpha-methoxycarbonyl-29-oic acid	123914-44-3	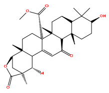	2	*Glycyrrhiza uralensis*
24	MOL005100	5,7-dihydroxy-2-(3-hydroxy-4-methoxyphenyl)chroman-4-one	520-33-2	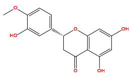	2	*Citrus reticulata*
25	MOL002644	Phellopterin	2543-94-4	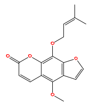	2	*Saposhnikovia divaricata*
26	MOL004576	taxifolin	480-18-2	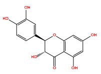	2	*Cinnamomum cassia*
27	MOL000285	(2R)-2-[(5R,10S,13R,14R,16R,17R)-16-hydroxy-3-keto-4,4,10,13,14-pentamethyl-1,2,5,6,12,15,16,17-octahydrocyclopenta[a]phenanthren-17-yl]-5-isopropyl-hex-5-enoic acid	465-18-9	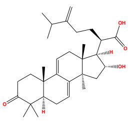	2	*Poria cocos*

Supplementary Table S18. Molecular docking binding energy results for 27 active ingredients and 10 candidate targets

	TNF (7JRA)	IL6 (4J4L)	MMP9 (8K5Y)	PTPRC (5FN7)	JUN (2P33)	CXCR4 (3ODU)	EGFR (5UGC)	CCL5 (5L2U)	PPARG (7E0A)	STAT1 (7NUF)
MOL000392	-9.5	-7.4	-8.9	-6.0	-8.4	-7.9	-8.5	-7.2	-9.2	-7.2
MOL013077	-8.6	-8.7	-10.5	-5.8	-8.9	-8.5	-8.6	-8.9	-7.6	-7.6
MOL002714	-9.2	-7.6	-9.6	-6.2	-8.2	-8.3	-8.6	-7.8	-10.1	-7.8
MOL002268	-7.7	-5.91	-9.4	-7.1	-8.7	-8.6	-8.9	-8	-10.1	-7.4
MOL004905	-8.4	-7.5	-8.7	-7.2	-8.1	-8.8	-8.6	-7.9	-8	-7.8
MOL004576	-7.6	-7.6	-9.4	-6.3	-8.3	-8.1	-8.4	-7.3	-9.7	-8.2
MOL000028	-7.8	-7.7	-7.7	-7	-8	-8.5	-8.6	-7.5	-8.3	-7.7
MOL004841	-7.9	-6.9	-9.1	-5.3	-8.1	-7.7	-7.8	-7.4	-9	-7.5
MOL002087	-8.7	-7	-8.7	-6.4	-8.2	-8.4	-7.7	-6.9	-7.6	-6.9
MOL000011	-7.4	-8	-8.2	-6.2	-8.9	-8.5	-8	-6.9	-6.9	-7.1
MOL000285	-7.3	-7.3	-7	-5.8	-7.4	-9	-8.7	-7.4	-8	-7
MOL011737	-7.1	-6.8	-7.9	-5.9	-8.2	-7.3	-7.7	-7.2	-9.8	-7
MOL013281	-6.4	-7	-9.6	-5.6	-8	-7.8	-8.3	-6.6	-9.1	-6.2
MOL011730	-7.1	-7	-9.6	-6.4	-7.9	-7.6	-7.5	-7	-6.8	-6.6
MOL000296	-7	-7.2	-7.4	-6.2	-7.1	-9	-7.5	-7.3	-7.8	-6.7
MOL002644	-9.3	-6.8	-7.8	-5.2	-8.2	-7.2	-7.7	-7.1	-6.5	-7.1
MOL011732	-6.7	-7.9	-7	-5.7	-9.2	-7.7	-7.3	-7	-7	-7.3
MOL000072	-6.8	-6.8	-8.1	-6.1	-7.9	-7.5	-6.8	-7.3	-7	-6.8
MOL005100	-8.9	-7	-7.4	-5.9	-7.4	-7.3	-6.4	-6.1	-6.9	-6.6
MOL000033	-6.7	-6.6	-7.7	-5.1	-7.3	-9	-7.7	-6	-7.3	-6.3
MOL005815	-6.3	-7.2	-7.4	-5.8	-8.1	-7.6	-7.4	-6.8	-6.1	-6.8
MOL008188	-6.8	-6.7	-7.2	-5.6	-7.7	-7.3	-7.2	-6.5	-6.1	-6.4
MOL000020	-7	-6.4	-7.3	-5	-6.6	-7.5	-7.1	-6.6	-8.3	-5.5
MOL000021	-6.8	-6.4	-7.1	-5.1	-6.7	-6.7	-7	-7.5	-7.8	-5.7
MOL007514	-7.1	-5.3	-6.8	-4.2	-5.9	-6.7	-6.3	-4.9	-7.5	-4.2
MOL005603	-7.6	-5.5	-7.1	-3.6	-4.8	-6.3	-6.9	-5.2	-5.2	-5.3
MOL002882	-6	-5.2	-6.7	-3.8	-5.7	-6	-6	-5	-7.5	-4.2

Supplementary Table S19. Performance comparison of machine learning models.

Model	Accuracy	Sensitivity	Specificity	F1-score
LASSO	0.83	0.77	0.9	0.83
SVM-RFE	0.83	0.69	1.00	0.82
RF	0.77	1.00	0.8	0.72

Supplementary Table S20. Data set information

GEO	Platform	Data sources	Tissue (Homo sapiens)	Samples (number)			Attribute	Author
				Total	HC	RA		
GSE1919	GPL91	whole-genome gene	Synovium	10	5	5	Test	U. Ungethuem (17)
GSE55235	GPL96	genome-wide transcriptomic	Synovium	20	10	10	Test	D. Woetzel (19)

Supplementary Table S21. Primers for RT-qPCR

Gene	Forward Primer (5'-3')	Reverse Primer (5'-3')
β-actin	CGGTGAAACTCTGGCTAGACAG	GCAAACCGTAGATGCTCAGGGA
IL-6	GGTGTTGCCTGCTGCCTTCC	GTTCTGAAGAGGTGAGTGGCTGTC
JUN	CCAAGAACTCGGACCTCCTCAC	CCGTTGCTGGACTGGATTATCAG
MMP9	GGCACCACCACAACATCACC	GGGCAAAGGCGTCGTCAATC
EGFR	ACAGCATAGACGACACCTTCCTC	TGGCTTGGACACTGGAGACTG
CCL5	CGCTGTCATCCTCATTGCTACTG	GCCACTGGTGTAGAAATACTCCTTG
CXCR4	ACGCCACCAACAGTCAGAGG	AAGTCGGGAATAGTCAGCAGGAG
PPARG	CCACAGGCCGAGAAGGAGAAG	CAGCGGGAAGGACTTTATGTATGAG

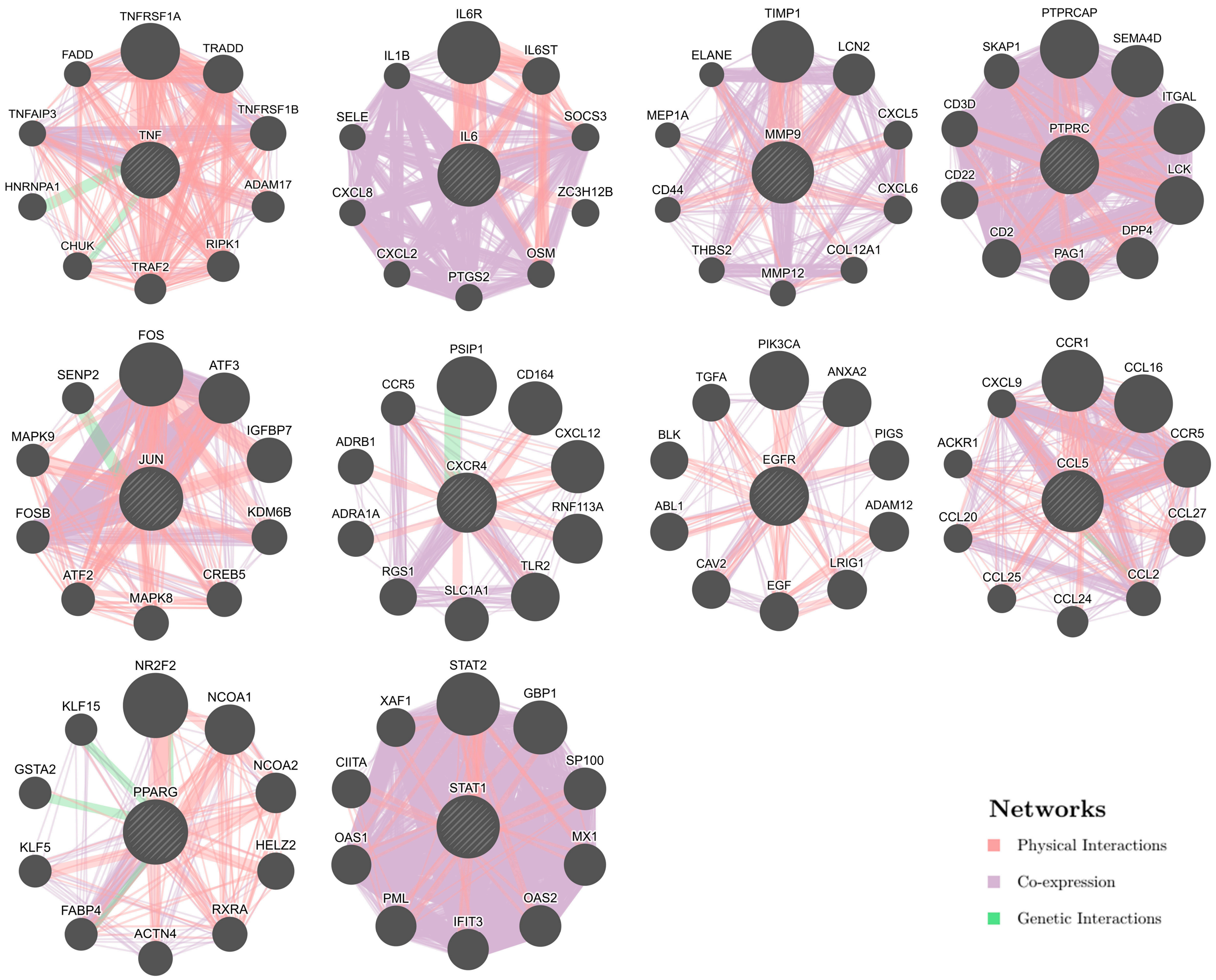

Supplementary Figure S1

Supplementary Figure S1. GeneMANIA functional association (GMFA) network analyses illustrated functionally related genes associated with 10 candidate targets and created an expanded database of potential targets for RA (GMFA-ED).

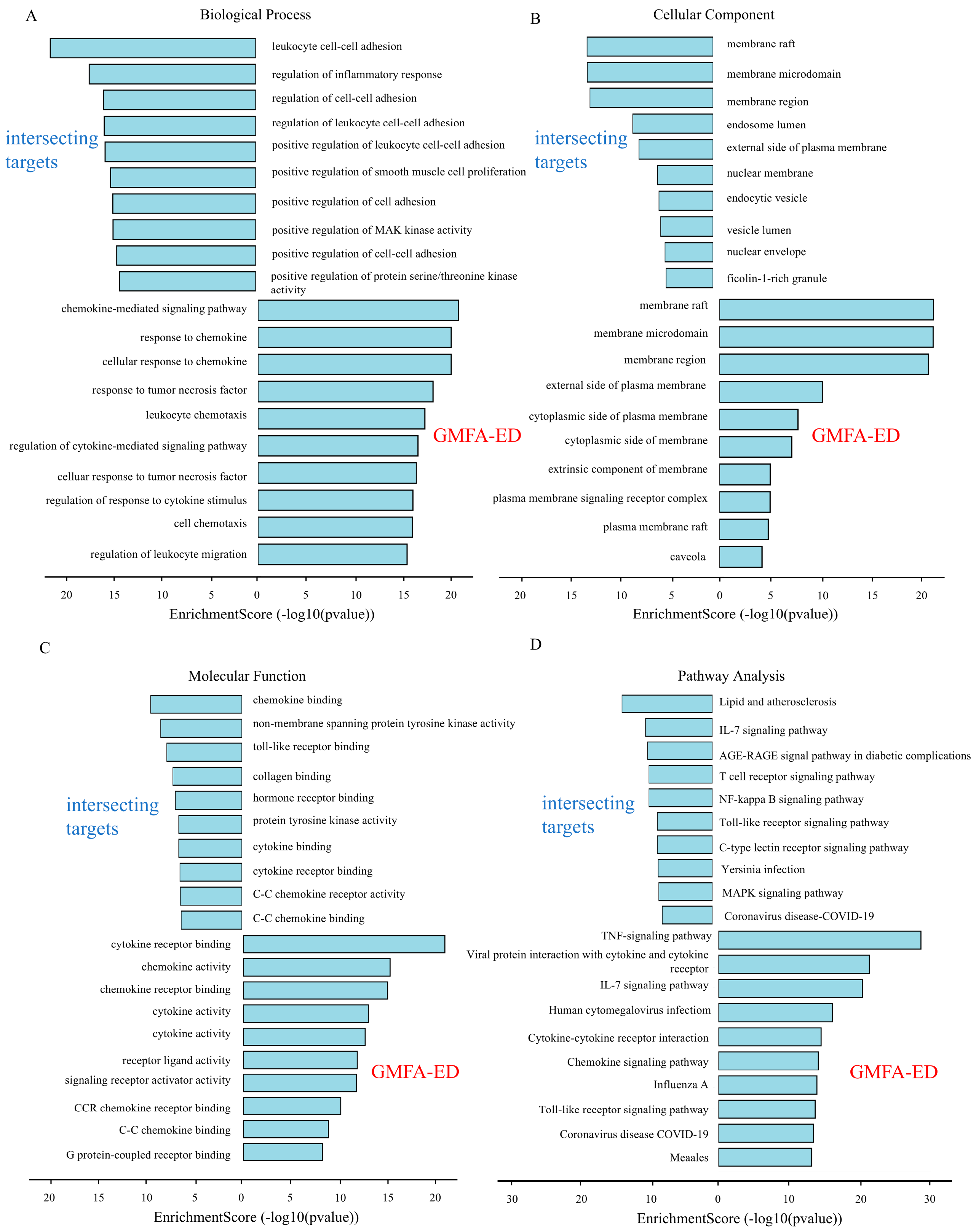

Supplementary Figure S2

Supplementary Figure S2. GO and KEGG enrichment analysis of intersecting targets and GMFA-ED targets. (A) BP terms of GO enrichment, （B）CC terms of GO enrichment, （C）MF terms of GO enrichment, （D）KEGG enrichment.
